# Identification of DNA-binding proteins using multi-features fusion and binary firefly optimization algorithm

**DOI:** 10.1186/s12859-016-1201-8

**Published:** 2016-08-26

**Authors:** Jian Zhang, Bo Gao, Haiting Chai, Zhiqiang Ma, Guifu Yang

**Affiliations:** 1 School of Computer Science and Information Technology, Northeast Normal University, Changchun, 130117 People’s Republic of China; 2 Office of Informatization Management and Planning, Northeast Normal University, Changchun, 130117 People’s Republic of China

**Keywords:** DNA-binding proteins, Binary firefly algorithm, Feature selection, Parameters optimization

## Abstract

**Background:**

DNA-binding proteins (DBPs) play fundamental roles in many biological processes. Therefore, the developing of effective computational tools for identifying DBPs is becoming highly desirable.

**Results:**

In this study, we proposed an accurate method for the prediction of DBPs. Firstly, we focused on the challenge of improving DBP prediction accuracy with information solely from the sequence. Secondly, we used multiple informative features to encode the protein. These features included evolutionary conservation profile, secondary structure motifs, and physicochemical properties. Thirdly, we introduced a novel improved Binary Firefly Algorithm (BFA) to remove redundant or noisy features as well as select optimal parameters for the classifier. The experimental results of our predictor on two benchmark datasets outperformed many state-of-the-art predictors, which revealed the effectiveness of our method. The promising prediction performance on a new-compiled independent testing dataset from PDB and a large-scale dataset from UniProt proved the good generalization ability of our method. In addition, the BFA forged in this research would be of great potential in practical applications in optimization fields, especially in feature selection problems.

**Conclusions:**

A highly accurate method was proposed for the identification of DBPs. A user-friendly web-server named iDbP (identification of DNA-binding Proteins) was constructed and provided for academic use.

**Electronic supplementary material:**

The online version of this article (doi:10.1186/s12859-016-1201-8) contains supplementary material, which is available to authorized users.

## Background

DNA-binding proteins (DBPs) are fundamental in many biological processes, such as recognition of specific nucleotide sequence, regulation of gene, transcription and translation, and DNA replication and repair [1, 2]. Thus, it is highly desirable to develop effective DBP identification methods. Traditionally, experimental techniques, which include filter binding assays [3], X-ray crystallography [4] and genetic analysis [5], are used to identify DBPs. Although these techniques can produce detailed information and provide confident assertion of the DBPs, they are both expensive and time-consuming. This spurred the development of computational methods to tackle this problem.

These computational methods can be divided into two categories: structure-based methods [6–8] and sequence-based methods [9–15]. Many of the early methods are structure based. Gao et al. [6] developed a knowledge-based method named DNA-binding Domain Hunter for identifying DBPs and associated binding sites using structural comparison. Zhao et al. [7] proposed a template-based prediction method by employing both structural similarity and binding affinity. Nimrod et al. [8] recruited random forests to identify DBPs by detecting evolutionarily conserved regions and using electrostatic features. However, the number of proteins with well annotation and good resolution structure are very limited. The structure-based methods may break down when homogeneous structures of a query protein is not available. Hence, many sequence-based methods had been proposed to deal with this problem. Kumar et al. [9] utilized various SVM modules and evolutionary information to forge the DNA-binder method. Kumar et al. [10] employed random forest to predict DBPs. Lin et al. [11] proposed the iDNA-Prot predictor by incorporating the features into the general form of pseudo amino acid composition that were extracted from protein sequence via the grey model and adopting the random forest operation engine. Song et al. [12] and Xu et al. [13] both applied the ensemble learning technique combined with hybrid features to predict DBPs. Zou et al. [14] conducted a comprehensive feature analysis of four categories of protein properties and three different feature transformation methods to find an optimal prediction model. Lou et al. [15] predicted DBPs by performing feature ranking with random forest and feature selection with forward best-first strategy. The features comprised properties from primary sequence, predicted structures and sequence alignment.

Although many efforts were put on the computational identification of DBPs, the prediction performance was still far from satisfactory. There are some possible reasons: (i) structure-based methods can provide reliable results in recognizing specific proteins. However, the insufficiency in known DBP structures leads to limited applications of these methods. Sequence-based methods are featured by their widely application, while the performance of these predictors are usually not as good as expected; (ii) the complexity of DBPs. The DBPs span over many protein families from enzymes to transcription factors [16], which makes it very difficult to describe DBPs discriminatively using mathematical models; (iii) A common approach to describe a protein in DBP prediction is by forming a feature vector, but the redundancy and contradiction among these features may seriously deteriorate the predication and generalization ability of the model.

In light of the aforementioned problems, we proposed a novel sequence-based predictor, named iDbP (identification of DNA-binding Proteins), to identify DBPs in this study. Firstly, instead of developing a narrow-application structured-based method, we focused on the challenge of sequenced-based methods. Secondly, a number of discriminative features, including evolutionary conservation, secondary structure motifs and physicochemical properties, were constructed to encode the proteins. These informative features have been proved to be associated with DNA binding interactions. Thirdly, a novel improved binary firefly algorithm (BFA) was introduced to remove redundant and noisy features as well as select optimal parameters for the classifier. In the proposed BFA, we used normalized Hamming distance to calculate attractiveness for fireflies, which greatly improved the converging rate. We also added a dynamic mutation operator to increase the diversity of fireflies. Based on the effective BFA, our predictor produced promising performance on the main dataset and two benchmark datasets. Tests on an independent testing dataset collected from PDB and a large-scale DBP dataset collected from UniProt database demonstrated the good generalization ability of iDbP.

## Methods

### Datasets

In this study, experimentally verified DBPs were collected from the Protein Data Bank (PDB, http://www.rcsb.org) by specifying keyword “DNA binding protein” and release date “before 2015-05-01” through “Advanced Search”, and 1248 sequences were obtained. Then, these sequences were pre-processed through the following procedures: (1) Sequences which contained unknown residues were discarded. (2) Sequences with less than 50 amino acid residues or belonged to fragments were removed [17]. (3) Sequences with multi-bindings were removed to avoid other influences. (4) Sequence similarity among the dataset was reduced to less than 30 % by using PISCES [18]. As a result, 455 experimentally verified DBPs were obtained as positive samples. Similarly, 455 experimentally verified non-binding proteins were also extracted from PDB with “Does not contain: DNA binding protein” as key words with less than 30 % identity. Finally, a main dataset was obtained by combining the 455 DBPs and 455 non-DBPs. This main dataset was used to find the optimal feature subset and train the iDbP prediction model. To construct the training dataset, 355 sequences were randomly picked from positive and negative samples of the main dataset, respectively. The remaining positive and negative samples were used for testing. In order to ensure unbiased and objective results, the process of under-sampling was performed 20 times. The final performance was the average prediction results of 20 experiments on different training and testing datasets.

To evaluate the effectiveness of the proposed method as well as to perform fair comparisons with previous methods [9–15], two benchmark training and testing datasets were adopted: (i) PDB594 and PDB186 [15]. The training dataset PDB594 contained 297 DBPs and 297 non-DBPs, and the testing dataset PDB186 contained 93 DBPs and 93 non-DBPs. Both PDB594 and PDB186 shared sequence similarity of less than 25 %; (ii) DNAdset and DNAiset [14]. DNAdset included 231 DBPs and 231 non-DBPs, and DNAiset contained 80 DBPs and 192 non-DBPs. The sequence similarity in DNAdset and DNAiset was less than 30 %.

In real life, the number of DBPs is much less than that of non-DBPs. To further test the generalization ability of our method, a new-compiled independent testing dataset (named DBP189) was introduced in this work. All the predictors that we compared with in this research were built before May 2015. Therefore, proteins released in PBD after May 2015 would be less likely to be used to train these models. DBP189 contained 21 DBPs and 167 non-DBPs, which were deposited in PDB between 2015-05-01 and 2016-05-01. None of these proteins shared more than 30 % sequence similarity with the main dataset. The main dataset and DBP189 were provided in Additional file 1.

### Feature vector

#### Evolutionary conservation profile

Highly conserved regions are often required for basic cellular function, stability or reproduction. Thus, evolutionary conservation analysis are often indicative of structural or functional importance [19, 20]. The position specific scoring matrix (PSSM), which carries evolutionary information of proteins, was widely used in various bioinformatics researches. In this study, the PSSM of each protein was generated by using PSI-BLAST [21] to search against the non-redundant database (ftp://ftp.ncbi.nlm.nih.gov/blast/db/nr.tar.gz) through 3 iterations with E-value of 0.0001. A *L* × 20 PSSM was generated for each protein, where *L* was the length of the sequence.

1 $$ PSSM=\left[\begin{array}{c}\hfill \begin{array}{cc}\hfill {E}_{1,1}\hfill & \hfill {E}_{1,2}\hfill \end{array}\kern1em \begin{array}{cc}\hfill \cdots \hfill & \hfill {E}_{1,20}\hfill \end{array}\hfill \\ {}\hfill \begin{array}{cc}\hfill {E}_{2,1}\hfill & \hfill {E}_{2,2}\hfill \end{array}\kern1em \begin{array}{cc}\hfill \cdots \hfill & \hfill {E}_{2,20}\hfill \end{array}\hfill \\ {}\hfill \begin{array}{cc}\hfill \vdots \kern1.25em \hfill & \hfill \vdots \hfill \end{array}\kern1.5em \begin{array}{cc}\hfill \cdots \kern0.75em \hfill & \hfill \vdots \hfill \end{array}\hfill \\ {}\hfill \begin{array}{cc}\hfill {E}_{L,1}\hfill & \hfill {E}_{L,2}\hfill \end{array}\kern1em \begin{array}{cc}\hfill \cdots \hfill & \hfill {E}_{L,20}\hfill \end{array}\hfill \end{array}\right] $$

Each score in PSSM represents whether the related substitution exceed or beneath expected frequency, and indicates whether this substitution would be favored in the process of evolution. Here, these preferences are statistical classified and analyzed by using the following formula:

2 $$ {P}_{m,n}={\displaystyle {\sum}_{m=1}^L{E}_{m,n}\times \delta}\left\{\begin{array}{c}\hfill \delta =1,\ {R}_m={a}_n\ \hfill \\ {}\hfill \delta =0,\ {R}_m\ne {a}_n\ \hfill \end{array}\right. $$

where *R*_*m*_ indicates the *m*-th (*mϵ*{1, 2, …, *L*}) residue in the protein sequence, and *a*_*n*_ (*nϵ*{1, 2, …, 20}) indicates the type of amino acid. To eliminate the influences of sequence length, *P*_*m,n*_ is normalized into the [0, 1] interval by using logistic function:

3 $$ {E}_{R_i\to {a}_i}=\frac{1}{1-{e}^{-{P}_{m,n}}} $$

Finally, feature vector $\left\{{E}_{R_i\to {a}_i}\Big|R\in \left[1,L\right],i\in \left\{1,2,\dots, 20\right\}\right\}$ was generated to construct the features of evolutionary conservation profile.

#### Secondary structure motifs

**Fig. 1:**
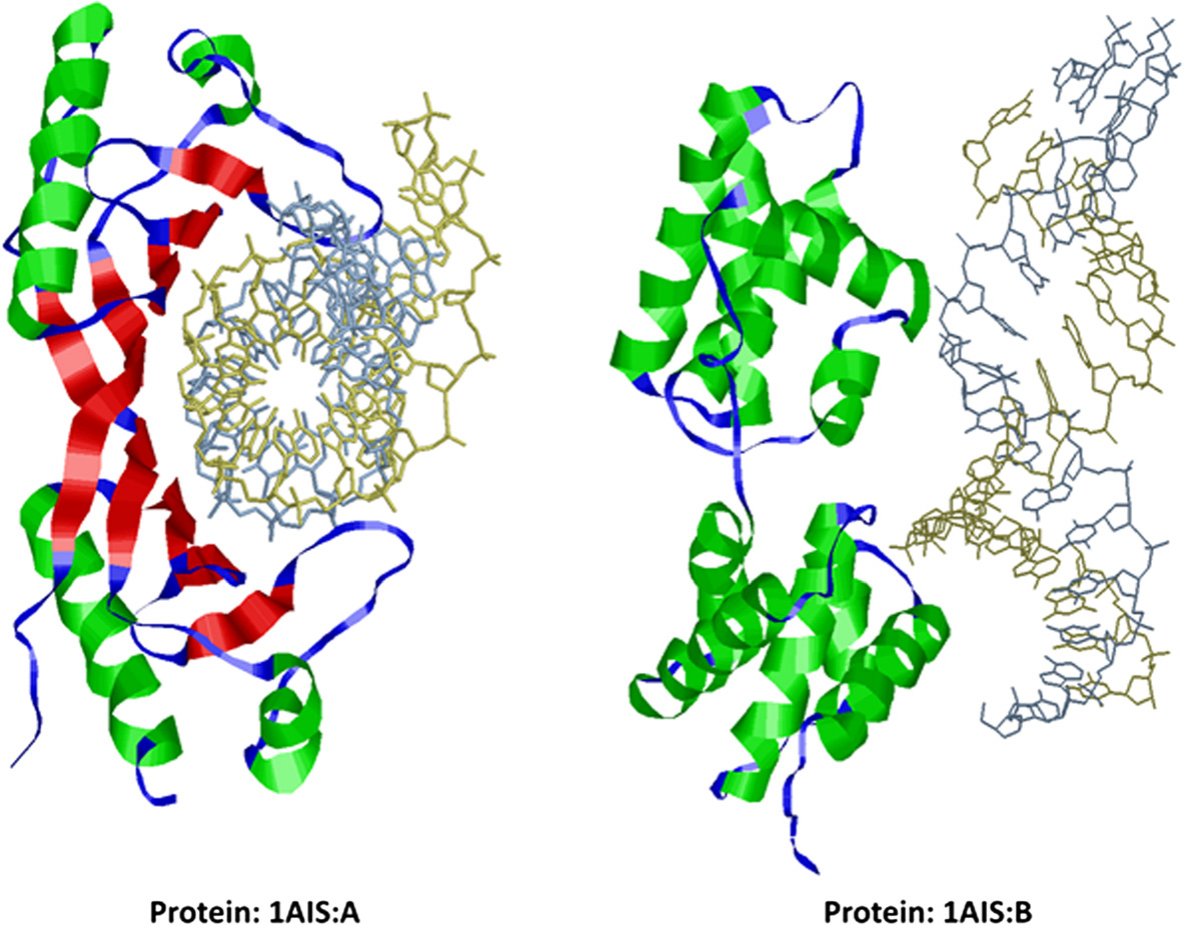
An example that illustrates the preferences of certain secondary structure motifs of a protein complex. Panel (**a**) is a TATA-binding protein (PDB ID: 1AIS_A). The binding surface is composed of strands (*red*) while the outer region is composed of helices (*green*). The general secondary structure pattern of this protein is strand-helix-strand-helix-strand-helix-strand-helix. Panel (**b**) is a transcription initialization protein (PDB ID: 1AIS_B) that is mainly composed of helices (*green*) and turns (*blue*)

**Fig. 2:**
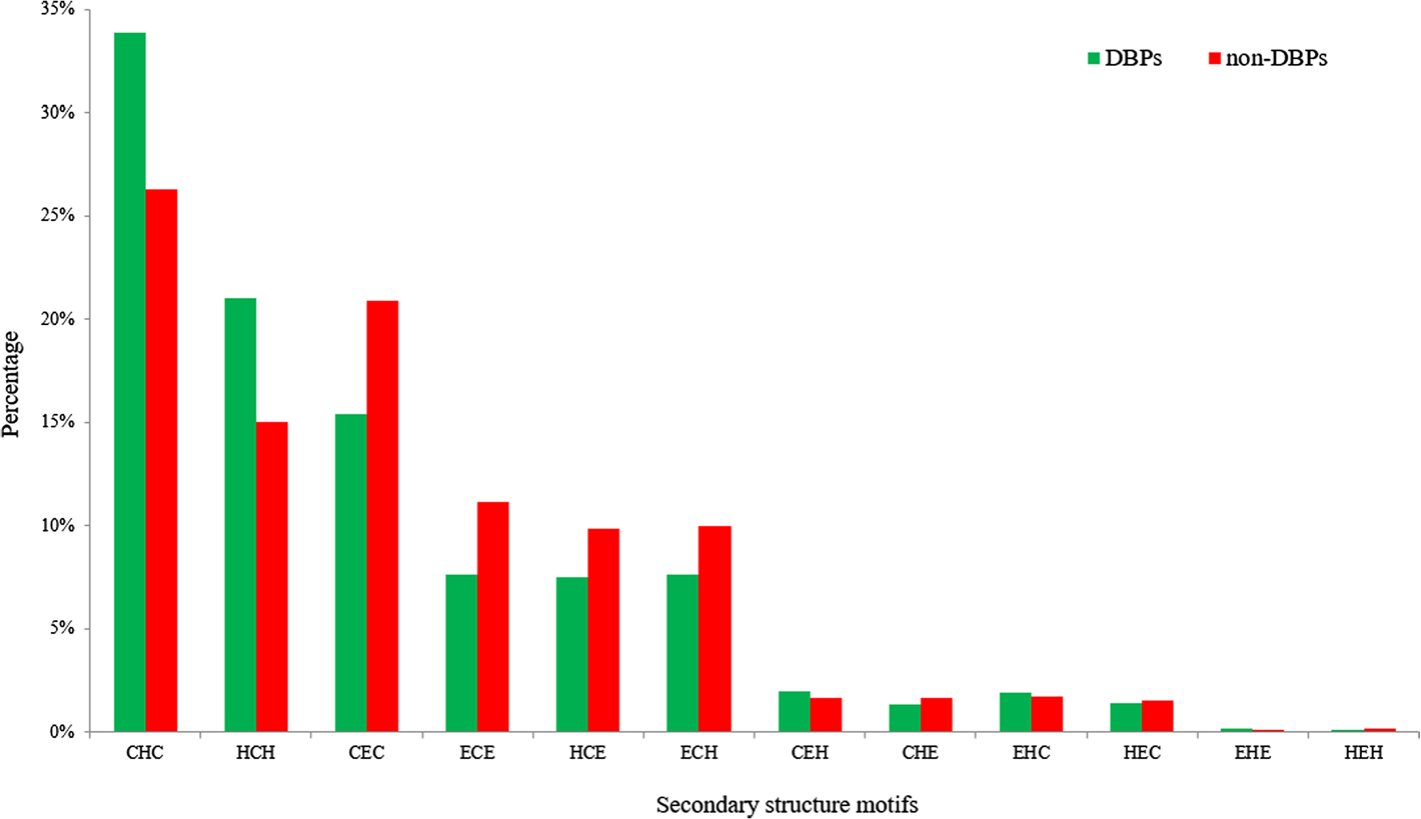
The distribution of secondary structure motifs

Secondary structure plays an important role in the function of DBPs [22]. Many DBPs show obvious preference of certain secondary structure motifs, such as helix-turn-helix and coil-helix-coil. These structures are usually solvent exposed and hydrophilic, which grant high probabilities in interaction with DNA segments [23]. Shown in Fig. 1 are the examples of DBP complexes. The secondary structure motifs repeat regularly in DBPs, and this phenomenon could be utilized to discriminate DBPs from non-binding proteins. Figure 2 shows the distributions of the secondary structure motifs on the main dataset. The over-expression of “CXC”, “HCX” and “ECX” confirms the experimental observation of enrichments of a series of helices or strands in DBPs.

To obtain secondary structure motifs, firstly, the predicted secondary structure for each residue was calculated as a probability matrix using PSIPRED [24] (Eq. (4)).

4 $$ ss\  probMarix=\left[\begin{array}{c}\hfill \begin{array}{cc}\hfill {P}_{1\to H}\hfill & \hfill {P}_{1\to E}\hfill \end{array}\kern0.75em {P}_{1\to C}\hfill \\ {}\hfill \begin{array}{cc}\hfill {P}_{2\to H}\hfill & \hfill {P}_{2\to E}\hfill \end{array}\kern0.75em {P}_{2\to C}\hfill \\ {}\hfill \begin{array}{cc}\hfill \vdots \kern0.5em \hfill & \hfill \vdots \hfill \end{array}\kern0.75em \begin{array}{cc}\hfill \kern0.75em \hfill & \hfill \vdots \hfill \end{array}\hfill \\ {}\hfill \begin{array}{cc}\hfill {P}_{L\to H}\hfill & \hfill {P}_{L\to E}\hfill \end{array}\kern0.75em {P}_{L\to C}\hfill \end{array}\right] $$

where *P*_*i* → *H*/*E*/*C*_ (*iϵ*{1, 2, …, *L*}) is the probability of the *i*-th residue to be part of a helix (H), strand (E) or coil (C). Next, *max*(*P*_*i* → *H*/*E*/*C*_) for each position would be selected as the corresponding secondary structure, and secondary structure segments were generated to represent the secondary structure distribution for the protein. Then, the secondary structure motifs were obtained from the segments:

5 $$ ss\  motif={\displaystyle \sum}\left\{se{g}_{\alpha }se{g}_{\beta }se{g}_{\gamma}\right\} $$

where *seg*_*α*/*β*/*γ*_ indicates continuous secondary structure segments of the same type and *α*, *β*, *γ* ∈ {*H*, *E*, *C*}. Finally, a protein was encoded by a 12-dimentional feature vector.

#### Physicochemical properties

Physiochemical properties reveal macroscopic phenomena among atoms and molecules such as motions, energy, force and dynamics [25]. For instance, Surendra et al. [26] pointed out that hydrophobic and polar residues contributed the bonds across the interfaces and binding residues were strongly correlated with exposed surface area. Solvation free energy [27] and transfer free energy [28], which helped to form small paths, were vital free energy to the hot spots. In addition, graph shape also played an important role in deciding the functional sites on the protein surface [29]. In this study, fourteen physiochemical properties, namely net charge [30], hydrophobicity [31], hydrophilicity [27], polarity [32], polarizability [33], solvation free energy [27], graph shape index [34], transfer free energy [28], amino acid composition [35], correlation coefficient in regression analysis [36], residue accessible surface area [37], partition coefficient [38], entropy of formation [39], and pKa values of side chain [40], were collected and used. In this encoding scheme, each property were first calculated by taking the sum of its value over the residues on the whole sequence. Then, the summarized value of each property was divided by the length of the sequence [41].

### Support vector machine

Support vector machine (SVM) is a machine learning technique derived from statistical learning theory first proposed by Vapnik [42]. It was successfully applied in many bioinformatics problems and yielded promising results. In this study, we utilized the LIBSVM toolset [43] and chose Radial Basis Function (RBF) as the kernel function. Two parameters c and *γ* of SVM were optimized using BFA. All feature descriptors were normalized into the [0, 1] interval by using logistic function.

### The proposed binary firefly algorithm

#### Continuous firefly algorithm

The continuous Firefly Algorithm (FA) is a swarm-intelligence and meta-heuristic optimization algorithm developed by Xin-She Yang in 2007 [44]. FA is based on the idealized behavior of the flashing characteristics of the fireflies. It is featured by its efficiency as well as robustness. As a novel meta-heuristic algorithm, FA has been proved to be able to find almost optima in continuous problems [45]. In essence, the idea of FA can be abstracted into the following three rules [46]:

(i) Every firefly has its own lightness and could be attracted by other fireflies;
(ii) The brightness and distance determine the attractiveness. That is, a brighter firefly will always attract its adjacent less bright ones. The attractiveness will decline if the distance between two fireflies increases. If a firefly cannot find a brighter firefly within the designated distance, it will make random movements;
(iii) The brightness of a firefly is referred as light intensity (*I*), which is defined as:

6 $$ I=F\left(f(x),\beta \right) $$

where *f*(*x*) is the objective function. The attractiveness *β* is proportional to *I*, and is defined as:

7 $$ \beta ={\beta}_0{e}^{-\gamma {r}^2} $$

where *β*_0_ is the attractiveness at *r = 0*; *γ* denotes the light absorption coefficient; and *r* represents the distance between any two fireflies. The movement of a firefly *x*_*i*_ attracted to another firefly *x*_*j*_ is defined as:

8 $$ {x}_i = {x}_i+\beta \left({x}_j-{x}_i\right)+\alpha {\varepsilon}_i $$

where *α* is the randomization parameter, and *ε*_*i*_ is an element of a vector drawn from random Gaussian or uniform distributions.

#### Binary firefly algorithm

The original FA is designed for continuous problems, which means that the outcome of the objective function (i.e. the brightness of a firefly) must lie in a continuous interval. Recently, several BFA were developed to solve discrete problems, such as scheduling, timetabling and combination. Compared with the original FA, BFA obeyed similar fundamental principles while redefined distance, attractiveness, or movement of the firefly [47–49]. Palit et al. [47] applied BFA to discover the plaintext from the cipher text. Sayadi et al. [48] defined a new firefly position and applied BFA to manufacture cell formation. Poursalehi et al. [49] introduced a new form of movement of fireflies to global best in each iteration, and applied BFA on fuel reload design of nuclear reactors. In this study, a novel improved BFA was proposed for feature selection as well as parameter optimization.

The feature selection task is a typical combination problem in essence. That is, to select an optimal combination of features from a given feature space. By using this optimal subset, the machine learning algorithm could produce the best predictive performance. Every feature must be either in or not in this subset. Theoretically, for an *n*-dimensional feature space, there will be 2^n^ possible solutions (NP-hard problem). Empirically, meta-heuristic algorithms will perform better than traditional filter or wrapper methods [50]. In BFA, every firefly represents a subset of the feature space and a group of parameters (i.e., a possible solution for the problem). The effectiveness of BFA is determined by two factors: the ability to converge to the potential global optimum rapidly and the capability of jumping out of local optima. In this work, normalized Hamming distance was used to calculate attractiveness and improve converging rate in feature selection; dynamic mutation operator was introduced to increase the diversity of fireflies. The pseudo code of BFA is provided in Algorithm 1.

- **Firefly representation**

In BFA, a binary string is used to encode a firefly. Every element in the string is either 0 or 1, the length and interpretation for the string are both problem specific. That is, a firefly *X* is defined as the following:

9 $$ X={x}_1{x}_2{x}_3\dots {x}_n\kern0.5em  where\ {x}_i\in \left\{0,1\right\} $$

**Fig. 3:**
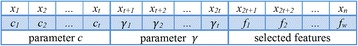
The coding scheme for a firefly

Figure 3 shows an instance of the definition of a firefly *X* with a length of *n*. The string is divided into three parts. The first part (*t* elements) and second part (*t* elements) are used to represent the values of parameters c and γ of SVM, respectively. The third part represents the features. Its length *w* is the same as the dimension of the feature space. In this part, 1 denotes the corresponding feature is selected, and 0 indicates the opposite.

b. **The attractiveness of a firefly**

**Fig. 4:**
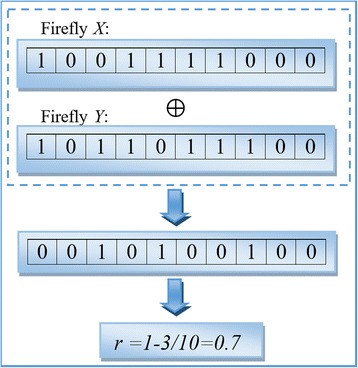
An Example of calculating parameter *r*. Firefly *X* = {1 0 0 1 1 1 1 0 0 0}, Firefly *Y* = {1 0 1 1 0 1 1 1 0 0}. The distance or difference is calculated by *X* ⊕ *Y* operation and equals {0 0 1 0 1 0 0 1 0 0}. Finally, the similarity ratio of between *X* and *Y* is *r*. -(3/10) = 0.7

Similar to FA, a firefly in BFA is also attracted by brighter fireflies. However, the attractiveness is not only determined by the brightness but also greatly affected by the similarity between fireflies. In BFA, the attractiveness *β* between a pair of fireflies is defined as $\beta ={\beta}_0{e}^{-\gamma {r}^2}.$ Here, *γ* controls the impact of *β* in the movement function; *r* determines the stride of the firefly movement. For two fireflies *X*_*i*_ and *X*_*j*_, *r* is defined based on the similarity ratio of the two fireflies (or the normalized Hamming distance of two vectors) as follows:

10 $$ r = 1-\frac{{\displaystyle {\sum}_{k=1}^n}\left|{X}_i^k\oplus {X}_j^k\right|}{n} $$

where ⊕ denotes the XOR operation, *n* is the length of *X*. Mathematically, the less identical bits two fireflies share, the greater stride a firefly would take and the more likely it would move towards the brighter one. *β* is the probability of a hetero-bit in the moving firefly changes to the corresponding bit in the brighter firefly (0 → 1 or 1 → 0). Compared with Cartesian distance and Euclidean distance, the normalized Hamming distance performs best in keeping good feature as well as removing bad ones, and also made the algorithm converge fast. Figure 4 demonstrates an example of calculating parameter *r*.

c. **The movement of a firefly**

**Fig. 5:**
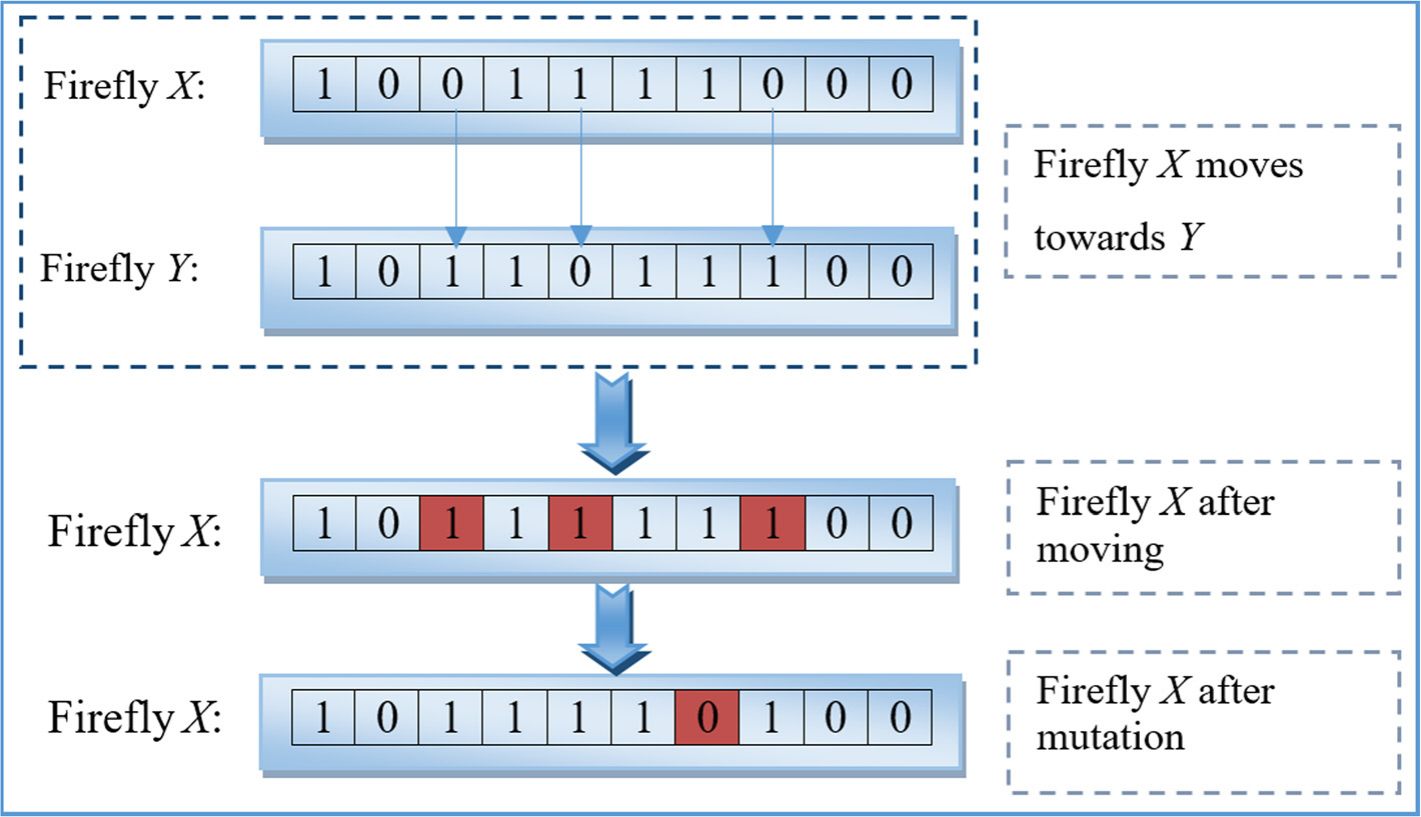
An example of movement and mutation for a firefly

When a firefly moves, every bit in its representation string will make a decision to move (change its value) or not. The decision is determined after two actions: the attraction, which is regulated by the attractiveness (*β*); and the mutation, which is controlled by a parameter (α). The movement of a bit *X*_*i*_^*k*^ in firefly *X*_*i*_ moving towards the corresponding bit *X*_*j*_^*k*^ in firefly *X*_*j*_ is defined as follows:

11 $$ {X}_i^k=g\left(f\left({X}_i^k,{X}_j^k,\beta \right),\alpha \right) $$

12 $$ f\left({X}_i^k,{X}_j^k,\beta \right)=\left\{\begin{array}{c}\hfill {X}_j^k,\kern1.25em \hfill \\ {}\hfill {X}_i^k,\kern1.25em \hfill \end{array}\begin{array}{c}\hfill if\ {X}_i^k\ne {X}_j^k\  and\  rand\left(0,1\right)<\beta \hfill \\ {}\hfill otherwise\kern8.5em \hfill \end{array}\right. $$

13 $$ g\left({X}_i^k,\alpha \right)=\left\{\begin{array}{c}\hfill 1-{X}_i^k,\kern1em \hfill \\ {}\hfill {X}_i^k,\kern3.25em \hfill \end{array}\begin{array}{c}\hfill if\  rand\left(0,1\right)<\alpha \hfill \\ {}\hfill otherwise\kern3.25em \hfill \end{array}\right. $$

14 $$ \alpha =0.5-\frac{0.5\times Iteration}{Max\  Iteration} $$

where the inner function *f*(*x,y,x*) of (Eq.11) regulates the attracted movement of bit *X*_*i*_^*k*^ to *X*_*j*_^*k*^, and the outer function *g*(*x, α*) regulates the random moving behavior (mutation) of *X*_*i*_^*k*^. It should be noted that an attracted movement would incur only when the two corresponding bits are different, while the mutation might occur on every bit with the same probability. The introduction of dynamic mutation operator grants the firefly the ability to escape from a local optimum and check nearby regions while flying. In this work, parameter *α* controls the probability of mutation. The mutation probability is high in initial iterations, which makes BFA focus on exploration. As the number of iteration increases, the mutation probability will decrease, and BFA will accelerate its converging pace gradually. Figure 5 demonstrates an example of firefly movement. If a firefly is attracted by another, each different bit in the attracted firefly would change with probability β. Then each bit in the new firefly mutates with probability α.

### Statistic inference and performance evaluation

**Fig. 6:**
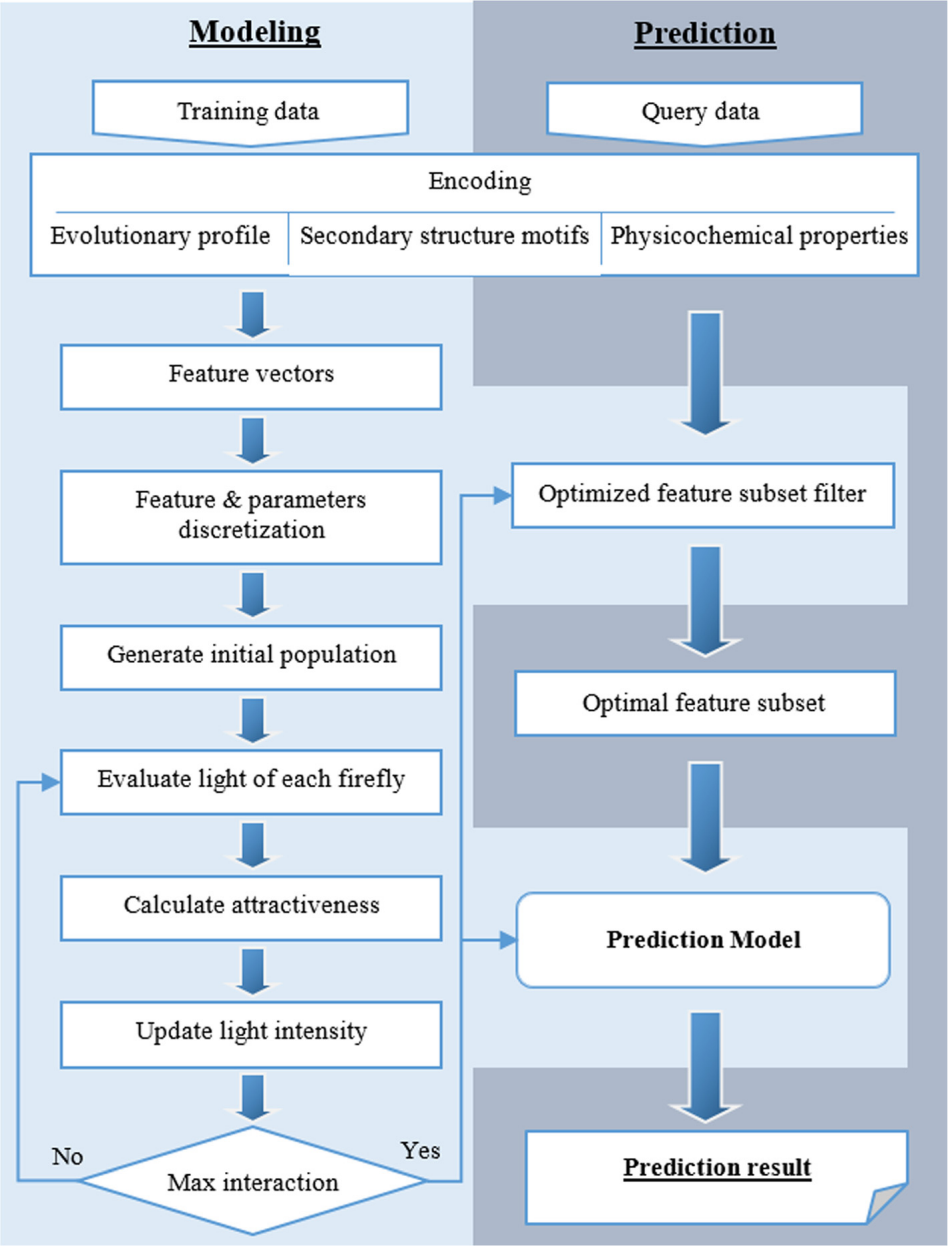
The flowchart of proposed method

Five indices were employed to measure the performance of our method. These indices included sensitivity (SN), specificity (SP), accuracy (ACC), and Matthews’s correlation coefficient (MCC):

15 $$ SN=\frac{TP}{TP+FN} $$

16 $$ SP=\frac{TN}{TN+FP} $$

17 $$ ACC=\frac{TP+TN}{TP+TN+FP+FN} $$

18 $$ MCC=\frac{TP\times TN-FN\times FP}{\sqrt{\left(TP+FN\right)\times \left(TP+FP\right)\times \left(TN+FP\right)\times \left(TN+FN\right)}} $$

where TP, FP, TN, and FN were the abbreviations of true positive, false positive, true negative, and false negative, respectively. The area under the receiver operating characteristic curve (ROC-AUC) was carried out when we assessed our method with other feature selection methods. The performance was evaluated by using leave-one-out cross-validation on the main dataset and selected optimal feature subset and parameters. Finally, the workflow of our method is shown in Fig. 6.

## Results and discussion

### The performance of the proposed method

The proposed method was implemented by combining informative features and optimizing parameters using BFA based on SVM. The settings of BFA were tuned as the following: the number of fireflies was set to 30; the visibility γ was set to 1; and the maximum iterations was set to 500. The light intensity was defined as follows:

19 $$ I=\omega \times MCC+\left(1-\omega \right)\times \left(1-\frac{n}{N}\right) $$

where *n* was the number of selected features, N was the total number of features, and ω was the weighting coefficient that controlled the trade-off between the prediction accuracy and the selected features. Usually, the weighting coefficients of an algorithm are determined empirically. In our research, ω was set as 0.55. Here, MCC was used as the key criterion to evaluate the performance of a feature subset, as it could provide balanced and unbiased measurement of the prediction ability of the model. $\frac{n}{N}$ was used to assess the number of selected features. This experiment was repeated 20 times. The final performance was the average of the 20 results. The experiment with the medium value of MCC were chosen and the corresponding optimal feature subset and parameters were used to build the iDbP prediction model. The following experiments were all based on the selected optimal feature subset and parameters. Finally, the proposed method achieved a promising performance with the mean MCC of 0.595, ACC of 0.795, SN of 0.863, SP of 0.726 on the main dataset.

### Comparison with other feature selection techniques

Feature selection is an important technique in predictive modeling. By removing redundant features, it can considerably improve the prediction accuracy. In this section, we compared BFA with several popular feature selection techniques: binary particle swarm optimization (BPSO) [50], genetic algorithm (GA) [51], minimum redundancy maximum relevance [52] combined with incremental feature selection (mRMR + IFS) [41], the original FA [44], and the straightforward method with all features.

PSO is a meta-heuristic algorithm that optimizes a problem by searching optimal particle (candidate solution). The position and velocity of the particle vary in each iteration to approach the best position (global optimum). BPSO is the binary version of PSO. GA is a classic intelligent algorithm that emulates genetic evolution. It uses binary representation in nature and is good at discrete optimizations. mRMR + IFS is a combined feature selection scheme. It firstly sorts the features with criteria of minimum redundancy maximum relevance. Then, it iteratively uses the first *n* ranked features to build models to find the best feature subset. For the original FA, which should only be used in continuous problems, the binary string of the feature vector was transferred to decimal values. All these methods were embedded with SVM and run 20 times on the main dataset using exactly the same procedure. The final performance for each method were the average performance of 20 results.

Table 1 lists the detailed results of five feature selection methods and the straightforward method with all features. Compared with simple feature fusion or filter feature selection, the meta-heuristic algorithms were more effective in selecting the optimal feature subsets. In addition, the FA produced an unsatisfactory performance, which proved that it was not suitable for discrete problem. Among the three meta-heuristic algorithms, BFA outperformed other methods with the highest MCC of 0.595.

**Table 1:** Comparison of BFA with different feature selection methods

Method	SN	SP	ACC	MCC
BFA	0.863	0.726	0.795	0.595
BPSO	0.830	0.710	0.770	0.544
GA	0.840	0.680	0.760	0.527
FA	0.720	0.610	0.665	0.332
mRMR + IFS	0.790	0.640	0.715	0.435
All features	0.680	0.760	0.600	0.365

**Fig. 7:**
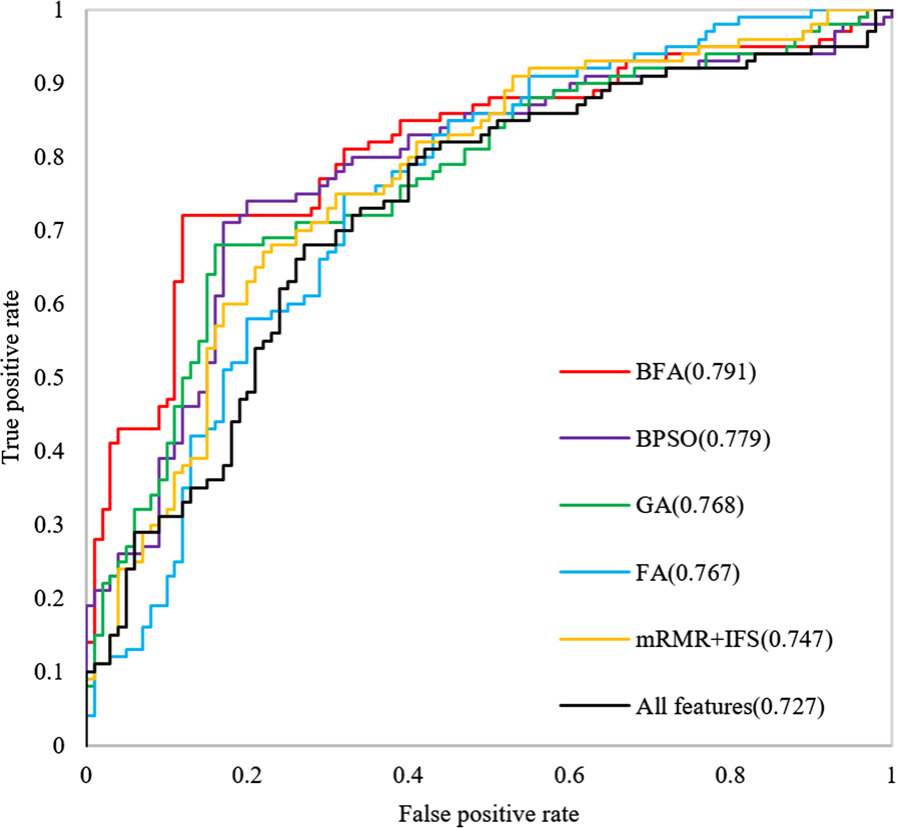
ROC curves of different feature selection methods

To assess the robustness of our BFA, we further drawn ROC curves for each method using the leave-one-out cross-validation on the main dataset. With all features, the predictor gave an AUC of 0.727. The mRMR + IFS scheme gave an AUC of 0.767. Additionally, the heuristic feature selection algorithms achieved better performance, an AUC of 0.747 for FA, an AUC of 0.768 for GA and an AUC of 0.779 for BPSO (Fig. 7). The newly proposed BFA produced an AUC values of 0.791, which was the highest among these feature selection methods. In our research, the BFA takes about 90 min to complete one entire experiment on a PC with a 3.20 GHz Intel Xeon CPU and 8GB RAM. Further improvement can be achieved by parallel computation, which is almost 4 times faster by computing 6 fireflies concurrently.

### Comparison with existing methods

#### Comparison with other predictors on benchmark datasets

In recent years, several methods were proposed to identify DBPs. These methods included DNAbinder [9], iDNA-Prot [11], enDNA-Prot [13], nDNA-Prot [12], DBPPred [15], DBD-Threader [53] and Zou’s method [14]. Among these methods, DNAbinder, iDNA-Prot, enDNA-Prot, nDNA-Prot, DBPPred and Zou’s method were sequence-based methods. To ensure a fair comparison with previous studies, the training dataset PDB594 of DBPPred was adopted to train iDbP and the independent testing dataset PDB186 was used to evaluate our predictor and compare with previous studies. Listed in Table 2 are the results of the comparison. Our iDbP achieved the highest SN of 0.894, ACC of 0.809 and MCC of 0.625. Additionally, we also compared the AUC value of iDbP with these predictors. As the AUC scores for iDNA-Prot, DNA-Prot, enDNA-Prot, nDNA-Prot, and DBD-Threader were unavailable, the comparisons were performed among DBPPred, DNAbinder, DNABIND and iDbP. The DBPPred, DNAbinder, DNABIND produced the AUC scores of 0.791, 0.607 and 0.694. Our iDbP yielded the highest AUC score of 0.803, which was slightly better than DBPPred.

**Table 2:** Comparison of iDbP with existing methods on dataset PDB186

Method	SN	SP	ACC	MCC
iDbP	0.894	0.722	0.809	0.625
DBPPred	0.796	0.742	0.769	0.538
iDNA-Prot	0.677	0.667	0.672	0.344
nDNA-Prot	0.710	0.623	0.667	0.335
enDNA-Prot	0.602	0.699	0.651	0.303
DNA-Prot	0.699	0.538	0.618	0.240
DNAbinder	0.570	0.645	0.608	0.216
DBD-Threader	0.237	0.957	0.597	0.279

Similarly, the training dataset DNAdset from Zou’s method was adopted to train iDbP and the independent testing dataset DNAiset was used to evaluate iDbP and compare with previous studies. As the services of DBPPred and DBDThreader were not availiable. The comparison on Zou’s benchmark dataset was performed among iDNA-Prot, DNAbinder, enDNA-Prot, nDNA-Prot, Zou’s method and our iDbP. As shown in Table 3, the iDbP yielded the best performance with the SN of 0.908, SP of 0.911, ACC of 0.910 and MCC of 0.803.

**Table 3:** Comparison of iDbP with existing methods on dataset DNAiset

Method	SN	SP	ACC	MCC
iDbP	0.908	0.911	0.910	0.803
Zou’s method	0.890	0.828	0.900	0.753
iDNA-Prot	0.875	0.798	0.837	0.709
nDNA-Prot	0.779	0.887	0.851	0.664
enDNA-Prot	0.760	0.868	0.832	0.623
DNAbinder	0.717	0.642	0.863	0.473

Theoretically, protein structures could provide more information than primary sequences. However, our experiments showed that the sequence-based method could produce approximate or even better results. In general, the sequence-based methods are significant supplements for the structure-based methods, especially when the high-resolution 3D structures or the homology templates of the query proteins are hard to obtain.

#### Comparison with other predictors on DBP189 dataset

To demonstrate the generalization ability of our iDbP, we performed further comparisons with previous methods on DBP189. Three DBP prediction tools, namely DNA-Prot, iDNA-Prot and DNAbinder, still provided online or local prediction services. The prediction results (shown in Table 4) on the DBP189 dataset indicated that our method still characterized by good predictive performance on imbalanced testing dataset. Among these methods, our iDbP achieved the highest MCC of 0.5996, which was about 5 % more than the second highest method DNA-Prot.

**Table 4:** Comparison of predictive quality on the DBP189 dataset

Method	SN	SP	ACC	MCC
iDbP	0.7619	0.9162	0.8989	0.5996
DNA-Prot	0.7143	0.9042	0.8830	0.5415
iDNA-Prot	0.6190	0.8563	0.8298	0.3960
DNAbinder	0.5714	0.8263	0.7979	0.3234

### Application to large-scale DBP prediction

In real-life application, computational tools are often used to identify possible candidate proteins in large scale. To simulate this scenario, we collected 15,413 DBPs from five most popular organisms (human, A. thaliana, mouse, S. cerevisiae and fruit fly) in UniProt database. After removing incomplete segments and unannotated proteins, we finally obtained a large-scale testing dataset with 2859 DBPs (Provided in Additional file 2). As shown in Table 5, by using our iDbP, nearly 59 % of human proteins, 53 % of A. thaliana proteins, 54 % of Mouse proteins, 61 % of S. cerevisiae proteins, and 59 % of Fruit fly proteins were successfully predicted as DBPs. In summary, about 56 % proteins were successfully recognized. The results showed that iDbP could be a reliable tool in large-scale applications.

**Table 5:** Number of annotated and recognized DBPs in UniProt database

Category	Number of proteins	Proteins with complete DNA binding annotations	Number of predicted	SN
			DBPs	
Human	6,813	1,049	613	58 %
A. thaliana	3,378	929	489	53 %
Mouse	2,514	424	232	54 %
S. cerevisiae	1,545	314	191	61 %
Fruit fly	1,163	143	84	59 %
Summary	15,413	2,859	1609	56 %

## Conclusion

In this work, we proposed a new method, named iDbP, to predict DBPs from primary sequence. Multiple informative features, which derived from evolutionary conservation profile, secondary structure motifs and physiochemical properties, were used to discriminate DBPs from non-binding proteins. Next, a novel improved BFA was forged to perform feature selection and parameter optimization. The experimental results of our predictor on two benchmark datasets outperformed many state-of-the-art predictors, which revealed the effectiveness of our method. Moreover, the promising performance on an independent testing dataset and large-scale proteins from UniProt database proved the good generalization ability of our method. In addition, the novel improved BFA would be of a powerful algorithm which could find widely applications in discrete optimization problems. The web-server is available for academic research at http://59.73.198.144:8080/iDbP/.

## Additional files

Additional file 1: The main dataset and DBP189 used in this study. (PDF 751 kb) Additional file 2: The large-scale testing dataset compiled from UniProt. (PDF 477 kb)
