# Supplementary material for: Identification of DNA-binding proteins using multi-features fusion and binary firefly optimization algorithm
Source: BMC Bioinformatics. 2016 Aug 26;17(1):323. doi: 10.1186/s12859-016-1201-8 (PMC5002159; doi:10.1186/s12859-016-1201-8)
Supplement: Additional file 1: — The main dataset and DBP189 used in this study. (PDF 751 kb) [file 12859_2016_1201_MOESM1_ESM.pdf]

Additional file 1

Positive samples:

>2JULA 256 NMR NA NA NA no Calsenilin <UNP CSEN_MOUSE> [MUS MUSCULUS]
MQRTKEAVKASDGNLLGDPGRIPLSKRESIKWQRPRFTRQALMRCCLIKWILSSAAPQGS
DSSDSELELSTVRHQPEGLDQLQAQTKFTKKELQSLYRGFKNECPTGLVDEDTFKLIYSQ
FFPQGDATTYAHFLFNAFDADGNGAIHFEDFVVGLSILLRGTVHEKLKWAFNLYDINKDG
CITKEEMLAIMKSIYDMMGRHTYPILREDAPLEHVERFFQKMDRNQDGVVTIDEFLETCQ
KDENIMNSMQLFENVI
>3P9AA 162 XRAY 1.75 0.178 0.216 no DNA-packaging protein gp3 <UNP TERM_BPP22> [ENTEROBACTERIA PHAGE P22]
MAAPKGNRFWEARSSHGRNPKFESPEALWAACCEYFEWVEANPLWEMKAFSYQGEVIQEP
IAKMRAMTITGLTLFIDVTLETWRTYRLREDLSEVVTRAEQVIYDQKFSGAAADLLNANI
IARDLGLKEQSQVEDVTPDKGDRDKRRSRIKELFNRGTGRDS
>4FJOA 97 XRAY 2.72 0.202 0.236 no DNA repair protein REV1 <UNP REV1_MOUSE> [MUS MUSCULUS]
AAPNLAGAVEFSDVKTLLKEWITTISDPMEEDILQVVRYCTDLIEEKDLEKLDLVIKYMK
RLMQQSVESVWNMAFDFILDNVQVVLQQTYGSTLKVT
>4FJOC 210 XRAY 2.72 0.202 0.236 no Mitotic spindle assembly checkpoint protein MAD2B <UNP MD2L2_MOUSE> [MUS MUSCULUS]
MTTLTRQDLNFGQVVADVLSEFLEVAVHLILYVREVYPVGIFQKRKKYNVPVQMSCHPEL
NQYIQDTLHCVKPLLEKNDVEKVVVVILDKEHRPVEKFVFEITQPPLLSINSDSLLSHVE
QLLAAFILKISVCDAVLDHNPPGCTFTVLVHTREAATRNMEKIQVIKDFPWILADEQDVH
MHDPRLIPLKTMTSDILKMQLYVEERAHKN
>3U4VA 117 XRAY 1.80 0.182 0.200 no Telomerase-associated protein 82 <UNP D2CVN6_TETTH> [TETRAHYMENA THERMOPHILA]
NFNIGSLSDQLSKQTLLISQLQVGKNRFSFKFEGRVVYKSSTFQNQQDSKYFFITAQDAN
NQEINMSFWQKVDQSYQTLKVGQYYYFIGGEVKQFKNNLELKFKFGDYQIIPKETLS
>3U4ZA 109 XRAY 2.30 0.225 0.266 no Telomerase-associated protein 82 <UNP D2CVN6_TETTH> [TETRAHYMENA THERMOPHILA]
TLLISEVLKTSKQYLSVLAQVVDIQSSDKNIRLKICDNSCNQELKVVIFPDLCYEWRDKF
SINKWYYFNEFVRQIYNDEVQLKNNIHSSIKESDDQRKVITYNQEQGVF
>2O8BB 1022 XRAY 2.75 0.245 0.281 no DNA mismatch repair protein MSH6 <UNP MSH6_HUMAN> [HOMO SAPIENS]
MGSAPQNSESQAHVSGGGDDSSRPTVWYHETLEWLKEEKRRDEHRRRPDHPDFDASTLYV
PEDFLNSCTPGMRKWWQIKSQNFDLVICYKVGKFYELYHMDALIGVSELGLVFMKGNWAH
SGFPEIAFGRYSDSLVQKGYKVARVEQTETPEMMEARCRKMAHISKYDRVVRREICRIIT
KGTQTYSVLEGDPSENYSKYLLSLKEKEEDSSGHTRAYGVCFVDTSLGKFFIGQFSDDRH
CSRFRTLVAHYPPVQVLFEKGNLSKETKTILKSSLSCSLQEGLIPGSQFWDASKTLRTLL
EEEYFREKLSDGIGVMLPQVLKGMTSESDSIGLTPGEKSELALSALGGCVFYLKKCLIDQ
ELLSMANFEEYIPLDSDTVSTTRSGAIFTKAYQRMVLDAVTLNNLEIFLNGTNGSTEGTL
LERVDTCHTPFGKRLLKQWLCAPLCNHYAINDRLDAIEDLMVVPDKISEVVELLKKLPDL
ERLLSKIHNVGSPLKSQNHPDSRAIMYEETTYSKKKIIDFLSALEGFKVMCKIIGIMEEV
ADGFKSKILKQVISLQTKNPEGRFPDLTVELNRWDTAFDHEKARKTGLITPKAGFDSDYD
QALADIRENEQSLLEYLEKQRNRIGCRTIVYWGIGRNRYQLEIPENFTTRNLPEEYELKS
TKKGCKRYWTKTIEKKLANLINAEERRDVSLKDCMRRLFYNFDKNYKDWQSAVECIAVLD
VLLCLANYSRGGDGPMCRPVILLPEDTPPFLELKGSRHPCITKTFFGDDFIPNDILIGCE

EEEQENGKAYCVLVTGPNMGGKSTLMRQAGLLAVMAQMGCYVPAEVCRLTPIDRVFTRLG
ASDRIMSGESTFFVELSETASILMHATAHSLVLVDELGRGTATFDGTAIANAVVKELAET
IKCRTLFSTHYHSLVEDYSQNVAVRLGHMACMVENECEDPSQETITFLYKFIKGACPKSY
GFNAARLANLPEEVIQKGHRKAREFEKMNQSLRLFREVCLASERSTVDAEAVHKLLTLIK
EL
>3VW4A 128 XRAY 2.70 0.260 0.262 no Rep <UNP Q06B24_ECOLX> [ESCHERICHIA COLI]
MGHHHHHHRNYHLFEKVRKWAYRAIRQGWPVFSQWLDAVIQRVEMYNASLPVPLSPAECR
AIGKSIAKYTHRKFSPEGFSAVQAARGRKGGTKSKRAAVPTSARSLKPWEALGISRATYY
RKLKCDPD
>1Q1VA 70 NMR NA NA NA no DEK protein <UNP DEK_HUMAN> [HOMO SAPIENS]
DEPLIKKLKKPPTDEELKETIKKLLASANLEEVTMKQICKKVYENYPTYDLTERKDFIKT
TVKELISLEH
>1R7JA 95 XRAY 1.47 0.232 0.244 no Conserved hypothetical protein Sso10a <UNP Q5W1E8_SULSO> [SULFOLOBUS SOLFATARICUS]
AKKKSKLEIIQAILEACKSGSPKTRIMYGANLSYALTGRYIKMLMDLEIIRQEGKQYMLT
KKGEELLEDIRKFNEMRKNMDQLKEKINSVLSIRQ
>4DFCA 93 XRAY 2.80 0.240 0.282 no Transcription-repair-coupling factor <UNP MFD_ECOLI> [ESCHERICHIA COLI]
GPHMASALVMKKGQRLSRDALRTQLDSAGYRHVDQVMEHGEYATRGALLDLFPMGSELPY
RLDFFDDEIDSLRVFDVDSQRTLEEVEAINLLP
>1SFUA 75 XRAY 2.00 0.237 0.270 no 34L protein <UNP Q9DHS8_YLDV> [YABA-LIKE DISEASE VIRUS]
MDLLSCTVNDAEIFSLVKKEVLSLNTNDYTTAISLSNRLKINKKKINQQLYKLQKEDTVK
MVPSNPPKWFKNYNC
>2FE3A 145 XRAY 1.75 0.172 0.229 no Peroxide operon regulator <UNP PERR_BACSU> [BACILLUS SUBTILIS]
MAAHELKEALETLKETGVRITPQRHAILEYLVNSMAHPTADDIYKALEGKFPNMSVATVY
NNLRVFRESGLVKELTYGDASSRFDFVTSDHYHAICENCGKIVDFHYPGLDEVEQLAAHV
TGFKVSHHRLEIYGVCQECSKKENH
>4FE7A 412 XRAY 2.90 0.222 0.279 no Xylose operon regulatory protein <UNP XYLR_ECOLI> [ESCHERICHIA COLI]
MGSSHHHHHHSSGLVPRGSHMFTKRHRITLLFNANKAYDRQVVEGVGEYLQASQSEWDIF
IEEDFRARIDKIKDWLGDGVIADFDDKQIEQALADVDVPIVGVGGSYHLAESYPPVHYIA
TDNYALVESAFLHLKEKGVNRFAFYGLPESSGKRWATEREYAFRQLVAEEKYRGVVYQGL
ETAPENWQHAQNRLADWLQTLPPQTGIIAVTDARARHILQVCEHLHIPVPEKLCVIGIDN
EELTRYLSRVALSSVAQGARQMGYQAAKLLHRLLDKEEMPLQRILVPPVRVIERRSTDYR
SLTDPAVIQAMHYIRNHACKGIKVDQVLDAVGISRSNLEKRFKEEVGETIHAMIHAEKLE
KARSLLISTTLSINEISQMCGYPSLQYFYSVFKKAYDTTPKEYRDVNSEVML
>3G1CA 104 XRAY 2.20 0.206 0.237 no The TrpR like protein from Eubacterium eligens ATCC 27750 <PDB 3G1C> [EUBACTERIUM ELIGENS]
MNNKLKTQAVEQLFQAILSLKDLDEAYDFFEDVCTINEILSLSQRFEVAKMLREHRTYLD
IAEKTGASTATISRVNRSLNYGNDGYDRVFERLGMLEKESEDNK
>1PH6B 217 XRAY 2.10 0.245 0.276 no Telomere-binding protein beta subunit <UNP TEBB_OXYNO> [STERKIELLA NOVA]
PQQQSAFKQLYTELFNNEGDFSKVSSNLKKPLKCYVKESYPHFLVTDGYFFVAPYFTKEA
VNEFHAKFPNVNIVDLTDKVIVINNWSLELRRVNSAEVFTSYANLEARLIVHSFKPNLQE
RLNPTRYPVNLFRDDEFKTTIQHFRHTALQAAINKTVKGDNLVDISKVADAAGKKGKVDA
GIVKASASKGDEFSDFSFKEGNTATLKIADIFVQEKG
>2LLHA 74 NMR NA NA NA no Nucleophosmin <UNP NPM_HUMAN> [HOMO SAPIENS]
GSHMQESFKKQEKTPKTPKGPSSVEDIKAKMQASIEKGGSLPKVEAKFINYVKNCFRMTD

QEAIQDLWQWRKSL
>3PVVA 101 XRAY 2.00 0.252 0.271 no Chromosomal replication initiator protein dnaA <UNP DNAA_MYCTA> [MYCOBACTERIUM TUBERCULOSIS]
GPHMISAATIMAATAEYFDTTVEELRGPGKTRALAQSRQIAMYLCRELTDLSLPKIGQAF
GRDHTTVMYAQRKILSEMAERREVFDHVKELTTRIRQRSKR
>3IVPA 126 XRAY 2.02 0.202 0.235 no Putative transposon-related DNA-binding protein <UNP Q180H4_CLOD6> [CLOSTRIDIUM DIFFICILE]
MRKKEDKYDFRALGLAIKEARKKQGLTREQVGAMIEIDPRYLTNIENKGQHPSLQVLYDL
VSLLNVSVDEFFLPASSQVKSTKRRQLENKIDNFTDADLVIMESVADGIVKSKEVGEMAG
ENLYFQ
>3AXJA 249 XRAY 2.10 0.210 0.238 no GM27569p <UNP Q7JVK6_DROME> [DROSOPHILA MELANOGASTER]
MGSSHHHHHHSQDPMSNFVNLDIFSNYQKYIDNEQEVRENIRIVVREIEHLSKEAQIKLQ
IIHSDLSQISAACGLARKQVELCAQKYQKLAELVPAGQYYRYSDHWTFITQRLIFIIALV
IYLEAGFLVTRETVAEMLGLKISQSEGFHLDVEDYLLGILQLASELSRFATNSVTMGDYE
RPLNISHFIGDLNTGFRLLNLKNDGLRKRFDALKYDVKKIEEVVYDVSIRGLSSKEKDQQ
EEPAVPATE
>3AXJB 298 XRAY 2.10 0.210 0.238 no Translin associated factor X, isoform B <UNP Q8INE1_DROME> [DROSOPHILA MELANOGASTER]
MPKNGGAGHRNTAPRKRQIPAAQLDEDSPIVQQFRIYSNELIMKHDRHERIVKLSRDITI
ESKRIIFLLHSIDSRKQNKEKVLEEARQRLNKLIAVNFRAVALELRDQDVYQFRSSYSPG
LQEFIQAYTYMEYLCHEDAEGENETKSVSDWQAIQAVMQYVEESSQPKEEPTEGEDVQAI
AQVESPKKFQFFVDPTEYILGLSDLTGELMRRCINSLGSGDTDTCLDTCKALQHFYSGYI
SLNCQRARELWRKITTMKQSVLKAENVCYNVKVRGGEAAKWGATFDQKPADEVDEGFY
>3K0SA 799 XRAY 2.20 0.188 0.246 no DNA mismatch repair protein mutS <UNP MUTS_ECOLI> [ESCHERICHIA COLI]
SAIENFDAHTPMMQQYLRLKAQHPEILLFYRMGDFYELFYDDAKRASQLLDISLTKRGAS
AGEPIPMAGIPYHAVENYLAKLVNQGESVAICEQIGDPATSKGPVERKVVRIVTPGTISD
EALLQERQDNLLAAIWQDSKGFGYATLDISSGRFRLSEPADRETMAAELQRTNPAELLYA
EDFAEMSLIEGRRGLRRRPLWEFEIDTARQQLNLQFGTRDLVGFGVENAPRGLCAAGCLL
QYAKDTQRTTLPHIRSITMEREQDSIIMDAATRRNLEITQNLAGGAENTLASVLDCTVTP
MGSRMLKRWLHMPVRDTRVLLERQQTIGALQDFTAGLQPVLRQVGDLERILARLALRTAR
PRDLARMRHAFQQLPELRAQLETVDSAPVQALREKMGEFAELRDLLERAIIDTPPVLVRD
GGVIASGYNEELDEWRALADGATDYLERLEVRERERTGLDTLKVGFNAVHGYYIQISRGQ
SHLAPINYMRRQTLKNAERYIIPELKEYEDKVLTSKGKALALEKQLYEELFDLLLPHLEA
LQQSASALAELDVLVNLAERAYTLNYTCPTFIDKPGIRITEGRHPVVEQVLNEPFIANPL
NLSPQRRMLIITGPNMGGKSTYMRQTALIALMAYIGSYVPAQKVEIGPIDRIFTRVGAAD
DLASGRSTFMVEMTETANILHNATEYSLVLMNEIGRGTSTYDGLSLAWACAENLANKIKA
LTLFATHYFELTQLPEKMEGVANVHLDALEHGDTIAFMHSVQDGAASKSYGLAVAALAGV
PKEVIKRARQKLRELESIS
>1UKLA 876 XRAY 3.00 0.239 0.297 no Importin beta-1 subunit <UNP IMB1_MOUSE> [MUS MUSCULUS]
MELITILEKTVSPDRLELEAAQKFLERAAVENLPTFLVELSRVLANPGNSQVARVAAGLQ
IKNSLTSKDPDIKAQYQQRWLAIDANARREVKNYVLQTLGTETYRPSSASQCVAGIACAE
IPVSQWPELIPQLVANVTNPNSTEHMKESTLEAIGYICQDIDPEQLQDKSNEILTAIIQG
MRKEEPSNNVKLAATNALLNSLEFTKANFDKESERHFIMQVVCEATQCPDTRVRVAALQN
LVKIMSLYYQYMETYMGPALFAITIEAMKSDIDEVALQGIEFWSNVCDEEMDLAIEASEA
AEQGRPPEHTSKFYAKGALQYLVPILTQTLTKQDENDDDDDWNPCKAAGVCLMLLSTCCE
DDIVPHVLPFIKEHIKNPDWRYRDAAVMAFGSILEGPEPNQLKPLVIQAMPTLIELMKDP
SVVVRDTTAWTVGRICELLPEAAINDVYLAPLLQCLIEGLSAEPRVASNVCWAFSSLAEA

AYEAADVADDQEEPATYCLSSSFELIVQKLLETTDRPDGHQNNLRSSAYESLMEIVKNSA
KDCYPAVQKTTLVIMERLQQVLQMESHIQSTSDRIQFNDLQSLLCATLQNVLRKVQHQDA
LQISDVVMASLLRMFQSTAGSGGVQEDALMAVSTLVEVLGGEFLKYMEAFKPFLGIGLKN
YAEYQVCLAAVGLVGDLCRALQSNILPFCDEVMQLLLENLGNENVHRSVKPQILSVFGDI
ALAIGGEFKKYLEVVLNTLQQASQAQVDKSDFDMVDYLNELRESCLEAYTGIVQGLKGDQ
ENVHPDVMLVQPRVEFILSFIDHIAGDEDHTDGVVACAAGLIGDLCTAFGKDVLKLVEAR
PMIHELLTEGRRSKTNKAKTLATWATKELRKLKNQA
>1T23A 93 NMR NA NA NA no Chromosomal protein MC1 <UNP HMC1_METTE>
[METHANOSARCINA THERMOPHILA]
SNTRNFVLRDEDGNEHGVFTGKQPRQAALKAANRGSGTKANPDIIRLRERGTKKVHVFKA
WKEIVDAPKNRPAWMPEKISKPFVKKERIEKLE
>2GMGA 105 NMR NA NA NA no hypothetical protein Pf0610 <UNP Q8U362_PYRFU> [PYROCOCCUS
FURIOSUS]
AHHHHHHGSATRREKIIELLLEGDYSPSELARILDMRGKGSKKVILEDLKVISKIAKREG
MVLLIKPAQCRKCGFVFKAEINIPSRCPKCKSEWIEEPRFKLERK
>1I11A 81 NMR NA NA NA no TRANSCRIPTION FACTOR SOX-5 <UNP SOX5_MOUSE> [MUS MUSCULUS]
GSPHIKRPMNAFMVWAKDERRKILQAFPDMHNSNISKILGSRWKAMTNLEKQPYYEEQAR
LSKQHLEKYPDYKYKPRPKRT
>4I1KA 146 XRAY 1.60 0.120 0.166 no B3 domain-containing transcription factor VRN1 <UNP
VRN1_ARATH> [ARABIDOPSIS THALIANA]
MRGSHHHHHHGSRSKFYESASARKRTVTAEERERAINAAKTFEPTNPFFRVVLRPSYLYR
GCIMYLPSGFAEKYLSGISGFIKVQLAEKQWPVRCLYKAGRAKFSQGWYEFTLENNLGEG
DVCVFELLRTRDFVLKVTAFRVNEYV
>2JMPA 107 NMR NA NA NA no Chromosomal replication initiator protein dnaA <UNP DNAA_MYCGE>
[MYCOPLASMA GENITALIUM]
MGGGGGGMEQFNAFKSLLKKHYEKTIGFHDKYIKDINRFVFKNNVLLILLENEFARNSLN
DNSEIIHLAESLYEGIKSVNFVNEQDFFFNLAKLEENSRDTLYQNSG
>3U50C 172 XRAY 2.50 0.218 0.253 no Telomerase-associated protein 82 <UNP D2CVN6_TETTH>
[TETRAHYMENA THERMOPHILA]
QRIYSSIEEIIQQAQASEIGQKKEFYVYGNLVSIQMKNKLYYYRCTCQGKSVLKYHGDSF
FCESCQQFINPQVHLMLRAFVQDSTGTIPVMIFDQQSSQLINQIDPSIHVQEAGQYVKNC
IENGQEEIIRQLFSKLDFARFIFEIQFENKEFNNEQEIAYKVLKIEKENIKE
>2WYQA 85 XRAY 1.65 0.126 0.160 no UV EXCISION REPAIR PROTEIN RAD23 HOMOLOG A <UNP
RD23A_HUMAN> [HOMO SAPIENS]
GIPMAVTITLKTLQQQTFKIRMEPDETVKVLKEKIEAEKGRDAFPVAGQKLIYAGKILSD
DVPIRDYRIDEKNFVVVMVTKTKAG
>2XJ3A 66 XRAY 1.23 0.148 0.184 no CYLR2 SYNONYM CYTOLYSIN REPRESSOR 2 <UNP Q8VL32_ENTFA>
[ENTEROCOCCUS FAECALIS]
MIINNLKLIREKKKISQSELAALLEVSRQTINGIEKNKYNPSLQLALKIAYYLNCPLEDI
FQWQPE
>2YVAA 196 XRAY 1.85 0.183 0.214 no DnaA initiator-associating protein diaA <UNP
DIAA_ECOLI> [ESCHERICHIA COLI]
MQERIKACFTESIQTQIAAAEALPDAISRAAMTLVQSLLNGNKILCCGNGTSAANAQHFA
ASMINRFETERPSLPAIALNTDNVVLTAIANDRLHDEVYAKQVRALGHAGDVLLAISTRG
NSRDIVKAVEAAVTRDMTIVALTGYDGGELAGLLGPQDVEIRIPSHRSARIQEMHMLTVN
CLCDLIDNTLFPHQDD
>2REBA 352 XRAY 2.30 0.210 NA no REC A <UNP RECA_ECOLI> [ESCHERICHIA COLI]
AIDENKQKALAAALGQIEKQFGKGSIMRLGEDRSMDVETISTGSLSLDIALGAGGLPMGR
IVEIYGPESSGKTTLTLQVIAAAQREGKTCAFIDAEHALDPIYARKLGVDIDNLLCSQPD

TGEQALEICDALARSGAVDVIVVDSVAALTPKAEIEGEIGDSHMGLAARMMSQAMRKLAG
NLKQSNTLLIFINQIRMKIGVMFGNPETTTGGNALKFYASVRLDIRRIGAVKEGENVVGS
ETRVKVVKNKIAAPFKQAEFQILYGEGINFYGELVDLGVKEKLIEKAGAWYSYKGEKIGQ
GKANATAWLKDNPETAKEIEKKVRELLLSNPNSTPDFSVDDSEGVAETNEDF
>4NDHA 182 XRAY 1.85 0.164 0.196 no Aprataxin <UNP APTX_HUMAN> [HOMO SAPIENS]
GSHMGHWSQGLKISMQDPKMQVYKDEQVVVIKDKYPKARYHWLVLPWTSISSLKAVAREH
LELLKHMHTVGEKVIVDFAGSSKLRFRLGYHAIPSMSHVHLHVISQDFDSPCLKNKKHWN
SFNTEYFLESQAVIEMVQEAGRVTVRDGMPELLKLPLRCHECQQLLPSIPQLKEHLRKHW
TQ
>2LFHA 68 NMR NA NA NA no DNA-binding protein inhibitor ID-3 <UNP ID3_HUMAN> [HOMO SAPIENS]
MGHHHHHHSHMGGGKGPAAEEPLSLLDDMNHCYSRLRELVPGVPRGTQLSQVEILQRVID
YILDLQVV
>2A1IA 146 XRAY 1.90 0.218 0.248 no DNA excision repair protein ERCC-1 <UNP ERCC1_HUMAN> [HOMO SAPIENS]
MGSSHHHHHHSQDPAKSNSIIVSPRQRGNPVLKFVRNVPWEFGDVIPDYVLGQSTCALFL
SLRYHNLHPDYIHGRLQSLGKNFALRVLLVQVDVKDPQQALKELAKMCILADCTLILAWS
PEEAGRYLETYKAYEQKPADLLMEKL
>2A1JA 63 XRAY 2.70 0.250 0.275 no DNA repair endonuclease XPF <UNP ERCC4_HUMAN> [HOMO SAPIENS]
MPQDFLLKMPGVNAKNCRSLMHHVKNIAELAALSQDELTSILGNAANAKQLYDFIHTSFA
EVV
>2A1JB 91 XRAY 2.70 0.250 0.275 no DNA excision repair protein ERCC-1 <UNP ERCC1_HUMAN> [HOMO SAPIENS]
MGSSHHHHHHSQDPADLLMEKLEQDFVSRVTECLTTVKSVNKTDSQTLLTTFGSLEQLIA
ASREDLALCPGLGPQKARRLFDVLHEPFLKV
>2A1KA 233 XRAY 2.00 0.243 0.259 no gp32 single stranded DNA binding protein <UNP Q7Y265_BPR69> [ENTEROBACTERIA PHAGE RB69]
KGFSSEDKGEWKLKLDASGNGQAVIRFLPAKTDDALPFAILVNHGFKKNGKWYIETCSST
HGDYDSCPVCQYISKNDLYNTNKTEYSQLKRKTSYWANILVVKDPQAPDNEGKVFKYRFG
KKIWDKINAMIAVDTEMGETPVDVTCPWEGANFVLKVKQVSGFSNYDESKFLNQSAIPNI
DDESFQKELFEQMVDLSEMTSKDKFKSFEELNTKFNQVLGTAALGGAAAAAAS
>4A11B 408 XRAY 3.31 0.178 0.233 no DNA EXCISION REPAIR PROTEIN ERCC-8 <UNP ERCC8_HUMAN> [HOMO SAPIENS]
MLGFLSARQTGLEDPLRLRRAESTRRVLGLELNKDRDVERIHGGGINTLDIEPVEGRYML
SGGSDGVIVLYDLENSSRQSYYTCKAVCSIGRDHPDVHRYSVETVQWYPHDTGMFTSSSF
DKTLKVWDTNTLQTADVFNFEETVYSHHMSPVSTKHCLVAVGTRGPKVQLCDLKSGSCSH
ILQGHRQEILAVSWSPRYDYILATASADSRVKLWDVRRASGCLITLDQHNGKKSQAVESA
NTAHNGKVNGLCFTSDGLHLLTVGTDNRMRLWNSSNGENTLVNYGKVCNNSKKGLKFTVS
CGCSSEFVFVPYGSTIAVYTVYSGEQITMLKGHYKTVDCCVFQSNFQELYSGSRDCNILA
WVPSLYEPVPDDDETTTKSQLNPAFEDAWSSSDEEGGTSAWSHPQFEK
>3VH5T 111 XRAY 2.40 0.194 0.244 no CENP-T <UNP F1NPG5_CHICK> [GALLUS GALLUS]
GSTREPEIASSLIKQIFSHYVKTPVTRDAYKIVEKASERYFKQISSDLEAYSQHAGRKTV
EMADVELLMRRQGLVTDKMPLHVLVERHLPLEYRKLLIPIAVSGNKVIPAK
>3BRGC 427 XRAY 2.20 0.221 0.255 no Recombining binding protein suppressor of hairless <UNP SUH_MOUSE> [MUS MUSCULUS]
GPLGSPPKRLTREAMRNYLKERGDQTVLILHAKVAQKSYGNEKRFFCPPPCVYLMGSGWK
KKKEQMERDGCSEQESQPCAFIGIGNSDQEMQQLNLEGKNYCTAKTLYISDSDKRKHFML
SVKMFYGNSDDIGVFLSKRIKVISKPSKKKQSLKNADLCIASGTKVALFNRLRSQTVSTR
YLHVEGGNFHASSQQWGAFYIHLLDDDESEGEEFTVRDGYIHYGQTVKLVCSVTGMALPR
LIIRKVDKQTALLDADDPVSQLHKCAFYLKDTERMYLCLSQERIIQFQATPCPKEQNKEM

INDGASWTIISTDKAEYTFYEGMGPVLAPVTPVPVVESLQLNGGGDVAMLELTGQNFTPN
LRVWFGDVEAETMYRCGESMLCVVPDISAFREGWRWVRQPVQVPVTLVRNDGVIYSTSLT
FTYTPEP
>1RIFA 282 XRAY 2.00 0.221 0.246 no DNA helicase uvsW <UNP UVSW_BPT4> [ENTEROBACTERIA PHAGE T4]
MDIKVHFHDFSHVRIDCEESTFHELRDFFSFEADGYRFNPRFRYGNWDGRIRLLDYNRLL
PFGLVGQIKKFCDNFGYKAWIDPQINEKEELSRKDFDEWLSKLEIYSGNKRIEPHWYQKD
AVFEGLVNRRRILNLPTSAGRSLIQALLARYYLENYEGKILIIVPTTALTTQMADDFVDY
RLFSHAMIKKIGGGASKDDKYKNDAPVVVGTWQTVVKQPKEWFSQFGMMMNDECHLATGK
SISSIISGLNNCMFKFGLSGSLRDGKANIMQYVGMFGEIFKP
>2QHBA 86 XRAY 2.40 0.251 0.264 no Telomere binding protein TBP1 <UNP Q84ZU4_NICGU> [NICOTIANA GLUTINOSA]
RRIRRPFSVAEVEALVEAVEHLGTGRWRDVKMRAFDNADHRTYVDLKDKWKTLVHTASIA
PQQRRGEPVPQDLLDRVLAAHAYWSQ
>2DGZA 113 NMR NA NA NA no Werner syndrome protein variant <UNP WRN_HUMAN> [HOMO SAPIENS]
GSSGSSGSSQPVISAQEQETQIVLYGKLVEARQKHANKMDVPPAILATNKILVDMAKMRP
TTVENVKRIDGVSEGKAAMLAPLWEVIKHFCQTNSVQTDLFSSTKPQSGPSSG
>1IN4A 334 XRAY 1.60 0.236 0.253 no HOLLIDAY JUNCTION DNA HELICASE RUVB <UNP RUVB_THEMA> [THERMOTOGA MARITIMA]
MSEFLTPERTVYDSGVQFLRPKSLDEFIGQENVKKKLSLALEAAKMRGEVLDHVLLAGPP
GLGKTTLAHIIASELQTNIHVTSGPVLVKQGDMAAILTSLERGDVLFIDEIHRLNKAVEE
LLYSAIEDFQIDIMIGKGPSAKSIRIDIQPFTLVGATTRSGLLSSPLRSRFGIILELDFY
TVKELKEIIKRAASLMDVEIEDAAAEMIAKRSRGTPRIAIRLTKRVRDMLTVVKADRINT
DIVLKTMEVLNIDDEGLDEFDRKILKTIIEIYRGGPVGLNALAASLGVEADTLSEVYEPY
LLQAGFLARTPRGRIVTEKAYKHLKYEVPENRLF
>2INGX 213 XRAY 3.60 0.251 0.302 no Breast cancer type 1 susceptibility protein <UNP BRCA1_HUMAN>
[HOMO SAPIENS]
GPRMSMVVSGLTPEEFMLVYKFARKHHITLTNLITEETTHVVMKTDAEFVCERTLKYFLG
IAGGKWVVSYFWVTQSIKERKMLNEHDFEVRGDVVNGRNHQGPKRARESQDRKIFRGLEI
CCYGPFTNKPTDQLEWMVQLCGASVVKELSSFTLGTGVHPIVVVQPDAWTEDNGFHAIGQ
MCEAPVVTREWVLDSVALYQCQELDTYLIPQIP
>2OBPA 96 XRAY 1.70 0.188 0.219 no Putative DNA-binding protein <UNP Q46TT3_RALEJ> [RALSTONIA EUTROPHA]
GMSDPGNEQNGDGIDPAIVEVLLVLREAGIENGATPWSLPKIAKRAQLPMSVLRRVLTQL
QAAGLADVSVEADGRGHASLTQEGAALAAQLFPDPF
>3TUOA 105 XRAY 1.70 0.161 0.218 no DNA-binding protein SATB1 <UNP SATB1_HUMAN> [HOMO SAPIENS]
GPGSGTMLPVFCVVEHYENAIEYDCKEEHAEFVLVRKDMLFNQLIEMALLSLGYSHSSAA
QAKGLIQVGKWNPVPLSYVTDAPDATVADMLQDVYHVVTLKIQLH
>3V20A 293 XRAY 2.35 0.246 0.293 no Endonuclease Bse634IR <UNP Q8RT53_GEOSE> [GEOBACILLUS STEAROTHERMOPHILUS]
MTTNLTNSNCVEEYKENGKTKIRIKPFNALIELYHHQTPTGSIKENLDKLENYVKDVVKA
KGLAIPTSGAFSNTRGTWFEVMIAIQSWNYRVKRELNDYLIIKMPNVKTFDFRKIFDNET
REKLHQLEKSLLTHKQQVRLITSNPDLLIIRQKDLIKSEYNLPINKLTHENIDVALTLFK
DIEGKCKWDSLVAGVGLKTSLRPDRRLQLVHEGNILKSLFAHLKMAYWNPKAEFKYYGAS
SEPVSKADDDALQTAATHTIVNVNSTPERAVDDIFSLTSFEDIDKMLDQIIKK
>4E1PA 61 XRAY 1.73 0.161 0.185 no Protein lsr2 <UNP LSR2_MYCTU> [MYCOBACTERIUM TUBERCULOSIS]
MAKKVTVTLVDDFDGSGAADETVEFGLDGVTYEIDLSTKNATKLRGDLKQWVAAGRRVGG
R

>1WH5A 80 NMR NA NA NA no ZF-HD homeobox family protein <UNP Y5541_ARATH> [ARABIDOPSIS THALIANA]
GSSGSSGSSAEAGGGIRKRHRTKFTAEQKERMLALAERIGWRIQRQDDEVIQRFCQETGV
PRQVLKVWLHNNKHSGPSSG
>3II2A 157 XRAY 2.00 0.173 0.237 no Putative uncharacterized protein <UNP Q70LE6_AFV1> [ACIDIANUSFILAMENTOUS VIRUS 1]
GSEMSVVEYEVVSKNLTSKMSHELLFSVKKRWFVKPFRHDRQLGKLHYKLLPGNYIKFGL
YVLKNQDYARFEIAWVHVDKDGKIEERTVYSIETYWHIFIDIENDLNCPYVLAKFIEMRP
EFHKTAWVEESNYSIAEDDIQMVESIKRYLERKIASD
>3II6A 203 XRAY 2.40 0.240 0.280 no DNA repair protein XRCC4 <UNP XRCC4_HUMAN> [HOMO SAPIENS]
MERKISRIHLVSEPSITHFLQVSWEKTLESGFVITLTDGHSAWTGTVSESEISQEADDME
MEKGKYVGELRKALLSGAGPADVYTFNFSKESCYFFFEKNLKDVSFRLGSFNLEKVENPA
EVIRELICYCLDTTAENQAKNEHLQKENERLLRDWNDVQGRFEKCVSAKEALETDLYKRF
ILVLNEKKTKIRSLHNKLLNAAQ
>4KOPA 177 XRAY 1.75 0.185 0.220 no Single-stranded DNA-binding protein WHY2, mitochondrial <UNP
WHY2_ARATH> [ARABIDOPSIS THALIANA]
MKDAAKPSGRLFAPYSIFKGKAALSVEPVLPSFTEIDSGNLRIDRRGSLMMTFMPAIGER
KYDWEKKQKFALSPTEVGSLISMGSKDSSEFFHDPSMKSSNAGQVRKSLSVKPHADGSGY
FISLSVNNSILKTNDYFVVPVTKAEFAVMKTAFSFALPHIMGWNRLTGHLEHHHHHH
>3KOJA 108 XRAY 1.90 0.202 0.218 no uncharacterized protein ycf41 <UNP Q5N255_SYNP6> [SYNECHOCOCCUS ELONGATUS PCC 6301]
MGHHHHHHSHMNSCILQATVVEAPQLRYAQDNQTPVAEMVVQFPGLSSKDAPARLKVVGW
GAVAQELQDRCRLNDEVVLEGRLRINSLLKPDGNREKQTELTVTRVHH
>2PKHA 148 XRAY 1.95 0.222 0.277 no Histidine utilization repressor <UNP Q87UX0_PSESM> [PSEUDOMONAS SYRINGAE PV. TOMATO]
SNAARGHRHTCKVMVLKEEAAGSERALALDMREGQRVFHSLIVHFENDIPVQIEDRFVNA
QVAPDYLKQDFTLQTPYAYLSQVAPLTEGEHVVEAILAEADECKLLQIDAGEPCLLIRRR
TWSGRQPVTAARLIHPGSRHRLEGRFTK
>2RH3A 121 XRAY 1.70 0.184 0.209 no Protein virC2 <UNP VIRC2_AGRT5> [AGROBACTERIUM TUMEFACIENS]
IQVFLSARPPAPEVSKIYDNLILQYSPSKSLQMILRRALGDFENMLADGSFRAAPKSYPI
PHTAFEKSIIVQTSRMFPVSLIEAARNHFDPLGLETARAFGHKLATAALACFFAREKATN
S
>1RH6A 55 XRAY 1.70 0.193 0.226 no Excisionase <UNP VXIS_LAMBD> [ENTEROBACTERIA PHAGE LAMBDA]
MYLTLQEWNARQRRPRSLETVRRWVRESRIFPPPVKDGREYLFHESAVKVDLNRP
>1F6VA 91 NMR NA NA NA no DNA TRANSPOSITION PROTEIN <UNP VPB_BPMU> [ENTEROBACTERIA PHAGE MU]
GSRIAKRTAINKTKKADVKAIADAWQINGEKELELLQQIAQKPGALRILNHSLRLAAMTA
HGKGERVNEDYLRQAFRELDLDVDISTLLRN
>3F6CA 134 XRAY 1.45 0.212 0.256 no Positive transcription regulator evgA <UNP
EVGA_ECOLI> [ESCHERICHIA COLI K-12]
SLNAIIIDDHPLAIAAIRNLLIKNDIEILAELTEGGSAVQRVETLKPDIVIIDVDIPGVN
GIQVLETLRKRQYSGIIIIVSAKNDHFYGKHCADAGANGFVSKKEGMNNIIAAIEAAKNG
YCYFPFSLNRFVGS
>4F6MA 133 XRAY 2.40 0.223 0.240 no Transcriptional regulator Kaiso <UNP KAISO_HUMAN> [HOMO SAPIENS]
ANKRMKVKHDDHYELIVDGRVYYICIVCKRSYVCLTSLRRHFNIHSWEKKYPCRYCEKVF
PLAEYRTKHEIHHTGERRYQCLACGKSFINYQFMSSHIKSVHSQDPSGDSKLYRLHPCRS

LQIRQYAYLSDRS
>4NE3A 93 XRAY 1.80 0.232 0.252 no Centromere protein S <UNP CENPS_HUMAN> [HOMO SAPIENS]
SYQQRLKAAVHYTVGCLCEEVALDKAMQFSKQTIAAISELTFRQCENFAKDLEMFARHAK
RTTINTEDVKLLARRSNSLLKYITDKSEEIAQA
>1GDTA 183 XRAY 3.00 0.235 NA no GAMMA DELTA RESOLVASE <UNP TNR1_ECOLI> [ESCHERICHIA COLI]
MRLFGYARVSTSQQSLDIQVRALKDAGVKANRIFTDKASGSSSDRKGLDLLRMKVEEGDV
ILVKKLDRLGRDTADMIQLIKEFDAQGVSIRFIDDGISTDGEMGKMVVTILSAVAQAERQ
RILERTNEGRQEAMAKGVVFGRKRKIDRDAVLNMWQQGLGASHISKTMNIARSTVYKVIN
ESN
>2AN7A 83 NMR NA NA NA no Protein parD <UNP PARD4_ECOLI> [ESCHERICHIA COLI]
MSRLTIDMTDQQHQSLKALAALQGKTIKQYALERLFPGDADADQAWQELKTMLGNRINDG
LAGKVSTKSVGEILDEELSGDRA
>1ZZKA 82 XRAY 0.95 0.124 0.127 no Heterogeneous nuclear ribonucleoprotein K <UNP HNRPK_HUMAN> [HOMO SAPIENS]
GAMGPIITTQVTIPKDLAGSIIGKGGQRIKQIRHESGASIKIDEPLEGSEDRIITITGTQ
DQIQNAQYLLQNSVKQYSGKFF
>2RRDA 101 NMR NA NA NA no HRDC domain from Bloom syndrome protein <UNP BLM_HUMAN> [HOMO SAPIENS]
GIPEFKQKALVAKVSQREEMVKKCLGELTEVCKSLGKVFGVHYFNIFNTVTLKKLAESLS
SDPEVLLQIDGVTEDKLEKYGAEVISVLQKYSEWTSPAEDS
>2L49A 99 NMR NA NA NA no C protein <UNP Q83VS7_BPP2> [ENTEROBACTERIA PHAGE P2]
MSNTISEKIVLMRKSEYLSRQQLADLTGVPYGTLSYYESGRSTPPTDVMMNILQTPQFTK
YTLWFMTNQIAPESGQIAPALAHFGQNETTSPHSGQKTG
>4E45E 137 XRAY 2.00 0.202 0.245 no Fanconi anemia group M protein <UNP FANCM_HUMAN> [HOMO SAPIENS]
GAMDPMRQSSLKKDWFLSEEEFKLWNRLYRLRDSDEIKEITLPQVQFSSLQNEENKPAQE
STTGIHQLSLSEWRLWQDHPLPTHQVDHSDRCRHFIGLMQMIEGMRHEEGECSYELEVES
YLQMEDVTSTFIAPRNE
>3GYDA 187 XRAY 1.79 0.170 0.212 no Cyclic nucleotide-binding domain <UNP Q1GZZ4_METFK> [METHYLOBACILLUS FLAGELLATUS KT]
MGSDKIHHHHHHENLYFQGMYPDLVHLGGADKYFEEILEIVNKIKLFGDFSNEEVRYLCS
YMQCYAAPRDCQLLTEGDPGDYLLLILTGEVNVIKDIPNKGIQTIAKVGAGAIIGEMSMI
DGMPRSASCVASLPTDFAVLSRDALYQLLANMPKLGNKVLIRLLQLLTARFRESYDRILP
KTLGELI
>2CXYA 125 XRAY 1.60 0.186 0.203 no BAF250b subunit <UNP ARI1B_HUMAN> [HOMO SAPIENS]
GSSGSSGEKITKVYELGNEPERKLWVDRYLTFMEERGSPVSSLPAVGKKPLDLFRLYVCV
KEIGGLAQVNKNKKWRELATNLNVGTSSSAASSLKKQYIQYLFAFECKIERGEEPPPEVF
STGDT
>1KN0A 212 XRAY 2.85 0.286 0.297 no Rad52 <UNP RAD52_HUMAN> [HOMO SAPIENS]
MSGTEEAILGGRDSHPAAGGGSVLCFGQCQYTAEEYQAIQKALRQRLGPEYISSRMAGGG
QKVCYIEGHRVINLANEMFGYNGWAHSITQQNVDFVDLNNGKFYVGVCAFVRVQLKDGSY
HEDVGYGVSEGLKSKALSLEKARKEAVTDGLKRALRSFGNALGNCILDKDYLRSLNKLPR
QLPLEVDLTKAKRQDLEPSVEEARYNSCRPNM
>2KNGA 55 NMR NA NA NA no Protein lsr2 <UNP LSR2_MYCTU> [MYCOBACTERIUM TUBERCULOSIS]
SGSGRGRGAIDREQSAAIREWARRNGHNVSTRGRIPADVIDAYHAATLEHHHHHH
>2QSFA 533 XRAY 2.35 0.226 0.245 no DNA repair protein RAD4 <UNP RAD4_YEAST> [SACCHAROMYCES CEREVISIAE]
MGNEVAGVEDISVEIKPSSKRNSDARRTSRNVCSNEERKRRKYFHMLYLVCLMVHGFIRN
EWINSKRLSRKLSNLVPEKVFELLHPQKDEELPLRSTRKLLDGLKKCMELWQKHWKITKK

YDNEGLYMRTWKEIEMSANNKRKFKTLKRSDFLRAVSKGHGDPDISVQGFVAMLRACNVN
ARLIMSCQPPDFTNMKIDTSLNGNNAYKDMVKYPIFWCEVWDKFSKKWITVDPVNLKTIE
QVRLHSKLAPKGVACCERNMLRYVIAYDRKYGCRDVTRRYAQWMNSKVRKRRITKDDFGE
KWFRKVITALHHRKRTKIDDYEDQYFFQRDESEGIPDSVQDLKNHPYYVLEQDIKQTQIV
KPGCKECGYLKVHGKVGKVLKVYAKRDIADLKSARQWYMNGRILKTGSRCKKVIKRTVGR
PKGEAEEEDERLYSFEDTELYIPPLASASGEITKNTFGNIEVFAPTMIPGNCCLVENPVA
IKAARFLGVEFAPAVTSFKFERGSTVKPVLSGIVVAKWLREAIETAIDGIEFI
>2QSFX 171 XRAY 2.35 0.226 0.245 no UV excision repair protein RAD23 <UNP RAD23_YEAST> [SACCHAROMYCES CEREVISIAE]
GSGNASSGALGTTGGATDAAQGGPPGSIGLTVEDLLSLRQVVSGNPEALAPLLENISARY
PQLREHIMANPEVFVSMLLEAVGDNMQDVMEGADDMVEGEDIEVTGEAAAAGLGQGEGEG
SFQVDYTPEDDQAISRLCELGFERDLVIQVYFACDKNEEAAANILFSDHAD
>1WJVA 79 NMR NA NA NA no Cell growth regulating nucleolar protein LYAR <UNP LYAR_MOUSE> [MUS MUSCULUS]
GSSGSSGMVFFTCNACGESVKKIQVEKHVSNCRNCECLSCIDCGKDFWGDDYKSHVKCIS
EGQKYGGKGYEAKSGPSSG
>1WJ2A 78 NMR NA NA NA no Probable WRKY transcription factor 4 <UNP WRKY4_ARATH> [ARABIDOPSIS THALIANA]
GSSGSSGVQTTSEVDLLDDGYRWRKYGQKVVKGNPYPRSYYKCTTPGCGVRKHVERAATD
PKAVVTTYEGKHNHDLPA
>3OLCX 298 XRAY 2.40 0.223 0.258 no DNA topoisomerase 2-binding protein 1 <UNP TOPB1_HUMAN> [HOMO SAPIENS]
MSRNDKEPFFVKFLKSSDNSKCFFKALESIKEFQSEEYLQIITEEEALKIKENDRSLYIC
DPFSGVVFDHLKKLGCRIVGPQVVIFCMHHQRCVPRAEHPVYNMVMSDVTISCTSLEKEK
REEVHKYVQMMGGRVYRDLNVSVTHLIAGEVGSKKYLVAANLKKPILLPSWIKTLWEKSQ
EKKITRYTDINMEDFKCPIFLGCIICVTGLCGLDRKEVQQLTVKHGGQYMGQLKMNECTH
LIVQEPKGQKYECAKRWNVHCVTTQWFFDSIEKGFCQDESIYKTEPRPEALEHHHHHH
>2KI2A 90 NMR NA NA NA no Ss-DNA binding protein 12RNP2 <UNP O25501_HELPY> [HELICOBACTER PYLORI]
MRNIYVGNLVYSATSEQVKELFSQFGKVFNVKLIYDRETKKPKGFGFVEMQEESVSEAIA
KLDNTDFMGRTIRVTEANPKKSLEHHHHHH
>2KIWA 111 NMR NA NA NA no Int protein <UNP Q4L7R3_STAHJ> [STAPHYLOCOCCUS HAEMOLYTICUS JCSC1435]
TFKQVADDWLKQYANDVKVSSVRAREKAIQHAIERFNTKPIQTIKKHDYQRFVDDISAQY
SKNYVDSIVASTNMIFKYAYDTRLIKAMPSEGIKRPKKKVSVELEHHHHHH
>3W3WB 69 XRAY 2.20 0.217 0.256 no Protein STE12 <UNP STE12_YEAST> [SACCHAROMYCES CEREVISIAE]
PRRRTVGMKSSQGNVPTGNKQSVGKSAKISKPLHIKTSAYQKQYKINLETKARPSAGDED
SAHPDKNKE
>3GVAA 116 XRAY 2.00 0.221 0.265 no Alkyltransferase-like protein 1 <UNP ATL1_SCHPO> [SCHIZOSACCHAROMYCES POMBE]
MRMDEFYTKVYDAVCEIPYGKVSTYGEIARYVGMPSYARQVGQAMKHLHPETHVPWHRVI
NSRGTISKRDISAGEQRQKDRLEEEGVEIYQTSLGEYKLNLPEYMWKPGSHHHHHH
>3GV6A 58 XRAY 1.76 0.214 0.266 no Chromobox protein homolog 6 <UNP CBX6_HUMAN> [HOMO SAPIENS]
ERVFAAESIIKRRIRKGRIEYLVKWKGWAIKYSTWEPEENILDSRLIAAFEQKERERE
>3OMYA 52 XRAY 1.30 0.151 0.173 no Protein traM <UNP TRAM8_ECOLX> [ESCHERICHIA COLI]
MPKIQTYVNNNVYEQITDLVTIRKQEGIEEASLSNVSSMLLELGLRVYMIQQ
>4H9NC 212 XRAY 1.95 0.209 0.209 no Death domain-associated protein 6 <UNP DAXX_HUMAN> [HOMO SAPIENS]
SPRTRGSRRQIQRLEQLLALYVAEIRRLQEKELDLSELDDPDSAYLQEARLKRKLIRLFG

RLCELKDCSSLTGRVIEQRIPYRGTRYPEVNRRIERLINKPGPDTFPDYGDVLRAVEKAA
ARHSLGLPRQQLQLMAQDAFRDVGIRLQERRHLDLIYNFGCHLTDDYRPGVDPALSDPVL
ARRLRENRSLAMSRLDEVISKYAMLQDKSEEG
>3FHWA 115 XRAY 1.90 0.276 0.289 no Primosomal replication protein n <UNP PRIB_BORPA> [BORDETELLA PARAPERTUSSIS]
MNTLELSARVLECGAMRHTPAGLPALELLLVHESEVVEAGHPRRVELTISAVALGDLALL
LADTPLGTEMQVQGFLAPARKDSVKVKLHLQQARRIAGSMGRDPLVGLEHHHHHH
>3RCOA 89 XRAY 1.80 0.178 0.230 no Tudor domain-containing protein 7 <UNP TDRD7_HUMAN> [HOMO SAPIENS]
ENLYFQGMLEGDLVSKMLRAVLQSHKNGVALPRLQGEYRSLTGDWIPFKQLGFPTLEAYL
RSVPAVVRIETSRSGEITCYAMACTETAR
>1ZP7A 206 XRAY 2.25 0.219 0.269 no Recombination protein U <UNP RECU_BACSU> [BACILLUS SUBTILIS]
MIRYPNGKTFQPKHSVSSQNSQKRAPSYSNRGMTLEDDLNETNKYYLTNQIAVIHKKPTP
VQIVNVHYPKRSAAVIKEAYFKQSSTTDYNGIYKGRYIDFEAKETKNKTSFPLQNFHDHQ
IEHMKQVKAQDGICFVIISAFDQVYFLEADKLFYFWDRKEKNGRKSIRKDELEETAYPIS
LGYAPRIDYISIIEQLYFSPSSGAKG
>2F2EA 146 XRAY 1.85 0.181 0.251 no PA1607 <UNP Q9I3B4_PSEAE> [PSEUDOMONAS AERUGINOSA]
MVKRTSHKQASCPVARPLDVIGDGWSMLIVRDAFEGLTRFGEFQKSLGLAKNILAARLRN
LVEHGVMVAVPAESGSHQEYRLTDKGRALFPLLVAIRQWGEDYFFAPDESHVRLVERDSG QPVPRLQVRAGDGSPLAAEDTRVSRD
>1F2RI 100 NMR NA NA NA no INHIBITOR OF CASPASE-ACTIVATED DNASE <UNP DFFA_MOUSE> [MUS MUSCULUS]
MELSRGASAPDPDDVRPLKPCLLRRNHSRDQHGVAASSLEELRSKACELLAIDKSLTPIT
LVLAEDGTIVDDDDYFLCLPSNTKFVALACNEKWTYNDSD
>4JLXA 366 XRAY 2.00 0.189 0.215 no Uncharacterized protein <UNP I3LM39_PIG> [SUS SCROFA]
GAMGAWKLQTVLEKVRLSRHEISEAAEVVNWVVEHLLRRLQGGESEFKGVALLRTGSYYE
RVKISAPNEFDVMFKLEVPRIQLEEYCNSGAHYFVKFKRNPGGNPLEQFLEKEILSASKM
LSKFRKIIKEEIKNIEDTGVTVERKRRGSPAVTLLISKPKEISVDIILALESKSSWPAST
QKGLPISQWLGAKVKNNLKRQPFYLVPKHAKEGSGFQEETWRLSFSHIEKDILKNHGQSK
TCCEIDGVKCCRKECLKLMKYLLEQLKKKFGNRRELAKFCSYHVKTAFFHVCTQDPHDNQ
WHLKNLECCFDNCVAYFLQCLKTEQLANYFIPGVNLFSRDLIDKPSKEFLSKQIEYERNN
GFPVFW
>2JD3A 130 XRAY 2.80 0.239 0.277 no STBB PROTEIN <UNP Q9S101_ECOLI> [ESCHERICHIA COLI]
MDDERKRKKYTLYLHPEKAADFQTLEAIESVPRSERGELFRNAFISGMALHQLDPRLPVL
LTAILSEEFSADQVVTLLSQTTGWKPSQADIRAVLTELGASQSVEKMPPSATDSVQEAMN
DVRLKMKKLF
>3KZ5A 52 XRAY 1.58 0.198 0.234 no Protein sopB <UNP SOPB_ECOLI> [ESCHERICHIA COLI]
GSHMSSRHQFAPGATVLYKGDKMVLNLDRSRVPTECIEKIEAILKELEKPAP
>4JW3A 120 XRAY 2.60 0.226 0.264 no Neocarzinostatin <UNP NCZS_STRML> [STREPTOMYCES MALAYENSIS]
AAPTATVTPSSGLSDGTVVKVAGAGLQAGTAYWVAQWARVDTGVWAYNPADNSSVTADAN
GSASTSLTVRRSFEGFLFDGTRWGTVDCTTAACQVGLSDAAGNGPEGVAISFAAHHHHHH
>1DMLA 319 XRAY 2.70 0.230 0.281 no DNA POLYMERASE PROCESSIVITY FACTOR <UNP VPAP_HHV11> [HUMAN HERPESVIRUS 1]
MTDSPGGVAPASPVEDASDASLGQPEEGAPCQVVLQGAELNGILQAFAPLRTSLLDSLLV
MGDRGILIHNTIFGEQVFLPLEHSQFSRYRWRGPTAAFLSLVDQKRSLLSVFRANQYPDL
RRVELAITGQAPFRTLVQRIWTTTSDGEAVELASETLMKRELTSFVVLVPQGTPDVQLRL
TRPQLTKVLNATGADSATPTTFELGVNGKFSVFTTSTCVTFAAREEGVSSSTSTQVQILS
NALTKAGQAAANAKTVYGENTHRTFSVVVDDCSMRAVLRRLQVGGGTLKFFLTTPVPSLC

VTATGPNAVSAVFLLKPQK
>3BTPA 556 XRAY 2.30 0.212 0.256 no Single-strand DNA-binding protein <UNP VIRE2_AGRT5> [AGROBACTERIUM TUMEFACIENS STR.]
MDPKAEGNGENITETAAGNVETSDFVNLKRQKREGVNSTGMSEIDMTGSQETPEHNMHGS
PTHTDDLGPRLDADMLDSQSSHVSSSAQGNRSEVENELSNLFAKMALPGHDRRTDEYILV
RQTGQDKFAGTTKCNLDHLPTKAEFNASCRLYRDGVGNYYPPPLAFERIDLPEQLAAQLH
NLEPREQSKQCFQYKLEVWNRAHAEMGITGTDIFYQTDKNIKLDRNYKLRPEDRYIQTEK
YGRREIQKRYEHQFQAGSLLPDILIKTPQNDIHFSYRFAGDAYANKRFEEFERAIKTKYG
SDTEIKLKSKSGIMHDSKYLESWERGSADIRFAEFAGENRAHNKQFPAATVNMGRQPDGQ
GGMTRDRHVSVDYLLQNLPNSPWTQALKEGKLWDRVQVLARDGNRYMSPSRLEYSDPEHF
TQLMDQVGLPVSMGRQSHANSVKFEQFDRQAAVIVADGPNLREVPDLSPEKLQQLSQKDV
LIADRNEKGQRTGTYTNVVEYERLMMKLPSDAAQLLAEPSDRYSRAFVRPEPALPPISDS
RRTYESRPRGPTVNSL
>3BTPB 63 XRAY 2.30 0.212 0.256 no Protein virE1 <UNP VIRE1_AGRT5> [AGROBACTERIUM TUMEFACIENS STR.]
MVIIKLNANKNMPVLAVEKPQEIHKEELSDHHQSNGFTSLDLEMIELENFVLHCPLPEEN
LAG
>1YUAA 122 NMR NA NA NA no TOPOISOMERASE I <UNP TOP1_ECOLI> [ESCHERICHIA COLI]
MNGEVAPPKEDPVPLPELPCEKSDAYFVLRDGAAGVFLAANTFPKSRETRAPLVEELYRF
RDRLPEKLRYLADAPQQDPEGNKTMVRFSRKTKQQYVSSEKDGKATGWSAFYVDGKWVEG
KK
>1YUIA 54 NMR NA NA NA no GAGA-FACTOR <UNP GAGA_DROME> [DROSOPHILA MELANOGASTER]
PKAKRAKHPPGTEKPRSRSQSEQPATCPICYAVIRQSRNLRRHLELRHFAKPGV
>3V6TA 499 XRAY 1.85 0.206 0.222 no dHax3 <PDB 3V6T> [XANTHOMONAS]
LEHHHHHHQWSGARALEALLTVAGELRGPPLQLDTGQLLKIAKRGGVTAVEAVHAWRNAL
TGAPLNLTPEQVVAIASHDGGKQALETVQRLLPVLCQAHGLTPQQVVAIASHDGGKQALE
TVQRLLPVLCQAHGLTPEQVVAIASHDGGKQALETVQALLPVLCQAHGLTPEQVVAIASN
GGGKQALETVQRLLPVLCQAHGLTPQQVVAIASNGGGKQALETVQRLLPVLCQAHGLTPQ
QVVAIASNGGGKQALETVQRLLPVLCQAHGLTPQQVVAIASNSGGKQALETVQRLLPVLC
QAHGLTPQQVVAIASNGGGKQALETVQRLLPVLCQAHGLTPQQVVAIASHDGGKQALETV
QRLLPVLCQAHGLTPEQVVAIASNGGGKQALETVQRLLPVLCQAHGLTPEQVVAIASHDG
GKQALETVQRLLPVLCQAHGLTPQQVVAIASNGGGRPALESIVAQLSRPDPALAALTNDH
LVALACLGGRPALDAVKKL
>1V63A 101 NMR NA NA NA no Nucleolar transcription factor 1 <UNP UBF1_MOUSE> [MUS MUSCULUS]
GSSGSSGPKKPPMNGYQKFSQELLSNGELNHLPLKERMVEIGSRWQRISQSQKEHYKKLA
EEQQRQYKVHLDLWVKSLSPQDRAAYKEYISNKRKSGPSSG
>3V68A 252 XRAY 1.56 0.199 0.225 no Putative uncharacterized protein <UNP Q8TZE5_PYRFU> [PYROCOCCUS FURIOSUS]
MIERILEFTAKHEEWIVGENVEDFTNENIAMFLSRVSNTVSSKIPGYLGEKIDVNGLLSI
KIEGSLEEKLKALISPKVSRQIGRLVMEDDKKLKKLLVEVAKAVLTREILKNELPIEFPG
GKIEGLKIQPRYEEDHINFTARYGSWIVVKRMIIDEKTPLLDIARLLASINETAVNKIKD
FADVDDKKIVEYFGGFKKVKKEEEIKEIVQLFREFKGNEFEVRYAAREMLSKLGLKVDVP
SKNLEKYLEKAG
>3V62C 69 XRAY 2.90 0.187 0.237 no ATP-dependent DNA helicase SRS2 <UNP SRS2_YEAST> [SACCHAROMYCES CEREVISIAE]
SHNPDDTTVDNRPIISNAKFLADAAMKKTQKFSKKVKNEPASSQMDIFSQLSRAKKKSKL
NNGEIIVID
>2FUFA 134 XRAY 1.45 0.170 0.189 no Large T antigen <UNP TALA_SV40> [SIMIAN VIRUS

40] GSKVEDPKDFPSELLSFLSHAVFSNRTLACFAIYTTKEKAALLYKKIMEKYSVTFISRHN
SYNHNILFFLTPHRHRVSAINNYAQKLCTFSFLICKGVNKEYLMYSALTRDPFSVIEESL
PGGLKEHDFNPESS
>4GFKA 196 XRAY 1.95 0.212 0.239 no Nucleoid occlusion factor SlmA <UNP SLMA_VIBCH> [VIBRIO CHOLERAE O1 BIOVAR EL TOR]
MAGNKKINRREEILQALAEMLESNEGASRITTAKLAKQVGVSEAALYRHFPSKTRMFEGL
IEFIEESLMSRINRIFDEEKDTLNRIRLVMQLLLAFAERNPGLTRILSGHALMFENERLR
DRINQLFERIETSLRQILRERKLREGKSFPVDENILAAQLLGQVEGSLNRFVRSDFKYLP
TANFDEYWALLSAQIK
>2GFUA 134 NMR NA NA NA no DNA mismatch repair protein MSH6 <UNP MSH6_HUMAN> [HOMO SAPIENS]
KAKNLNGGLRRSVAPAAPTSSDFSPGDLVWAKMEGYPWWPSLVYNHPFDGTFIREKGKSV
RVHVQFFDDSPTRGWVSKRLLKPYTGSKSKEAQKGGHFYSAKPEILRAMQRADEALNKDK
IKRLELAVSDEPSE
>2YRQA 173 NMR NA NA NA no High mobility group protein B1 <UNP HMGB1_HUMAN> [HOMO SAPIENS]
GSSGSSGMGKGDPKKPRGKMSSYAFFVQTCREEHKKKHPDASVNFSEFSKKCSERWKTMS
AKEKGKFEDMAKADKARYEREMKTYIPPKGETKKKFKDPNAPKRPPSAFFLFCSEYRPKI
KGEHPGLSIGDVAKKLGEMWNNTAADDKQPYEKKAAKLKEKYEKDIAAYRAKG
>4EOGA 480 XRAY 2.30 0.227 0.250 no Putative uncharacterized protein <PDB 4EOG> [PYROCOCCUS FURIOSUS]
MGMRVLVTTWGNPFQWEPITYEYRGIKVKSRNTLPILVKTLEPERILILVADTMANYYDS
GKNKPEIEEKSFSSYSEVVEDTKERILWHIKEEVIEELREEDPELAKKIENMLKDERITI
EVLPGVGVFGNITVEGEMLDFYYYATYKLAEWLPVQNNLEVYLDLTHGINFMPTFTYRAL
RNLLGLLAYLYNVKFEIVNSEPYPLGVSQEIREDTILHIREIGEGVVRPRPQYSPVEGKL
YWNAFISSVANGFPLVFASFYPNIRDVEDYLNKKLEEFLVGIEVGEREDGKPYVKREKAL
DRSFKNASKLYYALRVFNTKFQNYPKKEVPIEEIMEISKIFESLPRIGIILERQVEWLRN
LVYGRLWYENGEQKIKKGLLEIIKDKKDKRKEAEALKKGKTISLAEAAKLTRIFSPSGER
IETIESPNVVRNFIAHSGFEYNIVYVKYDRLSDRLYFFYKDKEKAANLAYEALLYRGEKE
>3P7NA 258 XRAY 2.10 0.266 0.329 no Sensor histidine kinase <UNP Q2NB98_ERYLH> [ERYTHROBACTER LITORALIS]
MRGSHHHHHHGMASMTGGQQMGRDLYDDDDKDHPFTMGQDRPIDGSGAPGADDTRVEVQP
PAQWVLDLIEASPIASVVSDPRLADNPLIAINQAFTDLTGYSEEECVGRNCRFLAGSGTE
PWLTDKIRQGVREHKPVLVEILNYKKDGTPFRNAVLVAPIYDDDDELLYFLGSQVEVDDD
QPNMGMARRERAAEMLKTLSPRQLEVTTLVASGLRNKEVAARLGLSEKTVKMHRGLVMEK
LNLKTSADLVRIAVEAGI
>4K2JA 140 XRAY 2.05 0.184 0.226 no KSHV (HHV-8) latency-associated nuclear antigen (LANA) <UNP Q9DUN0_HHV8> [HUMAN HERPESVIRUS 8]
SHPRYQQPPVPYRQIDDCPAKARPQHIFYRRFLGKDGRRDPKCQWKFAVIFWGNDPYGLK
KLSQAFQFGGVKAGPVSCLPHPGPDQSPITYCVYVYCQNKDTSKKVQMARLAWEASHPLA
GNLQSSIVKFKKPLPLTQPG
>3K2AA 67 XRAY 1.95 0.216 0.239 no Homeobox protein Meis2 <UNP MEIS2_HUMAN> [HOMO SAPIENS]
GSGIFPKVATNIMRAWLFQHLTHPYPSEEQKKQLAQDTGLTILQVNNWFINARRRIVQPM
IDQSNRA
>4DAMA 128 XRAY 1.70 0.155 0.192 no Single-stranded DNA-binding protein 1 <UNP SSB1_STRCO> [STREPTOMYCES COELICOLOR] MRGSHHHHHHGSMNEIMICAVGNVATTPVFRDLANGPSVRFRLAVTARYWDREKNAWTDG
HTNFFTVWANRQLATNASGSLAVGDPVVVQGRLKVRTDVREGQSRTSADIDAVAIGHDLA
RGTAAFRR
>4I99A 354 XRAY 2.30 0.221 0.236 no Chromosome partition protein Smc <UNP SMC_PYRFU>

[PYROCOCCUS FURIOSUS]
MPYIEKLELKGFKSYGNKKVVIPFSKGFTAIVGANGSGKSNIGDAILFVLGGLSAKAMRA
SRISDLIFAGSKNEPPAKYAEVAIYFNNEDRGFPIDEDEVVIRRRVYPDGRSSYWLNGRR
ATRSEILDILTAAMISPDGYNIVLQGDITKFIKMSPLERRLLIDDISGIAEYDSKKEKAL
EEEKEKKNVFMRTFEAISRNFSEIFAKLSPGGSARLILENPEDPFSGGLEIEAKPAGKDV
KRIEAMSGGEKALTALAFVFAIQKFKPAPFYLFDEIDAHLDDANVKRVADLIKESSKESQ
FIVITLRDVMMANADKIIGVSMRDGVSKVVSLSLEKAMKILEEIRKKQGWEHGN
>4I99C 87 XRAY 2.30 0.221 0.236 no Putative uncharacterized protein <UNP Q8TZY3_PYRFU> [PYROCOCCUS FURIOSUS]
KKVEIDEEIFVIDDFRVDIEKYVEELYKVVKKIYEKTGTPIKFWDLVPDVEPKIIARTFL
YLLFLENMGRVEIIQEEPFGEILVVPM
>4GNXA 114 XRAY 2.80 0.222 0.278 no Putative uncharacterized protein <UNP Q4P6U8_USTMA> [USTILAGO MAYDIS]
MEKPTPLINSSMLGQYVGQTVRIVGKVHKVTGNTLLMQTSDLGNVEIAMTPDSDVSSSTF
VEVTGKVSDAGSSFQANQIREFTTVDCGHDVDLTLVENVVQISAAFPNLFSDST
>4GNXB 136 XRAY 2.80 0.222 0.278 no Putative uncharacterized protein <UNP Q4PBD4_USTMA> [USTILAGO MAYDIS]
GKKAGNNTLRPVTIRQILNAEQPHPDAEFILDGAELGQLTFVAVVRNISRNATNVAYSVE
DGTGQIEVRQWLDSSSDDSSKASEIRNNVYVRVLGTLKSFQNRRSISSGHMRPVIDYNEV
MFHRLEAVHAHLQVTR
>4GNXC 444 XRAY 2.80 0.222 0.278 no Putative uncharacterized protein <UNP Q4P407_USTMA> [USTILAGO MAYDIS]
MPIYPIEGLSPYQNRWTIKARVTSKSDIRHWSNQRGEGKLFSVNLLDDSGEIKATGFNDA
VDRFYPLLQENHVYLISKARVNIAKKQFSNLQNEYEITFENSTEIEECTDATDVPEVKYE
FVRINELESVEANQQCDVIGILDSYGELSEIVSKASQRPVQKRELTLVDQGNRSVKLTLW
GKTAETFPTNAGVDEKPVLAFKGVKVGDFGGRSLSMFSSSTMLINPDITESHVLRGWYDN
DGAHAQFQPYTNGGVGGGAMGGGGAGANMAERRTIVQVKDENLGMSEKPDYFNVRATVVY
IKQENLYYTACASEGCNKKVNLDHENNWRCEKCDRSYATPEYRYILSTNVADATGQMWLS
GFNEDATQLIGMSAGELHKLREESESEFSAALHRAANRMYMFNCRAKMDTFNDTARVRYT ISRAAPVDFAKAGMELVDAIRAYM
>3C1YA 377 XRAY 2.10 0.223 0.257 no DNA integrity scanning protein disA <UNP DISA_THEMA> [THERMOTOGA MARITIMA]
MGSSHHHHHHSSGLVPRGSHMGVKSLVPQELIEKIKLISPGTELRKALDDIINANFGALI
FLVDDPKKYEDVIQGGFWLDTDFSAEKLYELSKMDGAIVLSEDITKIYYANVHLVPDPTI
PTGETGTRHRTAERLAKQTGKVVIAVSRRRNIISLYYKNYKYVVNQVDFLISKVTQAIST
LEKYKDNFNKLLSELEVLELENRVTLADVVRTLAKGFELLRIVEEIRPYIVELGEEGRLA
RMQLRELTEDVDDLLVLLIMDYSSEEVEEETAQNILQDFITRREPSPISISRVLGYDVQQ
AAQLDDVLVSARGYRLLKTVARIPLSIGYNVVRMFKTLDQISKASVEDLKKVEGIGEKRA
RAISESISSLKHRKTSE
>1C1KA 217 XRAY 1.45 0.205 0.253 no BPT4 GENE 59 HELICASE ASSEMBLY PROTEIN <UNP VG59_BPT4> [ENTEROBACTERIA PHAGE T4]
MIKLRMPAGGERYIDGKSVYKLYLMIKQHMNGKYDVIKYNWCMRVSDAAYQKRRDKYFFQ
KLSEKYKLKELALIFISNLVANQDAWIGDISDADALVFYREYIGRLKQIKFKFEEDIRNI
YYFSKKVEVSAFKEIFEYNPKVQSSYIFKLLQSNIISFETFILLDSFLNIIDKHDEQTDN
LVWNNYSIKLKAYRKILNIDSQKAKNVFIETVKSCKY
>3C1DA 159 XRAY 1.80 0.182 0.223 no Regulatory protein recX <UNP RECX_ECO57> [ESCHERICHIA COLI]
GPAYARLLDRAVRILAVRDHSEQELRRKLAAPIMGKNGPEEIDATAEDYERVIAWCHEHG
YLDDSRFVARFIASRSRKGYGPARIRQELNQKGISREATEKAMREADIDWAALARDQATR
KYGEPLPTVFSEKVKIQRFLLYRGYLMEDIQDIWRNFAD

>2QLCA 126 XRAY 2.30 0.219 0.274 no DNA repair protein radC homolog <UNP RADC_CHLTE> [CHLOROBIUM TEPIDUM TLS]
MNLKVKGARDVFEYMKGRIPDETKEHLFVLFLSTKNQILRHETITIGTLTASLIHPREIF
KAAIRESAHSIILVHNHPSGDVQPSNADKQVTSILKKAGDLLQIELLDHVIVGNNDWFSF
RDHALL
>1TZYB 126 XRAY 1.90 0.190 0.222 no Histone H2B <UNP H2B_CHICK> [GALLUS GALLUS] MPEPAKSAPAPKKGSKKAVTKTQKKGDKKRKKSRKESYSIYVYKVLKQVHPDTGISSKAM
GIMNSFVNDIFERIAGEASRLAHYNKRSTITSREIQTAVRLLLPGELAKHAVSEGTKAVT
KYTSSK
>1MOJA 182 XRAY 1.90 0.151 0.182 no Dps-like ferritin <UNP DPSA_HALN1> [HALOBACTERIUM SALINARUM]
MSTQKNARATAGEVEGSDALRMDADRAEQCVDALNADLANVYVLYHQLKKHHWNVEGAEF
RDLHLFLGEAAETAEEVADELAERVQALGGVPHASPETLQAEASVDVEDEDVYDIRTSLA
NDMAIYGDIIEATREHTELAENLGDHATAHMLREGLIELEDDAHHIEHYLEDDTLVTQGA
LE
>3BS3A 76 XRAY 1.65 0.185 0.219 no Putative DNA-binding protein <UNP Q5LGD2_BACFN> [BACTEROIDES FRAGILIS]
SNAMSNNQQMMLNRIKVVLAEKQRTNRWLAEQMGKSENTISRWCSNKSQPSLDMLVKVAE
LLNVDPRQLINGKIKI
>3EYIA 72 XRAY 1.45 0.157 0.194 no Z-DNA-binding protein 1 <UNP ZBP1_HUMAN> [HOMO SAPIENS]
HMASPQFSQQREEDIYRFLKDNGPQRALVIAQALGMRTAKDVNRDLYRMKSRHLLDMDEQ
SKAWTIYRWTIY
>3VZHA 244 XRAY 1.70 0.185 0.226 no Putative uncharacterized protein <UNP Q99YS3_STRP1> [STREPTOCOCCUS PYOGENES SEROTYPE M1]
GHMYRSRDFYVRVSGQRALFTNPATKGGSERSSYSVPTRQALNGIVDAIYYKPTFTNIVT
EVKVINQIQTELQGVRALLHDYSADLSYVSYLSDVVYLIKFHFVWNEDRKDLNSDRLPAK
HEAIMERSIRKGGRRDVFLGTRECLGLVDDISQEEYETTVSYYNGVNIDLGIMFHSFAYP
KDKKTPLKSYFTKTVMKNGVITFKAQSECDIVNTLSSYAFKAPEEIKSVNDECMEYDAME
KGEN
>3MU6A 71 XRAY 2.43 0.231 0.251 no Myocyte-specific enhancer factor 2A <UNP MEF2A_HUMAN> [HOMO SAPIENS]
GRKKIQITRIMDERNRQVTFTKRKFGLMKKAYELSVLCDCEIALIIFNSSNKLFQYASTD
MDKVLLKYTAY
>2D9HA 78 NMR NA NA NA no Zinc finger protein 692 <GB NP_060335> [HOMO SAPIENS]
GSSGSSGLQCEICGFTCRQKASLNWHQRKHAETVAALRFPCEFCGKRFEKPDSVAAHRSK
SHPALLLAPQESSGPSSG
>3VK0A 114 XRAY 1.88 0.184 0.230 no Transcriptional regulator <UNP Q7DDD9_NEIMB> [NEISSERIA MENINGITIDIS]
MMGNKLTLPAELPDEQDLRAVLAYNMRLFRVNKGWSQEELARQCGLDRTYVSAVERKRWN
IALSNIEKMAAALGVAAYQLLLPPQERLKLMTNSADTRQMPSESGILEHHHHHH
>3VEAA 151 XRAY 2.55 0.222 0.250 no Macrodomain Ter protein <UNP MATP_YERPE> [YERSINIA PESTIS]
MKYQQLENLESGWKWAYLVKKHREGEAITRHIENSAAQDAVEQLMKLENEPVKVQEWIDA
HMNVNLATRMKQTIRARRKRHFNAEHQHTRKKSIDLEFLVWQRLAVLARRRGNTLSDTVV
QLIEDAERKEKYASQMSSLKQDLKDILDKEV
>3V9RA 90 XRAY 2.40 0.242 0.290 no Uncharacterized protein YOL086W-A <UNP YO086_YEAST> [SACCHAROMYCES CEREVISIAE]
MNDDEDRAQLKARLWIRVEERLQQVLSSEDIKYTPRFINSLLELAYLQLGEMGSDLQAFA

RHAGRGVVNKSDLMLYLRKQPDLQERVTQE
>1P4EA 429 XRAY 2.70 0.236 0.275 no Recombinase FLP protein <UNP FLP_YEAST> [SACCHAROMYCES CEREVISIAE]
SQFDILCKTPPKVLVRQFVERFERPSGEKIASCAAELTYLCWMITHNGTAIKRATFMSYN
TIISNSLSFDIVNKSLQFKYKTQKATILEASLKKLIPAWEFTIIPYNGQKHQSDITDIVS
SLQLQFESSEEADKGNSHSKKMLKALLSEGESIWEITEKILNSFEYTSRFTKTKTLYQFL
FLATFINCGRFSDIKNVDPKSFKLVQNKYLGVIIQCLVTETKTSVSRHIYFFSARGRIDP
LVYLDEFLRNSEPVLKRVNRTGNSSSNKQEYQLLKDNLVRSYNKALKKNAPYPIFAIKNG
PKSHIGRHLMTSFLSMKGLTELTNVVGNFSDKRASAVARTTYTHQITAIPDHYFALVSRY
YAYDPISKEMIALKDETNPIEEWQHIEQLKGSAEGSIRYPAWNGIISQEVLDYLSSYINR
RIGHHHHHH
>4ABXA 175 XRAY 2.04 0.203 0.250 no DNA REPAIR PROTEIN RECN <UNP RECN_DEIRA> [DEINOCOCCUS RADIODURANS]
GIDPFTQRERARQIDLLAFQVQEISEVSPDPGEEEGLNTELSRLSNLHTIAQAAAGGVEL
LSDGDLNAAGLIGEAVRALNAGAKYDETVMQLQNELRAALESVQAIAGELRDVAEGSAAD
PEALDRVEARLSALSKLKNKYGPTLEDVVEFGAQAAEELAGLEEDERDAGSLQAD
>3GIOA 186 XRAY 2.40 0.233 0.241 no Putative uncharacterized protein <UNP O25318_HELPY> [HELICOBACTER PYLORI]
MGSSHHHHHHSSGLVPRGSHMPNTSQRNSFLQDVPYWMLQNRSEYITQGVDSSHIVDGKK
TEEIEKIATKRATIRVAQNIVHKLKEAYLSKTNRIKQKITNEMFIQMTQPIYDSLMNVDR
LGIYINPNNEEVFALVRARGFDKDALSEGLHKMSLDNQAVSILVAKVEEIFKDSVNYGDV
KVPIAM
>2KWQA 92 NMR NA NA NA no Protein MCM10 homolog <UNP MCM10_XENLA> [XENOPUS LAEVIS]
GPMGMQSIREQSCRVVTCKTCKYTHFKPKETCVSENHDFHWHNGVKRFFKCPCGNRTISL
DRLPKKHCSTCGLFKWERVGMLKEKTGPKLGG
>2KW3A 68 NMR NA NA NA no DNA-binding protein RFX5 <UNP RFX5_HUMAN> [HOMO SAPIENS]
GAGEPTTLLQRLRGTISKAVQNKVEGILQDVQKFSDNDKLYLYLQLPSGPTTGDKSSEPS
TLSNEEYM
>2KW3C 62 NMR NA NA NA no Regulatory factor X-associated protein <UNP RFXAP_HUMAN> [HOMO SAPIENS]
GHGTGSFGDRPARPTLLEQVLNQKRLSLLRSPEVVQFLQKQQQLLNQQVLEQRQQQFPGT
SM
>1KW4A 89 XRAY 1.75 0.220 0.234 no Polyhomeotic <UNP PHP_DROME> [DROSOPHILA MELANOGASTER]
METKRVNGTDRPPISSWSVDDVSNFIRELPGCQDYVDDFIQQEIDGQALLRLKEKHLVNA
MGMKLGPALKIVAKVESIKEVRDHHHHHH
>1KU9A 152 XRAY 2.80 0.245 0.280 no hypothetical protein MJ223 <UNP Y1563_METJA> [METHANOCALDOCOCCUS JANNASCHII]
MIIMEEAKKLIIELFSELAKIHGLNKSVGAVYAILYLSDKPLTISDIMEELKISKGNVSM
SLKKLEELGFVRKVWIKGERKNYYEAVDGFSSIKDIAKRKHDLIAKTYEDLKKLEEKCNE
EEKEFIKQKIKGIERMKKISEKILEALNDLDN
>2AQLA 173 XRAY 2.30 0.273 0.273 no Mortality factor 4-like protein 1 <UNP MO4L1_HUMAN> [HOMO SAPIENS]
MNRVEVKVKIPEELKPWLVDDWDLITRQKQLFYLPAKKNVDSILEDYANYKKSRGNTDNK
EYAVNEVVAGIKEYFNVMLGTQLLYKFERPQYAEILADHPDAPMSQVYGAPHLLRLFVRI
GAMLAYTPLDEKSLALLLNYLHDFLKYLAKNSATLFSASDYEVAPPEYHRKAV
>2L1PA 83 NMR NA NA NA no DNA-binding protein SATB1 <UNP SATB1_HUMAN> [HOMO SAPIENS]
MGHHHHHHSHMLPPEQWSHTTVRNALKDLLKDMNQSSLAKECPLSQSMISSIVNSTYYAN
VSAAKCQEFGRWYKHFKKTKDMM
>2NOGA 173 XRAY 2.00 0.216 0.274 no ISWI protein <UNP Q6DFM0_XENLA> [XENOPUS LAEVIS]

MVSEPKVPKAPRPPKQPNVQDFQFFPPRLFELLEKEILYYRKTIGYKVPRNPDLPNSAQV
QKEEQLKIDEAEPLNDEELEEKEKLLTQGFTNWNKRDFNQFIKANEKWGRDDIENIAREV
EGKTPEEVIEYSAVFWERCNELQDIEKTMAQIERGEARIQRRISILEHHHHHH
>3NO7A 80 XRAY 1.40 0.198 0.212 no Putative plasmid related protein <UNP Q6EEF9_LEIXC> [LEIFSONIA
XYLI SUBSP. CYNODONTIS]
GPEASARSEVKMTVTVGEERRARLRTAYTLTHLQEGHRTFSGFIAAALDAEVQRLEQRYN
EGRRFENAERGVTRGRPLGS
>3KDEC 77 XRAY 1.74 0.179 0.216 no Transposable element P transposase <UNP PELET_DROME>
[DROSOPHILA MELANOGASTER]
MKYCKFCCKAVTGVKLIHVPKCAIKRKLWEQSLGCSLGENSQICDTHFNDSQWKAAPAKG
QTFKRRRLNADAVPSKV
>2DQBA 376 XRAY 2.20 0.223 0.286 no Deoxyguanosinetriphosphate triphosphohydrolase, putative <UNP
Q5SL81_THET8> [THERMUS THERMOPHILUS]
MRFSREALLELEASRLAPYAQKARDTRGRAHPEPESLYRTPYQKDRDRILHTTAFRRLEY
KTQVLPGWAGDYYRTRLTHTLEVAQVSRSIARALGLNEDLTEAIALSHDLGHPPFGHTGE
HVLNALMQDHGGFEHNAQALRILTHLEVRYPGFRGLNLTYEVLEGIATHEAAYSPGFKPL
YEGQGTLEAQVVDLSDAIAYAAHDLDDGFRAGLLHPEELKEVELLQALALEEGLDLLRLP
ELDRRVLVRQLLGYFITAAIEATHRRVEEAGVQSAEAVRRHPSRLAALGEEAEKALKALK
AFLMERFYRHPEVLRERRKAEAVLEGLFAAYTRYPELLPREVQAKIPEEGLERAVCDYIA
GMTDRFALEAYRRLSP
>4BJIA 215 XRAY 1.45 0.152 0.170 no TRANSCRIPTION FACTOR TAU SUBUNIT SFC1 <UNP SFC1_SCHPO>
[SCHIZOSACCHAROMYCES POMBE]
GAMGKGQCRVWIITTNMGVESVPTCRHSKLGEPSKTIQEVIEALKPLFEKRPVWTRRALL
NHLDPSYTHYLKFALPYLSYLWTSGPFRDTYTRFGYDPRKDSNAAAYQALFFKLKLNGKH
KGTKTHVFDGKTLFPTNRVYQVCDIVDPTIAPLLKDTQLRSECHRDTGWYRSGRYYKVRD
LMREKLFALIEGEMPSEVAVNMILNAEEVEESDRY
>4BJ1A 319 XRAY 2.94 0.199 0.229 no PROTEIN RIF2 <UNP RIF2_YEAST> [SACCHAROMYCES CEREVISIAE]
GGGRVDHVFYQKFKSMALQELGTNYLSISYVPSLSKFLSKNLRSMKNCIVFFDKVEHIHQ
YAGIDRAVSETLSLVDINVVIIEMNDYLMKEGIQSSKSKECIESMGQASYSGQLDFEASE
KPSNHTSDLMMMVMRKINNDESIDHIVYFKFEQLDKLSTSTIIEPSKLTEFINVLSVLEK
SNNIAFKVLIYSNNVSISSLLSTSLKKKLNTKYTVFEMPILTCAQEQEYLKKMIKFTFDS
GSKLLQSYNSLVTCQLNNKESNLAIFFEFLKVFPHPFTYLFNAYTEIIVQSRTFDELLDK
IRNRLTIKNYPHSAYNFKK
>4OU0A 73 XRAY 1.40 0.199 0.238 no Replication protein A 32 kDa subunit <UNP RFA2_HUMAN>
[HOMO SAPIENS]
GPGSANGLTVAQNQVLNLIKAXPRPEGLNFQDLKNQLKHMSVSSIKQAVDFLSNEGHIYS
TVDDDHFKSTDAE
>1IGNA 246 XRAY 2.25 0.219 0.294 no RAP1 <PDB 1IGN> [SACCHAROMYCES CEREVISIAE]
GALPSHNKASFTDEEDEFILDVVRKNPTRRTTHTLYDEISHYVPNHTGNSIRHRFRVYLS
KRLEYVYEVDKFGKLVRDDDGNLIKTKVLPPSIKRKFSADEDYTLAIAVKKQFYRDLFQI
DPDTGRSLITDEDTPTAIARRNMTMDPNHVPGSEPNFAAYRTQSRRGPIAREFFKHFAEE
HAAHTENAWRDRFRKFLLAYGIDDYISYYEAEKAQNREPEPMKNLTNRPKRPGVPTPGNY
NSAAKR
>2L93A 55 NMR NA NA NA no DNA-binding protein H-NS <UNP HNS_SALTY> [SALMONELLA TYPHIMURIUM]
AARPAKYSYVDENGETKTWTGQGRTPAVIKKAMEEQGKQLEDFLIKELEHHHHHH
>3SJMA 64 XRAY 1.35 0.174 0.208 no Telomeric repeat-binding factor 2 <UNP TERF2_HUMAN> [HOMO SAPIENS] GSHMTTNITKKQKWTVEESEWVKAGVQKYGEGNWAAISKNYPFVNRTAVMIKDRWRTMKR

LGMN
>1UD9A 245 XRAY 1.68 0.202 0.238 no DNA polymerase sliding clamp A <UNP PCNA1_SULTO>
[SULFOLOBUS TOKODAII]
AHIVYDDVRDLKAIIQALLKLVDEALFDIKPEGIQLVAIDKAHISLIKIELPKEMFKEYD
VPEEFKFGFNTQYMSKLLKAAKRKEEIIIDADSPEVVKLTLSGALNRVFNVNNIEVLPPE
VPEVNLEFDIKATINASGLKNAIGEIAEVADTLLISGNEEKVVVKGEGENKVEVEFSKDT
GSLADIEFNKESSSAYDVEYLNDIISLTKLSDYVKVAFADQKPMQLEFNMEGGGKVTYLL
APKLS
>3EUSA 86 XRAY 1.80 0.204 0.254 no DNA-binding protein <UNP Q5LU41_SILPO> [SILICIBACTER POMEROYI]
QAMTKTLRTPEHVYLCQRLRQARLDAGLTQADLAERLDKPQSFVAKVETRERRLDVIEFA
KWMAACEGLDVVSEIVATIAEGRAQA
>3VU9A 245 XRAY 1.75 0.185 0.231 no Platinum sensitivity protein 3 <UNP PSY3_YEAST>
[SACCHAROMYCES
CEREVISIAE]
GSHMEVLKNIRIYPLSNFITSTKNYINLPNELRNLISEEQESKLGFLHIIESDFKPSVAL
QKLVNCTTGDEKILIIDIVSIWSQQKQRQHGAIYMNSLSCINITGLIVFLELLYDSPMDA
LRRCQVDNFNFQLRGIVIDNLSFLNFESDKNYDVINLSKFEKLFKILRKLREFLGCWIIT
KSFPTDFYNGIENTLVDKWSIKRKSGVTLYPTKLPDSYMKGMDLIIYREVVDGRPQYRRI
AALEE
>3VU9B 213 XRAY 1.75 0.185 0.231 no Chromosome segregation in meiosis protein 2 <UNP CSM2_YEAST> [SACCHAROMYCES CEREVISIAE]
MEYEDLELITIWPSPTKNKLCQFIKQNLSKEHVVTQLFFIDATSSFPLSQFQKLVPPTLP
ENVRIYENIRINTCLDLEELSAITVKLLQILSMNKINAQRGTEDAVTEPLKIILYINGLE
VMFRNSQFKSSPQRSHELLRDTLLKLRVMGNDENENASIRTLLEFPKEQLLDYYLKKNNN
TRTSSVRSKRRRIKNGDSLAEYIWKYYADSLFE
>1D4UA 111 NMR NA NA NA no NUCLEOTIDE EXCISION REPAIR PROTEIN XPA (XPA-MBD) <UNP XPA_HUMAN> [HOMO SAPIENS]
MEFDYVICEECGKEFMDSYLMDHFDLPTCDDCRDADDKHKLITKTEAKQEYLLKDCDLEK
REPPLKFIVKKNPHHSQWGDMKLYLKLQIVKRSLEVWGSQEALEEAKEVRQ
>4JJNK 382 XRAY 3.09 0.233 0.255 no Regulatory protein SIR3 <UNP SIR3_YEAST>
[SACCHAROMYCES CEREVISIAE]
SAKTLKDLDGWQVIITDDQGRVIDDNNRRRSRKRGGENVFLKRISDGLSFGKGESVIFND
NVTETYSVYLIHEIRLNTLNNVVEIWVFSYLRWFELKPKLYYEQFRPDLIKEDHPLEFYK
DKFFNEVNKSELYLTAELSEIWLKDFIAVGQILPESQWNDSSIDKIEDRDFLVRYACEPT
AEKFVPIDIFQIIRRVKEMEPKQSNEYLKRVSVPVSGQKTNRQVMHKMGVERSSKRLAKK
PSMKKIKIEPSADDDVNNGNIPSQRGTSTTHGSISPQEESVSPNISSASPSALTSPTDSS
KILQKRSISKELIVSEEIPINSSEQESDYEPNNETSVLSSKPGSKPEKTSTELVDGRENF
VYANNPEVSDDGGLEEETDEVS
>3V72A 335 XRAY 2.49 0.299 0.291 no DNA polymerase beta <UNP DPOLB_RAT> [RATTUS NORVEGICUS]
MSKRKAPQETLNGGITDMLVELANFEKNVSQAIHKYNAYRKAASVIAKYPHKIKSGAEAK
KLPGVGTKIAEKIDEFLATGKLRKLEKIRQDDTSSSINFLTRVTGIGPSAARKLVDEGIK
TLEDLRKNEDKLNHHQRIGLKYFEDFEKRIPREEMLQMQDIVLNEVKKLDPEYIATVCGS
FRRGAESSGDMDVLLTHPNFTSESSKQPKLLHRVVEQLQKVRFITDTLSKGETKFMGVCQ
LPSENDENEYPHRRIDIRLIPKDQYYCGVLYFTGSDIFNKNMRAHALEKGFTINKYTIRP
LGVTGVAGEPLPVDSEQDIFDYIQWRYREPKDRSE
>1XVPB 246 XRAY 2.60 0.180 0.234 no Orphan nuclear receptor NR1I3 <UNP NR1I3_HUMAN> [HOMO SAPIENS]
PVQLSKEQEELIRTLLGAHTRHMGTMFEQFVQFRPPAHLFIHHQPLPTLAPVLPLVTHFA

DINTFMVLQVIKFTKDLPVFRSLPIEDQISLLKGAAVEICHIVLNTTFCLQTQNFLCGPL
RYTIEDGARVGFQVEFLELLFHFHGTLRKLQLQEPEYVLLAAMALFSPDRPGVTQRDEID
QLQEEMALTLQSYIKGQQRRPRDRFLYAKLLGLLAELRSINEAYGYQIQHIQGLSAMMPL
LQEICS
>2LUAA 52 NMR NA NA NA no Protein male-specific lethal-2 <UNP MSL2_DROME> [DROSOPHILA MELANOGASTER]
SPPKPKCRCGISGSSNTLTTCRNSRCPCYKSYNSCAGCHCVGCKNPHKEDYV
>2LUYA 97 NMR NA NA NA no Meiotic chromosome segregation protein P8B7.28c <UNP YORS_SCHPO> [SCHIZOSACCHAROMYCES POMBE]
HMGKNDNDALIMCMRCRKVKGIDSYSKTQWSKTFTFVRGRTVSVSDPKVICRTCQPKQHD
SIWCTACQQTKGINEFSKAQRHVLDPRCQICVHSQRN
>1B8ZA 90 XRAY 1.60 0.215 0.236 no HISTONELIKE PROTEIN HU <UNP DBH_THEMA> [THERMOTOGA MARITIMA]
MNKKELIDRVAKKAGAKKKDVKLILDTILETITEALAKGEKVQIVGFGSFEVRKAAARKG
VNPQTRKPITIPERKVPKFKPGKALKEKVK
>1WIJA 140 NMR NA NA NA no ETHYLENE-INSENSITIVE3-like 3 protein <UNP EIL3_ARATH> [ARABIDOPSIS THALIANA]
GSSGSSGSQFVLQDLQDATLGSLLSSLMQHCDPPQRKYPLEKGTPPPWWPTGNEEWWVKL
GLPKSQSPPYRKPHDLKKMWKVGVLTAVINHMLPDIAKIKRHVRQSKCLQDKMTAKESAI
WLAVLNQEESLIQQSGPSSG
>1Q87A 221 XRAY 2.32 0.220 0.274 no 39 kDa initiator binding protein <UNP Q95VR4_TRIVA> [TRICHOMONAS VAGINALIS] PVNTKRSNGTKRVEFPTTKKSMCIGNSTPNEQETFRAKVDEIWFRLTQKTDGTVMRDFLI
EKAAEYFKQPEQPKQNAIEVISAIMAPQEEQTKSKADLYKFLAMFGPYETIMLKIASLLL
ISNNKGHWLTFDPQAEKNANNQRDSISGWFDQNEPNCLILKTPTGIRKIWNKPLIEATGQ
YLMDENGEKYDSWDKYFEMKPIETYLTAYPTFAPMHHHHHH
>2HGVA 164 XRAY 2.30 0.168 0.244 no GTP-sensing transcriptional pleiotropic repressor codY <UNP CODY_BACSU> [BACILLUS SUBTILIS]
GSSHHHHHHMALLQKTRIINSMLQAAAGKPVNFKEMAETLRDVIDSNIFVVSRRGKLLGY
SINQQIENDRMKKMLEDRQFPEEYTKNLFNVPETSSNLDINSEYTAFPVENRDLFQAGLT
TIVPIIGGGERLGTLILSRLQDQFNDDDLILAEYGATVVGMEIL
>2OXOA 103 XRAY 2.00 0.234 0.289 no Integrase <UNP VINT_LAMBD> [UNIDENTIFIED PHAGE]
MTLHSWLDRYEKILASRGIKQKTLINYMSKIKAIRRGLPDAPLEDITTKEIAAMLNGYID
EGKAASAKLIRSTLSDAFREAIAEGHITTNHVAATRAAKSEVR
>3LHKA 154 XRAY 2.20 0.206 0.249 no putative DNA binding protein MJ0014 <UNP Y014_METJA> [METHANOCALDOCOCCUS JANNASCHII]
SNAKIIGYARVSFNAQKDDLERQIQLIKSYAEENGWDIQILKDIGSGLNEKRKNYKKLLK
MVMNRKVEKVIIAYPDRLTRFGFETLKEFFKSYGTEIVIINKKHKTPQEELVEDLITIVS
HFAGKLYGMHSHKYKKLTKTVKEIVREEDAKEKE
>1JE5A 206 XRAY 1.90 0.221 0.268 no HELIX-DESTABILIZING PROTEIN <UNP VHED_BPT7> [ENTEROBACTERIA PHAGE T7]
MAKKIFTSALGTAEPYAYIAKPDYGNEERGFGNPRGVYKVDLTIPNKDPRCQRMVDEIVK
CHEEAYAAAVEEYEANPPAVARGKKPLKPYEGDMPFFDNGDGTTTFKFKCYASFQDKKTK
ETKHINLVVVDSKGKKMEDVPIIGGGSKLKVKYSLVPYKWNTAVGASVKLQLESVMLVEL
ATFGGGEDDWADEVEENGYVASGSAK
>1JEYA 609 XRAY 2.50 0.220 0.280 no Ku70 <UNP KU70_HUMAN> [HOMO SAPIENS]
MSGWESYYKTEGDEEAEEEQEENLEASGDYKYSGRDSLIFLVDASKAMFESQSEDELTPF
DMSIQCIQSVYISKIISSDRDLLAVVFYGTEKDKNSVNFKNIYVLQELDNPGAKRILELD
QFKGQQGQKRFQDMMGHGSDYSLSEVLWVCANLFSDVQFKMSHKRIMLFTNEDNPHGNDS

AKASRARTKAGDLRDTGIFLDLMHLKKPGGFDISLFYRDIISIAEDEDLRVHFEESSKLE
DLLRKVRAKETRKRALSRLKLKLNKDIVISVGIYNLVQKALKPPPIKLYRETNEPVKTKT
RTFNTSTGGLLLPSDTKRSQIYGSRQIILEKEETEELKRFDDPGLMLMGFKPLVLLKKHH
YLRPSLFVYPEESLVIGSSTLFSALLIKCLEKEVAALCRYTPRRNIPPYFVALVPQEEEL
DDQKIQVTPPGFQLVFLPFADDKRKMPFTEKIMATPEQVGKMKAIVEKLRFTYRSDSFEN
PVLQQHFRNLEALALDLMEPEQAVDLTLPKVEAMNKRLGSLVDEFKELVYPPDYNPEGKV
TKRKHDNEGSGSKRPKVEYSEEELKTHISKGTLGKFTVPMLKEACRAYGLKSGLKKQELL
EALTKHFQD
>1JEYB 565 XRAY 2.50 0.220 0.280 no Ku80 <UNP KU86_HUMAN> [HOMO SAPIENS]
MVRSGNKAAVVLCMDVGFTMSNSIPGIESPFEQAKKVITMFVQRQVFAENKDEIALVLFG
TDGTDNPLSGGDQYQNITVHRHLMLPDFDLLEDIESKIQPGSQQADFLDALIVSMDVIQH
ETIGKKFEKRHIEIFTDLSSRFSKSQLDIIIHSLKKCDISLQFFLPFSLGKEDGSGDRGD
GPFRLGGHGPSFPLKGITEQQKEGLEIVKMVMISLEGEDGLDEIYSFSESLRKLCVFKKI
ERHSIHWPCRLTIGSNLSIRIAAYKSILQERVKKTWTVVDAKTLKKEDIQKETVYCLNDD
DETEVLKEDIIQGFRYGSDIVPFSKVDEEQMKYKSEGKCFSVLGFCKSSQVQRRFFMGNQ
VLKVFAARDDEAAAVALSSLIHALDDLDMVAIVRYAYDKRANPQVGVAFPHIKHNYECLV
YVQLPFMEDLRQYMFSSLKNSKKYAPTEAQLNAVDALIDSMSLAKKDEKTDTLEDLFPTT
KIPNPRFQRLFQCLLHRALHPREPLPPIQQHIWNMLNPPAEVTTKSQIPLSKIKTLFPLI
EAKKKDQVTAQEIFQDNHEDGPTAK
>1QZQA 483 XRAY 2.40 0.185 0.234 no tyrosyl-DNA phosphodiesterase 1 <UNP TYDP1_HUMAN> [HOMO SAPIENS]
MEEYMPTEHHHHHHENLYFQGTSGEGQDIWDMLDKGNPFQFYLTRVSGVKPKYNSGALHI
KDILSPLFGTLVSSAQFNYCFDVDWLVKQYPPEFRKKPILLVHGDKREAKAHLHAQAKPY
ENISLCQAKLDIAFGTHHTKMMLLLYEEGLRVVIHTSNLIHADWHQKTQGIWLSPLYPRI
ADGTHKSGESPTHFKADLISYLMAYNAPSLKEWIDVIHKHDLSETNVYLIGSTPGRFQGS
QKDNWGHFRLKKLLKDHASSMPNAESWPVVGQFSSVGSLGADESKWLCSEFKESMLTLGK
ESKTPGKSSVPLYLIYPSVENVRTSLEGYPAGGSLPYSIQTAEKQNWLHSYFHKWSAETS
GRSNAMPHIKTYMRPSPDFSKIAWFLVTSANLSKAAWGALEKNGTQLMIRSYELGVLFLP
SAFGLDSFKVKQKFFAGSQEPMATFPVPYDLPPELYGSKDRPWIWNIPYVKAPDTHGNMW
VPS
>1N1JB 97 XRAY 1.67 0.181 0.206 no NF-YC <UNP NFYC_HUMAN> [HOMO SAPIENS]
GSHMEEIRNLTVKDFRVQELPLARIKKIMKLDEDVKMISAEAPVLFAKAAQIFITELTLR
AWIHTEDNKRRTLQRNDIAMAITKFDQFDFLIDIVPR
>2LTTA 80 NMR NA NA NA no Putative uncharacterized protein ydbC <UNP Q9CIP3_LACLA> [LACTOCOCCUS LACTIS SUBSP. LACTIS]
MADKLKFEIIEELIVLSENAKGWRKELNRVSWNDAEPKYDIRTWSPDHEKMGKGITLSEE
EFGVLLKELGNKLEHHHHHH
>4ICGC 75 XRAY 2.92 0.283 0.332 no Hemolysin expression-modulating protein <UNP F5ZMH5_SALTU> [SALMONELLA ENTERICA SUBSP. ENTERICA SEROVAR TYPHIMURIUM]
GSHMSDKPLTKTDYLMRLRRCQTIDTLERVIEKNKYELSDNELAVFYSAADHRLAELTMN
KLYDKIPSSVWKFIR
>2M8EA 57 NMR NA NA NA no SLEEPING BEAUTY TRANSPOSASE <PDB 2M8E> [SYNTHETIC CONSTRUCT]
ASMGKSKEISQDLRKKIVDLHKSGSSLGAISKRLKVPRSSVQTIVRKYKHHGTTQHH
>3M8EA 124 XRAY 2.00 0.240 0.270 no Putative DNA-binding protein <UNP Q8KNP2_BACTI> [BACILLUS THURINGIENSIS]
MGSSHHHHHHSSGLVPRGSHMNRDHFYTLNIAEIAERIGNDDCAYQVLMAFINENGEAQM
LNKTAVAEMIQLSKPTVFATVNSFYCAGYIDETRVGRSKIYTLSDLGVEIVECFKQKAME
MRNL
>1SD4A 126 XRAY 2.00 0.210 0.237 no PENICILLINASE REPRESSOR <UNP Q6UB84_STAAU>

[STAPHYLOCOCCUS AUREUS]
MTNKQVEISMAEWDVMNIIWDKKSVSANEIVVEIQKYKEVSDKTIRTLITRLYKKEIIKR
YKSENIYFYSSNIKEDDIKMKTAKTFLNKLYGGDMKSLVLNFAKNEELNNKEIEELRDIL
NDISKK
>2F7NA 207 XRAY 2.00 0.200 0.220 no DNA-binding stress response protein, Dps family <GB NP_295984> [DEINOCOCCUS RADIODURANS]
MTKKSTKSEAASKTKKSGVPETGAQGVRAGGADHADAAHLGTVNNALVNHHYLEEKEFQT
VAETLQRNLATTISLYLKFKKYHWDIRGRFFRDLHLAYDEFIAEIFPSIDEQAERLVALG
GSPLAAPADLARYSTVQVPQETVRDARTQVADLVQDLSRVGKGYRDDSQACDEANDPVTA
DMYNGYAATIDKIRWMLQAIMDDERLD
>2LVSA 105 NMR NA NA NA no Putative uncharacterized protein <UNP A2BLH2_HYPBU> [HYPERTHERMUS BUTYLICUS]
MPSVNDSLDIVEKLYKDGVPVKEIAKRSNNSMSTVYKALEKLEAMGRIKRRKGRYRQHRR
LTEEELATIRELYLKGATVYEIARQLGRPESTIYYALKKLGLKLE
>4G63A 470 XRAY 2.70 0.225 0.258 no Cytosolic IMP-GMP specific 5'-nucleotidase <UNP Q5ZZB6_LEGPH> [LEGIONELLA PNEUMOPHILA SUBSP. PNEUMOPHILA]
MDTHKVFVNRIINMRKIKLIGLDMDHTLIRYNSKNFESLVYDLVKERLAESFHYPEEIKK
FKFNFDDAIRGLVIDSKNGNILKLSRYGAIRLSYHGTKQISFSDQKKIYRSIYVDLGDPN
YMAIDTSFSIAFCILYGQLVDLKDTNPDKMPSYQAIAQDVQYCVDKVHSDGTLKNIIIKN
LKKYVIREKEVVEGLKHFIRYGKKIFILTNSEYSYSKLLLDYALSPFLDKGEHWQGLFEF
VITLANKPRFFYDNLRFLSVNPENGTMTNVHGPIVPGVYQGGNAKKFTEDLGVGGDEILY
IGDHIYGDILRLKKDCNWRTALVVEELGEEIASQIRALPIEKKIGEAMAIKKELEQKYVD
LCTRSIDESSQQYDQEIHDLQLQISTVDLQISRLLQEQNSFYNPKWERVFRAGAEESYFA
YQVDRFACIYMEKLSDLLEHSPMTYFRANRRLLAHDIDIAAALEHHHHHH
>4G6DA 73 XRAY 2.00 0.206 0.243 no RNA polymerase sigma factor rpoD <UNP RPOD_STAA8> [STAPHYLOCOCCUS AUREUS SUBSP. AUREUS]
MKEQLEDVLDTLTDREENVLRLRFGLDDGRTRTLEEVGKVFGVTRERIRQIEAKALRKLR
HPSRSKRLKDFMD
>4G6DB 198 XRAY 2.00 0.206 0.243 no ORF067 <UNP Q4Z9Y5_9CAUD> [STAPHYLOCOCCUS PHAGE G1]
MKLKILDKDNATLNVFHRNKEHKTIDNVPTANLVDWYPLSNAYEYKLSRNGEYLELKRLR
STLPSSYGLDDNNQDIIRDNNHRCKIGYWYNPAVRKDNLKIIEKAKQYGLPIITEEYDAN
TVEQGFRDIGVIFQSLKTIVVTRYLEGKTEEELRIFNMKSEESQLNEALKESDFSVDLTY
SDLGQIYNMLLLMKKISK
>4E0GA 462 XRAY 2.20 0.196 0.238 no Protelomerase <UNP Q7CWV1_AGRT5> [AGROBACTERIUM TUMEFACIENS]
MGSSHHHHHHSSGLVPRGSHMLAAKRKTKTPVLVERIDQFVGQIKEAMKSDDASRNRKIR
DLWDAEVRYHFDNGRTEKTLELYIMKYRNALKAEFGPKSTPLAICNMKKLRERLNTYIAR
GDYPKTGVATSIVEKIERAEFNTAGRKPTVLLRIADFIAAMNGMDAKQDMQALWDAEIAI
MNGRAQTTIISYITKYRNAIREAFGDDHPMLKIATGDAAMYDEARRVKMEKIANKHGALI
TFENYRQVLKICEDCLKSSDPLMIGIGLIGMTGRRPYEVFTQAEFSPAPYGKGVSKWSIL
FNGQAKTKQGEGTKFGITYEIPVLTRSETVLAAYKRLRESGQGKLWHGMSIDDFSSETRL
LLRDTVFNLFEDVWPKEELPKPYGLRHLYAEVAYHNFAPPHVTKNSYFAAILGHNNNDLE
TSLSYMTYTLPEDRDNALARLKRTNERTLQQMATIAPVSRKG
>2KKVA 121 NMR NA NA NA no Integrase <UNP Q5PJC5_SALPA> [SALMONELLA ENTERICA SUBSP. ENTERICA SEROVAR PARATYPHI A]
MENSGAYTFETIAREWHESNKRWSEDHRSRVLRYLELYIFPHIGSSDIRQLKTSHLLAPI
KEVDTSGKHDVAQRLQQRVTAIMRYAVQNDYIDSNPASDMAGALSTTKARHYPLEHHHHH
H

>2KKOA 108 NMR NA NA NA no POSSIBLE TRANSCRIPTIONAL REGULATORY PROTEIN (POSSIBLY ARSR- FAMILY) <UNP Q7U294_MYCBO> [MYCOBACTERIUM BOVIS]
MAGQSDRKAALLDQVARVGKALANGRRLQILDLLAQGERAVEAIATATGMNLTTASANLQ
ALKSGGLVEARREGTRQYYRIAGEDVARLFALVQVVADEHLEHHHHHH
>1C8CA 64 XRAY 1.45 0.229 0.287 no DNA-BINDING PROTEIN 7A <UNP DN72_SULSO> [SULFOLOBUS SOLFATARICUS]
MATVKFKYKGEEKQVDISKIKKVWRVGKMISFTYDEGGGKTGRGAVSEKDAPKELLQMLA
KQKK
>1C6VX 81 XRAY 3.00 0.203 0.362 no SIU89134 <UNP Q87706_SIVCZ> [SIMIAN IMMUNODEFICIENCY VIRUS]
QQSKNSKFKNFRVYYREGRDQLWKGPGELLWKGEGAVLLKVGTDIKVVPRRKAKIIKDYG
GGKEVDSSSHMEDTGEAREVA
>1YSEA 141 NMR NA NA NA no DNA-binding protein SATB1 <UNP SATB1_HUMAN> [HOMO SAPIENS]
GSHVSRSMNKPLEQQVSTNTEVSSEIYQWVRDELKRAGISQAVFARVAFNRTQGLLSEIL
RKEEDPKTASQSLLVNLRAMQNFLQLPEAERDRIYQDERERSLNAASAMGPAPLISTPPS
RPPQVKTATIATERNGKPENN
>2RT6A 98 NMR NA NA NA no Primosomal replication protein N' <UNP PRIC_ECOLI> [ESCHERICHIA COLI]
MKTALLLEKLEGQLATLRQRCAPVSQFATLSARFDRHLFQTRATTLQACLDEAGDNLAAL
RHAVEQQQLPQVAWLAEHLAAQLEAIAREASAWSLREW
>3BOSA 242 XRAY 1.75 0.168 0.201 no Putative DNA replication factor <UNP A1S6W5_SHEAM> [SHEWANELLA AMAZONENSIS]
GMRSNRVTQHPPLQLSLPVHLPDDETFTSYYPAAGNDELIGALKSAASGDGVQAIYLWGP
VKSGRTHLIHAACARANELERRSFYIPLGIHASISTALLEGLEQFDLICIDDVDAVAGHP
LWEEAIFDLYNRVAEQKRGSLIVSASASPMEAGFVLPDLVSRMHWGLTYQLQPMMDDEKL
AALQRRAAMRGLQLPEDVGRFLLNRMARDLRTLFDVLDRLDKASMVHQRKLTIPFVKEML
RL
>1NGNA 155 XRAY 2.10 0.213 0.263 no methyl-CpG binding protein MBD4 <UNP MBD4_MOUSE> [MUS MUSCULUS]
ALSPPRRKSFKKWTPPRSPFNLVQEILFHDPWKLLIATIFLNRTSGKMAIPVLWEFLEKY
PSAEVARAADWRDVSELLKPLGLYDLRAKTIIKFSDEYLTKQWRYPIELHGIGKYGNDSY
RIFCVNEWKQVHPEDHKLNKYHDWLWENHEKLSLS
>3FDQA 170 XRAY 1.75 0.217 0.232 no Motility gene repressor mogR <UNP MOGR_LISMO> [LISTERIA MONOCYTOGENES]
MPKSEIRKLLQEIKKQVDNPGNSSTTEIKKMASEAGIDEQTAEEIYHLLTEFYQAVEEHG
GIEKYMHSNISWLKIELELLSACYQIAILEDMKVLDISEMLSLNDLRIFPKTPSQLQNTY
YKLKKELIQVEDIPKNKPGRKRKTQKNTKKEKTNIFGKVVPALEHHHHHH
>2FZ4A 237 XRAY 2.40 0.243 0.265 no DNA repair protein RAD25 <UNP O29889_ARCFU> [ARCHAEOGLOBUS FULGIDUS]
MGSSHHHHHHSSGLVPRGSHMQMIAEIYYERGTIVVKGDAHVPHAKFDSRSGTYRALAFR
YRDIIEYFESNGIEFVDNAADPIPTPYFDAEISLRDYQEKALERWLVDKRGCIVLPTGSG
KTHVAMAAINELSTPTLIVVPTLALAEQWKERLGIFGEEYVGEFSGRIKELKPLTVSTYD
SAYVNAEKLGNRFMLLIFDEVHHLPAESYVQIAQMSIAPFRLGLTATFEREDGRHEI
>1X3CA 73 NMR NA NA NA no Zinc finger protein 292 <UNP ZN292_HUMAN> [HOMO SAPIENS]
GSSGSSGRKKPVSQSLEFPTRYSPYRPYRCVHQGCFAAFTIQQNLILHYQAVHKSDLPAF
SAEVEEESGPSSG
>4ATHA 83 XRAY 1.95 0.196 0.228 no MICROPHTHALMIA-ASSOCIATED TRANSCRIPTION FACTOR <UNP MITF_MOUSE> [MUS MUSCULUS]
GAMRFNINDRIKELGTLIPKSNDPDMRWNKGTILKASVDYIRKLQREQQRAKDLENRQKK
LEHANRHLLLRVQELEMQARAHG

>1ZAEA 70 NMR NA NA NA no Early protein GP16.7 <UNP VG167_BPPH2> [BACILLUS PHAGE PHI29]
HMDKTVNLSACEVAVLDLYEQSNIRIPSDIIEDLVNQRLQSEQEVLNYIETQRTYWKLEN
QKKLYRGSLK
>2QPYA 251 XRAY 2.50 0.269 0.269 no Androgen receptor <UNP ANDR_MOUSE> [MUS MUSCULUS]
CQPIFLNVLEAIEPGVVCAGHDNNQPDSFAALLSSLNELGERQLVHVVKWAKALPGFRNL
HVDDQMAVIQYSWMGLMVFAMGWRSFTNVNSRMLYFAPDLVFNEYRMHKSRMYSQCVRMR
HLSQEFGWLQITPQEFLCMKALLLFSIIPVDGLKNQKFFDELRMNYIKELDRIIACKRKN
PTSCSRRFYQLTKLLDSVQPIARELHQFTFDLLIKSHMVSVDFPEMMAEIISVQVPKILS
GKVKPIYFHTQ
>1G5HA 454 XRAY 1.95 0.183 0.224 no MITOCHONDRIAL DNA POLYMERASE ACCESSORY SUBUNIT <UNP DPOG2_MOUSE> [MUS MUSCULUS]
WLSGYAGPADGTQQPDAPEHAVAREALVDLCRRRHFLSGTPQQLSTAALLSGCHARFGPL
GVELRKNLASQWWSSMVVFREQVFAVDSLHQEPGSSQPRDSAFRLVSPESIREILQDREP
SKEQLVAFLENLLKTSGKLRATLLHGALEHYVNCLDLVNRKLPFGLAQIGVCFHPVSNSN
QTPSSVTRVGEKTEASLVWFTPTRTSSQWLDFWLRHRLLWWRKFAMSPSNFSSADCQDEL
GRKGSKLYYSFPWGKEPIETLWNLGDQELLHTYPGNVSTIQGRDGRKNVVPCVLSVSGDV
DLGTLAYLYDSFQLAENSFARKKSLQRKVLKLHPCLAPIKVALDVGKGPTVELRQVCQGL
LNELLENGISVWPGYSETVHSSLEQLHSKYDEMSVLFSVLVTETTLENGLIQLRSRDTTM
KEMMHISKLRDFLVKYLASASNVAAALDHHHHHH
>2XE0A 152 XRAY 2.31 0.215 0.256 no I-CREI V2V3 VARIANT <PDB 2XE0> [CHLAMYDOMONAS REINHARDTII]
NTKYNKEFLLYLAGFVDGDGSIIAQINPNASSKFKHRLRLTFYVTQKTQRRWFLDKLVDE
IGVGYVRDSGSVSQYVLSEIAPLHNFLTQLQPFLKLKQKQANLVLKIIEQLPSAKASPAA
FLEVCTWVDQIAALNDSATRKTTSETVAAVLD
>4M6WA 221 XRAY 2.90 0.257 0.299 no Fanconi anemia group M protein <UNP FANCM_HUMAN> [HOMO SAPIENS]
MGQEGKGTCILVGGHEITSGLEVISSLRAIHGLQVEVCPLNGCDYIVSNRMVVERRSQSE
MLNSVNKNKFIEQIQHLQSMFERICVIVEKDREKTGDTSRMFRRTKSYDSLLTTLIGAGI
RILFSSCQEETADLLKELSLVEQRKNVGIHVPTVVNSNKSEALQFYLSIPNISYITALNM
CHQFSSVKRMANSSLQEISMYAQVTHQKAEEIYRYIHYVFD
>4M6WB 208 XRAY 2.90 0.257 0.299 no Fanconi anemia-associated protein of 24 kDa <UNP FAP24_HUMAN> [HOMO SAPIENS]
MGHIVANEKWRGSQLAQEMQGKIKLIFEDGLTPDFYLSNRCCILYVTEADLVAGNGYRKR
LVRVRNSNNLKGIVVVEKTRMSEQYFPALQKFTVLDLGMVLLPVASQMEASCLVIQLVQE
QTKEPSKNPLLGKKRALLLSEPSLLRTVQQIPGVGKVKAPLLLQKFPSIQQLSNASIGEL
EQVVGGQAVAQQIHAFFTQPRLEHHHHHH
>2ADLA 72 NMR NA NA NA no CcdA <UNP Q9S0Z5_ECOLI> [ESCHERICHIA COLI]
MKQRITVTVDSDSYQLLKAYDVNISGLVSTTMQNEARRLRAERWKVENQEGMVEVARFIE
MNGSFADENKDW
>1WEUA 91 NMR NA NA NA no inhibitor of growth family, member 4 <UNP ING4_MOUSE> [MUS MUSCULUS]
GSSGSSGSPEYGMPSVTFGSVHPSDVLDMPVDPNEPTYCLCHQVSYGEMIGCDNPDCSIE
WFHFACVGLTTKPRGKWFCPRCSQESGPSSG
>1WEOA 93 NMR NA NA NA no cellulose synthase, catalytic subunit (IRX3) <UNP CESA7_ARATH> [ARABIDOPSIS THALIANA]
GSSGSSGPKPLKNLDGQFCEICGDQIGLTVEGDLFVACNECGFPACRPCYEYERREGTQN
CPQCKTRYKRLRGSPRVEGDEDEEDIDSGPSSG
>3WE2A 147 XRAY 2.70 0.237 0.271 no Bloom syndrome protein <UNP BLM_HUMAN> [HOMO SAPIENS]
GPLGSKTKDYKTRDVTDDVKSIVRFVQEHSSSQGMRNIKHVGPSGRFTMNMLVDIFLGSK

SAKIQSGIFGKGSAYSRHNAERLFKKLILDKILDEDLYINANDQAIAYVMLGNKAQTVLN
GNLKVDFMETENSSSVKKQKALVAKVS
>2P2UA 171 XRAY 2.75 0.245 0.290 no Host-nuclease inhibitor protein Gam, putative <UNP Q72CZ5_DESVH> [DESULFOVIBRIO VULGARIS]
SLSRRKPNPVIVADIRQAEGALAEIATIDRKVGEIEAQMNEAIDAAKARASQKSAPLLAR
RKELEDGVATFATLNKTEMFKDRKSLDLGFGTIGFRLSTQIVQMSKITKDMTLERLRQFG
ISEGIRIKEDVNKEAMQGWPDERLEMVGLKRRTTDAFYIEINREEVADTAA
>2LW1A 89 NMR NA NA NA no ABC transporter ATP-binding protein uup <UNP UUP_ECOLI> [ESCHERICHIA COLI]
GSHMKAETVKRSSSKLSYKLQRELEQLPQLLEDLEAKLEALQTQVADASFFSQPHEQTQK
VLADMAAAEQELEQAFERWEYLEALKNGG
>3THOA 382 XRAY 2.61 0.206 0.242 no Probable DNA double-strand break repair Rad50 ATPase <UNP RAD50_THEMA> [THERMOTOGA MARITIMA]
HHHHHHSSGENLYFQGHMRPERLTVRNFLGLKNVDIEFQSGITVVEGPNGAGKSSLFEAI
SFALFGNGIRYPNSYDYVNRNAVDGTARLVFQFERGGKRYEIIREINALQRKHNAKLSEI
LENGKKAAIAAKPTSVKQEVEKILGIEHRTFIRTVFLPQGEIDKLLISPPSEITEIISDV
FQSKETLEKLEKLLKEKMKKLENEISSGGAGGAGGSLEKKLKEMSDEYNNLDLLRKYLFD
KSNFSRYFTGRVLEAVLKRTKAYLDILTNGRFDIDFDDEKGGFIIKDWGIERPARGLSGG
ERALISISLAMSLAEVASGRLDAFFIDEGFSSLCTENKEKIASVLKELERLNKVIVFITC
DREFSEAFDRKLRITGGVVVNE
>3THXA 934 XRAY 2.70 0.212 0.273 no DNA mismatch repair protein Msh2 <UNP MSH2_HUMAN> [HOMO SAPIENS]
MAVQPKETLQLESAAEVGFVRFFQGMPEKPTTTVRLFDRGDFYTAHGEDALLAAREVFKT
QGVIKYMGPAGAKNLQSVVLSKMNFESFVKDLLLVRQYRVEVYKNRAGNKASKENDWYLA
YKASPGNLSQFEDILFGNNDMSASIGVVGVKMSAVDGQRQVGVGYVDSIQRKLGLCEFPD
NDQFSNLEALLIQIGPKECVLPGGETAGDMGKLRQIIQRGGILITERKKADFSTKDIYQD
LNRLLKGKKGEQMNSAVLPEMENQVAVSSLSAVIKFLELLSDDSNFGQFELTTFDFSQYM
KLDIAAVRALNLFQGSVEDTTGSQSLAALLNKCKTPQGQRLVNQWIKQPLMDKNRIEERL
NLVEAFVEDAELRQTLQEDLLRRFPDLNRLAKKFQRQAANLQDCYRLYQGINQLPNVIQA
LEKHEGKHQKLLLAVFVTPLTDLRSDFSKFQEMIETTLDMDQVENHEFLVKPSFDPNLSE
LREIMNDLEKKMQSTLISAARDLGLDPGKQIKLDSSAQFGYYFRVTCKEEKVLRNNKNFS
TVDIQKNGVKFTNSKLTSLNEEYTKNKTEYEEAQDAIVKEIVNISSGYVEPMQTLNDVLA
QLDAVVSFAHVSNGAPVPYVRPAILEKGQGRIILKASRHACVEVQDEIAFIPNDVYFEKD
KQMFHIITGPNMGGKSTYIRQTGVIVLMAQIGCFVPCESAEVSIVDCILARVGAGDSQLK
GVSTFMAEMLETASILRSATKDSLIIIDELGRGTSTYDGFGLAWAISEYIATKIGAFCMF
ATHFHELTALANQIPTVNNLHVTALTTEETLTMLYQVKKGVCDQSFGIHVAELANFPKHV
IECAKQKALELEEFQYIGESQGYDIMEPAAKKCYLEREQGEKIIQEFLSKVKQMPFTEMS
EENITIKLKQLKAEVIAKNNSFVNEIISRIKVTT
>3THXB 918 XRAY 2.70 0.212 0.273 no DNA mismatch repair protein Msh3 <GB EAW95862> [HOMO SAPIENS]
GPKSANKRSKSIYTPLELQYIEMKQQHKDAVLCVECGYKYRFFGEDAEIAARELNIYCHL
DHNFMTASIPTHRLFVHVRRLVAKGYKVGVVKQTETAALKAIGDNRSSLFSRKLTALYTK
STLIGEDVNPLIKLDDAVNVDEIMTDTSTSYLLCISENKENVRDKKKGNIFIGIVGVQPA
TGEVVFDSFQDSASRSELETRMSSLQPVELLLPSALSEQTEALIHRATSVSVQDDRIRVE
RMDNIYFEYSHAFQAVTEFYAKDTVDIKGSQIISGIVNLEKPVICSLAAIIKYLKEFNLE
KMLSKPENFKQLSSKMEFMTINGTTLRNLEILQNQTDMKTKGSLLWVLDHTKTSFGRRKL
KKWVTQPLLKLREINARLDAVSEVLHSESSVFGQIENHLRKLPDIERGLCSIYHKKCSTQ
EFFLIVKTLYHLKSEFQAIIPAVNSHIQSDLLRTVILEIPELLSPVEHYLKILNEQAAKV

GDKTELFKDLSDFPLIKKRKDEIQGVIDEIRMHLQEIRKILKNPSAQYVTVSGQEFMIEI
KNSAVSCIPTDWVKVGSTKAVSRFHSPFIVENYRHLNQLREQLVLDCSAEWLDFLEKFSE
HYHSLCKAVHHLATVDCIFSLAKVAKQGDYCRPTVQEERKIVIKNGRHPVIDVLLGEQDQ
YVPNNTDLSEDSERVMIITGPNMGGKSSYIKQVALITIMAQIGSYVPAEEATIGIVDGIF
TRMGAADNIYKGRSTFMEELTDTAEIIRKATSQSLVILDELGRGTSTHDGIAIAYATLEY
FIRDVKSLTLFVTHYPPVCELEKNYSHQVGNYHMGFLVSEDESKLDPGAAEQVPDFVTFL
YQITRGIAARSYGLNVAKLADVPGEILKKAAHKSKELEGLINTKRKRLKYFAKLWTMHNA
QDLQKWTEEFNMEETQTS
>4H7AA 168 XRAY 2.60 0.176 0.230 no CRISPR-associated protein Cse2 <UNP CSE2_THET8> [THERMUS THERMOPHILUS]
MSRGHHHHHHGSMSPGERFLDWLKRLQGQKAWTAARAAFRRSLAFPPGAYPRAMPYVEPF
LAKGDWRQEEREAHYLVAALYALKDGDHQVGRTLARALWEKAQGSASVEKRFLALLEADR
DQIAFRLRQAVALVEGGIDFARLLDDLLRWFSPERHVQARWAREYYGA
>4H79A 216 XRAY 1.90 0.150 0.196 no CRISPR-associated protein, Cse2 family <UNP Q47PI5_THEFY> [THERMOBIFIDA FUSCA]
GSMTTTETPKTISLTWVGTFVDQRVREIQEGYRLDNPRAVATLARLRRGAGKEIGDTPDL
WGLILDDRFYADAPPLKEKDMEVAENSAHIALTLYAIHQQSRRDDRMHQRGWGLGEAVRR
LMPSSEIDEPLRKRFVQVGHAVTYKALAQRLREIVTLLRRDAIPLDYGLLADQLYQFRTP
QGAQRVRTAWGRGFHAYRPKTTQNPDSTTTTEKDNS
>1K99A 99 NMR NA NA NA no Upstream binding factor 1 <UNP UBF1_HUMAN> [HOMO SAPIENS]
MKKLKKHPDFPKKPLTPYFRFFMEKRAKYAKLHPEMSNLDLTKILSKKYKELPEKKKMKY
IQDFQREKQEFERNLARFREDHPDLIQNAKKLEHHHHHH
>2HKVA 149 XRAY 1.70 0.185 0.227 no Hypothetical protein <UNP Q41IB9_9BACI> [EXIGUOBACTERIUM SIBIRICUM]
GMTDWQQALDRHVGVGVRTTRDLIRLIQPEDWDKRPISGKRSVYEVAVHLAVLLEADLRI
ATGATADEMAQFYAVPVLPEQLVDRLDQSWQYYQDRLMADFSTETTYWGVTDSTTGWLLE
AAVHLYHHRSQLLDYLNLLGYDIKLDLFE
>1HKQA 132 XRAY 2.75 0.238 0.272 no REPLICATION PROTEIN <UNP Q52546> [PSEUDOMONAS SYRINGAE PV. SAVASTANOI]
MVDNKVTQSNKLIESSHTLTLNEKRLVLCAASLIDSRKPLPKDGYLTIRADTFAEVFGID
VKHAYAALDDAATKLFNRDIRRYVKGKVVERMRWVFHVKYREGQGCVELGFSPTIIPHLT
MLHKEFTSYQLK
>1JKOC 52 XRAY 2.24 0.244 0.306 no DNA-INVERTASE HIN <UNP HIN_SALTY> [SALMONELLA TYPHIMURIUM LT2]
GRPRAINKHEQEQISRLLEKGHPRQQLAIIFGIGVSTLYRYFPASSIKKRMN
>1OY3D 282 XRAY 2.05 0.219 0.247 no transcription factor inhibitor I-kappa-B-beta <UNP IKBB_MOUSE> [MUS MUSCULUS]
VFGYVTEDGDTALHLAVIHQHEPFLDFLLGFSAGHEYLDLQNDLGQTALHLAAILGEAST
VEKLYAAGAGVLVAERGGHTALHLACRVRAHTCACVLLQPRPSHPRDASDTYLTQSQDCT
PDTSHAPAAVDSQPNPENEEEPRDEDWRLQLEAENYDGHTPLHVAVIHKDAEMVRLLRDA
GADLNKPEPTCGRTPLHLAVEAQAASVLELLLKAGADPTARMYGGRTPLGSALLRPNPIL
ARLLRAHGAPEPEDGGDKLSPCSSSGSDSDSDNRDEGDEYDD
>3OD8A 116 XRAY 2.40 0.194 0.241 no Poly [ADP-ribose] polymerase 1 <UNP PARP1_HUMAN> [HOMO SAPIENS]
MGSSHHHHHHSSGLVPRGSHMAESSDKLYRVEYAKSGRASCKKCSESIPKDSLRMAIMVQ
SPMFDGKVPHWYHFSCFWKVGHSIRHPDVEVDGFSELRWDDQQKVKKTAEAGGVTG
>2D8MA 129 NMR NA NA NA no DNA-repair protein XRCC1 <UNP XRCC1_HUMAN> [HOMO SAPIENS]
GSSGSSGEPRRPRAGPEELGKILQGVVVVLSGFQNPFRSELRDKALELGAKYRPDWTRDS
THLICAFANTPKYSQVLGLGGRIVRKEWVLDCHRMRRRLPSQRYLMAGPGSSSEEDEASH

SGGSGPSSG
>1D8BA 81 NMR NA NA NA no SGS1 RECQ HELICASE <UNP SGS1_YEAST> [SACCHAROMYCES CEREVISIAE]
ELNNLRMTYERLRELSLNLGNRMVPPVGNFMPDSILKKMAAILPMNDSAFATLGTVEDKY
RRRFKYFKATIADLSKKRSSE
>2L3NA 104 NMR NA NA NA no DNA-binding protein rap1, Telomere length regulator taz1 <UNP TAZ1_SCHPO> [SCHIZOSACCHAROMYCES POMBE, SYNTHETIC]
SVSILRSSVNHREVDEAIDNILRYTNSTEQQFLEAMESTGGRVRIAIAKLLSKQTSGGSG
GSKLGGSGGSRKDLSVKGMLYDSDSQQILNRLRERVSGSTAQSA
>2L3RA 162 NMR NA NA NA no E3 ubiquitin-protein ligase UHRF1 <UNP UHRF1_HUMAN> [HOMO SAPIENS]
GGMWDETELGLYKVNEYVDARDTNMGAWFEAQVVRVTRKAPSRDEPCSSTSRPALEEDVI
YHVKYDDYPENGVVQMNSRDVRARARTIIKWQDLEVGQVVMLNYNPDNPKERGFWYDAEI
SRKRETRTARELYANVVLGDDSLNDCRIIFVDEVFKIERPGE
>3BU8A 235 XRAY 2.15 0.238 0.256 no Telomeric repeat-binding factor 2 <UNP TERF2_HUMAN> [HOMO SAPIENS]
GAGEARLEEAVNRWVLKFYFHEALRAFRGSRYGDFRQIRDIMQALLVRPLGKEHTVSRLL
RVMQCLSRIEEGENLDCSFDMEAELTPLESAINVLEMIKTEFTLTEAVVESSRKLVKEAA
VIICIKNKEFEKASKILKKHMSKDPTTQKLRNDLLNIIREKNLAHPVIQNFSYETFQQKM
LRFLESHLDDAEPYLLTMAKKALKSESAASSTGKEDKQPAPGPVEKPPREPARQL
>3GZ5A 240 XRAY 2.20 0.223 0.273 no MutT/nudix family protein <UNP Q8EFJ3_SHEON> [SHEWANELLA ONEIDENSIS]
GSHMTEAEYLANYDPKAFKAQLLTVDAVLFTYHDQQLKVLLVQRSNHPFLGLWGLPGGFI
DETCDESLEQTVLRKLAEKTAVVPPYIEQLCTVGNNSRDARGWSVTVCYTALMSYQACQI
QIASVSDVKWWPLADVLQMPLAFDHLQLIEQARERLTQKALYSLVPGFALSEPFTLPELQ
HVHEVLLGKPIQGKSFRRRVEQADLLIDTGLKRTERGRPANLYCLKPDTASYRFLRNLEC
>2FMYA 220 XRAY 2.20 0.209 0.247 no carbon monoxide oxidation system transcription regulator CooA-1
<GB ABB15035> [CARBOXYDOTHERMUS HYDROGENOFORMANS]
ATQMRLTDTNLLEVLNSEEYSGVLKEFREQRYSKKAILYTPNTERNLVFLVKSGRVRVYL
AYEDKEFTLAILEAGDIFCTHTRAFIQAMEDTTILYTDIRNFQNIVVEFPAFSLNMVKVL
GDLLKNSLTIINGLVFKDARLRLAEFLVQAAMDTGLKVPQGIKLELGLNTEEIALMLGTT
RQTVSVLLNDFKKMGILERVNQRTLLLKDLQKLKEFSSGV
>3FMYA 73 XRAY 1.40 0.157 0.182 no HTH-type transcriptional regulator MQSA (ygiT/b3021) <UNP YGIT_ECOLI> [ESCHERICHIA COLI K-12]
GHMASVNAETVAPEFIVKVRKKLSLTQKEASEIFGGGVNAFSRYEKGNAXPHPSTIKLLR
VLDKHPELLNEIR
>4DHXA 75 XRAY 2.10 0.224 0.250 no 80 kDa MCM3-associated protein <UNP MCM3A_HUMAN> [HOMO SAPIENS]
GSLVLSELSQGLAVELMERVMMEFVRETCSQELKNAVETDQRVRVARCCEDVCAHLVDLF
LVEEIFQTAKETLQE
>4DHXB 101 XRAY 2.10 0.224 0.250 no Enhancer of yellow 2 transcription factor homolog <UNP ENY2_HUMAN> [HOMO SAPIENS]
MVVSKMNKDAQMRAAINQKLIETGERERLKELLRAKLIECGWKDQLKAHCKEVIKEKGLE
HVTVDDLVAEITPKGRALVPDSVKKELLQRIRTFLAQHASL
>1UL1X 379 XRAY 2.90 0.220 0.284 no Flap endonuclease-1 <UNP FEN1_HUMAN> [HOMO SAPIENS]
GIQGLAKLIADVAPSAIRENDIKSYFGRKVAIDASMSIYQFLIAVRQGGDVLQNEEGETT
SHLMGMFYRTIRMMENGIKPVYVFDGKPPQLKSGELAKRSERRAEAEKQLQQAQAAGAEQ
EVEKFTKRLVKVTKQHNDECKHLLSLMGIPYLDAPSEAEASCAALVKAGKVYAAATEDMD
CLTFGSPVLMRHLTASEAKKLPIQEFHLSRILQELGLNQEQFVDLCILLGSDYCESIRGI
GPKRAVDLIQKHKSIEEIVRRLDPNKYPVPENWLHKEAHQLFLEPEVLDPESVELKWSEP

NEEELIKFMCGEKQFSEERIRSGVKRLSKSRQGSTQGRLDDFFKVTGSLSSAKRKEPEPK GSTKKKAKTGAAGKFKRGK
>1UL4A 94 NMR NA NA NA no squamosa promoter binding protein-like 4 <UNP SPL4_ARATH> [ARABIDOPSIS THALIANA]
GSSGSSGLRLCQVDRCTADMKEAKLYHRRHKVCEVHAKASSVFLSGLNQRFCQQCSRFHD
LQEFDEAKRSCRRRLAGHNERRRKSSGESGPSSG
>3ULXA 174 XRAY 2.60 0.229 0.276 no Stress-induced transcription factor NAC1 <UNP Q27JE5_ORYSJ> [ORYZA SATIVA SUBSP. JAPONICA]
MGMRRERDAEAELNLPPGFRFHPTDDELVEHYLCRKAAGQRLPVPIIAEVDLYKFDPWDL
PERALFGAREWYFFTPRDRKYPNGSRPNRAAGNGYWKATGADKPVAPRGRTLGIKKALVF
YAGKAPRGVKTDWIMHEYRLADAGRAAAGAKKGSLRLDDWVLCRLYNKKNEWEK
>3ULJA 90 XRAY 1.06 0.121 0.139 no Lin28b, DNA-binding protein <UNP B4F6I0_XENTR> [XENOPUS (SILURANA) TROPICALIS]
GSDPQVLRGSGHCKWFNVRMGFGFISMTSREGSPLENPVDVFVHQSKLYMEGFRSLKEGE
PVEFTFKKSSKGFESLRVTGPGGNPCLGNE
>3F1ZA 133 XRAY 2.46 0.194 0.228 no putative nucleic acid-binding lipoprotein <UNP A6TEE6_KLEP7> [KLEBSIELLA PNEUMONIAE SUBSP. PNEUMONIAE MGH 78578]
GASKAFYSAGDKLFQPGDDAVASMQTYSVAQFLQPFTLNPAKASSDYLGKWVKVRGVIVD
IRRKSGIAGSYYFIVTMRDEQNKTDKRLTFNFGSHNSADVEALSNGSVATIVGQVHQVQD
STIPTLQNPKVVK
>1F1EA 154 XRAY 1.37 0.166 0.206 no HISTONE FOLD PROTEIN <UNP O93641_METKA> [METHANOPYRUS KANDLERI]
MAVELPKAAIERIFRQGIGERRLSQDAKDTIYDFVPTMAEYVANAAKSVLDASGKKTLME
EHLKALADVLMVEGVEDYDGELFGRATVRRILKRAGIERASSDAVDLYNKLICRATEELG
EKAAEYADEDGRKTVQGEDVEKAITYSMPKGGEL
>1IXCA 294 XRAY 2.20 0.219 0.245 no LysR-type regulatory protein <UNP Q9WXC7_ALCEU> [CUPRIAVIDUS NECATOR]
MEFRQLKYFIAVAEAGNMAAAAKRLHVSQPPITRQMQALEADLGVVLLERSHRGIELTAA
GHAFLEDARRILELAGRSGDRSRAAARGDVGELSVAYFGTPIYRSLPLLLRAFLTSTPTA
TVSLTHMTKDEQVEGLLAGTIHVGFSRFFPRHPGIEIVNIAQEDLYLAVHRSQSGKFGKT
CKLADLRAVELTLFPRGGRPSFADEVIGLFKHAGIEPRIARVVEDATAALALTMAGAASS
IVPASVAAIRWPDIAFARIVGTRVKVPISCIFRKEKQPPILARFVEHVRRSAKD
>4IX7A 115 XRAY 1.58 0.191 0.216 no RE55538p <UNP Q8SYK5_DROME> [DROSOPHILA MELANOGASTER]
DNVMVSIGPNNTCVPASVFENINWSVCSLATRKLLVTIFDRETLATHSVTGKPSPAFKDQ
DKPLKRMLDPGKIQDIIFAVTHKCNASEKEVRNAITTKCADENKMMKIQNVKRRS
>2KEBA 101 NMR NA NA NA no DNA polymerase subunit alpha B <UNP DPOA2_HUMAN> [HOMO SAPIENS]
MGSSHHHHHHGSSLEVLFQGPGSMSASAQQLAEELQIFGLDCEEALIEKLVELCVQYGQN
EEGMVGELIAFCTSTHKVGLTSEILNSFEHEFLSKRLSKAR
>1O57A 291 XRAY 2.20 0.100 0.237 no PUR OPERON REPRESSOR <UNP PURR_BACSU> [BACILLUS SUBTILIS]
MKFRRSGRLVDLTNYLLTHPHELIPLTFFSERYESAKSSISEDLTIIKQTFEQQGIGTLL
TVPGAAGGVKYIPKMKQAEAEEFVQTLGQSLANPERILPGGYVYLTDILGKPSVLSKVGK
LFASVFAEREIDVVMTVATKGIPLAYAAASYLNVPVVIVRKDNKVTEGSTVSINYVSGSS
NRIQTMSLAKRSMKTGSNVLIIDDFMKAGGTINGMINLLDEFNANVAGIGVLVEAEGVDE
RLVDEYMSLLTLSTINMKEKSIEIQNGNFLRFFKDNLLKNGETESHHHHHH
>2OZEA 298 XRAY 1.83 0.192 0.218 no Orf delta <UNP Q57280_STRPY> [STREPTOCOCCUS PYOGENES]
MIQYYYTKKEWGVVMEKEELKILEELRRILSNKNEAIVILNNYFKGGVGKSKLSTMFAYL
TDKLNLKVLMIDKDLQATLTKDLAKTFKVELPRVNFYEGLKNGNLASSIVHLTDNLDLIP
GTFDLMLLPKLTRSWTFENESRLLATLLAPLKSDYDLIIIDTVPTPSVYTNNAIVASDYV
MIPLQAEEESTNNIQNYISYLIDLQEQFNPGLDMIGFVPYLVDTDSATIKSNLEELYKQH
KEDNLVFQNIIKRSNKVSTWSKNGITEHKGYDKKVLSMYKNVFFEMLERIIQLENEKE

>4HQBA 148 XRAY 2.30 0.193 0.246 no Single-stranded DNA-binding protein DdrB <UNP DDRB_DEIRA> [DEINOCOCCUS RADIODURANS] DPFTMLQIEFITDLGARVTVNVEHESRLLDVQRHYGRLGWTSGEIPSGGYQFPIENEADF DWSLIGARKWKSPEGEELVIHRGHAYRRRELEAVDSRKLKLPAAIKYSRGAKVSDPQHVR EKADGDIEYVSLAIFRGGKRQERYAVPG
>2BKYA 97 XRAY 1.70 0.172 0.205 no DNA/RNA-BINDING PROTEIN ALBA 1 <UNP ALBA1_SULSO> [SULFOLOBUS SOLFATARICUS] MSSGTPTPSNVVLIGKKPVMNYVLAALTLLNQGVSEIVIKARGRAISKAVDTVEIVRNRF LPDKIEIKEIRVGSQVVTSQDGRQSRVSTIEIAIRKK
>2FC7A 82 NMR NA NA NA no ZZZ3 protein <GB AAH35079> [HOMO SAPIENS] GSSGSSGQQMQAESGFVQHVGFKCDNCGIEPIQGVRWHCQDCPPEMSLDFCDSCSDCLHE TDIHKEDHQLEPIYRSSGPSSG
>1OSVA 230 XRAY 2.50 0.242 0.276 no Bile acid receptor <UNP NR1H4_RAT> [RATTUS NORVEGICUS] AELTVDQQTLLDYIMDSYSKQRMPQEITNKILKEEFSAEENFLILTEMATSHVQILVEFT KRLPGFQTLDHEDQIALLKGSAVEAMFLRSAEIFNKKLPAGHADLLEERIRKSGISDEYI TPMFSFYKSVGELKMTQEEYALLTAIVILSPDRQYIKDREAVEKLQEPLLDVLQKLCKIY QPENPQHFACLLGRLTELRTFNHHHAEMLMSWRVNDHKFTPLLCEIWDVQ
>3RWRD 230 XRAY 3.94 0.274 0.326 no Non-homologous end-joining factor 1 <UNP NHEJ1_HUMAN> [HOMO SAPIENS] MEELEQGLLMQPWAWLQLAENSLLAKVFITKQGYALLVSDLQQVWHEQVDTSVVSQRAKE LNKRLTAPPAAFLCHLDNLLRPLLKDAAHPSEATFSCDCVADALILRVRSELSGLPFYWN FHCMLASPSLVSQHLIRPLMGMSLALQCQVRELATLLHMKDLEIQDYQESGATLIRDRLK TEPFEENSFLEQFMIEKLPEACSIGDGKPFVMNLQDLYMAVTTQHHHHHH
>4IBYA 200 XRAY 1.45 0.171 0.200 no Cellular tumor antigen p53 <UNP P53_HUMAN> [HOMO SAPIENS] SSSVPSQKTYQGSYGFRLGFLHSGTAKSVTCTYSPALNKMFCQLAKTCPVQLWVDSTPPP GTRVRAMAIYKQSQHMTEVVRRCPHHERCSDSDGLAPPQHLIRVEGNLRVEYLDDRNTFR HSVVVPYEPPEVGSDCTTIHYNYMCNRSCMGGMNRRPILTIITLEDSSGNLLGRNSFEVH VCACPGRDRRTEEENLRKKG
>3TEDA 271 XRAY 2.00 0.195 0.249 no Chromo domain-containing protein 1 <UNP CHD1_YEAST> [SACCHAROMYCES CEREVISIAE] GPDMDSIGESEVRALYKAILKFGNLKEILDELIADGTLPVKSFEKYGETYDEMMEAAKDC VHEEEKNRKEILEKLEKHATAYRAKLKSGEIKAENQPKDNPLTRLSLKKREKKAVLFNFK GVKSLNAESLLSRVEDLKYLKNLINSNYKDDPLKFSLGNNTPKPVQNWSSNWTKEEDEKL LIGVFKYGYGSWTQIRDDPFLGITDKIFLNEVHNPVAKKSASSSDTTPTPSKKGKGITGS SKKVPGAIHLGRRVDYLLSFLRGGLNTKSPS
>3TEKA 148 XRAY 2.00 0.207 0.255 no ThermoDBP-single stranded DNA binding protein <PDB 3TEK> [THERMOPROTEUS TENAX] MGEELREEERGEVRSELITKGEKKLVLIRWNTGKTSAGRLFGRYGPGGRPEFFKLLFGAV AGSLREQFGPDGENIFNRIRDSEKFRETSRELFDGLKKWFFEEAVPRYNLERGDIFMIST ELVLDPDTGELLWNRDKTQLIYWIRSDR
>2BA3A 51 NMR NA NA NA no NikA <GB BAA78022> [PLASMID R64] SDSAVRKKSEVRQKTVVRTLRFSPVEDETIRKKAEDSGLTVSAYIRNAALN
>3EWAA 322 XRAY 2.00 0.223 0.255 no DNA repair and recombination protein radA <UNP RADA_METMP> [METHANOCOCCUS MARIPALUDIS] MADVLTELPGVGPSTADKLIEGGYLDFMKIATATIGELTDIEGISEKAAAKMIMAARDLC DLGFKSGVELLKQRQSVWRLSTGSTELDTVLAGGIESQSVTEFAGMFGSGKTQIMHQTCV NLQMREKIFADLEGVVEEELEAPKAVYIDTEGTFRPERVVQMAEGAGIDGQTVLDNTFVA RAYNSDMQMLFAEKIEDLIKGGNNIKLVIIDSLTSTFRNEFTGRGKLAERQQKLGRHMAT LNKLADLYNCIVLVTNQVAAKPDAYFGVAEQAIGGHVVGHAATFRFFLRKSKGDKRVAKL YDSPHLPDSEAVFRITEKGIQD
>2H6BA 250 XRAY 2.20 0.186 0.229 no ChloroPhenol Reduction gene K <UNP Q18R04_DESHD> [DESULFITOBACTERIUM HAFNIENSE] MSVEGLGKDFCGAIIPDNFFPIEKLRNYTQMGLIRDFAKGSAVIMPGEEITSMIFLVEGK

IKLDIIFEDGSEKLLYYAGGNSLIGKLYPTGNNIYATAMEPTRTCWFSEKSLRTVFRTDE
DMIFEIFKNYLTKVAYYARQVAEMNTYNPTIRILRLFYELCSSQGKRVGDTYEITMPLSQ
KSIGEITGVHHVTVSRVLACLKRENILDKKKNKIIVYNLGELKHLSEQTSYYSDPNSSSV
DKLAAALDHH
>3B0DC 76 XRAY 2.20 0.208 0.261 no Centromere protein W <PDB 3B0D> [GALLUS GALLUS]
GRRTVPRGTLRKIIKKHKPHLRLAANTDLLVHLSFLLFLHRLAEEARTNAFENKCKIIKP
EHTIAAAKVILKKSRG
>3NFIA 237 XRAY 1.90 0.190 0.224 no DNA-directed RNA polymerase I subunit RPA49 <UNP RPA49_YEAST>
[SACCHAROMYCES CEREVISIAE]
GSHMDLPTRAQMDEITSNDRPTPLANIDATDVEQIYPIESIIPKKELQFIRVSSILKEAD
KEKKLELFPYQNNSKYVAKKLDSLTQPSQMTKLQMLYYLSLLLGVYENRRVNNKTKLLER
LNSPPEILVDGILSRFTVIKPGQFGRSKDRSYFIDPQNEDKILCYILAIIMHLDNFIVEI
TPLAHELNLKPSKVVSLFRVLGAIVKGATVAQAEAFGIPKSTAASYKIATMKVPFKL
>1Z4HA 66 NMR NA NA NA no Tor inhibition protein <UNP Q9AZ38_BPHK6> [ESCHERICHIA
COLI] MQHELQPDSLVDLKFIMADTGFGKTFIYDRIKSGDLPKAKVIHGRARWLYRDHCEFKNKL
LSRANG
>2Z4SA 440 XRAY 3.00 0.291 0.291 no Chromosomal replication initiator protein dnaA <UNP DNAA_THEMA> [THERMOTOGA MARITIMA]
MKERILQEIKTRVNRKSWELWFSSFDVKSIEGNKVVFSVGNLFIKEWLEKKYYSVLSKAV
KVVLGNDATFEITYEAFEPHSSYSEPLVKKRAVLLTPLNPDYTFENFVVGPGNSFAYHAA
LEVAKHPGRYNPLFIYGGVGLGKTHLLQSIGNYVVQNEPDLRVMYITSEKFLNDLVDSMK
EGKLNEFREKYRKKVDILLIDDVQFLIGKTGVQTELFHTFNELHDSGKQIVICSDREPQK
LSEFQDRLVSRFQMGLVAKLEPPDEETRKSIARKMLEIEHGELPEEVLNFVAENVDDNLR
RLRGAIIKLLVYKETTGKEVDLKEAILLLKDFIKPNRVKAMDPIDELIEIVAKVTGVPRE
EILSNSRNVKALTARRIGMYVAKNYLKSSLRTIAEKFNRSHPVVVDSVKKVKDSLLKGNK
QLKALIDEVIGEISRRALSG
>3EI3B 383 XRAY 2.30 0.210 0.251 no DNA damage-binding protein 2 <UNP Q2YDS1_DANRE> [DANIO RERIO]
MHHHHHHVDENLYFQGGGRTGGQKKVGQTSILHYIYKSSLGQSIHAQLRQCLQEPFIRSL
KSYKLHRTASPFDRRVTSLEWHPTHPTTVAVGSKGGDIILWDYDVQNKTSFIQGMGPGDA
ITGMKFNQFNTNQLFVSSIRGATTLRDFSGSVIQVFAKTDSWDYWYCCVDVSVSRQMLAT
GDSTGRLLLLGLDGHEIFKEKLHKAKVTHAEFNPRCDWLMATSSVDATVKLWDLRNIKDK
NSYIAEMPHEKPVNAAYFNPTDSTKLLTTDQRNEIRVYSSYDWSKPDQIIIHPHRQFQHL
TPIKATWHPMYDLIVAGRYPDDQLLLNDKRTIDIYDANSGGLVHQLRDPNAAGIISLNKF
SPTGDVLASGMGFNILIWNREDT
>1EIJA 80 NMR NA NA NA no HYPOTHETICAL PROTEIN MTH1615 <UNP DNBP_METTH>
[METHANOTHERMOBACTER THERMAUTOTROPHICUS]
MRQQLEMQKKQIMMQILTPEARSRLANLRLTRPDFVEQIELQLIQLAQMGRVRSKITDEQ
LKELLKRVAGKKREIKISRK
>2HUEA 175 XRAY 1.70 0.209 0.239 no Anti-silencing protein 1 <UNP ASF1_YEAST> [SACCHAROMYCES CEREVISIAE]
PLGSPNSSIVSLLGIKVLNNPAKFTDPYEFEITFECLESLKHDLEWKLTYVGSSRSLDHD
QELDSILVGPVPVGVNKFVFSADPPSAELIPASELVSVTVILLSCSYDGREFVRVGYYVN
NEYDEEELRENPPAKVQVDHIVRNILAEKPRVTRFNIVWDNENEGDLYPPEQPGV
>2HUEB 77 XRAY 1.70 0.209 0.239 no Histone H3 <UNP Q92133_XENLA> [XENOPUS LAEVIS]
MALIRKLPFQRLVREIAQDFKTDLRFQSSAVMALQEASEAYLVALFEDTNLCAIHAKRVT
IMPKDIQLARRIRGERA
>2HUEC 84 XRAY 1.70 0.209 0.239 no Histone H4 <UNP H4_XENLA> [XENOPUS LAEVIS]

MKVLRDNIQGITKPAIRRLARRGGVKRISGLIYEETRGVLKVFLENVIRDAVTYTEHAKR
KTVTAMDVVYALKRQGRTLYGFGG
>3SSCA 170 XRAY 2.10 0.197 0.259 no 5-methylcytosine-specific restriction enzyme B <UNP MCRB_ECOLI> [ESCHERICHIA COLI]
MESIQPWIEKFIKQAQQQRSQSTKDYPTSYRNLRVKLSFGYGNFTSIPWFAFLGEGQEAS
NGIYPVILYYKDFDELVLAYGISDTNEPHAQWQFSSDIPKTIAEYFQATSGVYPKKYGQS
YYACSQKVSQGIDYTRFASMLDNIINDYKLIFNSGKSVIPPLEGHHHHHH
>3GXQA 54 XRAY 2.35 0.237 0.261 no Putative regulator of transfer genes ArtA <UNP Q2FDC9_STAA3> [STAPHYLOCOCCUS AUREUS SUBSP. AUREUS USA300]
ENSVFFGKKKKVSLHLLVDPDMKDEIIKYAQEKDFDNVSQAGREILKKGLEQIA
>3TOCA 224 XRAY 2.20 0.217 0.258 no Putative uncharacterized protein <UNP Q99ZV9_STRP1> [STREPTOCOCCUS PYOGENES]
GSFTMNLNFSLLDEPIPLRGGTILVLEDVCVFSKIVQYCYQYEEDSELKFFDHKMKTIKE
SEIMLVTDILGFDVNSSTILKLIHADLESQFNEKPEVKSMIDKLVATITELIVFECLENE
LDLEYDEITILELIKSLGVKVETQSDTIFEKCLEILQIFKYLTKKKLLIFVNSGAFLTKD
EVASLQEYISLTNLTVLFLEPRELYDFPQYILDEDYFLITKNMV
>2IJGX 526 XRAY 2.10 0.238 0.262 no Cryptochrome DASH, chloroplast/mitochondrial <UNP CRYD_ARATH> [ARABIDOPSIS THALIANA]
MNDHIHRVPALTEEEIDSVAIKTFERYALPSSSSVKRKGKGVTILWFRNDLRVLDNDALY
KAWSSSDTILPVYCLDPRLFHTTHFFNFPKTGALRGGFLMECLVDLRKNLMKRGLNLLIR
SGKPEEILPSLAKDFGARTVFAHKETCSEEVDVERLVNQGLKRVGNSTKLELIWGSTMYH
KDDLPFDVFDLPDVYTQFRKSVEAKCSIRSSTRIPLSLGPTPSVDDWGDVPTLEKLGVEP
QEVTRGMRFVGGESAGVGRVFEYFWKKDLLKVYKETRNGMLGPDYSTKFSPWLAFGCISP
RFIYEEVQRYEKERVANNSTYWVLFELIWRDYFRFLSIKCGNSLFHLGGPRNVQGKWSQD
QKLFESWRDAKTGYPLIDANMKELSTTGFMSNRGRQIVCSFLVRDMGLDWRMGAEWFETC
LLDYDPCSNYGNWTYGAGVGNDPREDRYFSIPKQAQNYDPEGEYVAFWLQQLRRLPKEKR
HWPGRLMYMDTVVPLKHGNGPMAGGSKSGGGFRGSHSGRRSRHNGP
>4JQFA 179 XRAY 1.60 0.199 0.219 no CST complex subunit STN1 <UNP STN1_HUMAN> [HOMO SAPIENS]
AEALSNPGALDLPSLTSLLSEKAKEFLMENRVQSFYQQELEMVESLLSLANQPVIHSASS
DQVNFKKDTTSKAIHSIFKNAIQLLQEKGLVFQKDDGFDNLYYVTREDKDLHRKIHRIIQ
QDCQKPNHMEKGCHFLHILACARLSIRPGLSEAVLQQVLELLEDQSDIVSTMEHYYTAF
>3QOEA 302 XRAY 3.00 0.183 0.247 no Heterocyst differentiation protein <UNP Q2ACK9_9CYAN> [FISCHERELLA THERMALIS]
SNAMSNDVDLIKRLGPSAMDQIMLYLAFSAMRTSGHRHGAFLDAAATAAKCAIYMTYLEQ
GQNLRMTGHLHHLEPKRVKAIVEEVRQALTEGKLLKMLGSQEPRYLIQFPYVWMEKYPWR
PGRSRIPGTSLTSEEKRQIEQKLPSNLPDAHLITSFEFLELIEFLHKRSQEDLPKEHQMP
LSEALAEHIKRRLLYSGTVTRIDSPWGMPFYALTRPFYAPADDQERTYIMVEDTARFFRM
MRDWAEKRPNTMRVLEELDILPEKMQQAKDELDEIIRAWADKYHQDDGVPVVLQMVFGKK
ED
>3QRFF 82 XRAY 2.80 0.248 0.283 no Forkhead box protein P3 <UNP FOXP3_HUMAN> [HOMO SAPIENS]
MRPPFTYATLIRWAILEAPEKQRTLNEIYHWFTRMFAFFRNHPATWKNAIRHNLSLHKCF
VRVESEKGAVWTVDELEFRKKR
>2ECCA 76 NMR NA NA NA no Homeobox and leucine zipper protein Homez <UNP HOMEZ_HUMAN> [HOMO SAPIENS]
GSSGSSGKRKTKEQLAILKSFFLQCQWARREDYQKLEQITGLPRPEIIQWFGDTRYALKH
GQLKWFRDNASGPSSG
>3M03A 95 XRAY 2.50 0.228 0.266 no Origin recognition complex subunit 6 <UNP ORC6_HUMAN> [HOMO SAPIENS]

MSNIGIRDLAVQFSCIEAVNMASKILKSYESSLPQTQQVDLDLSRPLFTSAALLSACKIL
KLKVDKNKMVATSGVKKAIFDRLCKQLEKIGQQVD
>1UB4A 110 XRAY 1.70 0.251 0.232 no MazF protein <UNP CHPA_ECOLI> [ESCHERICHIA COLI]
VSRYVPDMGDLIWVDFDPTKGSEQAGHRPAVVLSPFMYNNKTGMCLCVPCTTQSKGYPFE
VVLSGQERDGVALADQVKSIAWRARGATKKGTVAPEELQLIKAKINVLIG
>1UB4C 85 XRAY 1.70 0.251 0.232 no MazE protein <UNP CHPR_ECOLI> [ESCHERICHIA COLI]
GPHMIHSSVKRWGNSPAVRIPATLMQALNLNIDDEVKIDLVDGKLIIEPVRKEPVFTLAE
LVNDITPENLHENIDWGEPKDKEVW
>1MSZA 86 NMR NA NA NA no DNA-binding protein SMUBP-2 <UNP SMBP2_HUMAN> [HOMO SAPIENS]
MGSLNGGSPEGVESQDGVDHFRAMIVEFMASKKMQLEFPPSLNSHDRLRVHQIAEEHGLR
HDSSGEGKRRFITVSKRAGSHHHHHH
>4JOLA 64 XRAY 2.91 0.211 0.252 no Protein CBFA2T1 <UNP MTG8_HUMAN> [HOMO SAPIENS]
SEEMIDHRLTDREWAEEWKHLDHLLNCIMDMVEKTRRSLTVLRRCQEADREELNYWIRRY
SDAE
>4JOIA 166 XRAY 2.05 0.213 0.249 no CST complex subunit STN1 <UNP STN1_HUMAN> [HOMO SAPIENS]
LDPVFLAFAKLYIRDILDMKESRQVPGVFLYNGHPIKQVDVLGTVIGVRERDAFYSYGVD
DSTGVINCICWKKLNTESVSAAPSAARELSLTSQLKKLQETIEQKTKIEIGDTIRVRGSI
RTYREEREIHATTYYKVDDPVWNIQIARMLELPTIYRKVYDQPFHS
>4JOIC 122 XRAY 2.05 0.213 0.249 no CST complex subunit TEN1 <UNP TEN1L_HUMAN> [HOMO SAPIENS]
MLPKPGTYYLPWEVSAGQVPDGSTLRTFGRLCLYDMIQSRVTLMAQHGSDQHQVLVCTKL
VEPFHAQVGSLYIVLGELQHQQDRGSVVKARVLTCVEGMNLPLLEQAIREQRLYKQERGG
SQ
>2G99A 308 XRAY 1.90 0.212 0.243 no WD-repeat protein 5 <UNP WDR5_HUMAN> [HOMO SAPIENS]
KPTPVKPNYALKFTLAGHTKAVSSVKFSPNGEWLASSSADKLIKIWGAYDGKFEKTISGH
KLGISDVAWSSDSNLLVSASDDKTLKIWDVSSGKCLKTLKGHSNYVFCCNFNPQSNLIVS
GSFDESVRIWDVKTGKCLKTLPAHSDPVSAVHFNRDGSLIVSSSYDGLCRIWDTASGQCL
KTLIDDDNPPVSFVKFSPNGKYILAATLDNTLKLWDYSKGKCLKTYTGHKNEKYCIFANF
SVTGGKWIVSGSEDNLVYIWNLQTKEIVQKLQGHTDVVISTACHPTENIIASAALENDKT
IKLWKSDC
>2G9WA 138 XRAY 1.80 0.204 0.252 no conserved hypothetical protein <UNP P95163_MYCTU>
[MYCOBACTERIUM TUBERCULOSIS]
MAKLTRLGDLERAVMDHLWSRTEPQTVRQVHEALSARRDLAYTTVMAVLQRLAKKNLVLQ
IRDDRAHRYAPVHGRDELVAGLMVDALAQAEDSGSRQAALVHFVERVGADEADALRRALA
ELEAGHGNRPPAGAATET
>3D6WA 111 XRAY 2.40 0.184 0.209 no Methyl-accepting/DNA response regulator <UNP Q73A38_BACC1>
[BACILLUS CEREUS]
GKSVVTLKTTDGWIPVPFSKVMYLEAKDKKTYVNAEELTGTHKYSLQEFEYLLPKDSFIR
CHRSFIVNVNHIKAIYPDTHSTFLLSMDNGERVPVSQSYASYFRKLLGFGS
>1WG6A 127 NMR NA NA NA no HYPOTHETICAL PROTEIN (RIKEN cDNA 2810455B10) <GB XP_110852> [MUS
MUSCULUS]
GSSGSSGLKGEPDCYALSLESSEQLTLEIPLNDSGSAGLGVSLKGNKSRETGTDLGIFIK
SIIHGGAAFKDGRLRMNDQLIAVNGETLLGKSNHEAMETLRRSMSMEGNIRGMIQLVILR
RSGPSSG
>1WG2A 64 NMR NA NA NA no zinc finger (AN1-like) family protein <UNP ZF2N3_ARATH> [ARABIDOPSIS
THALIANA]
GSSGSSGPSRPVRPNNRCFSCNKKVGVMGFKCKCGSTFCGSHRYPEKHECSFDFKEVGSG
PSSG
>2ZTDA 212 XRAY 2.40 0.204 0.268 no Holliday junction ATP-dependent DNA helicase ruvA <UNP
RUVA_MYCTU> [MYCOBACTERIUM TUBERCULOSIS]

MASMTGGQQMGRGSEFMIASVRGEVLEVALDHVVIEAAGVGYRVNATPATLATLRQGTEA
RLITAMIVREDSMTLYGFPDGETRDLFLTLLSVSGVGPRLAMAALAVHDAPALRQVLADG
NVAALTRVPGIGKRGAERMVLELRDKVGVAATGGALSTNGHAVRSPVVEALVGLGFAAKQ
AEEATDTVLAANHDATTSSALRSALSLLGKAR
>1ZS3A 182 XRAY 2.70 0.209 0.258 no Lactococcus lactis MG1363 DpsA <PDB 1ZS3> [LACTOCOCCUS LACTIS]
MITKLMIDEKYAKELDKAEIDHHKPTAGAMLGHVLSNLFIENIRLTQAGIYAKSPVKCEY
LREIAQREVEYFFKISDLLLDENEIVPSTTEEFLKYHKFITEDPKAKYWTDEDLLESFIV
DFQAQNMFITRAIKLANKEEKFALAAGVVELYGYNLQVIRNLAGDLGKSVADFHDEDEDN
DN
>2Q79A 93 XRAY 1.80 0.227 0.241 no Regulatory protein E2 <UNP VE2_HPV16> [HUMAN PAPILLOMAVIRUS TYPE 16]
TTPIVHLKGDANTLKCLRYRFKKHCTLYTAVSSTWHWTGHNVKHKSAIVTLTYDSEWQRD
QFLSQVKIPKTITVSTGFMSIGGGTGGGSGGGS
>1EE8A 266 XRAY 1.90 0.214 0.258 no MUTM (FPG) PROTEIN <UNP FPG_THET8> [THERMUS THERMOPHILUS]
PELPEVETTRRRLRPLVLGQTLRQVVHRDPARYRNTALAEGRRILEVDRRGKFLLFALEG
GVELVAHLGMTGGFRLEPTPHTRAALVLEGRTLYFHDPRRFGRLFGVRRGDYREIPLLLR
LGPEPLSEAFAFPGFFRGLKESARPLKALLLDQRLAAGVGNIYADEALFRARLSPFRPAR
SLTEEEARRLYRALREVLAEAVELGGSTLSDQSYRQPDGLPGGFQTRHAVYGREGLPCPA
CGRPVERRVVAGRGTHFCPTCQGEGP
>2OWYA 306 XRAY 2.50 0.234 0.279 no Recombination-associated protein rdgC <UNP RDGC_PSEAE> [PSEUDOMONAS AERUGINOSA]
MWFRNLLVYRLTQDLQLDADSLEKALGEKSARPCASQELTTYGFTAPFGKGPDAPLVHVS
QDFFLISARKEERILPGSVVRDALKEKVDEIEAQQMRKVYKKERDQLKDEIVQTLLPRAF
IRRSSTFAAIAPSLGLILVDSASAKKAEDLLSTLREALGSLPVRPLSVKVAPTATLTDWV
KTQEAAGDFHVLDECELRDTHEDGGVVRCKRQDLTSEEIQLHLTAGKLVTQLSLAWSDKL
SFVLDDKLAVKRLRFEDLLQEQAEKDGGEDALGQLDASFTLMMLTFAEFLPALFEALGGE
EIPQGV
>1T0FC 54 XRAY 1.85 0.211 0.235 no Transposon Tn7 transposition protein tnsC <UNP TNSC_ECOLI> [ESCHERICHIA COLI]
GSAIKVVKPSDWDSLPDTDLRYIYSQRQPEKTMHERLKGKGVIVDMASLFKQAG
>1WOCA 103 XRAY 2.00 0.221 0.276 no Primosomal replication protein n <UNP PRIB_ECOLI> [ESCHERICHIA COLI]
TNRLVLSGTVCRAPLRKVSPSGIPHCQFVLEHRSVQEEAGFHRQAWCQMPVIVSGHENQA
ITHSITVGSRITVQGFISCHKAKNGLSKMVLHAEQIELIDSGD
>2COBA 70 NMR NA NA NA no LCoR protein <UNP Q5VW16_HUMAN> [HOMO SAPIENS]
GSSGSSGRGRYRQYNSEILEEAISVVMSGKMSVSKAQSIYGIPHSTLEYKVKERLGTLKN
PPKKKMKLMR
>2PI2E 142 XRAY 2.00 0.233 0.255 no Replication protein A 14 kDa subunit <UNP RFA3_HUMAN> [HOMO SAPIENS]
MGHHHHHHHHHHSSGHIEGRHMVDMMDLPRSRINAGMLAQFIDKPVCFVGRLEKIHPTGK
MFILSDGEGKNGTIELMEPLDEEISGIVEVVGRVTAKATILCTSYVQFKEDSHPFDLGLY
NEAVKIIHDFPQFYPLGIVQHD

>1YD0A 96 XRAY 1.50 0.170 0.185 no UvrABC system protein C <UNP UVRC_THEMA> [THERMOTOGA MARITIMA]
MKEKIRKKILLAPEEPGVYIFKNKGVPIYIGKAKRLSNRLRSYLNPQTEKVFRIGEEADE
LETIVVMNEREAFILEANLIKKYRPKYNVRLKDTDF
>1YDXA 406 XRAY 2.30 0.199 0.231 no type I restriction enzyme specificity protein MG438 <UNP T1SX_MYCGE> [MYCOPLASMA GENITALIUM]
MGHHHHHHHHHHSSGHIDDDDKHMTPKLKLNNNINWTKRTIDSLFDLKKGEMLEKELITP
EGKYEYFNGGVKNSGRTDKFNTFKNTISVIVGGSCGYVRLADKNFFCGQSNCTLNLLDPL
ELDLKFAYYALKSQQERIEALAFGTTIQNIRISDLKELEIPFTSNKNEQHAIANTLSVFD
ERLENLASLIEINRKLRDEYAHKLFSLDEAFLSHWKLEALQSQMHEITLGEIFNFKSGKY
LKSEERLEEGKFPYYGAGIDNTGFVAEPNTEKDTISIISNGYSLGNIRYHEIPWFNGTGS
IALEPMNNEIYVPFFYCALKYLQKDIKERMKSDDSPFLSLKLAGEIKVPYVKSFQLQRKA
GKIVFLLDQKLDQYKKELSSLTVIRDTLLKKLFPDMTERTKSIKDY
>3TRBA 104 XRAY 2.00 0.201 0.243 no Virulence-associated protein I <UNP Q83BL4_COXBU> [COXIELLA BURNETII]
SNAMAANRMRPIHPGEILAEELGFLDKMSANQLAKHLAIPTNRVTAILNGARSITADTAL
RLAKFFGTTPEFWLNLQDAYDIKMALKKSGKKIEKEVTPYDQAA
>1WPKA 146 NMR NA NA NA no ADA regulatory protein <UNP ADA_ECOLI> [ESCHERICHIA COLI]
MKKATCLTDDQRWQSVLARDPNADGEFVFAVRTTGIFCRPSCRARHALRENVSFYANASE
ALAAGFRPCKRCQPEKANAQQHRLDKITHACRLLEQETPVTLEALADQVAMSPFHLHRLF KATTGMTPKAWQQAWRARRLRESLAK
>2WP0A 180 XRAY 2.67 0.228 0.266 no HOBA <UNP O25828_HELPY> [HELICOBACTER PYLORI]
MKNFYDWIKEFIRDQGEFIAQQSGWLELERSSYAKLIAQTISHVLNGGSLLVSADSSRHW
FLNYILSNLNPKDLKERPLLSVIDFNASSFYPKNDANLSLATIEMTYQNPMFWHVGKIEN
EGLKTILLSKIPSFLWLFEELKEDCLLLKEHDSLLDYKLLQLFKLFENALFSVLYNKVTL
>4IOOA 127 XRAY 1.25 0.150 0.167 no Bromodomain-containing protein 4 <UNP BRD4_HUMAN> [HOMO SAPIENS]
SMNPPPPETSNPNKPKRQTNQLQYLLRVVLKTLWKHQFAWPFQQPVDAVKLNLPDYYKII
KTPMDMGTIKKRLENNYYWNAQECIQDFNTMFTNCYIYNKPGDDIVLMAEALEKLFLQKI NELPTEE
>3IO5A 333 XRAY 2.40 0.212 0.248 no Recombination and repair protein <UNP UVSX_BPT4> [ENTEROBACTERIA PHAGE T4]
GSHMDVVRTKIPMMNIALSGEITGGMQSGLLILAGPSKSFKSNFGLTMVSSYMRQYPDAV
CLFYDSEFGITPAYLRSMGVDPERVIHTPVQSLEQLRIDMVNQLDAIERGEKVVVFIDSL
GNLASKKETEDALNEKVVSDMTRAKTMKSLFRIVTPYFSTKNIPCIAINHTYETQEMFSK
TVMGGGTGPMYSADTVFIIGKRQIKDGSDLQGYQFVLNVEKSRTVKEKSKFFIDVKFDGG
IDPYSGLLDMALELGFVVKPKNGWYAREFLDEETGEMIREEKSWRAKDTNCTTFWGPLFK
HQPFRDAIKRAYQLGAIDSNEIVEAEVDELINS
>3PGZA 193 XRAY 2.10 0.212 0.256 no Single-stranded DNA-binding protein <UNP Q6G302_BARHE> [BARTONELLA HENSELAE] MAHHHHHHMGTLEAQTQGPGSMAGSLNKVILIGNLGADPEIRRLNSGDQVANLRIATSES
WRDRNTNERKERTEWHNIVIFNENLVKVVEQYLKKGSKIYIEGQLQTRKWQDQNGNDRYT
TEIVLQKYRGELQMLDGRAAAGGEQMQGANQSSGAYSSVGFGDNSANQRDVFGSNNSQLG
ESFSHKLDDDVPF
>3ZLJC 53 XRAY 3.10 0.229 0.263 no DNA MISMATCH REPAIR PROTEIN MUTS <UNP MUTS_ECOLI> [ESCHERICHIA COLI K-12]
PNAAATQVDGTQMSLLSVPEETSPAVEALENLDPRSLTPRQALEWIYRLKSLV
>1ZRJA 50 NMR NA NA NA no E1B-55kDa-associated protein 5 isoform c <UNP HNRL1_HUMAN> [HOMO SAPIENS] GSSGSSGMDVRRLKVNELREELQRRGLDTRGLKAELAERLQAALSGPSSG
>3POVA 488 XRAY 2.50 0.209 0.264 no ORF 37 <UNP P88925_HHV8> [HUMAN HERPESVIRUS 8 TYPE M]
GAMEATPTPADLFSEDYLVDTLDGLTVDDQQAVLASLSFSKFLKHAKVRDWCAQAKIQPS
MPALRMAYNYFLFSKVGEFIGSEDVCNFFVDRVFGGVRLLDVASVYAACSQMNAHQRHHI

CCLVERATSSQSLNPVWDALRDGIISSSKFHWAVKQQNTSKKIFSPWPITNNHFVAGPLA
FGLRCEEVVKTLLATLLHPDETNCLDYGFMQSPQNGIFGVSLDFAANVKTDTEGRLQFDP
NCKVYEIKCRFKYTFAKMECDPIYAAYQRLYEAPGKLALKDFFYSISKPAVEYVGLGKLP
SESDYLVAYDQEWEACPRKKRKLTPLHNLIRECILHNSTTESDVYVLTDPQDTRGQISIK
ARFKANLFVNVRHSYFYQVLLQSSIVEEYIGLDSGIPRLGSPKYYIATGFFRKRGYQDPV
NCTIGGDALDPHVEIPTLLIVTPVYFPRGAKHRLLHQAANFWSRSAKDTFPYIKWDFSYL
SANVPHSP
>3GA8A 78 XRAY 1.70 0.166 0.186 no HTH-type transcriptional regulator MqsA (YgiT/B3021) <UNP YGIT_ECOLI> [ESCHERICHIA COLI K-12]
GHMKCPVCHQGEMVSGIKDIPYTFRGRKTVLKGIHGLYCVHCEESIMNKEESDAFMAQVK
AFRASVNAETVAPEFIVK
>3RLOA 204 XRAY 1.80 0.169 0.192 no Gamma-interferon-inducible protein 16 <UNP IF16_HUMAN> [HOMO SAPIENS]
GSVDDSAQSDLKEVMVLNATESFVYEPKEQKKMFHATVATENEVFRVKVFNIDLKEKFTP
KKIIAIANYVCRNGFLEVYPFTLVADVNADRNMEIPKGLIRSASVTPKINQLCSQTKGSF
VNGVFEVHKKNVRGEFTYYEIQDNTGKMEVVVHGRLTTINCEEGDKLKLTCFELAPKSGN
TGELRSVIHSHIKVIKTRKNAAAS
>1Z91A 147 XRAY 2.50 0.223 0.305 no Organic hydroperoxide resistance transcriptional regulator <UNP OHRR_BACSU> [BACILLUS SUBTILIS] MENKFDHMKLENQLSFLLYASSREMTKQYKPLLDKLNITYPQYLALLLLWEHETLTVKKM
GEQLYLDSGTLTPMLKRMEQQGLITRKRSEEDERSVLISLTEDGALLKEKAVDIPGTILG LSKQSGEDLKQLKSALYTLLETLHQKN
>3TB6A 298 XRAY 2.21 0.188 0.231 no Arabinose metabolism transcriptional repressor <UNP ARAR_BACSU> [BACILLUS SUBTILIS]
GIDPFTSAKSALHSNKTIGVLTTYISDYIFPSIIRGIESYLSEQGYSMLLTSTNNNPDNE
RRGLENLLSQHIDGLIVEPTKSALQTPNIGYYLNLEKNGIPFAMINASYAELAAPSFTLD
DVKGGMMAAEHLLSLGHTHMMGIFKADDTQGVKRMNGFIQAHRERELFPSPDMIVTFTTE
EKESKLLEKVKATLEKNSKHMPTAILCYNDEIALKVIDMLREMDLKVPEDMSIVGYDDSH
FAQISEVKLTSVKHPKSVLGKAAAKYVIDCLEHKKPKQEDVIFEPELIIRQSARKLNE
>2O99A 182 XRAY 1.70 0.207 0.261 no Acetate operon repressor <UNP ICLR_ECOLI> [ESCHERICHIA COLI]
GHMSRNLLAIVHPILRNLMEESGETVNMAVLDQSDHEAIIIDQVQCTHLMRMSAPIGGKL
PMHASGAGKAFLAQLSEEQVTKLLHRKGLHAYTHATLVSPVHLKEDLAQTRKRGYSFDDE
EHALGLRCLAACIFDEHREPFAAISISGPISRITDDRVTEFGAMVIKAAKEVTLAYGGMR
GS
>2G7OA 70 XRAY 1.40 0.195 0.224 no Protein traM <UNP TRAM1_ECOLI> [ESCHERICHIA COLI]
ESAFNQTEFNKLLLECVVKTQSSVAKILGIESLSPHVSGNSKFEYANMVEDIREKVSSEM
ERFFPKNDDE
>3CNBA 143 XRAY 2.00 0.208 0.256 no DNA-binding response regulator, merR family <UNP Q47UF8_COLP3> [COLWELLIA PSYCHRERYTHRAEA] MSLNVKNDFSILIIEDDKEFADMLTQFLENLFPYAKIKIAYNPFDAGDLLHTVKPDVVML
DLMMVGMDGFSICHRIKSTPATANIIVIAMTGALTDDNVSRIVALGAETCFGKPLNFTLL
EKTIKQLVEQKKATSEGHHHHHH
>1KGSA 225 XRAY 1.50 0.181 0.210 no DNA BINDING RESPONSE REGULATOR D <UNP Q9WYN0_THEMA> [THERMOTOGA MARITIMA]
MNVRVLVVEDERDLADLITEALKKEMFTVDVCYDGEEGMYMALNEPFDVVILDIMLPVHD
GWEILKSMRESGVNTPVLMLTALSDVEYRVKGLNMGADDYLPKPFDLRELIARVRALIRR
KSESKSTKLVCGDLILDTATKKAYRGSKEIDLTKKEYQILEYLVMNKNRVVTKEELQEHL
WSFDDEVFSDVLRSHIKNLRKKVDKGFKKKIIHTVRGIGYVARDE
>3RYPA 210 XRAY 1.60 0.209 0.234 no Catabolite gene activator <UNP CRP_ECOLI> [ESCHERICHIA COLI]
MVLGKPQTDPTLEWFLSHCHIHKYPSKSTLIHQGEKAETLYYIVKGSVAVLIKDEEGKEM

ILSYLNQGDFIGELGLFEEGQERSAWVRAKTACEVAEISYKKFRQLIQVNPDILMRLSAQ
MARRLQVTSEKVGNLAFLDVTGRIAQTLLNLAKQPDAMTHPDGMQIKITRQEIGQIVGCS RETVGRILKMLEDQNLISAHGKTIVVYGTR
>3DSHA 246 XRAY 2.00 0.206 0.242 no Interferon regulatory factor 5 <UNP IRF5_HUMAN> [HOMO SAPIENS]
EQLLPDLLISPHMLPLTDLEIKFQYRGRPPRALTISNPHGCRLFYSQLEATQEQVELFGP
ISLEQVRFPSPEDIPSDKQRFYTNQLLDVLDRGLILQLQGQDLYAIRLCQCKVFWSGPCA
SAHDSCPNPIQREVKTKLFSLEHFLNELILFQKGQTNTPPPFEIFFCFGEEWPDRKPREK
KLITVQVVPVAARLLLEMFSGELSWSADDIRLQISNPDLKDRMVEQFKELHHIWQSQQRL
QPVAQA
>4BHXA 94 XRAY 1.95 0.174 0.224 no PATERNALLY-EXPRESSED GENE 3 PROTEIN <UNP PEG3_HUMAN>
[HOMO SAPIENS]
GHMTDSEFFHQRFRNLIYVEFVGPRKTLIKLRNLCLDWLQPETRTKEEIIELLVLEQYLT
IIPEKLKPWVRAKKPENCEKLVTLLENYKEMYQP
>2GPEA 52 XRAY 1.90 0.205 0.249 no Bifunctional protein putA <UNP PUTA_ECOLI> [ESCHERICHIA COLI]
MGTTTMGVKLDDATRERIKSAATRIDRTPHWLIKQAIFSYLEQLENSDTLPE
>1XD7A 145 XRAY 2.30 0.260 0.285 no ywnA <UNP P71036_BACSU> [BACILLUS SUBTILIS SUBSP. SUBTILIS]
MSLINSRLAVAIHILSLISMDEKTSSEIIADSVNTNPVVVRRMISLLKKADILTSRAGVP
GASLKKDPADISLLEVYRAVQKQEELFAVHENPNPKCPVGKKIQNALDETFESVQRAMEN
ELASKSLKDVMNHLFEGGSHHHHHH
>3R4KA 260 XRAY 2.46 0.192 0.231 no Transcriptional regulator, IclR family <UNP
Q1GKY2_SILST> [RUEGERIA SP. TM1040]
GMGTVSKALTLLTYFNHGRLEIGLSDLTRLSGMNKATVYRLMSELQEAGFVEQVEGARSY
RLGPQVLRLAALREASVPILSASRRVLRELSEDTGETTHLSLLQGEQLASLSHAYSSRNA
TKVMMEDAEVLTFHGTASGLAVLAYSEPSFVDAVLAAPLTARTPQTQTDPAAIRAEIAEV
RRTGLAQSIGGFEAEVHSHAVPIFGPDRAVLGALAVAAPTSRMTPDQKRTIPPALRAAGL SLTERIGGACPPEFPTDIAA
>3AL2A 235 XRAY 2.00 0.172 0.204 no DNA topoisomerase 2-binding protein 1 <UNP TOPB1_HUMAN> [HOMO
SAPIENS]
GPLGSLKKQYIFQLSSLNPQERIDYCHLIEKLGGLVIEKQCFDPTCTHIVVGHPLRNEKY
LASVAAGKWVLHRSYLEACRTAGHFVQEEDYEWGSSSILDVLTGINVQQRRLALAAMRWR
KKIQQRQESGIVEGAFSGWKVILHVDQSREAGFKRLLQSGGAKVLPGHSVPLFKEATHLF
SDLNKLKPDDSGVNIAEAAAQNVYCLRTEYIADYLMQESPPHVENYCLPEAISFI
>2ALCA 65 NMR NA NA NA no ETHANOL REGULON TRANSCRIPTIONAL ACTIVATOR <UNP ALCR_EMENI> [EMERICELLA
NIDULANS]
GSMADTRRRQNHSCDPCRKGKRRCDAPENRNEANENGWVSCSNCKRWNKDCTFNWLSSQR
SKNSS
>1XNAA 183 NMR NA NA NA no DNA-REPAIR PROTEIN XRCC1 <UNP XRCC1_HUMAN> [HOMO SAPIENS]
MPEIRLRHVVSCSSQDSTHCAENLLKADTYRKWRAAKAGEKTISVVLQLEKEEQIHSVDI
GNDGSAFVEVLVGSSAGGAGEQDYEVLLVTSSFMSPSESRSGSNPNRVRMFGPDKLVRAA
AEKRWDRVKIVCSQPYSKDSPFGLSFVRFHSPPDKDEAEAPSQKVTVTKLGQFRVKEEEE
SAN
>3U61B 324 XRAY 3.20 0.244 0.305 no DNA polymerase accessory protein 44 <UNP DPA44_BPT4>
[ENTEROBACTERIA PHAGE T4]
GPGGSMITVNEKEHILEQKYRPSTIDECILPAFDKETFKSITSKGKIPHIILHSPSPGTG
KTTVAKALCHDVNADMMFVNGSDCKIDFVRGPLTNFASAASFDGRQKVIVIDEFDRSGLA
ESQRHLRSFMEAYSSNCSIIITANNIDGIIKPLQSRCRVITFGQPTDEDKIEMMKQMIRR
LTEICKHEGIAIADMKVVAALVKKNFPDFRKTIGELDSYSSKGVLDAGILSLVTNDRGAI
DDVLESLKNKDVKQLRALAPKYAADYSWFVGKLAEEIYSRVTPQSIIRMYEIVGENNQYH
GIAANTELHLAYLFIQLACEMQWK
>3U61F 228 XRAY 3.20 0.244 0.305 no DNA polymerase processivity component <UNP DPA5_BPT4>

[ENTEROBACTERIA PHAGE T4]
MKLSKDTTALLKNFATINSGIMLKSGQFIMTRAVNGTTYAEANISDVIDFDVAIYDLNGF
LGILSLVNDDAEISQSEDGNIKIADARSTIFWPAADPSTVVAPNKPIPFPVASAVTEIKA
EDLQQLLRVSRGLQIDTIAITVKEGKIVINGFNKVEDSALTRVKYSLTLGDYDGENTFNF
IINMANMKMQPGNYKLLLWAKGKQGAAKFEGEHANYVVALEADSTHDF
>3N4PA 279 XRAY 2.15 0.222 0.272 no Terminase subunit UL89 protein <UNP TERL_HCMVA> [HUMAN HERPESVIRUS 5]
MGHHHHHHDYDIPTTENLYFQGGGTNKISQNTVLITDQSREEFDILRYSTLNTNAYDYFG
KTLYVYLDPAFTTNRKASGTGVAAVGAYRHQFLIYGLEHFFLRDLSESSEVAIAECAAHM
IISVLSLHPYLDELRIAVEGNTNQAAAVRIACLIRQSVQSSTLIRVLFYHTPDQNHIEQP
FYLMGRDKALAVEQFISRFNSGYIKASQELVSYTIKLSHDPIEYLLEQIQNLHRVTLAEG
TTARYSAKRQNRISDDLIIAVIMATYLCDDIHAIRFRVS
>4LRVA 109 XRAY 2.50 0.203 0.242 no DNA sulfur modification protein DndE <UNP B3HI59_ECOLX> [ESCHERICHIA COLI]
SMLPNRMALSRQTEDQLKKLKGYTGITPNIAARLAFFRSVESEFRYSPERDSKKLDGTLV
LDKITWLGETLQATELVLKMLYPQLEQKALIKAWAAHVEDGIAALRNHK
>2P5KA 64 XRAY 1.00 0.139 0.160 no Arginine repressor <UNP ARGR_BACSU> [BACILLUS SUBTILIS]
MNKGQRHIKIREIITSNEIETQDELVDMLKQDGYKVTQATVSRDIKELHLVKVPTNNGSY
KYSL
>1AKHB 83 XRAY 2.50 0.201 0.302 no MATING-TYPE PROTEIN ALPHA-2 <UNP MTAL2_YEAST> [SACCHAROMYCES CEREVISIAE] TKPYRGHRFTKENVRILESWFAKNIENPYLDTKGLENLMKNTSLSRIQIKNWVSNRRRKE
KTITIAPELADLLSGEPLAKKKE
>3P83A 245 XRAY 3.05 0.188 0.216 no DNA polymerase sliding clamp <UNP PCNA_ARCFU> [ARCHAEOGLOBUS FULGIDUS]
MIDVIMTGELLKTVTRAIVALVSEARIHFLEKGLHSRAVDPANVAMVIVDIPKDSFEVYN
IDEEKTIGVDMDRIFDISKSISTKDLVELIVEDESTLKVKFGSVEYKVALIDPSAIRKEP
RIPELELPAKIVMDAGEFKKAIAAADKISDQVIFRSDKEGFRIEAKGDVDSIVFHMTETE
LIEFNGGEARSMFSVDYLKEFCKVAGSGDLLTIHLGTNYPVRLVFELVGGRAKVEYILAP
RIESE
>3P83D 217 XRAY 3.05 0.188 0.216 no Ribonuclease HII <UNP RNH2_ARCFU> [ARCHAEOGLOBUS FULGIDUS]
GPLGSPEFPGRLMKAGIDEAGKGCVIGPLVVAGVACSDEDRLRKLGVKDSKKLSQGRREE
LAEEIRKICRTEVLKVSPENLDERMAAKTINEILKECYAEIILRLKPEIAYVDSPDVIPE
RLSRELEEITGLRVVAEHKADEKYPLVAAASIIAKVEREREIERLKEKFGDFGSGYASDP
RTREVLKEWIASGRIPSCVRMRWKTVSNLRQKTLDDF
>2D7LA 81 NMR NA NA NA no WD repeat and HMG-box DNA binding protein 1 <UNP WDHD1_HUMAN> [HOMO SAPIENS]
GSSGSSGRPKTGFQMWLEENRSNILSDNPDFSDEADIIKEGMIRFRVLSTEERKVWANKA
KGETASEGTEAKKRKSGPSSG
>1S6MA 293 XRAY 2.28 0.210 0.245 no TrwC <UNP Q47673_ECOLI> [ESCHERICHIA COLI]
MLSHMVLTRQDIGRAASYYEDGADDYYAKDGDASEWQGKGAEELGLSGEVDSKRFRELLA
GNIGEGHRIMRSATRQDSKERIGLDLTFSAPKSVSLQALVAGDAEIIKAHDRAVARTLEQ
AEARAQARQKIQGKTRIETTGNLVIGKFRHETSRERDPQLHTHAVILNMTKRSDGQWRAL
KNDEIVKATRYLGAVYNAELAHELQKLGYQLRYGKDGNFDLAHIDRQQIEGFSKRTEQIA
EWYAARGLDPNSVSLEQKQAAKVLSRAKKTSVDREALRAEWQATAKELGIDFS
>3BQOA 211 XRAY 2.00 0.220 0.237 no Telomeric repeat-binding factor 1 <UNP TERF1_HUMAN> [HOMO SAPIENS]
EEEEEDAGLVAEAEAVAAGWMLDFLCLSLCRAFRDGRSEDFRRTRNSAEAIIHGLSSLTA
CQLRTIYICQFLTRIAAGKTLDAQFENDERITPLESALMIWGSIEKEHDKLHEEIQNLIK

IQAIAVCMENGNFKEAEEVFERIFGDPNSHMPFKSKLLMIISQKDTFHSFFQHFSYNHMM EKIKSYVNYVLSEKSSTFLMKAAAKVVESKR
>3VIBA 210 XRAY 2.40 0.212 0.256 no MtrR <UNP C0ITL7_NEIGO> [NEISSERIA GONORRHOEAE]
MRKTKTEALKTKEHLMLAALETFYRKGIARTSLNEIAQAAGVTRDALYWHFKNKEDLFDA
LFQRICDDIENCIAQDAADAEGGSWTVFRHTLLHFFERLQSNDIHYKFHNILFLKCEHTE
QNAAVIAIARKHQAIWREKITAVLTEAVENQDLADDLDKETAVIFIKSTLDGLIWRWFSS GESFDLGKTAPRIIGIMMDNLENHPCLRRK
>3V4GA 180 XRAY 1.60 0.182 0.198 no Arginine repressor <UNP ARGR_VIBVY> [VIBRIO VULNIFICUS]
MHHHHHHSSGVDLGTENLYFQSNAMRPSEKQDNLVRAFKALLKEERFGSQGEIVEALKQE
GFENINQSKVSRMLTKFGAVRTRNAKMEMVYCLPTELGVPTVSSSLRELVLDVDHNQALV
VIHTGPGAAQLIARMLDSLGKSEGILGVVAGDDTIFITPTLTITTEQLFKSVCELFEYAG
>1JHGA 101 XRAY 1.30 0.127 0.172 no TRP OPERON REPRESSOR <UNP TRPR_ECOLI> [ESCHERICHIA COLI]
SAAMAEQRHQEWLRFVDLLKNAYQNDLHLPLLNLMLTPDEREALGTRVRIIEELLRGEMS
QRELKNELGAGIATITRGSNSLKAAPVELRQWLEEVLLKSD
>2LJ6A 177 NMR NA NA NA no Probable ATP-dependent DNA ligase <UNP Q9I1X7_PSEAE> [PSEUDOMONAS AERUGINOSA]
MPSSKPLAEYARKRDFRQTPEPSGRKPRKDSTGLLRYCVQKHDASRLHYDFRLELDGTLK
SWAVPKGPCLDPAVKRLAVQVEDHPLDYADFEGSIPQGHYGAGDVIVWDRGAWTPLDDPR
EGLEKGHLSFALDGEKLSGRWHLIRTNLRGKQSQWFLVKAKDGEARSLDRFDVLKER
>3MAJA 382 XRAY 2.05 0.187 0.232 no DNA processing chain A <UNP Q6N563_RHOPA> [RHODOPSEUDOMONAS PALUSTRIS]
GHMDVGERSSDQGTTVLTEAQRIDWMRLIRAENVGPRTFRSLINHFGSARAALERLPELA
RRGGAARAGRIPSEDEARREIEAGRRIGVELVAPGETGYPTRLATIDDAPPLLGVHALPE
ALAVMARPMIAIVGSRNASGAGLKFAGQLAADLGAAGFVVISGLARGIDQAAHRASLSSG
TVAVLAGGHDKIYPAEHEDLLLDIIQTRGAAISEMPLGHVPRGKDFPRRNRLISGASVGV
AVIEAAYRSGSLITARRAADQGREVFAVPGSPLDPRAAGTNDLIKQGATLITSASDIVEA
VASILERPIELPGREPEHAPPEGEPDTGDRTRILALLGPSPVGIDDLIRLSGISPAVVRT ILLELELAGRLERHGGSLVSLS
>2MA1A 75 NMR NA NA NA no DNA helicase RecQ <UNP Q9RUU2_DEIRA> [DEINOCOCCUS RADIODURANS]
HDAPLFEALRAWRLQKAKELSLPPYTIFHDATLKTIAELRPGSHATLGTVSGVGGRKLAA
YGDEVLQVVRDSSGG
>2WKDA 119 XRAY 2.10 0.240 0.256 no ORF34P2 <UNP Q09WL7_9CAUD> [LACTOCOCCUS PHAGE P2]
GTIITVTAQANEKNTRTVSTAKGDKKIISVPLFEKEKGSNVKVAYGSAFMPDFIQMGDTV
TVSGRVQAKESGEYVNYNFVFPTVEKVFITNDNSSQSQAKQDLFGGSEPIEVNSEDLPF
>1MP9A 198 XRAY 2.00 0.209 0.254 no TATA-binding protein <UNP TBP_SULAC> [SULFOLOBUS ACIDOCALDARIUS] YIIPDEIPYKAVVNIENIVATVTLDQTLDLYAMERSVPNVEYDPDQFPGLIFRLESPKIT
SLIFKSGKMVVTGAKSTDELIKAVKRIIKTLKKYGMQLTGKPKIQIQNIVASANLHVIVN
LDKAAFLLENNMYEPEQFPGLIYRMDEPRVVLLIFSSGKMVITGAKREDEVHKAVKKIFD
KLVELDCVKPVEEEELEF
>3EQXA 373 XRAY 1.60 0.170 0.197 no FIC DOMAIN CONTAINING TRANSCRIPTIONAL REGULATOR <UNP Q8E9K5_SHEON> [SHEWANELLA ONEIDENSIS]
GMEWQAEQAYNHLPPLPLDSKLAELAETLPILKACIPARAALAELKQAGELLPNQGLLIN
LLPLLEAQGSSEIENIVTTTDKLFQYAQEDSQADPMTKEALRYRTALYQCFTQLSNRPLC
VTTALEICSTIKSVQMDVRKVPGTSLTNQATGEVIYTPPAGESVIRDLLSNWEAFLHNQD
DVDPLIKMAMAHYQFEAIHPFIDGNGRTGRVLNILYLIDQQLLSAPILYLSRYIVAHKQD
YYRLLLNVTTQQEWQPWIIFILNAVEQTAKWTTHKIAAARELIAHTTEYVRQQLPKIYSH
ELVQVIFEQPYCRIQNLVESGLAKRQTASVYLKQLCDIGVLEEVQSGKEKLFVHPKFVTL
MTKDSNQFSRYAL
>1VJFA 180 XRAY 1.62 0.146 0.167 no DNA-binding protein, putative <UNP Q9ABV9_CAUCR> [CAULOBACTER CRESCENTUS CB15]

MGSDKIHHHHHHMKTRADLFAFFDAHGVDHKTLDHPPVFRVEEGLEIKAAMPGGHTKNLF
LKDAKGQLWLISALGETTIDLKKLHHVIGSGRLSFGPQEMMLETLGVTPGSVTAFGLIND
TEKRVRFVLDKALADSDPVNFHPLKNDATTAVSQAGLRRFLAALGVEPMIVDFAAMEVVG
>2EBIA 86 NMR NA NA NA no DNA binding protein GT-1 <UNP Q9FX53_ARATH> [ARABIDOPSIS THALIANA]
KKRAETWVQDETRSLIMFRRGMDGLFNTSKSNKHLWEQISSKMREKGFDRSPDMCTDKWR
NLLKEFKKAKHHDRGNGSAKMSYYKE
>3JRHA 98 XRAY 2.88 0.224 0.253 no DNA-binding protein fis <UNP FIS_ECOLI> [ESCHERICHIA COLI]
MFEQRVNSDVLTVSTVNSQDQVTQKPLRDSVKQALKNYFAQLNGQDVNDLYELVLAEVEQ
PLLDMVMQYTRGNQTRAALMMGINRGTLRKKLKKYGMN
>2JR1A 79 NMR NA NA NA no Virulence regulator <UNP Q9PFC9_XYLFA> [XYLELLA FASTIDIOSA]
FNVKQKSEITALVKEVTPPRKAPSKAKREAPIKYWLPHSGATWSGRGKIPKPFEAWIGTA
AYTAWKAKHPDEKFPAFPG
>4G4KA 103 XRAY 1.52 0.181 0.209 no Accessory gene regulator protein A <UNP AGRA_STAAU>
[STAPHYLOCOCCUS AUREUS]
MDNSVETIELKRGSNSVYVQYDDIMFFESSTKSHRLIAHLDNRQIEFYGNLKELSQLDDR
FFRCHNSFVVNRHNIESIDSKERIVYFKNKEHCYASVRNVKKI
>3027A 68 XRAY 2.80 0.252 0.289 no Putative uncharacterized protein <UNP Q9P9J8_SULIS> [SULFOLOBUS
ISLANDICUS]
MRPGIRKLVVLNPRAYKGGSGHTTFYLLIPKDIAEALDIKPDDTFILNMEQKDGDIVLSY
KRVKELKI
>2KJ8A 118 NMR NA NA NA no Putative prophage CPS-53 integrase <UNP INTS_ECOLI> [ESCHERICHIA COLIK-12]
SSNNNSFSAIYKEWYEHKKQVWSVGYATELAKMFDDDILPIIGGLEIQDIEPMQLLEVIR
RFEDRGAMERANKARRRCGEVFRYAIVTGRAKYNPAPDLADAMKGYRKKNLEHHHHHH
>3KJPA 299 XRAY 1.80 0.211 0.234 no Protection of telomeres protein 1 <UNP POTE1_HUMAN> [HOMO SAPIENS]
MSLVPATNYIYTPLNQLKGGTIVNVYGVVKFFKPPYLSKGTDYCSVVTIVDQTNVKLTCL
LFSGNYEALPIIYKNGDIVRFHRLKIQVYKKETQGITSSGFASLTFEGTLGAPIIPRTSS
KYFNFTTEDHKMVEALRVWASTHMSPSWTLLKLCDVQPMQYFDLTCQLLGKAEVDGASFL
LKVWDGTRTPFPSWRVLIQDLVLEGDLSHIHRLQNLTIDILVYDNHVHVARSLKVGSFLR
IYSLHTKLQSMNSENQTMLSLEFHLHGGTSYGRGIRVLPESNSDVDQLKKDLESANLTA
>4NJYA 163 XRAY 1.32 0.139 0.164 no Histidine triad nucleotide-binding protein 2, mitochondrial
<UNPHINT2_HUMAN> [HOMO SAPIENS]
MAAAVVLAAGLRAARRAVAATGVRGGQVRGAAGVTDGNEVAKAQQATPGGAAPTIFSRIL
DKSLPADILYEDQQCLVFRDVAPQAPVHFLVIPKKPIPRISQAEEEDQQLLGHLLLVAKQ
TAKAEGLGDGYRLVINDGKLGAQSVYHLHIHVLGGRQLQWPPG
>3UFDA 82 XRAY 2.80 0.207 0.237 no Regulatory protein <UNP Q8GGH0_9ENTR> [ENTEROBACTER SP.
RFL1396] GSHMESFLLSKVSFVIKKIRLEKGMTQEDLAYKSNLDRTYISGIERNSRNLTIKSLELIM
KGLEVSDVVFFEMLIKEILKHD
>3MKWB 138 XRAY 2.99 0.234 0.268 no Protein sopB <UNP SOPB_ECOLI> [ESCHERICHIA COLI]
MGSSHHHHHHSSGLVPRGSHYRPTSAYERGQRYASRLQNEFAGNISALADAENISRKIIT
RCINTAKLPKSVVALFSHPGELSARSGDALQKAFTDKEELLKQQASNLHEQKKAGVIFEA
DEVITLLTSVLKTSSASR
>3FPNB 106 XRAY 1.80 0.231 0.248 no Geobacillus stearothermophilus UvrB interaction domain <PDB 3FPN>
[GEOBACILLUS STEAROTHERMOPHILUS] GSHMGSPEEYRELVVSLRVGMEIERNALLRRLVDIQYDRNDIDFRRGTFRVRGDVVEIFP
ASRDEHCIRVEFFGDEIERIREVDALTGEVLGEREHVAIFPASHFV
>2FPHX 165 XRAY 1.70 0.243 0.299 no ylmH <GB AAL00310> [STREPTOCOCCUS PNEUMONIAE]
GIYQHFSIEDRPFLDKGMEWIKKVEDSYAPFLTPFINPHQEKLLKILAKTYGLACSSSGE
FVSSEYVRVLLYPDYFQPEFSDFEISLQEIVYSNKFEYLTHAKILGTVINQLGIERKLFG
DILVDEERAQIMINQQFLLLFQDGLKKIGRIPVSLEERPFTEKID

>3O4QA 150 XRAY 1.55 0.166 0.211 no integrase <UNP Q4ZJZ6_RSVSR> [ROUS SARCOMA VIRUS]
GSGRGLGPLQIWQTDFTLEPRMAPRSWLAVTVDTASSAIVVTQHGRVTSVAAQHHWATAI
AVLGRPKAIKTDNGSCFTSKSTREWLARWGIAHTTGIPGNSQGQAMVERANRLLKDKIRV LAEGDGFMKRIPTSKQGELLAKAMYALNHF
>3LX2A 259 XRAY 2.40 0.246 0.305 no DNA polymerase sliding clamp 2 <UNP PCNA2_PYRKO> [THERMOCOCCUS KODAKARENSIS] MTFEIVFDSAREFESLIATLEKFFDEAVFQVNMEGIQMRAIDPSRVVLVDLNLPEMLFSK
YSVESEEAIAFDLKRFLKVLKLARSRDTLVLRKGGENFLEVGLLGDENTWFKLPLIDANT
PEIEIPSLPWTVKAVVLAGALKRAVKAAKLVSDSIYFMATPEKLTFKAEGNDSEVRTVLT
MEDPGLLDLEHKMTKAKSAYGVAYLEDILRSLADADEVIIRFGFDIPLLLKYMVRDAGEV SFLIAPRVEEGRSHHHHHH
>3KHKA 544 XRAY 2.55 0.214 0.242 no Type I restriction-modification system methylation subunit <UNP Q8PZR3_METMA> [METHANOSARCINA MAZEI] MSLDIEQQFLNDLDNQLWRAADKLRSNLDAANYKHVVLGLIFLKYVSDAFEERQQELTEL
FQKDDDDNIYYLPREDYDSDEAYQQAIAEELEIGDYYTEKNVFWVPKTARWNKLRDVITL
PTGSVIWQDEQGEDVKLRSVSWLIDNAFDDIEKANPKLKGILNRISQYQLDADKLIGLIN
EFSLTSFNNPEYNGEKLNLKSKDILGHVYEYFLGQFALAEGKQGGQYYTPKSIVTLIVEM
LEPYKGRVYDPAMGSGGFFVSSDKFIEKHANVKHYNASEQKKQISVYGQESNPTTWKLAA
MNMVIRGIDFNFGKKNADSFLDDQHPDLRADFVMTNPPFNMKDWWHEKLADDPRWTINTN
GEKRILTPPTGNANFAWMLHMLYHLAPTGSMALLLANGSMSSNTNNEGEIRKTLVEQDLV
ECMVALPGQLFTNTQIPACIWFLTKDKNAKNGKRDRRGQVLFIDARKLGYMKDRVLRDFK
DEDIQKLADTFHNWQQEWSEENNQAGFCFSADLALIRKNDFVLTPGRYVGAEAEEDEGHH
HHHH
>2M14A 119 NMR NA NA NA no RNA polymerase II transcription factor B subunit 1 <UNP TFB1_YEAST> [SACCHAROMYCES CEREVISIAE] PSHSGAAIFEKVSGIIAINEDVSPAELTWRSTDGDKVHTVVLSTIDKLQATPASSEKMML
RLIGKVDESKKRKDNEGNEVVPKPQRHMFSFNNRTVMDNIKMTLQQIISRYKDADGNSS
>2BNZA 53 XRAY 2.60 0.227 0.258 no ORF OMEGA <UNP Q57468_STRPY> [STREPTOCOCCUS PYOGENES]
MAKKDIMGDKTVRVRADLHHIIKIETAKNGGNVKEVMDQALEEYIRKYLPDKL
>2Z3XA 63 XRAY 2.10 0.257 0.283 no Small, acid-soluble spore protein C <UNP SSPC_BACSU> [BACILLUS SUBTILIS]
AKLLIPQAASAIEQMKLEIASEFGVQLGAETTSRANGSVGGEITKRLVRLAQQNMGGQFH
GQQ
>2JX3A 131 NMR NA NA NA no Protein DEK <UNP DEK_HUMAN> [HOMO APIENS]
FTIAQGKGQKLCEIERIHFFLSKKKTDELRNLHKLLYNRPGTVSSLKKNVGQFSGFPFEK
GSVQYKKKEEMLKKFRNAMLKSICEVLDLERSGVNSELVKRILNFLMHPKPSGKPLPKSK KTCSKGSKKER
>2NTIA 249 XRAY 2.50 0.211 0.251 no DNA polymerase sliding clamp B <UNP PCNA2_SULSO>
[SULFOLOBUS SOLFATARICUS]
MVKIVYPNAKDFFSFINSITNVTDSIILNFTEDGIFSRHLTEDKVLMAIMRIPKDVLSEY
SIDSPTSVKLDVSSVKKILSKASSKKATIELTETDSGLKIIIRDEKSGAKSTIYIKAEKG
QVEQLTEPKVNLAVNFTTDESVLNVIAADVTLVGEEMRISTEEDKIKIEAGEEGKRYVAF
LMKDKPLKELSIDTSASSSYSAEMFKDAVKGLRGFSAPTMVSFGENLPMKIDVEAVSGGH
MIFWIAPRL
>3RH2A 212 XRAY 2.42 0.207 0.250 no Hypothetical TetR-like transcriptional
regulator
<UNP A1S1Q5_SHEAM> [SHEWANELLA AMAZONENSIS]
GMKTRDKIIQASLELFNEHGERTITTNHIAAHLDISPGNLYYHFRNKEDIIRCIFDQYEQ
HLLLGFKPYADQKVDLELLMSYFDAMFYTMWQFRFMYANLADILARDDTLKARYLKVQQA
VLEQSIAVLNQLKKDGILQIEDERIADLADTIKMIIGFWISYKLTQSSIATISKASLYEG
LLRVLMIFKAYSTPDSLANFDRLEQHFRSQSN
>2HQLA 110 XRAY 2.00 0.210 0.272 no Hypothetical protein MG376 homolog <UNP
Y554_MYCPN> [MYCOPLASMA PNEUMONIAE]
GGGGGGMLNRVFLEGEIESSCWSVKKTGFLVTIKQMRFFGERLFTDYYVIYANGQLAYEL

EKHTKKYKTISIEGILRTYLERKSEIWKTTIEIVKIFNPKNEIVIDYKEI
>1KNUA 274 XRAY 2.50 0.224 0.264 no PEROXISOME PROLIFERATOR ACTIVATED RECEPTOR GAMMA
<UNP PPAT_HUMAN> [HOMO SAPIENS]
LNPESADLRALAKHLYDSYIKSFPLTKAKARAILTGKTTDKSPFVIYDMNSLMMGEDKIK
FKHITPLQEQSKEVAIRIFQGCQFRSVEAVQEITEYAKSIPGFVNLDLNDQVTLLKYGVH
EIIYTMLASLMNKDGVLISEGQGFMTREFLKSLRKPFGDFMEPKFEFAVKFNALELDDSD
LAIFIAVIILSGDRPGLLNVKPIEDIQDNLLQALELQLKLNHPESSQLFAKLLQKMTDLR
QIVTEHVQLLQVIKKTETDMSLHPLLQEIYKDLY
>1UFIA 64 XRAY 1.65 0.236 0.309 no Major centromere autoantigen B <UNP CENPB_HUMAN> [HOMO SAPIENS]
GSHMPVPSFGEAMAYFAMVKRYLTSFPIDDRVQSHILHLEHDLVHVTRKNHARQAGVRGL
GHQS
>3Q8DA 242 XRAY 2.30 0.197 0.234 no DNA repair protein recO <UNP D5CXR1_ECOKI> [ESCHERICHIA COLI]
MEGWQRAFVLHSRPWSETSLMLDVFTEESGRVRLVAKGARSKRSTLKGALQPFTPLLLRF
GGRGEVKTLRSAEAVSLALPLSGITLYSGLYINELLSRVLEYETRFSELFFDYLHCIQSL
AGVTGTPEPALRRFELALLGHLGYGVNFTHCAGSGEPVDDTMTYRYREEKGFIASVVIDN
KTFTGRQLKALNAREFPDADTLRAAKRFTRMALKPYLGGKPLKSRELFRQFMPKRTVKTH
YE
>2DIGA 68 NMR NA NA NA no Lamin-B receptor <UNP LBR_HUMAN> [HOMO SAPIENS]
GSSGSSGMPSRKFADGEVVRGRWPGSSLYYEVEILSHDSTSQLYTVKYKDGTELELKEND
IKSGPSSG

>3PC6A 104 XRAY 1.90 0.182 0.227 no DNA repair protein XRCC1 <UNP XRCC1_MOUSE> [MUS MUSCULUS]
MPELPDFFEGKHFFLYGEFPGDERRRLIRYVTAFNGELEDYMNERVQFVITAQEWDPNFE
EALMENPSLAFVRPRWIYSCNEKQKLLPHQLYGVVPQAHHHHHH
>3W3WA 1078 XRAY 2.20 0.217 0.256 no Importin subunit beta-3 <UNP IMB3_YEAST> [SACCHAROMYCES CEREVISIAE]
MSALPEEVNRTLLQIVQAFASPDNQIRSVAEKALSEEWITENNIEYLLTFLAEQAAFSQD
TTVAALSAVLFRKLALKAPITHIRKEVLAQIRSSLLKGFLSERADSIRHKLSDAIAECVQ
DDLPAWPELLQALIESLKSGNPNFRESSFRILTTVPYLITAVDINSILPIFQSGFTDASD
NVKIAAVTAFVGYFKQLPKSEWSKLGILLPSLLNSLPRFLDDGKDDALASVFESLIELVE
LAPKLFKDMFDQIIQFTDMVIKNKDLEPPARTTALELLTVFSENAPQMCKSNQNYGQTLV
MVTLIMMTEVSIDDDDAAEWIESDDTDDEEEVTYDHARQALDRVALKLGGEYLAAPLFQY
LQQMITSTEWRERFAAMMALSSAAEGCADVLIGEIPKILDMVIPLINDPHPRVQYGCCNV
LGQISTDFSPFIQRTAHDRILPALISKLTSECTSRVQTHAAAALVNFSEFASKDILEPYL
DSLLTNLLVLLQSNKLYVQEQALTTIAFIAEAAKNKFIKYYDTLMPLLLNVLKVNNKDNS
VLKGKCMECATLIGFAVGKEKFHEHSQELISILVALQNSDIDEDDALRSYLEQSWSRICR
ILGDDFVPLLPIVIPPLLITAKATQDVGLIEEEEAANFQQYPDWDVVQVQGKHIAIHTSV
LDDKVSAMELLQSYATLLRGQFAVYVKEVMEEIALPSLDFYLHDGVRAAGATLIPILLSC
LLAATGTQNEELVLLWHKASSKLIGGLMSEPMPEITQVYHNSLVNGIKVMGDNCLSEDQL
AAFTKGVSANLTDTYERMQDRHGDGDEYNENIDEEEDFTDEDLLDEINKSIAAVLKTTNG
HYLKNLENIWPMINTFLLDNEPILVIFALVVIGDLIQYGGEQTASMKNAFIPKVTECLIS
PDARIRQAASYIIGVCAQYAPSTYADVCIPTLDTLVQIVDFPGSKLEENRSSTENASAAI
AKILYAYNSNIPNVDTYTANWFKTLPTITDKEAASFNYQFLSQLIENNSPIVCAQSNISA

VVDSVIQALNERSLTEREGQTVISSVKKLLGFLPSSDAMAIFNRYPADIMEKVHKWFA
>1R5KA 261 XRAY 2.70 0.208 0.236 no Estrogen receptor <UNP ESR1_HUMAN> [HOMO SAPIENS] MDPMIKRSKKNSLALSLTADQMVSALLDAEPPILYSEYDPTRPFSEASMMGLLTNLADRE
LVHMINWAKRVPGFVDLTLHDQVHLLECAWLEILMIGLVWRSMEHPGKLLFAPNLLLDRN
QGKCVEGMVEIFDMLLATSSRFRMMNLQGEEFVCLKSIILLNSGVYTFLSSTLKSLEEKD
HIHRVLDKITDTLIHLMAKAGLTLQQQHQRLAQLLLILSHIRHMSNKGMEHLYSMKCKNV
VPLYDLLLEMLDAHRLHAPTS
>1U9NA 236 XRAY 2.30 0.208 0.231 no Transcriptional repressor EthR <UNP P96222_MYCTU> [MYCOBACTERIUM TUBERCULOSIS]
MGSSHHHHHHSSGLVPRGSHVTTSAASQASLPRGRRTARPSGDDRELAILATAENLLEDR
PLADISVDDLAKGAGISRPTFYFYFPSKEAVLLTLLDRVVNQADMALQTLAENPADTDRE
NMWRTGINVFFETFGSHKAVTRAGQAARATSVEVAELWSTFMQKWIAYTAAVIDAERDRG
AAPRTLPAHELATALNLMNERTLFASFAGEQPSVPEARVLDTLVHIWVTSIYGENR
>3II6X 263 XRAY 2.40 0.240 0.280 no DNA ligase 4 <UNP DNLI4_HUMAN> [HOMO SAPIENS]
GAMGSKISNIFEDVEFCVMSGTDSQPKPDLENRIAEFGGYIVQNPGPDTYCVIAGSENIR
VKNIILSNKHDVVKPAWLLECFKTKSFVPWQPRFMIHMCPSTKEHFAREYDCYGDSYFID
TDLNQLKEVFSGIKNSNEQTPEEMASLIADLEYRYSWDCSPLSMFRRHTVYLDSYAVIND
LSTKNEGTRLAIKALELRFHGAKVVSCLAEGVSHVIIGEDHSRVADFKAFRRTFKRKFKI
LKESWVTDSIDKCELQEENQYLI
>2DA6A 102 NMR NA NA NA no Hepatocyte nuclear factor 1-beta <UNP HNF1B_HUMAN> [HOMO SAPIENS]
GSSGSSGRNRFKWGPASQQILYQAYDRQKNPSKEEREALVEECNRAECLQRGVSPSKAHG
LGSNLVTEVRVYNWFANRRKEEAFRQKLAMDAYSSNSGPSSG

>3DPJA 194 XRAY 1.90 0.196 0.243 no Transcription regulator, TetR family <UNP Q5LS67_SILPO> [SILICIBACTER POMEROYI]
SNAMVQAQTRDQIVAAADELFYRQGFAQTSFVDISAAVGISRGNFYYHFKTKDEILAEVI
RLRLARTAQMLADWQGTGDSPRARIASFIDLMIMNRAKITRYGCPVGSLCTELSKLDHAA
QGQANGLFTLFRDWLQRQFAEAGCTTEAPALAMHLLARSQGAATLAQSFHDEGFLRSEVA
DMHRWLDNTLPMTT
>1TC3C 51 XRAY 2.45 0.234 0.318 no TC3 TRANSPOSASE <UNP TC3A_CAEEL> [CAENORHABDITIS ELEGANS]
PRGSALSDTERAQLDVMKLLNVSLHEMSRKISRSRHCIRVYLKDPVSYGTS
>3EZ2A 398 XRAY 2.05 0.191 0.232 no Plasmid partition protein A <UNP PARA_ECOLX> [ESCHERICHIA COLI]
MSDSSQLHKVAQRANRMLNVLTEQVQLQKDELHANEFYQVYAKAALAKLPLLTRANVDYA
VSEMEEKGYVFDKRPAGSSMKYAMSIQNIIDIYEHRGVPKYRDRYSEAYVIFISNLKGGV
SKTVSTVSLAHAMRAHPHLLMEDLRILVIDLDPQSSATMFLSHKHSIGIVNATSAQAMLQ
NVSREELLEEFIVPSVVPGVDVMPASIDDAFIASDWRELCNEHLPGQNIHAVLKENVIDK
LKSDYDFILVDSGPHLDAFLKNALASANILFTPLPPATVDFHSSLKYVARLPELVKLISD
EGCECQLATNIGFMSKLSNKADHKYCHSLAKEVFGGDMLDVFLPRLDGFERCGESFDTVI
SANPATYVGSADALKNARIAAEDFAKAVFDRIEFIRSN
>2Q2KA 70 XRAY 3.00 0.258 0.297 no Hypothetical protein <UNP Q2FDA3_STAA3> [STAPHYLOCOCCUS AUREUS]
MGSSHHHHHHSSGLVPGSHMDKKETKHLLKIKKEDYPQIFDFLENVPRGTKTAHIREALR
RYIEEIGENP
>3GE4A 173 XRAY 1.70 0.145 0.179 no DNA PROTECTION DURING STARVATION PROTEIN <UNP

Q8YE98_BRUME> [BRUCELLA MELITENSIS]
MAHHHHHHMPKKSMHATRNDLPSNTKTTMIALLNENLAATIDLALITKQAHWNLKGPQFI
AVHEMLDGFRAELDDHVDTIAERAVQIGGTAYGTTQVVVKESRLKPYPTDIYAVHDHLVA
LIERYGDVANLVRKSIKDADDAGDDDTADIFTAASRSLDKALWFLEAHVQESN
>1U3EM 174 XRAY 2.92 0.220 0.247 no HNH homing endonuclease <UNP YG31_BPSP1>
[BACILLUS PHAGE SPO1]
MEWKDIKGYEGHYQVSNTGEVYSIKSGKTLKHQIPKDGYHRIGLFKGGKGKTFQVHRLVA
IHFCEGYEEGLVVDHKDGNKDNNLSTNLRWVTQKINVENQMSRGTLNVSKAQQIAKIKNQ
KPIIVISPDGIEKEYPSTKCACEELGLTRGKVTDVLKGHRIHHKGYTFRYKLNG
>2CYYA 151 XRAY 1.80 0.220 0.245 no Putative HTH-type transcriptional regulator
PH1519
<UNP REG6_PYRHO> [PYROCOCCUS HORIKOSHII]
MRVPLDEIDKKIIKILQNDGKAPLREISKITGLAESTIHERIRKLRESGVIKKFTAIIDP
EALGYSMLAFILVKVKAGKYSEVASNLAKYPEIVEVYETTGDYDMVVKIRTKNSEELNNF
LDLIGSIPGVEGTHTMIVLKTHKETTELPIK
>3FPNA 119 XRAY 1.80 0.231 0.248 no Geobacillus stearothermophilus UvrA
interaction domain <PDB 3FPN> [GEOBACILLUS STEAROTHERMOPHILUS]
GSHMTIEQMVDRLLSYPERTKMQILAPIVSGKKGTHAKTLEDIRKQGYVRVRIDREMREL
TGDIELEKNKKHSIDVVVDRIIIKDGIAARLADSLETALKLADGKVVVDVIGEGELLFS

>1XVPA 236 XRAY 2.60 0.180 0.234 no Retinoic acid receptor RXR-alpha <UNP
RXRA_HUMAN> [HOMO SAPIENS]
NEDMPVERILEAELAVEPKTETYVEANMGLNPSSPNDPVTNICQAADKQLFTLVEWAKRI
PHFSELPLDDQVILLRAGWNELLIASFSHRSIAVKDGILLATGLHVHRNSAHSAGVGAIF
DRVLTELVSKMRDMQMDKTELGCLRAIVLFNPDSKGLSNPAEVEALREKVYASLEAYCKH
KYPEQPGRFAKLLLRLPALRSIGLKCLEHLFFFKLIGDTPIDTFLMEMLEAPHQMT

>1KZYC 259 XRAY 2.50 0.216 0.256 no TUMOR SUPPRESSOR P53-BINDING PROTEIN 1 <UNP
TP53B_HUMAN> [HOMO SAPIENS]
ALEEQRGPLPLNKTLFLGYAFLLTMATTSDKLASRSKLPDGPTGSSEEEEEFLEIPPFNK
QYTESQLRAGAGYILEDFNEAQCNTAYQCLLIADQHCRTRKYFLCLASGIPCVSHVWVHD
SCHANQLQNYRNYLLPAGYSLEEQRILDWQPRENPFQNLKVLLVSDQQQNFLELWSEILM
TGGAASVKQHHSSAHNKDIALGVFDVVVTDPSCPASVLKCAEALQLPVVSQEWVIQCLIV
GERIGFKQHPKYKHDYVSH
>1YJMA 110 XRAY 2.20 0.213 0.243 no Polynucleotide 5'-hydroxyl-kinase <UNP
PNKP_MOUSE> [MUS MUSCULUS]
MSQLGSRGRLWLQSPTGGPPPIFLPSDGQALVLGRGPLTQVTDRKCSRNQVELIADPESR
TVAVKQLGVNPSTVGVHELKPGLSGSLSLGDVLYLVNGLYPLTLRWEELS
>2NTIB 246 XRAY 2.50 0.211 0.251 no DNA polymerase sliding clamp C <UNP PCNA3_SULSO>
[SULFOLOBUS SOLFATARICUS]
MMKAKVIDAVSFSYILRTVGDFLSEANFIVTKEGIRVSGIDPSRVVFLDIFLPSSYFEGF
EVSQEKEIIGFKLEDVNDILKRVLKDDTLILSSNESKLTLTFDGEFTRSFELPLIQVEST
QPPSVNLEFPFKAQLLTITFADIIDELSDLGEVLNIHSKENKLYFEVIGDLSTAKVELST
DNGTLLEASGADVSSSYGMEYVANTTKMRRASDSMELYFGSQIPLKLRFKLPQEGYGDFY
IAPRAD
>4JW3C 139 XRAY 2.60 0.226 0.264 no Alpha-helical artificial proteins <PDB 4JW3>
[SYNTHETIC CONSTRUCT]

MRGSHHHHHHTDPEKVEMYIKNLQDDSYFVRRAAAAALGKIGDERAVEPLIKALKDEDRF
VRSSAAYALGEIGDERAVEPLIKALKDEDWFVRRAAAVALGEIGGERVRAAMEKLAETGT
GFARKVAVNYLETHKSLIS
>3JTZA 88 XRAY 1.30 0.214 0.239 no Integrase <UNP Q9Z3B4_YERPE> [YERSINIA PESTIS]
MSLTDAKIRTLKPSDKPFKVSDSHGLYLLVKPGGSRHWYLKYRISGKESRIALGAYPAIS
LSDARQQREGIRKMLALNINLEHHHHHH
>1IYMA 55 NMR NA NA NA no EL5 <UNP EL5_ORYSA> [ORYZA SATIVA]
AMDDGVECAVCLAELEDGEEARFLPRCGHGFHAECVDMWLGSHSTCPLCRLTVVV
>3ASLA 70 XRAY 1.41 0.161 0.196 no E3 ubiquitin-protein ligase UHRF1 <UNP
UHRF1_HUMAN> [HOMO SAPIENS]
SGPSCKHCKDDVNRLCRVCACHLCGGRQDPDKQLMCDECDMAFHIYCLDPPLSSVPSEDE
WYCPECRNDA
>3M9QA 101 XRAY 1.29 0.171 0.192 no Protein male-specific lethal-3 <UNP MSL3_DROME>
[DROSOPHILA MELANOGASTER]
MKKHHHHHHMTELRDETPLFHKGEIVLCYEPDKSKARVLYTSKVLNVFERRNEHGLRFYE
YKIHFQGWRPSYDRAVRATVLLKDTEENRQLQRELAEAAKL
>1U2WA 122 XRAY 1.90 0.236 0.261 no Cadmium efflux system accessory protein <UNP
CADC_STAAU> [STAPHYLOCOCCUS AUREUS]
MKKKDTCEIFGYDEEKVNRIQGDLQTVDISGVSQILKAIADENRAKITYALCQDEELCVC
DIANILGVTIANASHHLRTLYKQGVVNFRKEGKLALYSLGDEHIRQIMMIALAHKKEVKV
NV
>3U61A 199 XRAY 3.20 0.244 0.305 no DNA polymerase accessory protein 62 <UNP
DPA62_BPT4> [ENTEROBACTERIA PHAGE T4]
SLFKDDIQLNEHQVAWYSKDWTAVQSAADSFKEKAENEFFEIIGAINNKTKCSIAQKDYS
KFMVENALSQFPECMPAVYAMNLIGSGLSDEAHFNYLMAAVPRGKRYGKWAKLVEDSTEV
LIIKLLAKRYQVNTNDAINYKSILTKNGKLPLVLKELKGLVTDDFLKEVTKNVKEQKQLK
KLALEWGLEHHHHHHHHHH

>4LG8A 354 XRAY 1.89 0.158 0.197 no Pre-mRNA-processing factor 19 <UNP PRP19_HUMAN>
[HOMO SAPIENS]
MHHHHHHSSGRENLYFQGTPEIIQKLQDKATVLTTERKERGKTVPEELVKPEELSKYRQV
ASHVGLHSASIPGILALDLCPSDTNKILTGGADKNVVVFDKSSEQILATLKGHTKKVTSV
VFHPSQDLVFSASPDATIRIWSVPNASCVQVVRAHESAVTGLSLHATGDYLLSSSDDQYW
AFSDIQTGRVLTKVTDETSGCSLTCAQFHPDGLIFGTGTMDSQIKIWDLKERTNVANFPG
HSGPITSIAFSENGYYLATAADDSSVKLWDLRKLKNFKTLQLDNNFEVKSLIFDQSGTYL
ALGGTDVQIYICKQWTEILHFTEHSGLTTGVAFGHHAKFIASTGMDRSLKFYSL
>2ZDSA 340 XRAY 2.30 0.167 0.210 no Putative DNA-binding protein <UNP O69946_STRCO>
[STREPTOMYCES COELICOLOR]
MPRNFTLFTGQWADLPLEEVCRLARDFGYDGLELACWGDHFEVDKALADPSYVDSRHQLL
DKYGLKCWAISNHLVGQAVCDAIIDERHEAILPARIWGDGDAEGVRQRAAAEIKDTARAA
ARLGVDTVIGFTGSAIWHLVAMFPPAPESMIERGYQDFADRWNPILDVFDAEGVRFAHEV
HPSEIAYDYWTTHRALEAVGHRPAFGLNFDPSHFVWQDLDPVGFLWDFRDRIYHVDCKEA
RKRLDGRNGRLGSHLPWGDPRRGWDFVSAGHGDVPWEDVFRMLRSIDYQGPVSVEWEDAG
MDRLQGAPEALTRLKAFDFEPPSASFDAAFNSLEHHHHHH
>2OD5A 116 XRAY 1.79 0.190 0.213 no hypothetical protein <PDB 2OD5> [UNCULTURED
MARINE ORGANISM]
GMTGAVETESMKTVRIREKIKKFLGDRPRNTAEILEHINSTMRHGTTSQQLGNVLSKDKD

IVKVGYIKRSGILSGGYDICEWATRNWVAEHCPEWTEGQPIILNEEGDFTLGPLPE
>4IRGA 109 XRAY 1.70 0.190 0.228 no Transcriptional regulator ERG <UNP ERG_HUMAN> [HOMO SAPIENS]
GAMGSGIQRPGSGQIQLWQFLLELLSDSSNSSCITWEGTNGEFKMTDPDEVARRWGERKS
KPNMNYDKLSRALRYYYDKNIMTKVHGKRYAYKFDFHGIAQALQPHPPE
>1YIOA 208 XRAY 2.20 0.205 0.275 no response regulatory protein <UNP O30989_PSEFL> [PSEUDOMONAS FLUORESCENS]
MTAKPTVFVVDDDMSVREGLRNLLRSAGFEVETFDCASTFLEHRRPEQHGCLVLDMRMPG
MSGIELQEQLTAISDGIPIVFITAHGDIPMTVRAMKAGAIEFLPKPFEEQALLDAIEQGL
QLNAERRQARETQDQLEQLFSSLTGREQQVLQLTIRGLMNKQIAGELGIAEVTVKVHRHN
IMQKLNVRSLANLVHLVEKYESFERGVS
>2ICTA 94 XRAY 1.63 0.198 0.201 no antitoxin higa <UNP YDDM_ECOLI> [ESCHERICHIA COLI] MKMANHPRPGDIIQESLDELNVSLREFARAMEIAPSTASRLLTGKAALTPEMAIKLSVVI
GSSPQMWLNLQNAWSLAEAEKTVDVSRLRRLVTQ
>2QUQA 562 XRAY 2.80 0.228 0.285 no Centromere DNA-binding protein complex CBF3 subunit B <UNP CBF3B_YEAST> [SACCHAROMYCES CEREVISIAE]
KLITASSSKEYLPDLLLFWQNYEYWITNIGLYKTKQRDLTRTPANLDTDTEECMFWMNYL
QKDQSFQLMNFAMENLGALYFGSIGDISELYLRVEQYWDRRADKNHSVDGKYWDALIWSV
FTMCIYYMPVEKLAEIFSVYPLHEYLGSNKRLNWEDGMQLVMCQNFARCSLFQLKQCDFM
AHPDIRLVQAYLILATTTFPYDEPLLANSLLTQCIHTFKNFHVDDFRPLLNDDPVESIAK
VTLGRIFYRLCGCDYLQSGPRKPIALHTEVSSLLQHAAYLQDLPNVDVYREENSTEVLYW
KIISLDRDLDQYLNKSSKPPLKTLDAIRRELDIFQYKVDSLEEDFRSNNSRFQKFIALFQ
ISTVSWKLFKMYLIYYDTADSLLKVIHYSKVIISLIVNNFHAKSEFFNRHPMVMQTITRV
VSFISFYQIFVESAAVKQLLVDLTELTANLPTIFGSKLDKLVYLTERLSKLKLLWDKVQL
LDSGDSFYHPVFKILQNDIKIIELKNDEMFSLIKGLGSLVPLNKLRQESLLEEEDENNTE
PSDFRTIVEEFQSEYNISDILS
>2K6GA 109 NMR NA NA NA no Replication factor C subunit 1 <UNP RFC1_HUMAN> [HOMO SAPIENS]

KRTNYQAYRSYLNREGPKALGSKEIPKGAENCLEGLIFVITGVLESIERDEAKSLIERYG
GKVTGNVSKKTNYLVMGRDSGQSKSDKAAALGTKIIDEDGLLNLIRNLE
>1X6FA 88 NMR NA NA NA no Zinc finger protein 462 <UNP ZN462_HUMAN> [HOMO SAPIENS]
GSSGSSGLKRDFIILGNGPRLQNSTYQCKHCDSKLQSTAELTSHLNIHNEEFQKRAKRQE
RRKQLLSKQKYADGAFADFKQESGPSSG
>3AAFA 134 XRAY 1.90 0.213 0.244 no Werner syndrome ATP-dependent helicase <UNP WRN_HUMAN> [HOMO SAPIENS]
GIRMDDSEDTSWDFGPQAFKLLSAVDILGEKFGIGLPILFLRGSNSQRLADQYRRHSLFG
TGKDQTESWWKAFSRQLITEGFLVEVSRYNKFMKICALTKKGRNWLHKANTESQSLILQA
NEELCPKKFLLPSS
>4HIKA 143 XRAY 1.64 0.195 0.216 no Protection of telomeres protein 1 <UNP POT1_SCHPO> [SCHIZOSACCHAROMYCES POMBE]
MSDSFSLLSQITPHQRCSFYAQVIKTWYSDKNFTLYVTDYTENELFFPMSPYTSSSRWRG
PFGRFSIRCILWDEHDFYCRNYIKEGDYVVMKNVRTKIDHLGYLECILHGDSAKRYNMSI
EKVDSEEPELNEIKSRKRLYVQN
>3NCTA 144 XRAY 2.20 0.186 0.210 no Protein psiB <UNP PSIB1_ECOLI> [ESCHERICHIA COLI] MKTELTLNVLQTMNAQEYEDIRAAGSDERRELTHAVMRELDAPDNWTMNGEYGSEFGGFF
PVQVRFTPAHERFHLALCSPGDVSQVWVLVLVNAGGEPFAVVQVQRRFASEAVSHSLALA

ASLDTQGYSVNDIIHILMAEGGQV
>3KXTA 56 XRAY 1.60 0.178 0.217 no Chromatin protein Cren7 <UNP CREN7_SULSO> [SULFOLOBUS SOLFATARICUS]
MKPVKVKTPAGKEAELVPEKVWALAPKGRKGVKIGLFKDPETGKYFRHKLPDDYPI
>2AIFA 135 XRAY 1.90 0.203 0.270 no ribosomal protein L7A <UNP Q7YYQ3_CRYPV> [CRYPTOSPORIDIUM PARVUM]
GSSQNEASEDTGFNPKAFPLASPDLNNKIINLVQQACNYKQLRKGANEATKALNRGIAEI
VLLAADAEPLEILLHLPLVCEDKNTPYVFVRSKVALGRACGVSRPVIAAAITSKDGSSLS
SQITELKDQIEQILV
>4AC0A 956 XRAY 1.89 0.194 0.232 no CENTROMERE DNA-BINDING PROTEIN COMPLEX CBF3 SUBUNIT A <UNP CBF3A_YEAST> [SACCHAROMYCES CEREVISIAE]
MRSSILFLLKLMKIMDVQQQQEAMSSEDRFQELVDSLKPRTAHQYKTYYTKYIQWCQLNQ
IIPTPEDNSVNSVPYKDLPISAELIHWFLLDTLITDDKPGEKREETEDLDEEEENSFKIA
TLKKIIGSLNFLSKLCKVHENPNANIDTKYLESVTKLHTHWIDSQKAITTNETNNTNTQV
LCPPLLKVSLNLWNPETNHLSEKFFKTCSEKLRFLVDFQLRSYLNLSFEERSKIRFGSLK
LGKRDRDAIIYHKVTHSAEKKDTPGHHQLLALLPQDCPFICPQTTLAAYLYLRFYGIPSV
SKGDGFPNLNADENGSLLQDIPILRGKSLTTYPREETFSNYYTTVFRYCHLPYKRREYFN
KCNLVYPTWDEDTFRTFFNEENHGNWLEQPEAFAFPDKIPFDFKKIMNFKSPYTSYSTNA
KKDPFPPPKDLLVQIFPEIDEYKRHDYEGLSQNSRDFLDLMEVLRERFLSNLPWIYKFFP
NHDIFQDPIFGNSDFQSYFNDKTIHSKGSPILSFDILPGFNKIYKNKTNFYSLLIERPSQ
LTFASSHNPDTHPTQKQESEGPLQMSQLDTTQLNELLKQQSFEYVQFQTLSNFQILLSVF
NKIFEKLEMKKSSRGYILHQLNLFKITLDERIKKSKIDDADKFIRDNQPIKKEENIVNED
GPNTSRRTKRPKQIRLLSIADSSDESSTEDSNVFKKDGESIEDGAYGENEDENDSEMQEQ
LKSMINELINSKISTFLRDQMDQFELKINALLDKILEEKVTRIIEQKLGSHTGKFSTLKR
PQLYMTEEHNVGFDMEVPKKLRTSGKYAETVKDNDDHQAMSTTASPSPEQDQEAKSYTDE
QEFMLDKSIDSIEGIILEWFTPNAKYANQCVHSMNKSGNKSWRANCEALYKERKSIVEFY
IYLVNHESLDRYKAVDICEKLRDQNEGSFSRLAKFLRKWRHDHQNSFDGLLVYLSN
>1PL5A 142 XRAY 2.50 0.261 0.288 no Regulatory protein SIR4 <UNP SIR4_YEAST> [SACCHAROMYCES CEREVISIAE]

SNTTEILTSVDVLGTHSQTGTQQSNMYTSTQKTELEIDNKDSVTECSKDMKEDGLSFVDI
VLSKAASALDEKEKQLAVANEIIRSLSDEVMRNEIRITSLQGDLTFTKKCLENARSQISE
KDAKINKLMEKDFQVNKEIKPY
>1CI4A 89 XRAY 1.90 0.210 0.262 no BARRIER-TO-AUTOINTEGRATION FACTOR (BAF) <UNP BAF_HUMAN> [HOMO SAPIENS]
MTTSQKHRDFVAEPMGEKPVGSLAGIGEVLGKKLEERGFDKAYVVLGQFLVLKKDEDLFR
EWLKDTCGANAKQSRDCFGCLREWCDAFL
>1OY3B 136 XRAY 2.05 0.219 0.247 no Transcription factor p65 <UNP TF65_MOUSE> [MUS MUSCULUS]
TAELKICRVNRNSGSCLGGDEIFLLCDKVQKEDIEVYFTGPGWEARGSFSQADVHRQVAI
VFRTPPYADPSLQAPVRVSMQLRRPSDRELSEPMEFQYLPDTDDRHRIEEKRKRTYETFK SIMKKSPFNGPTEPRP
>1PGZA 195 XRAY 2.60 0.237 0.269 no Heterogeneous nuclear ribonucleoprotein A1 <UNP ROA1_HUMAN> [HOMO SAPIENS]
SKSESPKEPEQLRKLFIGGLSFETTDESLRSHFEQWGTLTDCVVMRDPNTKRSRGFGFVT
YATVEEVDAAMNARPHKVDGRVVEPKRAVSREDSQRPGAHLTVKKIFVGGIKEDTEEHHL
RDYFEQYGKIEVIEIMTDRGSGKKRGFAFVTFDDHDSVDKIVIQKYHTVNGHNCEVRKAL
SKQEMASASSSQRGR

>3UX8A 670 XRAY 2.10 0.212 0.245 no Excinuclease ABC, A subunit <UNP E8SW61_GEOS2>
[GEOBACILLUS]
MGSSHHHHHHSSGLVPRGSHMDKIIVKGARAHNLKNIDVEIPRGKLVVLTGLSGSGKSSL
AFDTIYAEGQRRYVESLSAYARQFLGQMEKPDVDAIEGLSPAISIDQKTTSRNPRSTVGT
VTEIYDYLRLLFARIGRLVGGKHIGEVTAMSVTEALAFFDGLELTEKEAQIARLILREIR
DRLGFLQNVGLDYLTLSRSAGTLSGGEAQRIRLATQIGSRLTGVLYVLDEPSIGLHQRDN
DRLIATLKSMRDLGNTLIVVEHDEDTMLAADYLIDIGPGAGIHGGEVVAAGTPEEVMNDP
NSLTGQYLSGKKFIPIPAERRRPDGRWLEVVGAREHNLKNVSVKIPLGTFVAVTGVSGSG
KSTLVNEVLYKALAQKLHRAKAKPGEHRDIRGLEHLDKVIDIDQSPIGRTPRSNPATYTG
VFDDIRDVFASTNEAKVRGYKKGRFSFNVKGGRCEACHGDGIIKIEMHFLPDVYVPCEVC
HGKRYNRETLEVTYKGKNIAEVLDMTVEDALDFFASIPKIKRKLETLYDVGLGYMKLGQP
ATTLSGGEAQRVKLAAELHRRSNGRTLYILDEPTTGLHVDDIARLLDVLHRLVDNGDTVL
VIEHNLDVIKTADYIIDLGPEGGDRGGQIVAVGTPEEVAEVKESHTGRYLKPILERDRAR
MQARYEAAKA
>1L8YA 91 NMR NA NA NA no upstream binding factor 1 <UNP UBF1_HUMAN> [HOMO SAPIENS]
MGKLPESPKRAEEIWQQSVIGDYLARFKNDRVKALKAMEMTWNNMEKKEKLMWIKKAAED
QKRYERELSEMRAPPAATNSSKKLEHHHHHH
>4ER8A 165 XRAY 2.60 0.228 0.281 no TnpArep for protein <UNP YAFM_ECOLI>
[ESCHERICHIA COLI]
MSEYRRYYIKGGTWFFTVNLRNRRSQLLTTQYQMLRHAIIKVKRDRPFEINAWVVLPEHM
HCIWTLPEGDDDFSSRWREIKKQFTHACGLKNIWQPRFWEHAIRNTKDYRHHVDYIYINP
VKHGWVKQVSDWPFSTFHRDVARGLYPIDWAGDVTDFSAGERIIS
>1OV9A 50 XRAY 2.30 0.258 0.269 no VicH protein <UNP Q9KSX6_VIBCH> [VIBRIO
CHOLERAE] SEITKTLLNIRSLRAYARELTIEQLEEALDKLTTVVQERKEAEAEEIAAR
>3QMDA 79 XRAY 1.90 0.225 0.241 no CpG-binding protein <UNP CXXC1_HUMAN> [HOMO
SAPIENS] MHHHHHHSSRENLYFQGQIKRSARMCGECEACRRTEDCGHCDFCRDMKKFGGPNKIRQKC
RLRQCQLRARESYKYFPSS
>1IRZA 64 NMR NA NA NA no ARR10-B <UNP ARR10_ARATH> [ARABIDOPSIS THALIANA]
TAQKKPRVLWTHELHNKFLAAVDHLGVERAVPKKILDLMNVDKLTRENVASHLQKFRVAL

KKVS
>3FYMA 130 XRAY 1.00 0.110 0.140 no Putative uncharacterized protein <UNP
Q99UJ0_STAAM> [STAPHYLOCOCCUS AUREUS SUBSP. AUREUS MU50]
MKTVGEALKGRRERLGMTLTELEQRTGIKREMLVHIENNEFDQLPNKNYSEGFIRKYASV
VNIEPNQLIQAHQDEIPSNQAEWDEVITVFNNNKDLDYKSKSKEPIQLLVIMGITVLITL
LLWIMLVLIF
>1OQJA 97 XRAY 1.55 0.234 0.210 no Glucocorticoid Modulatory Element Binding
protein-1
<UNP GMEB1_HUMAN> [HOMO SAPIENS]
GAMEDMEIAYPITCGESKAILLWKKFVCPGINVKCVKFNDQLISPKHFVHLAGKSTLKDW
KRAIRLGGIMLRKMMDSGQIDFYQHDKVCSNTCRSTK
>4GS3A 107 XRAY 1.09 0.154 NA no Single-stranded DNA-binding protein <UNP
Q8RBI8_THETN> [THERMOANAEROBACTER TENGCONGENSIS]
SNAMAGNFLENNTVTLVGKVFTPLEFSHELYGEKFFNFILEVPRLSETKDYLPITISNRL
FEGMNLEVGTRVKIEGQLRSYNRKSPEEGKNKLILTVFARDISVVPE
>3V9RB 88 XRAY 2.40 0.242 0.290 no Uncharacterized protein YDL160C-A <UNP
YD160_YEAST> [SACCHAROMYCES CEREVISIAE]

MLSKEALIKILSQNEGGNDMKIADEVVPMIQKYLDIFIDEAVLRSLQSHKDINGERGDKS
PLELSHQDLERIVGLLLMDMLEHHHHHH
>2DT5A 211 XRAY 2.16 0.195 0.238 no AT-rich DNA-binding protein <UNP Q5SHS3_THET8>
[THERMUS THERMOPHILUS]
MKVPEAAISRLITYLRILEELEAQGVHRTSSEQLGGLAQVTAFQVRKDLSYFGSYGTRGV
GYTVPVLKRELRHILGLNRKWGLCIVGMGRLGSALADYPGFGESFELRGFFDVDPEKVGR
PVRGGVIEHVDLLPQRVPGRIEIALLTVPREAAQKAADLLVAAGIKGILNFAPVVLEVPK
EVAVENVDFLAGLTRLSFAILNPKWREEMMG
>1Y6UA 70 NMR NA NA NA no Excisionase from transposon Tn916 <UNP Q79DA1_ENTFA>
[ENTEROCOCCUS FAECALIS]
AGHMKQTDIPIWERYTLTIEEASKYFRIGENKLRRLAEENKNANWLIMNGNRIQIKRKQF
EKIIDTLDAI
>3FRQA 195 XRAY 1.76 0.183 0.221 no Repressor protein MphR(A) <UNP Q9EVJ6_ECOLX>
[ESCHERICHIA COLI]
GMPRPKLKSDDEVLEAATVVLKRCGPIEFTLSGVAKEVGLSRAALIQRFTNRDTLLVRMM
ERGVEQVRHYLNAIPIGAGPQGLWEFLQVLVRSMNTRNDFSVNYLISWYELQVPELRTLA
IQRNRAVVEGIRKRLPPGAPAAAELLLHSVIAGATMQWAVDPDGELADHVLAQIAAILCL MFPEHDDFQLLQAHA
>1USTA 93 NMR NA NA NA no HISTONE H1 <UNP H1_YEAST> [SACCHAROMYCES CEREVISIAE]
KKEEASSKSYRELIIEGLTALKERKGSSRPALKKFIKENYPIVGSASNFDLYFNNAIKKG
VEAGDFEQPKGPAGAVKLAKKKSPEVKKEKEVS
>3S4WB 1323 XRAY 3.41 0.241 0.272 no Fanconi anemia group D2 protein homolog <UNP
FACD2_MOUSE> [MUS MUSCULUS]
SHNSHEVEENGSVFVKLLKASGLTLKTGENQNQLGVDQVIFQRKLFQALRKHPAYPKVIE
EFVNGLESYTEDSESLRNCLLSCERLQDEEASMGTFYSKSLIKLLLGIDILQPAIIKMLF
EKVPQFLFESENRDGINMARLIINQLKWLDRIVDGKDLTAQMMQLISVAPVNLQHDFITS
LPEILGDSQHANVGKELGELLVQNTSLTVPILDVFSSLRLDPNFLSKIRQLVMGKLSSVR
LEDFPVIVKFLLHSVTDTTSLEVIAELRENLNVQQFILPSRIQASQSKLKSKGLASSSGN
QENSDKDCIVLVFDVIKSAIRYEKTISEAWFKAIERIESAAEHKSLDVVMLLIIYSTSTQ
TKKGVEKLLRNKIQSDCIQEQLLDSAFSTHYLVLKDICPSILLLAQTLFHSQDQRIILFG
SLLYKYAFKFFDTYCQQEVVGALVTHVCSGTEAEVDTALDVLLELIVLNASAMRLNAAFV

KGILDYLENMSPQQIRKIFCILSTLAFSQQPGTSNHIQDDMHLVIRKQLSSTVFKYKLIG
IIGAVTMAGIMAEDRSVPSNSSQRSANVSSEQRTQVTSLLQLVHSCTEHSPWASSLYYDE
FANLIQERKLAPKTLEWVGQTIFNDFQDAFVVDFCAAPEGDFPFPVKALYGLEEYSTQDG
IVINLLPLFYQECAKDASRATSQESSQRSMSSLCLASHFRLLRLCVARQHDGNLDEIDGL
LDCPLFLPDLEPGEKLESMSAKDRSLMCSLTFLTFNWFREVVNAFCQQTSPEMKGKVLSR
LKDLVELQGILEKYLAVIPDYVPPFASVDLDTLDMMPRKTFVSLQNYRAFFRELDIEVFS
ILHSGLVTKFILDTEMHTEATEVVQLGPAELLFLLEDLSQKLENMLTAPFAKRICCFKNK
GRQNIGFSHLHQRSVQDIVHCVVQLLTPMCNHLENIHNFFQCLGAEHLSADDKARATAQE
QHTMACCYQKLLQVLHALFAWKGFTHQSKHRLLHSALEVLSNRLKQMEQDQPLEELVSQS
FSYLQNFHHSVPSFQCGLYLLRLLMALLEKSAVPNQKKEKLASLAKQLLCRAWPHGEKEK
NPTFNDHLHDVLYIYLEHTDNVLKAIEEITGVGVPELVSAPKDAASSTFPTLTRHTFVIF
FRVMMAELEKTVKGLQAGTAADSQQVHEEKLLYWNMAVRDFSILLNLMKVFDSYPVLHVC
LKYGRRFVEAFLKQCMPLLDFSFRKHREDVLSLLQTLQLNTRLLHHLCGHSKIRQDTRLT
KHVPLLKKSLELLVCRVKAMLVLNNCREAFWLGTLKNRDLQGEEIISQDPSSSESNAEDS
EDG
>2FXQA 264 XRAY 1.85 0.192 0.247 no Single-strand binding protein <UNP SSB_THEAQ>

[THERMUS AQUATICUS]
MARGLNQVFLIGTLTARPDMRYTPGGLAILDLNLAGQDAFTDESGQEREVPWYHRVRLLG
RQAEMWGDLLEKGQLIFVEGRLEYRQWEKDGEKKSEVQVRAEFIDPLEGRGRETLEDARG
QPRLRRALNQVILMGNLTRDPDLRYTPQGTAVVRLGLAVNERRRGQEEERTHFLEVQAWR
ELAEWASELRKGDGLLVIGRLVNDSWTSSSGERRFQTRVEALRLERPTRGPAQAGGSRPP
TVQTGGVDIDEGLEDFPPEEDLPF
>1ZI0A 307 XRAY 2.60 0.227 0.266 no DNA gyrase subunit A <UNP GYRA_ECOLI>
[ESCHERICHIA COLI]
TQEDVVVTLSHQGYVKYQPLSEYEAQRRGGKGKSAARIKEEDFIDRLLVANTHDHILCFS
SRGRVYSMKVYQLPEATRGARGRPIVNLLPLEQDERITAILPVTEFEEGVKVFMATANGT
VKKTVLTEFNRLRTAGKVAIKLVDGDELIGVDLTSGEDEVMLFSAEGKVVRFKESSVRAM
GCNTTGVRGIRLGEGDKVVSLIVPRGDGAILTATQNGYGKRTAVAEYPTKSRATKGVISI
KVTERNGLVVGAVQVDDCDQIMMITDAGTLVRTRVSEISIVGRNTQGVILIRTAEDENVV
GLQRVAE
>1KKXA 123 NMR NA NA NA no Transcription regulatory protein ADR6 <UNP SWI1_YEAST>
[SACCHAROMYCES CEREVISIAE]
MRGSGSHHHHHHGSNNKQYELFMKSLIENCKKRNMPLQSIPEIGNRKINLFYLYMLVQKF
GGADQVTRTQQWSMVAQRLQISDYQQLESIYFRILLPYERHMISQEGIKETQAKRILQPS
LIS
>2K75A 106 NMR NA NA NA no uncharacterized protein Ta0387 <UNP Q9HL44_THEAC>
[THERMOPLASMA ACIDOPHILUM]
SDLVKIRDVSLSTPYVSVIGKITGIHKKEYESDGTTKSVYQGYIEDDTARIRISSFGKQL
QDSDVVRIDNARVAQFNGYLSLSVGDSSRIESVNVNIPLEHHHHHH
>3V60A 84 XRAY 2.60 0.212 0.249 no Ubiquitin-like protein SMT3 <UNP SMT3_YEAST>
[SACCHAROMYCES CEREVISIAE]
GSHMRPETHINLKVSDGSSEIFFKIKKTTPLRRLMEAFAKRQGKEMDSLRFLYDGIRIQA
DQTPEDLDMEDNDIIEAHREQIGG
>2HX0A 154 XRAY 1.55 0.197 0.209 no Putative DNA-binding protein <UNP Q57K43_SALCH>
[SALMONELLA CHOLERAESUIS]
MAGDPNSMTVSHHNASTARFYALRLLPGQEVFSQLHAFVQQNQLRAAWIAGCTGSLTDVA
LRYAGQEATTSLTGTFEVISLNGTLELTGEHLHLAVSDPYGVMLGGHMMPGCTVRTTLEL

VIGELPALTFSRQPCAISGYDELHISSRLEHHHH
>1T0FA 276 XRAY 1.85 0.211 0.235 no Transposon Tn7 transposition protein tnsA
<UNP TNSA_ECOLI> [ESCHERICHIA COLI]
GSAMAKANSSFSEVQIARRIKEGRGQGHGKDYIPWLTVQEVPSSGRSHRIYSHKTGRVHH
LLSDLELAVFLSLEWESSVLDIREQFPLLPSDTRQIAIDSGIKHPVIRGVDQVMSTDFLV
DCKDGPFEQFAIQVKPAAALQDERTLEKLELERRYWQQKQIPWFIFTDKEINPVVKENIE
WLYSVKTEEVSAELLAQLSPLAHILQEKGDENIINVCKQVDIAYDLELGKTLSEIRALTA
NGFIKFNIYKSFRANKCADLCISQVVNMEELRYVAN
>2O3CA 282 XRAY 2.30 0.199 0.236 no APEX nuclease 1 <UNP Q7SXL6_BRARE> [DANIO
RERIO] GSHMEAPILYEDPPEKLTSKDGRAANMKITSWNVDGLRAWVKKNGLDWVRKEDPDILCLQ
ETKCAEKALPADITAMPEYPHKYWAGSEDKEGYSGVAMLCKTEPLNVTYGIGKEEHDKEG
RVITAEFPDFFLVTAYVPNASRGLVRLDYRKTWDVDFRAYLCGLDARKPLVLCGDLNVAH
QEIDLKNPKGNRKNAGFTPEEREGFTQLLEAGFTDSFRELYPDQAYAYTFWTYMMNARSK
NVGWRLDYFVLSSALLPGLCDSKIRNTAMGSDHCPITLFLAV
>3SQIA 534 XRAY 2.82 0.196 0.251 no KLLA0E03807p <UNP Q6CPM4_KLULA>

[KLUYVEROMYCES LACTIS]
MSKLDSLLKELPTRTAHLYRSIWHKYTEWLKTMPDLTGADLKLFLSQKYIVKYIASHDDI
AKDPLPTCDAMIWFSRALDIENNDVLVLQQRLYGLVKLLEFDYSNVIAILQKISINLWNP
STDSLQSKHFKTCQDKLKLLLDFQWKFNTNVSFEDRTTVSLKDLQCILDDENGKCGLAHS
SKPNFVLVPNFQSPFTCPIFTMAVYYYLRFHGVKKYYKGDGYQILSQLEHIPIIRGKSLD
QYPRELTLGNWYPTIFKYCQLPYTKKHWFQVNQEWPQFPDFSDSSENTSTLAESDSENTI
GIPDFYIEKMNRTKLQPCPQVHVHLFPTDLPPDIQAVFDLLNSVLVTSLPLLYRVFPTHD
IFLDPSLKTPQNIAFLTGTLPLDIESQEHLLAQLIDKTGTVSELVPNPVKIDQNEHTLTP
IGTSLSQTDIPMLDQLKTELQKLIQLQTSTGFSQLITVLLEIFQRLDFKKSNKQFVIDLL
QSCRKDMRNKLMDPCSLSTNFADELSDDENEKGNKTGAIYDPETDNGNEESVSD
>1TZYA 129 XRAY 1.90 0.190 0.222 no Histone H2A-IV <UNP H2A4_CHICK> [GALLUS GALLUS]
MSGRGKQGGKARAKAKSRSSRAGLQFPVGRVHRLLRKGNYAERVGAGAPVYLAAVLEYLT
AEILELAGNAARDNKKTRIIPRHLQLAIRNDEELNKLLGKVTIAQGGVLPNIQAVLLPKK
TDSHKAKAK
>2ZVVA 276 XRAY 2.00 0.232 0.245 no Proliferating cellular nuclear antigen 1 <UNP PCNA1_ARATH> [ARABIDOPSIS THALIANA]
MGSSHHHHHHSSGLVPRGSHMLELRLVQGSLLKKVLESIKDLVNDANFDCSSTGFSLQAM
DSSHVALVSLLLRSEGFEHYRCDRNLSMGMNLGNMSKMLKCAGNDDIITIKADDGGDTVT
FMFESPTQDKIADFEMKLMDIDSEHLGIPDAEYHSIVRMPSNEFSRICKDLSSIGDTVVI
SVTKEGVKFSTAGDIGTANIVLRQNTTVDKPEDAIVIEMKEPVSLSFALRYMNSFTKATP
LSDTVTISLSSELPVVVEYKVAEMGYIRYYLAPKIE
>2XQCA 140 XRAY 1.90 0.176 0.209 no TRANSPOSASE <UNP O83028_DEIRA> [DEINOCOCCUS RADIODURANS]
MTYVILPLEMKKGRGYVYQLEYHLIWCVKYRHQVLVGEVADGLKDILRDIAAQNGLEVIT
MEVMPDHVHLLLSATPQQAIPDFVKALKGASARRMFVAYPQLKEKLWGGNLWNPSYCILT
VSENTRAQIQKYIESQHDKE
>2P5MA 83 XRAY 1.95 0.187 0.224 no Arginine repressor <UNP ARGR_BACSU> [BACILLUS SUBTILIS]
MQRFNPLSKLKRALMDAFVKIDSASHMIVLKTMPGNAQAIGALMDNLDWDEMMGTICGDD
TILIICRTPEDTEGVKNRLLELL
>3OKGA 412 XRAY 1.95 0.199 0.240 no Restriction endonuclease S subunits <UNP Q8R9Q6_THETN> [THERMOANAEROBACTER TENGCONGENSIS]

MSHHHHHHSMDIEFMTEGPYKLPPGWRWVRLGEVCLPTERRDPTKNPSTYFVYVDISAID
STVGKIVSPKEILGQHAPSRARKVIRSGDVIFATTRPYLKNIALVPPDLDGQICSTGFCV
IRANREFAEPEFLFHLCRSDFITNQLTASKMRGTSYPAVTDNDVYNTLIPLPPLEEQRRI
VAKVEALMERVREVRRLRAEAQKDTELLMQTALAEVFPHPGADLPPGWRWVRLGEVCDII
MGQSPPSSTYNFEGNGLPFFQGKADFGDLHPTPRIWCSAPQKVARPGDVLISVRAPVGST
NVANLACCIGRGLAALRPRDSLERFWLLYYLHYLEPELSKMGAGSTFNAITKKDLQNVFI
PLPPLEEQRRIVAYLDQIQQQVAALKRAQAETEAELKRLEQAILDKAFRGDL
>4HD0A 339 XRAY 2.30 0.225 0.277 no DNA double-strand break repair protein Mre11 <UNP MRE11_PYRFU> [PYROCOCCUS FURIOSUS]
HHHHHHMKFAHLADIHLGYEQFHKPQREEEFAEAFKNALEIAVQENVDFILIAGDLFHSS
RPSPGTLKKAIALLQIPKEHSIPVFAIEGNHDRTQRGPSVLNLLEDFGLVYVIGMRKEKV
ENEYLTSERLGNGEYLVKGVYKDLEIHGMKYMSSAWFEANKEILKRLFRPTDNAILMLHQ
GVREVSEARGEDYFEIGLGDLPEGYLYYARGHIHKRYETSYSGSPVVYPGSLERWDFGDY

EVRYEWDGIKFKERYGVNKGFYIVEDFKPRFVEIKVRPFIDVKIKGSEEEIRKAIKRLIP
LIPKNAYVRLNIGWRKPFDLTEIKELLNVEYLKIDTWRI
>2KHQA 110 NMR NA NA NA no Integrase <UNP Q49VW8_STAS1> [STAPHYLOCOCCUS SAPROPHYTICUS SUBSP. SAPROPHYTICUS ATCC 15305]
MITFADYFYQWYEVNKLPHVSESTKRHYESAYKHIKDHFRHKLLKDIKRTEYQKFLNEYG
LTHSYETIRKLNSYIRNAFDDAIHEGYVIKNPTYKAELHASVLEHHHHHH
>1RW2A 152 NMR NA NA NA no ATP-dependent DNA helicase II, 80 kDa subunit <UNP KU86_HUMAN> [HOMO SAPIENS]
MHHHHHHKLKTEQGGAHFSVSSLAEGSVTSVGSVNPAENFRVLVKQKKASFEEASNQLIN
HIEQFLDTNETPYFMKSIDCIRAFREEAIKFSEEQRFNNFLKALQEKVEIKQLNHFWEIV
VQDGITLITKEEASGSSVTAEEAKKFLAPKDK
>3PGGA 121 XRAY 2.14 0.216 0.258 no U6 snRNA-associated Sm-like protein LSm5. SM domain
<UNP Q5CXX3_CRYPV> [CRYPTOSPORIDIUM PARVUM]
SYKVNYMSETPANKSQGGSNQKGGNIILPLALIDKCIGNRIYVVMKGDKEFSGVLRGFDE
YVNMVLDDVQEYGFKADEEDISGGNKKLKRVMVNRLETILLSGNNVAMLVPGGDPDSFNF
S
>2OKFA 140 XRAY 1.60 0.143 0.155 no FdxN element excision controlling factor protein
<UNP Q3M7W6_ANAVT> [ANABAENA VARIABILIS]
GMSARDVFHEVVKTALKKDGWQITDDPLTISVGGVNLSIDLAAQKLIAAERQGQKIAVEV
KSFLKQSSAISEFHTALGQFINYRGALRKVEPDRVLYLAVPLTTYKTFFQLDFPKEIIIE
NQVKMLVYDVEQEVIFQWIN
>3HI2B 101 XRAY 2.00 0.207 0.248 no Motility quorum-sensing regulator mqsR <UNP MQSR_ECOLI> [ESCHERICHIA COLI K-12]
GSHMEKRTPHTRLSQVKKLVNAGQVRTTRSALLNADELGLDFDGMCNVIIGLSESDFYKS
MTTYSDHTIWQDVYRPRLVTGQVYLKITVIHDVLIVSFKEK
>1X51A 155 NMR NA NA NA no A/G-specific adenine DNA glycosylase <UNP MUTYH_HUMAN> [HOMO SAPIENS]
GSSGSSGPRKASRKPPREESSATCVLEQPGALGAQILLVQRPNSGLLAGLWEFPSVTWEP
SEQLQRKALLQELQRWAGPLPATHLRHLGEVVHTFSHIKLTYQVYGLALEGQTPVTTVPP
GARWLTQEEFHTAAVSTAMKKVFRVYQGQSGPSSG
>3MUJA 138 XRAY 1.92 0.171 0.193 no Transcription factor COE3 <UNP COE3_HUMAN> [HOMO SAPIENS]
SMEATPCIKAISPSEGWTTGGATVIIIGDNFFDGLQVVFGTMLVWSELITPHAIRVQTPP
RHIPGVVEVTLSYKSKQFCKGAPGRFVYTALNEPTIDYGFQRLQKVIPRHPGDPERLPKE
VLLKRAADLVEALYGMPH
>2KKPA 117 NMR NA NA NA no Phage integrase <UNP Q2RHJ2_MOOTA> [MOORELLA THERMOACETICA ATCC 39073]
MIEPSKITVEQWLNRWLTDYAKPHLRQSTWESYETVLRLHVIPTLGSIPLKKLQPADIQR
LYASKLESGLSPTRVRYIHVVLHEAMSQARESGLLLQNPTEAAKPPRHPLEHHHHHH
>4HTOA 240 XRAY 2.81 0.210 0.259 no DNA ligase 4 <UNP DNLI4_HUMAN> [HOMO SAPIENS]
MAASQTSQTVASHVPFADLCSTLERIQKSKGRAEKIRHFREFLDSWRKFHDALHKNHKDV
TDSFYPAMRLILPQLERERMAYGIKETMLAKLYIELLNLPRDGKDALKLLNYRTPTGTHG
DAGDFAMIAYFVLKPRCLQKGSLTIQQVNDLLDSIASNNSAKRKDLIKKSLLQLITQSSA
LEQKWLIRMIIKDLKLGVSQQTIFSVFHNDAAELHNVTTDLEKVCRQLHDPSVGLSDISI
>1HLVA 131 XRAY 2.50 0.222 0.260 no MAJOR CENTROMERE AUTOANTIGEN B <UNP

CENPB_HUMAN> [HOMO SAPIENS]
MGPKRRQLTFREKSRIIQEVEENPDLRKGEIARRFNIPPSTLSTILKNKRAILASERKYG
VASTCRKTNKLSPYDKLEGLLIAWFQQIRAAGLPVKGIILKEKALRIAEELGMDDFTASN GWLDRFRRRRS
>1P92A 226 XRAY 2.10 0.243 0.280 no Diphtheria toxin repressor <UNP DTXR_CORDI>
[CORYNEBACTERIUM DIPHTHERIAE]
MKDLVDTTEMYLRTIYELEEEGVTPLRARIAERLEQSGPTVSQTVARMERDGLVVVASDR
SLQMTPTGRTLATAVMRKARLAERLLTDIIGLDINKVHDEACRWEHVMSDEVERRLVKVL
KDVSRSPFGNPIPGLDELGVGNSDAAAPGTRVIDAATSMPRKVRIVQINEIFQVETDQFT
QLLDADIRVGSEVEIVDRDGHITLSHNGKDVELLDDLAHTIRIEEL
>3QU3A 137 XRAY 1.30 0.169 0.200 no Interferon regulatory factor 7 <UNP IRF7_MOUSE>
[MUS MUSCULUS]
GSHMAEVRGVQRVLFGDWLLGEVSSGQYEGLQWLNEARTVFRVPWKHFGRRDLDEEDAQI
FKAWAVARGRWPPSGVNLPPPEAEAAERRERRGWKTNFRCALHSTGRFILRQDNSGDPVD
PHKVYELSRELGSTVGP
>2DA7A 71 NMR NA NA NA no Zinc finger homeobox protein 1b <UNP SIP1_HUMAN> [HOMO
SAPIENS]
GSSGSSGSPINPYKDHMSVLKAYYAMNMEPNSDELLKISIAVGLPQEFVKEWFEQRKVYQ YSNSRSGPSSG
>2F7TA 227 XRAY 2.25 0.193 0.277 no Mos1 transposase <UNP O61446_DROSE>
[DROSOPHILA MAURITIANA]
WVPHELNERQMERRKNTCEILLSRYKRKSFLHRIVTGDEKWIFFVNPKRKKSYVDPGQPA
TSTARPNRFGKKTMLCVWWDQSGVIYYELLKPGETVNAARYQQQLINLNRALQRKRPEYQ
KRQHRVIFLHDNAPSHTARAVRDTLETLNWEVLPHAAYSPDLAPSDYHLFASMGHALAEQ
RFDSYESVKKWLDEWFAAKDDEFYWRGIHKLPERWEKCVASDGKYFE
>2ZQEA 83 XRAY 1.70 0.191 0.227 no MutS2 protein <UNP Q5SHT5_THET8> [THERMUS
THERMOPHILUS]
MREVKEVDLRGLTVAEALLEVDQALEEARALGLSTLRLLHGKGTGALRQAIREALRRDKR
VESFADAPPGEGGHGVTVVALRP
>3S51A 1308 XRAY 3.30 0.259 0.278 no Fanconi anemia group I protein homolog <UNP
FANCI_MOUSE> [MUS MUSCULUS]
MDLKILSLATDKTTDKLQEFLQTLKDDDLASLLQNQAVKGRAVGTLLRAVLKGSPCSEED
GALRRYKIYSCCIQLVESGDLQQDVASEIIGLLMLEVHHFPGPLLVDLASDFVGAVREDR
LVNGKSLELLPIILTALATKKEVLACGKGDLNGEEYKRQLIDTLCSVRWPQRYMIQLTSV
FKDVCLTPEEMNLVVAKVLTMFSKLNLQEIPPLVYQLLVLSSKGSRRSVLDGIIAFFREL
DKQHREEQSSDELSELITAPADELYHVEGTVILHIVFAIKLDCELGRELLKHLKAGQQGD

PSKCLCPFSIALLLSLTRIQRFEEQVFDLLKTSVVKSFKDLQLLQGSKFLQTLVPQRTCV
STMILEVVRNSVHSWDHVTQGLIEFGFILMDSYGPKKILDGKAVEIGTSLSKMTNQHACK
LGANILLETFKIHEMIRQEILEQVLNRVVTRTSSPINHFLDLFSDIIMYAPLILQNCSKV
TETFDYLTFLPLQTVQGLLKAVQPLLKISMSMRDSLILVLRKAMFASQLDARKSAVAGFL
LLLKNFKVLGSLPSSQCTQSIGVTQVRVDVHSRYSAVANETFCLEIIDSLKRSLGQQADI
RLMLYDGFYDVLRRNSQLASSIMQTLFSQLKQFYEPEPDLLPPLKLGACVLTQGSQIFLQ
EPLDHLLSCIQHCLAWYKSRVVPLQQGDEGEEEEEELYSELDDMLESITVRMIKSELEDF
ELDKSADFSQNTNVGIKNNICACLIMGVCEVLMEYNFSISNFSKSKFEEILSLFTCYKKF
SDILSEKAGKGKAKMTSKVSDSLLSLKFVSDLLTALFRDSIQSHEESLSVLRSSGEFMHY
AVNVTLQKIQQLIRTGHVSGPDGQNPDKIFQNLCDITRVLLWRYTSIPTSVEESGKKEKG
KSISLLCLEGLQKTFSVVLQFYQPKVQQFLQALDVMGTEEEEAGVTVTQRASFQIRQFQR
SLLNLLSSEEDDFNSKEALLLIAVLSTLSRLLEPTSPQFVQMLSWTSKICKEYSQEDASF

CKSLMNLFFSLHVLYKSPVTLLRDLSQDIHGQLGDIDQDVEIEKTDHFAVVNLRTAAPTV
CLLVLSQAEKVLEEVDWLIAKIKGSANQETLSDKVTPEDASSQAVPPTLLIEKAIVMQLG
TLVTFFHELVQTALPSGSCVDTLLKGLSKIYSTLTAFVKYYLQVCQSSRGIPNTVEKLVK
LSGSHLTPVCYSFISYVQNKSSDAPKCSEKEKAAVSTTMAKVLRETKPIPNLVFAIEQYE
KFLIQLSKKSKVNLMQHMKLSTSRDFKIKGSVLDMVLREDEEHHHHHH
>2WPOC 112 XRAY 2.67 0.228 0.266 no CHROMOSOMAL REPLICATION INITIATOR PROTEIN
DNAA <UNP DNAA_HELPY> [HELICOBACTER PYLORI]
MDTNNNIEKEILALVKQNPKVSLIEYENYFSQLKYNPNASKSDIAFFYAPNQVLCTTITA
KYGALLKEILSQNKVGMHLAHSVDVRIEVAPKIQINAQSNINYKAIKTSVKD
>2DQRA 122 XRAY 3.01 0.205 0.276 no Replication termination protein <UNP RTP_BACSU>
[BACILLUS SUBTILIS]
MKEEKRSSTGFLVKQRAFLKLYMITMTEQERLYGLKLLKVLQSEFKEIGFKPNHTEVYRS
LHELLDDGILKQIKVKKEGAKLQEVVLYQFKDYEAAKLYKKQLKVELDRCKKLIEKALSD
NF

Negative samples:

>2OKMA 147 XRAY 1.65 0.198 0.216 no Collagen adhesin <UNP Q9EU70_ENTFA> [ENTEROCOCCUS FAECALIS]
ERDYPFFYKVGDLAGESNQVRWFLNVNLNKSDVTEDISIADRQGSGQQLNKESFTFDIVN
DKETKYISLAEFEQQGYGKIDFVTDNDFNLRFYRDKARFTSFIVRYTSTITEAGQHQATF ENSYDINYQLNNQDATNEKNTSQVKNV
>1M48A 133 XRAY 1.95 0.200 0.269 no interleukin-2 <UNP IL2_HUMAN> [HOMO SAPIENS]
APTSSSTKKTQLQLEHLLLDLQMILNGINNYKNPKLTRMLTFKFYMPKKATELKHLQCLE
EELKPLEEVLNLAQSKNFHLRPRDLISNINVIVLELKGSETTFMCEYADETATIVEFLNR
WITFCQSIISTLT
>1F5NA 592 XRAY 1.70 0.224 0.255 no INTERFERON-INDUCED GUANYLATE-BINDING PROTEIN 1 <UNP GBP1_HUMAN>
[HOMO SAPIENS]
MASEIHMTGPMCLIENTNGRLMANPEALKILSAITQPMVVVAIVGLYRTGKSYLMNKLAG
KKKGFSLGSTVQSHTKGIWMWCVPHPKKPGHILVLLDTEGLGDVEKGDNQNDSWIFALAV
LLSSTFVYNSIGTINQQAMDQLYYVTELTHRIRSKSSPDENENEVEDSADFVSFFPDFVW
TLRDFSLDLEADGQPLTPDEYLTYSLKLKKGTSQKDETFNLPRLCIRKFFPKKKCFVFDR
PVHRRKLAQLEKLQDEELDPEFVQQVADFCSYIFSNSKTKTLSGGIQVNGPRLESLVLTY
VNAISSGDLPCMENAVLALAQIENSAAVQKAIAHYEQQMGQKVQLPTESLQELLDLHRDS
EREAIEVFIRSSFKDVDHLFQKELAAQLEKKRDDFCKQNQEASSDRCSGLLQVIFSPLEE
EVKAGIYSKPGGYRLFVQKLQDLKKKYYEEPRKGIQAEEILQTYLKSKESMTDAILQTDQ
TLTEKEKEIEVERVKAESAQASAKMLHEMQRKNEQMMEQKERSYQEHLKQLTEKMENDRV
QLLKEQERTLALKLQEQEQLLKEGFQKESRIMKNEIQDLQTKMRRRKACTIS
>2D42A 249 XRAY 2.07 0.230 0.265 no non-toxic crystal protein <PDB 2D42> [BACILLUS THURINGIENSIS]
AIINLLRELEIYGMQYANSHQYTYGSSYSDDTNPIRIAGLDARIPDPIVTDPVNHIVLDR
RIITNTTSNSLEGVFSFSNAYTSRTSSQTRDGVTAGTNITGKYFANLFFEQVGLSGRIAF
EGAVTNENKYTLDATQDFRDSQTIRVPPFHRATGVYTLEQGAFEKMTVLECVVSGNGIIR
YYRTLPDNSYTEIVQRVNIIDVLQANGTPGFTISKEQNRAYFTGEGTISGQIGLQTFIDV
VIEPLPGHA
>1JUVA 193 XRAY 1.70 0.248 0.269 no DIHYDROFOLATE REDUCTASE <UNP DYR_BPT4> [ENTEROBACTERIA PHAGE
T4]
MIKLVFRYSPTKTVDGFNELAFGLGDGLPWGRVKKDLQNFKARTEGTIMIMGAKTFQSLP
TLLPGRSHIVVCDLARDYPVTKDGDLAHFYITWEQYITYISGGEIQVSSPNAPFETMLDQ

NSKVSVIGGPALLYAALPYADEVVVSRIVKRHRVNSTVQLDASFLDDISKREMVETHWYK
IDEVTTLTESVYK
>1PU6A 218 XRAY 1.64 0.153 0.185 no 3-METHYLADENINE DNA GLYCOSYLASE <UNP O25323_HELPY> [HELICOBACTER PYLORI]
VLDSFEILKALKSLDLLKNAPAWWWPNALKFEALLGAVLTQNTKFEAVLKSLENLKNAFI
LENDDEINLKKIAYIEFSKLAECVRPSGFYNQKAKRLIDLSGNILKDFQSFENFKQEVTR
EWLLDQKGIGKESADAILCYACAKEVMVVDKYSYLFLKKLGIEIEDYDELQHFFEKGVQE
NLNSALALYENTISLAQLYARFHGKIVEFSKQKLELKL
>2QKLA 127 XRAY 2.33 0.229 0.259 no SPBC3B9.21 protein <UNP Q9P805_SCHPO> [SCHIZOSACCHAROMYCES POMBE]
MEDENILRNAVNLQVLKFHYPEIESIIDIASHVAVYQFDVGSQKWLKTSIEGTFFLVKDQ
RARVGYVILNRNSPENLYLFINHPSNVHLVDRYLIHRTENQHVVGLWMFDPNDMSRIFNI VKESLLR
>3I96A 119 XRAY 1.65 0.163 0.185 no Ethanolamine utilization protein eutS <UNP EUTS_ECOLI> [ESCHERICHIA COLI]
MDKERIIQEFVPGKQVTLAHLIAHPGEELAKKIGVPDAGAIGIMTLTPGETAMIAGDLAL
KAADVHIGFLDRFSGALVIYGSVGAVEEALSQTVSGLGRLLNYTLCEMTKSLEHHHHHH
>1LLFA 534 XRAY 1.40 0.142 0.169 no Lipase 3 <UNP LIP3_CANRU> [CANDIDA CYLINDRACEA]
APTAKLANGDTITGLNAIINEAFLGIPFAEPPVGNLRFKDPVPYSGSLNGQKFTSYGPSC
MQQNPEGTFEENLGKTALDLVMQSKVFQAVLPQSEDCLTINVVRPPGTKAGANLPVMLWI
FGGGFEIGSPTIFPPAQMVTKSVLMGKPIIHVAVNYRVASWGFLAGDDIKAEGSGNAGLK
DQRLGMQWVADNIAGFGGDPSKVTIFGESAGSMSVLCHLIWNDGDNTYKGKPLFRAGIMQ
SGAMVPSDPVDGTYGNEIYDLFVSSAGCGSASDKLACLRSASSDTLLDATNNTPGFLAYS
SLRLSYLPRPDGKNITDDMYKLVRDGKYASVPVIIGDQNDEGTIFGLSSLNVTTNAQARA
YFKQSFIHASDAEIDTLMAAYPQDITQGSPFDTGIFNAITPQFKRISAVLGDLAFIHARR
YFLNHFQGGTKYSFLSKQLSGLPIMGTFHANDIVWQDYLLGSGSVIYNNAFIAFATDLDP
NTAGLLVNWPKYTSSSQSGNNLMMINALGLYTGKDNFRTAGYDALMTNPSSFFV
>2CI1A 276 XRAY 1.08 0.112 0.141 no NG, NG-DIMETHYLARGININE DIMETHYLAMINOHYDROLASE 1 <UNP DDAH1_BOVIN> [BOS TAURUS]
ATFGRATHVVVRALPESLAQQALRRTKGDEVDFARAERQHQLYVGVLGSKLGLQVVQLPA
DESLPDCVFVEDVAVVCEETALITRPGAPSRRKEADMMKEALEKLQLNIVEMKDENATLD
GGDVLFTGREFFVGLSKRTNQRGAEILADTFKDYAVSTVPVVDALHLKSFCSMAGPNLIA
IGSSESAQKALKIMQQMSDHRYDKLTVPDDTAANCIYLNIPSKGHVLLHRTPEEYPESAK
VVYEKLKDHMLIPVSNSELEKVDGLLTXXSSVLINKK
>1L3LA 234 XRAY 1.66 0.246 0.266 no Transcriptional activator protein traR <UNP TRAR_9RHIZ> [AGROBACTERIUM TUMEFACIENS]
MQHWLDKLTDLAAIEGDECILKTGLADIADHFGFTGYAYLHIQHRHITAVTNYHRQWQST
YFDKKFEALDPVVKRARSRKHIFTWSGEHERPTLSKDERAFYDHASDFGIRSGITIPIKT
ANGFMSMFTMASDKPVIDLDREIDAVAAAATIGQIHARISFLRTTPTAEDAAWLDPKEAT
YLRWIAVGKTMEEIADVEGVKYNSVRVKLREAMKRFDVRSKAHLTALAIRRKLI
>2FFYA 358 XRAY 1.07 0.133 0.164 no Beta-lactamase <UNP AMPC_ECOLI> [ESCHERICHIA COLI]
APQQINDIVHRTITPLIEQQKIPGMAVAVIYQGKPYYFTWGYADIAKKQPVTQQTLFELG
SVSKTFTGVLGGDAIARGEIKLSDPTTKYWPELTAKQWNGITLLHLATYTAGGLPLQVPD
EVKSSSDLLRFYQNWQPAWAPGTQRLYANSSIGLFGALAVKPSGLSFEQAMQTRVFQPLK
LNHTWINVPPAEEKNYAWGYREGKAVHVSPGALDAEAYGVKSTIEDMARWVQSNLKPLDI
NEKTLQQGIQLAQSRYWQTGDMYQGLGWEMLDWPVNPDSIINGSDAKIALAARPVKAITP
PTPAVRASWVHKTGATGGFGSYVAFIPEKELGIVMLANKNYPNPARVDAAWQILNALQ
>3QX1A 84 XRAY 1.60 0.206 0.218 no FAS-associated factor 1 <UNP FAF1_HUMAN> [HOMO SAPIENS]

GSHMEPVSKLRIRTPSGEFLERRFLASNKLQIVFDFVASKGFPWDEYKLLSTFPRRDVTQ
LDPNKSLLEVKLFPQETLFLEAKE
>3C1QA 123 XRAY 1.70 0.158 0.208 no General secretion pathway protein F <UNP GSPF_VIBCH> [VIBRIO CHOLERAE]
MGFAFKRGISTPDLALITRQLATLVQSGMPLEECLRAVAEQSEKPRIRTMLVAVRAKVTE
GYTLSDSLGDYPHVFDELFRSMVAAGEKSGHLDSVLERLADYAENRQKMRSKLQQASENL
YFQ
>3HYNA 189 XRAY 1.20 0.118 0.139 no Putative signal transduction protein <PDB 3HYN> [EUBACTERIUM RECTALE ATCC 33656]
GMSYQNANYSAFYVSEPFSESNLGANSTHDFVYYNMLRMWKGEDNSFPFNDAHDKTYNVR
DGSDWEKTLKPRLHTRLDNSKNIILFLSSITANSRALREEMNYGIGTKGLPVIVIYPDYD
KKSDIVDSNGNFKKQIKDLWDKLPAFRDNMSSVATLHIPCTKSVIISALNNEDFMVNTMA
DAEKYYYKP
>3FOKA 307 XRAY 2.50 0.225 0.254 no uncharacterized protein Cgl0159 <UNP Q8NTZ2_CORGL>
[CORYNEBACTERIUM GLUTAMICUM]
MTPPIISPESFEALRRMRAAEPTMVAERFKQRRKRELLGEDGKLFIVAADHPARGALAVG
DNETAMANRYELLERMAIALSRPGVDGVLGTPDIIDDLAALGLLDDKIVVGSMNRGGLRG
ASFEMDDRYTGYNVSSMVDRGVDFAKTLVRINLSDAGTAPTLEATAHAVNEAAAAQLPIM
LEPFMSNWVNGKVVNDLSTDAVIQSVAIAAGLGNDSSYTWMKLPVVEEMERVMESTTMPT
LLLGGEGGNDPDATFASWEHALTLPGVRGLTVGRTLLYPQDGDVAAAVDTAARLVHTDIQ
QFTSQSI
>1LR5A 163 XRAY 1.90 0.200 0.241 no Auxin binding protein 1 <UNP ABP1_MAIZE> [ZEA MAYS]
SCVRDNSLVRDISQMPQSSYGIEGLSHITVAGALNHGMKEVEVWLQTISPGQRTPIHRHS
CEEVFTVLKGKGTLLMGSSSLKYPGQPQEIPFFQNTTFSIPVNDPHQVWNSDEHEDLQVL
VIISRPPAKIFLYDDWSMPHTAAVLKFPFVWDEDCFEAAKEQL
>1KOEA 172 XRAY 1.50 0.190 0.224 no ENDOSTATIN <UNP COIA1_MOUSE> [MUS MUSCULUS]
QPVLHLVALNTPLSGGMRGIRGADFQCFQQARAVGLSGTFRAFLSSRLQDLYSIVRRADR
GSVPIVNLKDEVLSPSWDSLFSGSQGQLQPGARIFSFDGRDVLRHPAWPQKSVWHGSDPS
GRRLMESYCETWRTETTGATGQASSLLSGRLLEQKAASCHNSYIVLCIENSF
>3EAGA 326 XRAY 2.55 0.186 0.243 no UDP-N-acetylmuramate:L-alanyl-gamma-D-glutamyl-meso-diaminopimelate ligase <UNP Q9JRY9_NEIMB> [NEISSERIA MENINGITIDIS MC58]
SNAMKHIHIIGIGGTFMGGLAAIAKEAGFEVSGCDAKMYPPMSTQLEALGIDVYEGFDAA
QLDEFKADVYVIGNVAKRGMDVVEAILNLGLPYISGPQWLSENVLHHHWVLGVAGTHGKT
TTASMLAWVLEYAGLAPGFLIGGVPENFGVSARLPQTPRQDPNSQSPFFVIEADEYDTAF
FDKRSKFVHYRPRTAVLNNLEFDHADIFADLGAIQTQFHYLVRTVPSEGLIVCNGRQQSL
QDTLDKGCWTPVEKFGTEHGWQAGEANADGSFDVLLDGKTAGRVKWDLMGRHNRMNALAV
IAAARHVGVDIQTACEALGAFKNVKR
>1T3YA 141 XRAY 1.15 0.119 0.159 no Coactosin-like protein <UNP COTL1_HUMAN> [HOMO SAPIENS]
ATKIDKEACRAAYNLVRDDGSAVIWVTFKYDGSTIVPGEQGAEYQHFIQQCTDDVRLFAF
VRFTTGDAMSKRSKFALITWIGENVSGLQRAKTGTDKTLVKEVVQNFAKEFVISDRKELE
EDFIKSELKKAGGANYDAQTE
>3L9VA 189 XRAY 2.15 0.215 0.262 no Putative thiol-disulfide isomerase or thioredoxin <PDB 3L9V>
[SALMONELLA ENTERICA SUBSP. ENTERICA SEROVAR TYPHIMURIUM]
SNAEWESITPPVVDAPAVVEFFSFYCPPCYAFSQTMGVDQAIRHVLPQGSRMVKYHVSLL
GPLGHELTRAWALAMVMKETDVIEKAFFTAGMVEKRLHSPDDVRRVFMSATGISRGEYDR
SIKSPAVNDMVALQERLFKEYGVRGTPSVYVRGRYHINNAAFGAFSVENFRSRYAAVVRK
LLAGNPDAD
>1SW6A 327 XRAY 2.10 0.222 0.251 no REGULATORY PROTEIN SWI6 <UNP SWI6_YEAST> [SACCHAROMYCES

CEREVISIAE]
NDDINKGPSGDNENNGTDDNDRTAGPIITFTHDLTSDFLSSPLKIMKALPSPVVNDNEQK
MKLEAFLQRLLFPEIQEMPTSLNNDSSNRNSEGGSSNQQQQHVSFDSLLQEVNDAFPNTQ
LNLNIPVDEHGNTPLHWLTSIANLELVKHLVKHGSNRLYGDNMGESCLVKAVKSVNNYDS
GTFEALLDYLYPCLILEDSMNRTILHHIIITSGMTGCSAAAKYYLDILMGWIVKKQNRPI
QSGTNEKESKPNDKNGERKDSILENLDLKWIIANMLNAQDSNGDTCLNIAARLGNISIVD
ALLDYGADPFIANKSGLRPVDFGAGLE
>3CKCA 527 XRAY 1.50 0.192 0.210 no SusD <UNP Q8A1G2_BACTN> [BACTEROIDES THETAIOTAOMICRON]
GINDLDISPIDPQTGGSFDQQGVFVKGYAMLGVTGQKGIDGSPDLDGQDEGESGFYRTTF
NCNELPTDECLWAWQKNQDIPQLTSISWSPSSQRTEWVYVRLGYDITQYNFFLDQTEGMT
DAETLRQRAEIRFLRALHYWYFLDLFGKAPFKEHFSNDLPVEKKGTELYTYIQNELNEIE
ADMYEPRQAPFGRADKAANWLLRARLYLNAGVYTGQTDYAKAEEYASKVIGSAYKLCTNY
SELFMADNDENENAMQEIILPIRQDGVKTRNYGGSTYLVCGTRVAGMPRMGTTNGWSCIF
ARAAMVQKFFSNLEDVPMLPADVEIPTKGLDTDEQIDAFDAEHGIRTEDMIKAAGDDRAL
LYSGVGGGRRKIQTDAISGFTDGLSIVKWQNYRSDGKPVSHATYPDTDIPLFRLAEAYLT
RAEAIFRQGGDATGDINELRKRANCTRKVQTVTEQELIDEWAREFYLEGRRRSDLVRFGM
FTTNKYLWDWKGGAMNGTSVASYYNKYPIPVSDINNNRNMSQNEGYK
>4IVKA 434 XRAY 1.80 0.162 0.182 no Carboxylesterases <UNP K4HQE7_9BACT> [UNCULTURED BACTERIUM]
MKTSAKFLSFAVSFVLLIIASTSFAEGPVTATKPKEAGFTSEGLARIDAYLKNEIQAKTM
PGAVMMIKRNGETAYFSSFGLRDPDTKEPMTAETIFRIYSMSKPITTVAAMMLVEEGKLQ
LDEPVSKYIPSFANVKVGVETKGENGMALETGPVKRAITIQDLMRHTSGITYGFVGDGLV
KKAYIASNLFDGDFDNAEFAERIAKLPLVYQPGTTWDYGHSTDILGRVVEVVSGKSLYQF
EKERLLDPLGMKDTGFYVTDPAKKSLVAEAMPNDRKIGGSEMFDPRVQKKWEPGGQGMVS
TIGDYARFTQMVLNGGTLDGKRYLSPKTIAYMGSNHIPQASGIVPGAYYLPGPGVGFGLG
FAVRTEAGVTPVEGSVGDLSWGGAGGTVFWIDPKENLTVVFMAPMVSPRARVWRTLRNIV
YGAFDRLEHHHHHH
>3F4AA 169 XRAY 1.80 0.168 0.211 no Uncharacterized protein YGR203W <UNP YG4E_YEAST>
[SACCHAROMYCES CEREVISIAE]
MGSSHHHHHHSSGRENLYFQGMDSYSITNVKYLDPTELHRWMQEGHTTTLREPFQVVDVR
GSDYMGGHIKDGWHYAYSRLKQDPEYLRELKHRLLEKQADGRGALNVIFHCMLSQQRGPS
AAMLLLRSLDTAELSRCRLWVLRGGFSRWQSVYGDDESVTAGYLPDLWR
>2PJUA 225 XRAY 2.10 0.204 0.252 no Propionate catabolism operon regulatory protein
<UNP PRPR_ECOLI> [ESCHERICHIA COLI]
MSLAHPPRLNDDKPVIWTVSVTRLFELFRDISLEFDHLANITPIQLGFEKAVTYIRKKLA
NERCDAIIAAGSNGAYLKSRLSVPVILIKPSGYDVLQFLAKAGKLTSSIGVVTYQETIPA
LVAFQKTFNLRLDQRSYITEEDARGQINELKANGTEAVVGAGLITDLAEEAGMTGIFIYS
AATVRQAFSDALDMTRMSLRHNTHDATRNALRTRYVLEGHHHHHH
>4BOUA 160 XRAY 1.55 0.177 0.229 no OTU DOMAIN-CONTAINING PROTEIN 3 <UNP OTUD3_HUMAN> [HOMO SAPIENS]
GPEFVSFANQLQALGLKLREVPGDGNCLFRALGDQLEGHSRNHLKHRQETVDYMIKQRED
FEPFVEDDIPFEKHVASLAKPGTFAGNDAIVAFARNHQLNVVIHQLNAPLWQIRGTEKSS
VRELHIAYRYGEHYDSVRRINDNSEAPAHLQTDFQMLHQD
>3PE6A 303 XRAY 1.35 0.115 0.147 no Monoglyceride lipase <UNP Q6IBG9_HUMAN> [HOMO SAPIENS]
MPEESSPRRTPQSIPYQDLPHLVNADGQYLFCRYWAPTGTPKALIFVSHGAGEHSGRYEE
LARMLMGLDLLVFAHDHVGHGQSEGERMVVSDFHVFVRDVLQHVDSMQKDYPGLPVFLLG
HSMGGAIAILTAAERPGHFAGMVLISPLVLANPESATTFKVLAAKVLNSVLPNLSSGPID
SSVLSRNKTEVDIYNSDPLICRAGLKVCFGIQLLNAVSRVERALPKLTVPFLLLQGSADR
LCDSKGAYLLMELAKSQDKTLKIYEGAYHVLHKELPEVTNSVFHEINMWVSQRTATAGTA

SPP
>3NKZA 123 XRAY 2.11 0.158 0.192 no Flagellar protein fliT <UNP FLIT_YERE8> [YERSINIA ENTEROCOLITICA SUBSP. ENTEROCOLITICA] SNAMERHQHLLSEYQQILTLSEQMLVLATEGNWDALVDLEMTYLKAVESTANITISSCSS
LMLQDLLREKLRAILDNEIEIKRLLQLRLDRLSDLVGQSTKQQAVNNTYGQFPDHALLLG
ETQ
>3M62A 968 XRAY 2.40 0.203 0.257 no Ubiquitin conjugation factor E4 <UNP UFD2_YEAST> [SACCHAROMYCES CEREVISIAE]
GSPEFRSMTAIEDILQITTDPSDTRGYSLLKSEEVPQGSTLGVDFIDTLLLYQLTENEKL
DKPFEYLNDCFRRNQQQKRITKNKPNAESLHSTFQEIDRLVIGYGVVALQIENFCMNGAF
INYITGIVSNVNSYTDFLSQIIQRAILEGTALDLLNAVFPTLLEYCNKHVSHFDLNESVI
YNNVLTIFELFVTFKPIAEIFTKIDGFFADYSCKPQDFERKTILGPILSLSPIEAAVAIR
NYGDNLLRSKQQTAMIHESLQAEHKVVIDRLFFIVDKLVRGSLNSRTDMISYFAHIANKN
HLRRADHPPFKELSSNGFMSNITLLLVRFSQPFLDISYKKIDKIDANYFNNPSLFIDLSG
ETRLNSDFKEADAFYDKNRKTADSKPNFISDCFFLTLTYLHYGLGGTLSFEEKMGSEIKA
LKEEIEKVKKIAANHDVFARFITAQLSKMEKALKTTESLRFALQGFFAHRSLQLEVFDFI
CGASTFLIRVVDPEHEFPFKQIKLPLIPDQIGVENVDNADFLRAHAPVPFKYYPEFVVEG
PVNYSLYISKYQTSPIFRNPRLGSFVEFTTMVLRCPELVSNPHLKGKLVQLLSVGAMPLT
DNSPGFMMDIFEHDELVNKNLLYALLDFYVIVEKTGSSSQFYDKFNSRYSISIILEELYY
KIPSYKNQLIWQSQNNADFFVRFVARMLNDLTFLLDEGLSNLAEVHNIQNELDNRARGAP
PTREEEDKELQTRLASASRQAKSSCGLADKSMKLFEIYSKDIPAAFVTPEIVYRLASMLN
YNLESLVGPKCGELKVKDPQSYSFNPKDLLKALTTVYINLSEQSEFISAVAKDERSFNRN
LFVRAVDILGRKTGLASPEFIEKLLNFANKAEEQRKADEEEDLEYGDVPDEFLDPLMYTI
MKDPVILPASKMNIDRSTIKAHLLSDSTDPFNRMPLKLEDVTPNEELRQKILCFKKQKKE
EAKHKASE
>1WDJA 187 XRAY 2.00 0.189 0.241 no hypothetical protein TT1808 <UNP Q5SI60_THET8> [THERMUS THERMOPHILUS] MPLVLDLARPVSEEELRRLSELNPGYQWERSPEGRLWVSPTGGESGRRSLQLAYQLARWN
EERGLGVVFDSSTGFKFPDGSILSPDAAFVERGAWEALSEAEREGFPPLAPKAVFEVRSA
SQDPEELRAKMGIYLRNGVLLGVLVDPYARAVEVFRPGKPPLRLEGVERVSLDPELPGFA
LSLPPLW
>2ZUVA 759 XRAY 1.85 0.151 0.192 no Lacto-N-biose phosphorylase <UNP Q5NU17_BIFLO> [BIFIDOBACTERIUM LONGUM]
MTSTGRFTLPSEENFAEKTKELAELWGADAIRNSDGTHLDEAVLALGKKIYNAYFPTRAH
NEWITLHMDETPQVYLLTDRILAESDTVDIPLMESFFAEQLKPNRDADPHKYWEVVDRTT
GEVVDSANWTLDADEDTVHVSGVAAWHEYTVSFLAYIIWDPVEMYNHLTNDWGDKEHEIP
FDIYHPATRKFVFDTFEQWLKDSPQTDVVRFTTFFYQFTLLFDEKRREKVVDWFGCACTV
SPRALDDFEAKYGYRLRPEDFVDGGAYNSAWRVPRKAQRDWIDFLSGFVRENVKQLADMS
HAAGKEAMMFLGDQWIGTEPYKDGFDELGLDAVVGSIGDGTTTRMIADIPGVKYTEGRFL
PYFFPDTFYEGNDPSIEGLDNWRKARRAILRSPISRMGYGGYLSLAAKFPKFVDTVTHIA
NEFRDIHDRTGGVAAEGELNVAILNSWGKMRSWMAFTVAHALPNKQTYSYYGILESLSGM
RVNVRFISFDDVLAHGIDSDIDVIINGGPVDTAFTGGDVWTNPKLVETVRAWVRGGGAFV
GVGEPSSAPRFQTGRFFQLADVIGVDEERYQTLSVDKYFPPVVPDHFITADVPVDPAARE
AWEQAGYRIPLSGCGGGQSIKPLGGIDFGEPVLNTYPVNENVTLLRADGGQVQLATNDYG
KGRGVVYISGLPYSAANARLLERVLFYASHNEDKYAAWSSSNPECEVAHFPEQGLYCVINN
TDQPQKTTVTLADGTTEDFDLPDSGIAWREALEHHHHHH
>3SKQA 249 XRAY 2.10 0.196 0.227 no Mitochondrial distribution and morphology protein 38 <UNP MDM38_YEAST> [SACCHAROMYCES CEREVISIAE]
KLFPNLLPSTYESGKDKQAKRNKLIEIRKKTSEFLHETLEESNLITYNTIENAEKKQKFL
NFFRKLYSAKEGKIMTFQHDEISAIAQMFKNDSVLDNLSRPQLAAMSKFMSLRPFGNDNM

LRYQIRSKLKDIMNDDKTIDYEGVESLSQEELYQACVSRGMKAYGVSKEDLVDNLKVWLE
LRLRQKIPSVLMVLSSTFTFGGLPKENYSKAFSPLAEKKETKSKYDDLLDLYYDGILQVL
SSIPDPVYN
>3C37A 253 XRAY 1.70 0.221 0.254 no Peptidase, M48 family <UNP Q74D82_GEOSL> [GEOBACTER SULFURREDUCENS PCA] MATSMTDIKGFNMISIEQEKELGNKFAVEIEKQQQPVNDPEVQRYVDKVGKRLLSGARAV
EFDYVFKVVKDDSVNAFAIPGGRVYVHTGLLKAADNETELAGVLAHEINHAVARHGTRQM
TQEYGYSLVLSLVLGDNPNMLAQLAGQLFGKAGMMSYSREYENQADFLGVETMYKAGYNP
NGLTSFFQKLNAMDGGTQSNVARFFSTHPLTSERIQRVQAEIAKLPPQRYLTDETEFKKI
KGRLKLEHHHHHH
>4G0RA 735 XRAY 2.70 0.216 0.217 no Capsid protein VP1 <UNP CAPSD_PAVHH> [H-1 PARVOVIRUS]
MAPPAKRAKRGWVPPGYKYLGPGNSLDQGEPTNPSDAAAKEHDEAYDQYIKSGKNPYLYF
SPADQRFIDQTKDAKDWGGKVGHYFFRTKRAFAPKLSTDSEPGTSGVSRPGKRTKPPAHI
FVNQARAKKKRASLAAQQRTLTMSDGTETNQPDTGIANARVERSADGGGSSGGGGSGGGG
IGVSTGTYDNQTTYKFLGDGWVEITAHASRLLHLGMPPSENYCRVTVHNNQTTGHGTKVK
GNMAYDDTHQQIWTPWSLVDANAWGVWFQPSDWQFIQNSMESLNLDSLSQELFNVVVKTV
TEQQGAGQDAIKVYNNDLTACMMVALDSNNILPYTPAAQTSETLGFYPWKPTAPAPYRYY
FFMPRQLSVTSSNSAEGTQITDTIGEPQALNSQFFTIENTLPITLLRTGDEFTTGTYIFN
TDPLKLTHTWQTNRHLGMPPRITDLPTSDTATASLTANGDRFGSTQTQNVNYVTEALRTR
PAQIGFMQPHDNFEANRGGPFKVPVVPLDITAGEDHDANGAIRFNYGKQHGEDWAKQGAA
PERYTWDAIDSAAGRDTARCFVQSAPISIPPNQNQILQREDAIAGRTNMHYTNVFNSYGP
LSAFPHPDPIYPNGQIWDKELDLEHKPRLHVTAPFVCKNNPPGQLFVRLGPNLTDQFDPN
STTVSRIVTYSTFYWKGILKFKAKLRPNLTWNPVYQATTDSVANSYMNVKKWLPSATGNM
HSDPLICRPVPHMTY
>3S8IA 148 XRAY 1.70 0.177 0.199 no Protein DDI1 homolog 1 <UNP DDI1_HUMAN> [HOMO SAPIENS]
MGSSHHHHHHSSGLVPRGSGQVTMLYINCKVNGHPLKAFVDSGAQMTIMSQACAERCNIM
RLVDRRWAGVAKGVGTQRIIGRVHLAQIQIEGDFLQCSFSILEDQPMDMLLGLDMLRRHQ CSIDLKKNVLVIGTTGTQTYFLPEGELP
>3FSAA 125 XRAY 0.98 0.121 0.135 no Azurin <UNP AZUR_PSEAE> [PSEUDOMONAS AERUGINOSA]
AECSVDIQGNDQMQFNTNAITVDKSCKQFTVNLSHPGNLPKNVMGHNWVLSTAADMQGVV
TDGMASGLDKDYLKPDDSRVIAHTKLIGSGEKDSVTFDVSKLKEGEQYMFFCAAHAAMKG
TLTLK
>1DGWA 178 XRAY 1.70 0.196 0.252 no CANAVALIN <UNP CANA_CANEN> [CANAVALIA ENSIFORMIS]
NNPYLFRSNKFLTLFKNQHGSLRLLQRFNEDTEKLENLRDYRVLEYCSKPNTLLLPHHSD
SDLLVLVLEGQAILVLVNPDGRDTYKLDQGDAIKIQAGTPFYLINPDNNQNLRILKFAIT
FRRPGTVEDFFLSSTKRLPSYLSAFSKNFLEASYDSPYDEIEQTLLQEEQEGVIVKMP
>4DCMA 375 XRAY 2.30 0.203 0.252 no Ribosomal RNA large subunit methyltransferase G <UNP RLMG_ECOLI> [ESCHERICHIA COLI]
HMRSLTLQRFPATDDVNPLQAWEAADEYLLQQLDDTEIRGPVLILNDAFGALSCALAEHK
PYSIGDSYISELATRENLRLNGIDESSVKFLDSTADYPQQPGVVLIKVPKTLALLEQQLR
ALRKVVTSDTRIIAGAKARDIHTSTLELFEKVLGPTTTTLAWKKARLINCTFNEPQLADA
PQTVSWKLEGTDWTIHNHANVFSRTGLDIGARFFMQHLPENLEGEIVDLGCGNGVIGLTL
LDKNPQAKVVFVDESPMAVASSRLNVETNMPEALDRCEFMINNALSGVEPFRFNAVLCNP
PFHQQHALTDNVAWEMFHHARRCLKINGELYIVANRHLDYFHKLKKIFGNCTTIATNNKF
VVLKAVKLEHHHHHH
>2Q4KA 251 XRAY 2.50 0.166 0.236 no Uncharacterized protein C11orf68 <UNP CK068_HUMAN> [HOMO SAPIENS]
MEPGEELEEEGSPGGREDGFTAEHLAAEAMAADMDPWLVFDARTTPATELDAWLAKYPPS
QVTRYGDPGSPNSEPVGWIAVYGQGYSPNSGDVQGLQAAWEALQTSGRPITPGTLRQLAI

THHVLSGKWLMHLAPGFKLDHAWAGIARAVVEGRLQVAKVSPRAKEGGRQVICVYTDDFT
DRLGVLEADSAIRAAGIKCLLTYKPDVYTYLGIYRANRWHLCPTLYESRFQLGGSARGSR
VLDRANNVELT
>3JZLA 409 XRAY 1.91 0.211 0.241 no Putative cystathionine beta-lyase involved in aluminum resistance <UNP Q720C5_LISMF> [LISTERIA MONOCYTOGENES STR. 4B]
GMNNIQAIRKKVETQIDDLQNKTDEIAEFNQAKVLDAFQENKVSDFHFHPSTGYGYDDEG
RDTLERVYATVFKTEAALVRPQIISGTHAISTVLFGILRPDDELLYITGQPYDTLEEIVG
IRKQGQGSLKDFHIGYSSVPLLENGDVDFPRIAKKMTPKTKMIGIQRSRGYADRPSFTIE
KIKEMIVFVKNINPEVIVFVDNCYGEFVEYQEPPEVGADIIAGSLIKNPGGGLAKTGGYI
AGKEALVDLCGYRLTTPGIGREAGASLYSLLEMYQGFFLAPHVTAQAIKGARFTAAMLAE
FGVEADPVWDAPRTDLIQSVSFHNKEKMVAFAQAIQAASPVNAHVLPIGAYMPGYEDDVI
MAAGTFIQGASLELTADGPIREPYQLYVQGGLTYEHIKIAVTRAIQKIV
>1QWRA 319 XRAY 1.80 0.199 0.232 no Mannose-6-phosphate isomerase <UNP MANA_BACSU> [BACILLUS SUBTILIS] SNAMTQSPIFLTPVFKEKIWGGTALRDRFGYSIPSESTGECWAISAHPKGPSTVANGPYK
GKTLIELWEEHREVFGGVEGDRFPLLTKLLDVKEDTSIKVHPDDYYAGENEEGELGKTEC
WYIIDCKENAEIIYGHTARSKTELVTMINSGDWEGLLRRIKIKPGDFYYVPSGTLHALCK
GALVLETQQNSDATYRVYDYDRLDSNGSPRELHFAKAVNAATVPHVDGYIDESTESRKGI
TIKTFVQGEYFSVYKWDINGEAEMAQDESFLICSVIEGSGLLKYEDKTCPLKKGDHFILP
AQMPDFTIKGTCTLIVSHI
>1GP0A 143 XRAY 1.40 0.209 0.237 no CATION-INDEPENDENT MANNOSE-6-PHOSPHATE RECEPTOR <UNP MPRI_HUMAN> [HOMO SAPIENS]
MKSNEHDDCQVTNPSTGHLFDLSSLSGRAGFTAAYSEKGLVYMSICGENENCPPGVGACF
GQTRISVGKANKRLRYVDQVLQLVYKDGSPCPSKSGLSYKSVISFVCRPEAGPTNRPMLI
SLDKQTCTLFFSWHTPLACEQAT
>1QD6C 240 XRAY 2.10 0.226 0.283 no OUTER MEMBRANE PHOSPHOLIPASE (OMPLA) <UNP PA1_ECOLI> [ESCHERICHIA COLI]
FTLYPYDTNYLIYTQTSDLNKEAIASYDWAENARKDEVKFQLSLAFPLWRGILGPNSVLG
ASYTQKSWWQLSNSEESSPFRETNYEPQLFLGFATDYRFAGWTLRDVEMGYNHDSNGRSD
PTSRSWNRLYTRLMAENGNWLVEVKPWYVVGNTDDNPDITKYMGYYQLKIGYHLGDAVLS
AKGQYNWNTGYGGAELGLSYPITKHVRLYTQVYSGYGESLIDYNFNQTRVGVGVMLNDLF
>2QNAB 66 XRAY 2.84 0.235 0.282 no Snurportin-1 <UNP SPN1_HUMAN> [HOMO SAPIENS]
MEELSQALASSFSVSQDLNSTAAPHPRLSQYKSKYSSLEQSERRRRLLELQKSKRLDYVN
HARRLA
>1GQEA 365 XRAY 1.81 0.222 0.247 no RELEASE FACTOR 2 <UNP RF2_ECOLI> [ESCHERICHIA COLI] MFEINPVNNRIQDLTERSDVLRGYLDYDAKKERLEEVNAELEQPDVWNEPERAQALGKER
SSLEAVVDTLDQMKQGLEDVSGLLELAVEADDEETFNEAVAELDALEEKLAQLEFRRMFS
GEYDSADCYLDIQAGSGGTEAQDWASMLERMYLRWAESRGFKTEIIEESEGEVAGIKSVT
IKISGDYAYGWLRTETGVHRLVRKSPFDSGGRRHTSFSSAFVYPEVDDDIDIEINPADLR
IDVYRASGAGGQHVNRTESAVRITHIPTGIVTQCQNDRSQHKNKDQAMKQMKAKLYEVEM
QKKNAEKQAMEDNKSDIGWGSQIRSYVLDDSRIKDLRTGVETRNTQAVLDGSLDQFIEAS
LKAGL
>4G4SO 276 XRAY 2.49 0.184 0.232 no Proteasome chaperone 1 <UNP POC1_YEAST> [SACCHAROMYCES CEREVISIAE]
MLFKQWNDLPEPKHLLDLPEISKNLQSLEVCPVPKVEFPQDLDVPQYSTAVITTKIMNPL
FPKNLLQLTSIGEIKTTLTVKSPSLPQSSGKHSWNYDENFPNEVDPDQKNDTADETVYGF
SFPIYSFGKTLLFSMEENFISISPIFGNMISRSIISQLAQFSPDIIVIGTSDKIASMKVM
TENECTLQPPEFITGFIGSVLTQLIVGPSKGLKFKCLVAPSEGPNGFEKLSLSDMGSLVD
LCGQWLGFEPSRYSEECYRLWRCDSAAIGAQSGLYI

>2VGNA 386 XRAY 2.50 0.210 0.251 no DOM34 <UNP DOM34_YEAST> [SACCHAROMYCES CEREVISIAE]
MKVISLKKDSFNKGGAVITLLPEDKEDLFTVYQIVDKDDELIFKKKFTSKLDEAGKKKST
DLVKLKIKVISEDFDMKDEYLKYKGVTVTDESGASNVDIPVGKYLSFTLDYVYPFTIIKQ
NFNKFMQKLLNEACNIEYKSDTAAVVLQEGIAHVCLVTSSSTILKQKIEYSMPKKKRTTD
VLKFDEKTEKFYKAIYSAMKKDLNFDKLKTIILCSPGFYAKILMDKIFQYAEEEHNKKIL
DNKGMFFIAHCSTGYLQGINEVLKNPLYASKLQDTKYSKEIMVMDEFLLHLNKDDDKAWY
GEKEVVKAAEYGAISYLLLTDKVLHSDNIAQREEYLKLMDSVESNGGKALVLSTLHSLGE
ELDQLTGIACILKYPLPDLDEDDGEE
>1WLFA 179 XRAY 2.05 0.213 0.258 no Peroxisome biogenesis factor 1 <UNP PEX1_MOUSE> [MUS MUSCULUS]
SSDRLAGAGSGGAVVTVAFTNARDCFLHLPRRLVAQLHLLQNQAIEVASDHQPTYLSWVE
GRHFNDQSENVAEINRQVGQKLGLSSGDQVFLRPCSHVVSCQQVEVEPLSADDWEILELH
AISLEQHLLDQIRIVFPKAVVPIWVDQQTYIFIQIVTLMPAAPYGRLETNTKLLIQPKT
>2YEXA 276 XRAY 1.30 0.135 0.164 no SERINE/THREONINE-PROTEIN KINASE CHK1 <UNP CHK1_HUMAN> [HOMO SAPIENS] MAVPFVEDWDLVQTLGEGAYGEVQLAVNRVTEEAVAVKIVDMKRAVDCPENIKKEICINK
MLNHENVVKFYGHRREGNIQYLFLEYCSGGELFDRIEPDIGMPEPDAQRFFHQLMAGVVY
LHGIGITHRDIKPENLLLDERDNLKISDFGLATVFRYNNRERLLNKMCGTLPYVAPELLK
RREFHAEPVDVWSCGIVLTAMLAGELPWDQPSDSCQEYSDWKEKKTYLNPWKKIDSAPLA
LLHKILVENPSARITIPDIKKDRWYNKPLKKGAKRP
>3GKUA 225 XRAY 2.95 0.223 0.287 no Probable RNA-binding protein <PDB 3GKU> [CLOSTRIDIUM SYMBIOSUM ATCC 14940]
SNAMDMVTVTAKTVEEAVTKALIELQTTSDKLTYEIVEKGSAGFLGIGSKPAIIRAKRKE
TLQDKAIEFLEQVFDAMNMAVDISVEYNETEKEMNVNLKGDDMGILIGKRGQTLDSLQYL
VSLVVNKSSSDYIRVKLDTENYRERRKETLETLAKNIAYKVKRTKRSVSLEPMNPYERRI
IHAALQNDKYVVTRSDGEEPFRHVIISLKRENRRDRNDRSDRNEK
>2FJRA 189 XRAY 1.95 0.219 0.247 no Repressor protein CI <UNP RPC1_BP186> [ENTEROBACTERIA PHAGE 186]
DSLGWSNVDVLDRICEAYGFSQKIQLANHFDIASSSLSNRYTRGAISYDFAAHCALETGA
NLQWLLTGEGEAFVNNRESSDAKRIEGFTLSEEILKSDKQLSVDAQFFTKPLTDGMAIRS
EGKIYFVDKQASLSDGLWLVDIKGAISIRELTKLPGRKLHVAGGKVPFECGIDDIKTLGR
VVGVYSEVN
>3T3PA 457 XRAY 2.20 0.189 0.220 no Integrin alpha-IIb <UNP ITA2B_HUMAN> [HOMO SAPIENS]
LNLDPVQLTFYAGPNGSQFGFSLDFHKDSHGRVAIVVGAPRTLGPSQEETGGVFLCPWRA
EGGQCPSLLFDLRDETRNVGSQTLQTFKARQGLGASVVSWSDVIVACAPWQHWNVLEKTE
EAEKTPVGSCFLAQPESGRRAEYSPCRGNTLSRIYVENDFSWDKRYCEAGFSSVVTQAGE
LVLGAPGGYYFLGLLAQAPVADIFSSYRPGILLWHVSSQSLSFDSSNPEYFDGYWGYSVA
VGEFDGDLNTTEYVVGAPTWSWTLGAVEILDSYYQRLHRLRGEQMASYFGHSVAVTDVNG
DGRHDLLVGAPLYMESRADRKLAEVGRVYLFLQPRGPHALGAPSLLLTGTQLYGRFGSAI
APLGDLDRDGYNDIAVAAPYGGPSGRGQVLVFLGQSEGLRSRPSQVLDSPFPTGSAFGFS
LRGAVDIDDNGYPDLIVGAYGANQVAVYRAQPVVKAS
>3EC3A 250 XRAY 1.92 0.190 0.236 no Protein disulfide-isomerase A4 <UNP PDIA4_RAT> [RATTUS NORVEGICUS]
GPLGSPPSKEILTLKQVQEFLKDGDDVVILGVFQGVGDPGYLQYQDAANTLREDYKFHHT
FSTEIAKFLKVSLGKLVLMQPEKFQSKYEPRMHVMDVQGSTEASAIKDYVVKHALPLVGH
RKTSNDAKRYSKRPLVVVYYSVDFSFDYRTATQFWRNKVLEVAKDFPEYTFAIADEEDYA
TEVKDLGLSESGGDVNAAILDESGKKFAMEPEEFDSDALREFVMAFKKGKLKPVIKSQPV
PKNNKGAAAS
>3NNRA 228 XRAY 2.49 0.232 0.260 no Transcriptional regulator, TetR family <UNP A1U6M1_MARAV>

[MARINOBACTER AQUAEOLEI] GMTMKTRDKILLSSLELFNDKGERNITTNHIAAHLAISPGNLYYHFRNKSDIIYEIFQEY
EKLVDYYLDIPEDRPITLEDMTFYLESVFDGLWSYRFFHRDLEYLLDSDPRLRQDYREFT
NRCLAAINRIFAKLADAGIIQPQPEDLRSAMSLNVWLVITNWMAFLKTAHAAEEPASLSL
TELKQGIYQVLTLEVPYLTPEYRERVLALREKYRPTLPEAQGISGVEA
>4NHEA 328 XRAY 1.95 0.177 0.207 no Oxidoreductase, Gfo/Idh/MocA family <UNP Q97PV8_STRPN>
[STREPTOCOCCUS PNEUMONIAE] SNAMLKLGVIGTGAISHHFIEAAHTSGEYQLVAIYSRKLETAATFASRYQNIQLFDQLEV
FFKSSFDLVYIASPNSLHFAQAKAALSAGKHVILEKPAVSQPQEWFDLIQTAEKNNCFIF
EAARNYHEKAFTTIKNFLADKQVLGADFNYAKYSSKMPDLLAGQTPNVFSDRFAGGALMD
LGIYPLYAAVRLFGKANDATYHAQQLDNSIDLNGDGILFYPDYQVHIKAGKNITSNLPCE
IYTTDGTLTLNTIEHIRSAIFTDHQGNQVQLPIQQAPHTMTEEVAAFAHMIQQPDLNLYQ
TWLYDAGSVHELLYTMRQTAGIRFEAEK
>2AVTA 378 XRAY 2.00 0.245 0.284 no DNA polymerase III beta subunit <GB AAF98349> [STREPTOCOCCUS PYOGENES]
MIQFSINRTLFIHALNTTKRAISTKNAIPILSSIKIEVTSTGVTLTGSNGQISIENTIPV
SNENAGLLITSPGAILLEASFFINIISSLPDISINVKEIEQHQVVLTSGKSEITLKGKDV
DQYPRLQEVSTENPLILKTKLLKSIIAETAFAASLQESRPILTGVHIVLSNHKDFKAVAT
DSHRMSQRLITLDNTSADFMVVLPSKSLREFSAVFTDDIETVEVFFSPSQILFRSEHISF
YTRLLEGNYPDTDRLLMTEFETEVVFNTQSLRHAMERAFLISNATQNGTVKLEITQNHIS
AHVNSPEVGKVNEDLDIVSQSGSDLTISFNPTYLIESLKAIKSETVKIHFLSPVRPFTLT
PGDEEESFIQLITPVRTN
>3D37A 381 XRAY 2.10 0.222 0.273 no Tail protein, 43 kDa <UNP Q9JZC8_NEIMB> [NEISSERIA MENINGITIDIS MC58]
MQNNSYGYAVSVRVGGKEHRHWERYDIDSDFLIPADSFDFVIGRLGPEAAIPDLSGESCE
VVIDGQIVMTGIIGSQRHGKSKGSRELSLSGRDLAGFLVDCSAPQLNVKGMTVLDAAKKL
AAPWPQIKAVVLKAENNPALGKIDIEPGETVWQALTHIANSVGLHPWLEPDGTLVVGGAD
YSSPPVATLCWSRTDSRCNIERMDIEWDTDNRFSEVTFLAQSHGRSGDSAKHDLKWVYKD
PTMTLHRPKTVVVSDADNLAALQKQAKKQLADWRLEGFTLTITVGGHKTRDGVLWQPGLR
VHVIDDEHGIDAVFFLMGRRFMLSRMDGTQTELRLKEDGIWTPDAYPKKAEAARKRKGKR KGVSHKGKKGGKKQAETAVFE
>1NNHA 294 XRAY 1.65 0.172 0.191 no asparaginyl-tRNA synthetase-related peptide <UNP Q8TZN6_PYRFU>
[PYROCOCCUS FURIOSUS]
MNAVEIISREISPTLDIQTKILEYMTDFFVKEGFKWLLPVIISPITDPLWPDPAGEGMEP
AEVEIYGVKMRLTHSMILHKQLAIAMGLKKIFVLSPNIRLESRQKDDGRHAYEFTQLDFE
VERAKMEDIMRLIERLVYGLFRKAEEWTGREFPKTKRFEVFEYSEVLEEFGSDEKASQEM
EEPFWIINIPREFYDREVDGFWRNYDLILPYGYGEVASGGEREWEYEKIVAKIRKAGLNE
DSFRPYLEIAKAGKLKPSAGAGIGVERLVRFIVGAKHIAEVQPFPRIPGIPAVI
>3MFIA 520 XRAY 1.76 0.165 0.187 no DNA polymerase eta <UNP POLH_YEAST> [SACCHAROMYCES CEREVISIAE] GPGGDPHMSKFTWKELIQLGSPSKAYESSLACIAHIDMNAFFAQVEQMRCGLSKEDPVVC
VQWNSIIAVSYAARKYGISRMDTIQEALKKCSNLIPIHTAVFKKGEDFWQYHDGCGSWVQ
DPAKQISVEDHKVSLEPYRRESRKALAIFKWACDLVERASIDEVFLDLGRICFNMLMFDN
EYELTGDLKLKDALSNIREAFIGGNYDINSHLPLIPEKIKSLKFEGDVFNPEGRDLITDW
DDVILALGSQVCKGIRDSIKDILGYTTSCGLSSTKNVCKLASNYKKPDAQTIVKNDCLLD
FLDCGKFEITSFWTLGGVLGKELIDVLDLPHENSIKHIRETWPDNAGQLKEFLDAKVKQS
DYDRSTSNIDPLKTADLAEKLFKLSRGRYGLPLSSRPVVKSMMSNKNLRGKSCNSIVDCI
SWLEVFCAELTSRIQDLEQEYNKIVIPRTVSISLKTKSYEVYRKSGPVAYKGINFQSHEL
LKVGIKFVTDLDIKGKNKSYYPLTKLSMTITNFDIIDLQK
>3U2GA 286 XRAY 2.30 0.180 0.221 no S-layer protein MA0829 <UNP Q8TSG7_METAC> [METHANOSARCINA ACETIVORANS]

MGTYEIRGQVASGFGDQSWDASSFAGFYYDIDDNVSTETLTVSDLDGNVIPEGGLVYTTT
IADVDFEYYNPDAGWDQYPVMGFFAEEYIPINPDKADKIAKLVLDSDDKYTIRTGEMLDL
GEGYAIEAKQVDVDGEKVWLEFTKDGEFVDDEIISVSTADDEANTWDVELDDIEDEDDVV
VLKVHVNQVFQGAVDSIAQIEGLWLIDYANAMTIESDDEFGNLDDVSIDGDTLKISNEDT
FTLTRDSEEEIGEGMYFMIADTSSSDLRYYPYVEKTIGLEHHHHHH

>1VR0A 247 XRAY 2.49 0.192 0.229 no Probable 2-phosphosulfolactate phosphatase <UNP COMB_CLOAB> [CLOSTRIDIUM ACETOBUTYLICUM]
MGSDKIHHHHHHMKIDLIISADDIKEEKVKNKTAVVIDMLRATSVITTALNNGCKRVVPV
LTVEEALKKVKEYGKDAILGGERKGLKIEGFDFSNSPMEYTEDVVKGKTLIMTTTNGTRA
IKGSETARDILIGSVLNGEAVAEKIVELNNDVVIVNAGTYGEFSIDDFICSGYIINCVMD
RMKKLELTDAATTAQYVYKTNEDIKGFVKYAKHYKRIMELGLKKDFEYCCKKDIVKLVPQ
YTNGEIL

>1O6DA 163 XRAY 1.66 0.229 0.261 no Hypothetical UPF0247 protein TM0844 <UNP Y844_THEMA> [THERMOTOGA MARITIMA] MSLRVRIAVIGKLDGFIKEGIKHYEKFLRRFCKPEVLEIKRVHRGSIEEIVRKETEDLTN
RILPGSFVMVMDKRGEEVSSEEFADFLKDLEMKGKDITILIGGPYGLNEEIFAKAHRVFS
LSKMTFTHGMTVLIVLEQIFRAFKIIHGENYHYEGGSHHHHHH

>2DB7A 64 XRAY 1.90 0.192 0.220 no Hairy/enhancer-of-split related with YRPW motif 1 <UNP HEY1_HUMAN> [HOMO SAPIENS]
GSSGSSGGYFDAHALAMDYRSLGFRECLAEVARYLSIIEGLDASDPLRVRLVSHLNNYAS
QREA

>1AISB 200 XRAY 2.10 0.212 0.268 no TRANSCRIPTION INITIATION FACTOR IIB <UNP TF2B_PYRWO> [PYROCOCCUS WOESEI]
VSDAAERNLAFALSELDRITAQLKLPRHVEEEAARLYREAVRKGLIRGRSIESVMAACVY
AACRLLKVPRTLDEIADIARVDKKEIGRSYRFIARNLNLTPKKLFVKPTDYVNKFADELG
LSEKVRRRAIEILDEAYKRGLTSGKSPAGLVAAALYIASLLEGEKRTQREVAEVARVTEV
TVRNRYKELVEKLKIKVPIA

>2FD6U 276 XRAY 1.90 0.239 0.276 no Urokinase plasminogen activator surface receptor <UNP UPAR_HUMAN> [HOMO SAPIENS]
SLRCMQCKTNGDCRVEECALGQDLCRTTIVRLWEEGEELELVEKSCTHSEKTNRTLSYRT
GLKITSLTEVVCGLDLCNQGNSGRAVTYSRSRYLECISCGSSDMSCERGRHQSLQCRSPE
EQCLDVVTHWIQEGEEGRPKDDRHLRGCGYLPGCPGSNGFHNNDTFHFLKCCNTTKCNEG
PILELENLPQNGRQCYSCKGNSTHGCSSEETFLIDCRGPMNQCLVATGTHEPKNQSYMVR
GCATASMCQHAHLGDAFSMNHIDVSCCTKSGCNHPD

>2PJZA 263 XRAY 1.90 0.198 0.242 no Hypothetical protein ST1066 <UNP Q972R9_SULTO> [SULFOLOBUS TOKODAII]
MIQLKNVGITLSGKGYERFSLENINLEVNGEKVIILGPNGSGKTTLLRAISGLLPYSGNI
FINGMEVRKIRNYIRYSTNLPEAYEIGVTVNDIVYLYEELKGLDRDLFLEMLKALKLGEE
ILRRKLYKLSAGQSVLVRTSLALASQPEIVGLDEPFENVDAARRHVISRYIKEYGKEGIL
VTHELDMLNLYKEYKAYFLVGNRLQGPISVSELLESSIVEGERNDALLVLDIMDKKVSIV
KGDLGMKFGALGSLNRIYGIIGA

>3LUIA 115 XRAY 1.80 0.203 0.245 no Sorting nexin-17 <UNP SNX17_HUMAN> [HOMO SAPIENS]
SNAMHFSIPETESRSGDSGGSAYVAYNIHVNGVLHCRVRYSQLLGLHEQLRKEYGANVLP
AFPPKKLFSLTPAEVEQRREQLEKYMQAVRQDPLLGSSETFNSFLRRAQQETQQV

>3OLOA 118 XRAY 2.09 0.180 0.227 no Two-component sensor histidine kinase <UNP Q8YZM9_ANASP> [NOSTOC SP.] SNAMNIQSELEFKFAHYLINNAVEASFCLGDNWQFLYVNDATCRMTEYSREQLLSMNLQD
IDVDFALHDWEEIRQKNNYTFKTRYRSQSGRIFLVEMSLTFLEDQERRFSCVFVREKS

>4DNHA 396 XRAY 2.50 0.177 0.240 no Uncharacterized protein <UNP Q92T60_RHIME> [SINORHIZOBIUM MELILOTI]

SLVSIDLPIEGRLARYDLTGRPVPFNSRDAKAFSRVAFAAAHVVADPLADNDPWLAPAID
WERTLAFRHRLWDLGLGVAESMDTAQRGMGLGWPEARELIRRSLAEARGRPDALIACGAG
TDHLAPGPDVSIDDILAAYESQIEAIEAEGGRIILMASRALAAAAKGPEDYIRVYDRVLS
QVKEPVIIHWLGEMFDPALEGYWGNADHMAAMKTCLDVLEAHAAKVDGIKISLLSKEKEI
VMRRQLPKGVRMYTGDDFNYAELIAGDEEGHSDALLGIFDAIAPVASAALEALGSGRNGE
FFELLEPTVPLSRHIFKAPTRFYKTGVVFLAYLNGLQDHFVMIGGQQSARSLVHLAELFR
LADKAGALADPELATARMRRVLAMHGVEEGHHHHHH
>1LJ8A 493 XRAY 1.70 0.171 0.197 no mannitol dehydrogenase <UNP O08355_PSEFL> [PSEUDOMONAS FLUORESCENS] MKLNKQNLTQLAPEVKLPAYTLADTRQGIAHIGVGGFHRAHQAYYTDALMNTGEGLDWSI
CGVGLRSEDRKARDDLAGQDYLFTLYELGDTDDTEVRVIGSISDMLLAEDSAQALIDKLA
SPEIRIVSLTITEGGYCIDDSNGEFMAHLPQIQHDLAHPSSPKTVFGFICAALTQRRAAG
IPAFTVMSCDNLPHNGAVTRKALLAFAALHNAELHDWIKAHVSFPNAMVDRITPMTSTAH
RLQLHDEHGIDDAWPVVCEPFVQWVLEDKFVNGRPAWEKVGVQFTDDVTPYEEMKIGLLN
GSHLALTYLGFLKGYRFVHETMNDPLFVAYMRAYMDLDVTPNLAPVPGIDLTDYKQTLVD
RFSNQAIADQLERVCSDGSSKFPKFTVPTINRLIADGRETERAALVVAAWALYLKGVDEN
GVSYTIPDPRAEFCQGLVSDDALISQRLLAVEEIFGTAIPNSPEFVAAFERCYGSLRDNG VTTTLKHLLKKPV
>4HVTA 711 XRAY 1.70 0.172 0.209 no Post-proline cleaving enzyme <UNP Q68XJ3_RICTY> [RICKETTSIA TYPHI]
MAHHHHHHMAMEDNNKQIFNPKETKFLEEAEGVEALEWAKERTSKTEKALQAMQEYKQIK
KEIETIFYDQRKTPYGVIRKGYVYNFWMDDKNPQGLWRRTLVDNYSKDKPNWEVLIDFDK
LSKKIGKKVAYRGVSNCFQNPNRYLISMSFGGKDEMFFREWDLEKKDFVKNGFEPITNSG
KLLEGKFTYPTWINKDTIIFNLVLHKNEITSSLYPNSLYIWKRGESIEKAKKLFEVPKEY
IYVSAGKLLSDTISSSLIFISANKDFYNYDNYILDTKYKNLKLQKINMPSDATLQGSFKE
YVFWLLRSDWKFKSHNIKAGSLVALHFTDLLKTESDKTSLKILFTPTANEVFNFISTTKD
RVFLATYDNVVAKVVTFTLENEQWTKPVVLKLPYQNAIFGMSSYEEEEEALITIENSIVP
PTIYLWVKTHELKIIRKALYSFDSENYVLEQKEATSFDGVKIPYFLVYKKGIKFDGKNPT
LLEAYGGFQVINAPYFSRIKNEVWVKNAGVSVLANIRGGGEFGPEWHKSAQGIKRQTAFN
DFFAVSEELIKQNITSPEYLGIKGGSNGGLLVSVAMTQRPELFGAVACEVPILDMIRYKE
FGAGHSWVTEYGDPEIPNDLLHIKKYAPLENLSLTQKYPTVLITDSVLDQRVHPWHGRIF
EYVLAQNPNTKTYFLESKDSGHGSGSDLKESANYFINLYTFFANALKLKIN
>3KVHA 214 XRAY 1.70 0.185 0.217 no Protein syndesmos <UNP SDOS_HUMAN> [HOMO SAPIENS]
MVPELKQISRVEAMRLGPGWSHSCHAMLYAANPGQLFGRIPMRFSVLMQMRFDGLLGFPG
GFVDRRFWSLEDGLNRVLGLGLGCLRLTEADYLSSHLTEGPHRVVAHLYARQLTLEQLHA
VEISAVHSRDHGLEVLGLVRVPLYTQKDRVGGFPNFLSNAFVSTAKCQLLFALKVLNMMP
EEKLVEALAAATEKQKKALEKLLPASSAHHHHHH
>4IIYA 371 XRAY 1.20 0.164 0.175 no MccF <UNP Q2KKH9_ECOLX> [ESCHERICHIA COLI]
MGHHHHHHHHHHHSSGHIDDDDKHMLEMIQSHPLLAAPLAVGDTIGFFSSSAPATVTAK
NRFFRGVEFLQRKGFKLVSGKLTGKTDFYRSGTIKERAQEFNELVYNPDITCIMSTIGGD
NSNSLLPFLDYDAIIANPKIIIGYADTTALLAGIYAKTGLITFYGPALIPSFGEHPPLVD
ITYESFIKILTRKQSGIYTYTLPEKWSDESINWNENKILRPKKLYKNNCAFYGSGKVEGR
VIGGNLNTLTGIWGSEWMPEIRNGDILFIEDSRKSIATVERLFSMLKLNRVFDKVSAIIL
GKHELFDCAGSKRRPYEVLTEVLDGKQIPVLDGFDCSHTHPMLTLPLGVKLAIDFDNKNI
SITEQYLSTEK
>1S9RA 410 XRAY 1.60 0.168 0.202 no Arginine deiminase <UNP ARCA_MYCAR> [MYCOPLASMA ARGININI]
MSVFDSKFKGIHVYSEIGELESVLVHEPGREIDYITPARLDELLFSAILESHDARKEHKQ
FVAELKANDINVVELIDLVAETYDLASQEAKDKLIEEFLEDSEPVLSEEHKVVVRNFLKA
KKTSRELVEIMMAGITKYDLGIEADHELIVDPMPNLYFTRDPFASVGNGVTIHYMRYKVR

QRETLFSRFVFSNHPKLINTPWYYDPSLKLSIEGGDVFIYNNDTLVVGVSERTDLQTVTL
LAKNIVANKECEFKRIVAINVPKWTNLMHLDTWLTMLDKDKFLYSPIANDVFKFWDYDLV
NGGAEPQPVENGLPLEGLLQSIINKKPVLIPIAGEGASQMEIERETHFDGTNYLAIRPGV
VIGYSRNEKTNAALEAAGIKVLPFHGNQLSLGMGNARCMSMPLSRKDVKW
>2Y1EA 398 XRAY 1.65 0.187 0.213 no 1-DEOXY-D-XYLULOSE 5-PHOSPHATE REDUCTOISOMERASE <UNP
A2VLK3_MYCTU> [MYCOBACTERIUM TUBERCULOSIS]
TMAHHHHHHVTNSTDGRADGRLRVVVLGSTGSIGTQALQVIADNPDRFEVVGLAAGGAHL
DTLLRQRAQTGVTNIAVADEHAAQRVGDIPYHGSDAATRLVEQTEADVVLNALVGALGLR
PTLAALKTGARLALANKESLVAGGSLVLRAARPGQIVPVDSEHSALAQCLRGGTPDEVAK
LVLTASGGPFRGWSAADLEHVTPEQAGAHPTWSMGPMNTLNSASLVNKGLEVIETHLLFG
IPYDRIDVVVHPQSIIHSMVTFIDGSTIAQASPPDMKLPISLALGWPRRVSGAAAACDFH
TASSWEFEPLDTDVFPAVELARQAGVAGGCMTAVYNAANEEAAAAFLAGRIGFPAIVGII
ADVLHAADQWAVEPATVDDVLDAQRWARERAQRAVSGM
>2FK5A 200 XRAY 1.90 0.190 0.233 no fuculose-1-phosphate aldolase <UNP Q5SHB9_THET8> [THERMUS
THERMOPHILUS]
MRARLYAAFRQVGEDLFAQGLISATAGNFSVRTKGGFLITKSGVQKARLTPEDLLEVPLE
GPIPEGASVESVVHREVYRRTGARALVHAHPRVAVALSFHLSRLRPLDLEGQHYLKEVPV
LAPKTVSATEEAALSVAEALREHRACLLRGHGAFAVGLKEAPEEALLEAYGLMTTLEESA
QILLYHRLWQGAGPALGGGE
>1MQVA 125 XRAY 1.78 0.220 0.265 no CYTOCHROME C <UNP CYCP_RHOPA> [RHODOPSEUDOMONAS
PALUSTRIS] ATDVIAQRKAILKQMGEATKPIAAMLKGEAKWDQAVVQKSLAAIADDSKKLPALFPADSK
TGGDTAALPKIFEDKAKFDDLFAKLAAAATAAQGTIKDEASLKANIGGVLGNCKSCHDDF
RAKKS
>3LAAA 200 XRAY 1.35 0.115 0.128 no Haemagglutinin family protein <UNP Q3JLD6_BURP1>
[BURKHOLDERIA PSEUDOMALLEI]
MAHHHHHHMGTLEAQTQGPGSMGSLSTSISSITTNTTNLGNSTAAALGGGATYDPATGAI
SAPSYTTYNANGTTATNTSVGAAIDNINANGIKYFHANSTDPDSVATGTNSVAIGPNAVA
NVDYSVAIGSGATTSAAVPVASASVGGLTFGGFAGSAPIGVFSVGAPGAERQITNVAAGR
ISAASTDAVNGSQLYATNSN
>4NMWA 259 XRAY 1.50 0.143 0.179 no Pimelyl-[acyl-carrier protein] methyl ester esterase <UNP
BIOH_SALTY> [SALMONELLA ENTERICA SUBSP. ENTERICA SEROVAR TYPHIMURIUM]
SNAMNDIWWQTYGEGNCHLVLLHGWGLNAEVWHCIREELGSHFTLHLVDLPGYGRSSGFG
AMTLEEMTAQVAKNAPDQAIWLGWSLGGLVASQMALTHPERVQALVTVASSPCFSAREGW
PGIKPEILGGFQQQLSDDFQRTVERFLALQTLGTETARQDARTLKSVVLAQPMPDVEVLN
GGLEILKTVDLREALKNVNMPFLRLYGYLDGLVPRKIVPLLDTLWPHSTSQIMAKAAHAP
FISHPAAFCQALMTLKSSL
>4JDNA 152 XRAY 2.00 0.157 0.191 no Virulence plasmid protein pGP3-D <UNP D7DHH5_CHLTL>
[CHLAMYDIA TRACHOMATIS]
SLGLLKAFNNFPITNKIQCNGLFTPSNIETLLGGTEIGKFTVTPKSSGSMFLVSADIIAS
RMEGGVVLALVREGDSKPCAISYGYSSGVPNLCSLRTSITNTGLTPTTYSLRVGGLESGV
VWVNALSNGNDILGITNTSNVSFLEVIPQTNA
>2QDJA 304 XRAY 2.00 0.231 0.276 no Retinoblastoma-associated protein <UNP RB_HUMAN> [HOMO
SAPIENS] TEEPDFTALCQKLKIPDHVRERAWLTWEKVSSVDGVLGGYIQKKKELWGICIFIAAVDLD
EMSFTFTELQKNIEISVHKFFNLLKEIDTSTKVDNAMSRLLKKYDVLFALFSKLERTCEL
IYLTQPSSSISTEINSALVLKVSWITFLLAKGEVLQMEDDLVISFQLMLCVLDYFIKLSP
PMLLKEPYKTAVIPINGSPRTPRRGQNRSARIAKQLENDTRIIEVLCKEHECNIDEVKNV
YFKNFIPFMNSLGLVTSNGLPEVENLSKRYEEIYLKNKDLDARLFLDHDKTLQTDSIDSF

ETQR
>1Y8QB 640 XRAY 2.25 0.208 0.248 no Ubiquitin-like 2 activating enzyme E1B <UNP ULE1B_HUMAN> [HOMO SAPIENS]
MALSRGLPRELAEAVAGGRVLVVGAGGIGCELLKNLVLTGFSHIDLIDLDTIDVSNLNRQ
FLFQKKHVGRSKAQVAKESVLQFYPKANIVAYHDSIMNPDYNVEFFRQFILVMNALDNRA
ARNHVNRMCLAADVPLIESGTAGYLGQVTTIKKGVTECYECHPKPTQRTFPGATIRNTPS
EPIHCIVWAKYLFNQLFGEEDADQEVSPDRADPEAAWEPTEAEARARASNEDGDIKRIST
KEWAKSTGYDPVKLFTKLFKDDIRYLLTMDKLWRKRKPPVPLDWAEVQSQGEETNASDQQ
NEPQLGLKDQQVLDVKSYARLFSKSIETLRVHLAEKGDGAELIWDKDDPSAMDFVTSAAN
LRMHIFSMNMKSRFDIKSMAGNIIPAIATTNAVIAGLIVLEGLKILSGKIDQCRTIFLNK
QPNPRKKLLVPCALDPPNPNCYVCASKPEVTVRLNVHKVTVLTLQDKIVKEKFAMVAPDV
QIEDGKGTILISSEEGETEANNHKKLSEFGIRNGSRLQADDFLQDYTLLINILHSEDLGK
DVEFEVVGDAPEKVGPKQAEDAAKSITNGSDDGAQPSTSTAQEQDDVLIVDSDEEDSSNN
ADVSEEERSRKRKLDEKENLSAKRSRIEQKEELDDVIALD
>2NS0A 85 XRAY 2.00 0.190 0.216 no Hypothetical protein <UNP Q0S2K3_RHOSR> [RHODOCOCCUS SP.]
MTVSDRELEECIRALLDARADSASICPSDVARAVAPDDWRPLMEPVREAAGRLADAGEVE
VTQKGAVVDPRSARGPIRIRWTRTD
>4JOSA 250 XRAY 1.45 0.152 0.175 no Adenosylhomocysteine nucleosidase <UNP B0TZL4_FRAP2> [FRANCISELLA PHILOMIRAGIA SUBSP. PHILOMIRAGIA]
MHHHHHHSSGVDLGTENLYFQSMKKIAILGAMEIEIQPILQKLEKYETVEYANNKYYVAN
YNGIELVVAYSKIGKVFSSLTATIMIEHFGVDALLFTGVAGGLQDLQVGDMIAATATVQH
DVDITAFGYPYGKIPISEVEIATSARILEQAKVIAKELNLNLHTGVIATGDQFVHSAERK
DFVVKEFDAKAIEMEGASVNLICNEMNIPSFILRSISDTADGDAPDNFDEFAKMAANRSA
DFVMKLVDRI
>3R6DA 221 XRAY 1.25 0.150 0.179 no NAD-dependent epimerase/dehydratase <UNP D1BQI7_VEIPT> [VEILLONELLA PARVULA DSM 2008] SNAMYKYITILGAAGQIAQKLTATLLTYTDMHITLYGRQLKTRIPPEIIDHERVTVIEGS
FQNPGKLEQAVTNAEVVFVGAMESGSDMASIVKALSRKNIRRVIGVSMAGLSGEFPVALE
KWTFDNLPISYVQGERQARNVLRESNLNYTILRLTWLYNDPEKTDYELIPEGAQFNDAQV
SREAVVKAIFDILHAADETPFHRTSIGVGEPGTHYDKPSFH
>3CZ7A 364 XRAY 2.00 0.158 0.189 no Regulator of Ty1 transposition protein 109 <UNP RT109_YEAST> [SACCHAROMYCES CEREVISIAE]
MSLNDFLSSVLPVSEQFEYLSLQSIPLETHAVVTPNKDDKRVPKSTIKTQHFFSLFHQGK
VFFSLEVYVYVTLWDEADAERLIFVSKADTNGYCNTRVSVRDITKIILEFILSIDPNYYL
QKVKPAIGGSGFQQDLYLSFTCPREILTKICLFTRPASQYLFPDSSKNSKKHILNGEELM
KWWGFILDRLLIECFQNDTQAKLRIPGEDPARVRSYLRGMKYPLWQVGDIFTSKENSLAV
YNIPLFPDDPXARFIHQLAEEDRLLKVSLSSFWIELQERQEFKLSVTSSVMGISGYSLAT
PSLFPSSADVIVPKSRKQFRAIKKYITGEEYDTEEGAIEAFTNIRDFLLLRMATNLQSLT
GKRE
>2NYKA 285 XRAY 2.10 0.215 0.240 no M157 <UNP Q6XK79_9BETA> [MURID HERPESVIRUS 1]
IFNPDPDDTYIVNMDDFQFTFTMEFEVTVTRGGVHKRTISVDNGRPVVVWDVGDRDPKIC
KICPDVSSTDIEYVFLDIQKMRLNNLLTQSLWDTQRICVRYACLFLGFDVICDVYHTTDT
VRVAYTGQTGKINIQGSGKFSTSDAKEIGTYMIKSNVREIKNRWRSTVQKLKQLAYMNAT
EVEFWYNTTGLTTCVVTSRSNVPFTVELSLNTNCSAIVTDESTVDCQILTVKAPGSHAQR
CYVTSSLGWKGVVTPPSQYRTKRVPVNISSSKWTGIVNWKGNVNR
>1RV9A 259 XRAY 1.53 0.197 0.209 no conserved hypothetical protein NMB0706 <GB NP_273748> [NEISSERIA MENINGITIDIS] MKTITETLNLAPKGKNFLTADWPAPANVKTLITTRNGGVSQGAYQSLNLGTHVGDNPEAV
RRNREIVQQQVGLPVAYLNQIHSTVVVNAAEALGGTPDADASVDDTGKVACAVMTADCLP

VLFCDRAGTAVAAAHAGWRGLAGGVLQNTIAAMKVPPVEMMAYLGPAISADAFEVGQDVF
DAFCTPMPEAATAFEGIGSGKFLADLYALARLILKREGVGGVYGGTHCTVLERDTFFSYR
RDGATGRMASLIWLDGNAV
>2BPA1 426 XRAY 3.00 0.209 NA no SUBUNIT OF BACTERIOPHAGE PHIX174 <UNP VGF_BPPHX> [ENTEROBACTERIA PHAGE PHIX174]
SNIQTGAERMPHDLSHLGFLAGQIGRLITISTTPVIAGDSFEMDAVGALRLSPLRRGLAI
DSTVDIFTFYVPHRHVYGEQWIKFMKDGVNATPLPTVNTTGYIDHAAFLGTINPDTNKIP
KHLFQGYLNIYNNYFKAPWMPDRTEANPNELNQDDARYGFRCCHLKNIWTAPLPPETELS
RQMTTSTTSIDIMGLQAAYANLHTDQERDYFMQRYRDVISSFGGKTSYDADNRPLLVMRS
NLWASGYDVDGTDQTSLGQFSGRVQQTYKHSVPRFFVPEHGTMFTLALVRFPPTATKEIQ
YLNAKGALTYTDIAGDPVLYGNLPPREISMKDVFRSGDSSKKFKIAEGQWYRYAPSYVSP
AYHLLEGFPFIQEPPSGDLQERVLIRHHDYDQCFQSVQLLQWNSQVKFNVTVYRNLPTTR
DSIMTS
>3EGLA 277 XRAY 2.41 0.181 0.218 no DegV family protein <UNP Y2349_CORGL> [CORYNEBACTERIUM GLUTAMICUM]
SNAMPVRVIVDSSACLPTHVAEDLDITVINLHVMNNGEERSTSGLSSLELAASYARQLER
GGDDGVLALHISKELSSTWSAAVTAAAVFDDDSVRVVDTSSLGMAVGAAAMAAARMAKDG
ASLQECYDIAVDTLKRSETWIYLHRIDEIWKSGRISTATAMVSTALATRPIMRFNGGRME
IAAKTRTQSKAFAKLVELAQIRADGEPVFIAIGQNEAREAAKQLEELLRNALPEGSSFMS
VDIDPTLAVHSGPGAVSVSAVFANQAPELSTGKAGAK
>2EVVA 207 XRAY 2.59 0.224 0.262 no hypothetical protein HP0218 <UNP O25006_HELPY> [HELICOBACTER PYLORI]
MGSSHHHHHHSSGRENLYFQGHMKTFEVMIQTDSKGYLDAKFGGNAPKAFLNSNGLPTYS
PKISWQKVEGAQSYALELIDHDAQKVCGMPFVHWVVGNIAHNVLEENASMMDKRIVQGVN
SLTQGFIRSPLNESEKQRSNLNNSVYIGPMPPNGDHHYLIQVYALDIPKLALKAPFFLGD
LHDKMRNHIIAIGRKEFLYKQFVRKGS
>2R8BA 251 XRAY 2.56 0.197 0.253 no Uncharacterized protein Atu2452 <UNP Q8UCN1_AGRT5> [AGROBACTERIUM TUMEFACIENS STR.]
GHMPRPSLRRGEETSGARSLHEIAPPAEKAVRKPLNLLPFRKDTPMTKDSYFHKSRAGVA
GAPLFVLLHGTGGDENQFFDFGARLLPQATILSPVGDVSEHGAARFFRRTGEGVYDMVDL
ERATGKMADFIKANREHYQAGPVIGLGFSNGANILANVLIEQPELFDAAVLMHPLIPFEP
KISPAKPTRRVLITAGERDPICPVQLTKALEESLKAQGGTVETVWHPGGHEIRSGEIDAV
RGFLAAYGGGS
>4C7AA 193 XRAY 2.30 0.238 0.284 no SMOOTHENED <UNP Q90X26_DANRE> [DANIO RERIO]
MAVILHPNETIFNDFCKKSTTCEVLKYNTCLGSPLPYTHTSLILAEDSETQEEAFEKLAM
WSGLRNAPRCWAVIQPLLCAVYMPKCENGKVELPSQHLCQATRNPCSIVERERGWPNFLK
CENKEQFPKGCQNEVQKLKFNTSGQCEAPLVKTDIQASWYKDVEGCGIQCDNPLFTEDEH
SDMHKLEHHHHHH
>2NWAA 88 XRAY 2.70 0.271 0.285 no Hypothetical protein ytmB <UNP YTMB_BACSU> [BACILLUS SUBTILIS]
MGMPVEFNTLIVTKGKEVRIDENIFTLEKDGYRVYPMEIPMDVRKTKFGEKSGTAEVQKL
QWEEGRTIITYKLTSLHSVNLEHHHHHH
>4LBOA 348 XRAY 1.70 0.159 0.192 no Uncharacterized protein <UNP B9JQV3_AGRVS> [AGROBACTERIUM VITIS]
SMRWKRMMQLLDVHCEGEIGKVAIGGVPKIPGDTVADQLHWLNTDPKGRELRHFLVLEPR
GAPIGSVNLLLPAKDSRADAAFIILQPDQAHASSGSNSICVTTALLESGMIEMQEPETVV
MLETAAGLVKAVAQCRDGHCDSVTLTMVPSFVHELDAQIATESWGEIRFDLAYGGVFYAL
VDVRQLGLTIEPGNARRLVEAGMLLKGEINQRIQVVHPDIPAISGVAYVMFRDEDPDGAV

RTCTTMWPGRVDRSPCGTGNSANLATLHARGRVKPGDSFLSRSIIGSQFTVGLQGLTTVA
GRSAVIPTITGRGFTYGIHQVALDAFDPLGGGFVLTDVWGAAAETIKI
>3NHVA 144 XRAY 2.50 0.204 0.247 no BH2092 protein <UNP Q9KB42_BACHD> [BACILLUS HALODURANS]
ANPNEAYRHYMKKLSYETDIADLSIDIKKGYEGIIVVDVRDAEAYKECHIPTAISIPGNK
INEDTTKRLSKEKVIITYCWGPACNGATKAAAKFAQLGFRVKELIGGIEYWRKENGEVEG
TLGAKADLFWNMKKESLEHHHHHH
>1SZ7A 200 XRAY 1.55 0.207 0.245 no Trafficking protein particle complex subunit 3 <UNP TPPC3_HUMAN> [HOMO SAPIENS]
MSHHHHHHGSSRQANRGTESKKMSSELFTLTYGALVTQLCKDYENDEDVNKQLDKMGFNI
GVRLIEDFLARSNVGRCHDFRETADVIAKVAFKMYLGITPSITNWSPAGDEFSLILENNP
LVDFVELPDNHSSLIYSNLLCGVLRGALEMVQMAVEAKFVQDTLKGDGVTEIRMRFIRRI
EDNLPAGEEAAAWSHPQFEK
>3C8DA 403 XRAY 1.80 0.150 0.194 no Enterochelin esterase <UNP Q83SB9_SHIFL> [SHIGELLA FLEXNERI 2A STR. 2457T]
SNAMTALKVGSESWWQSKHGPEWQRLNDEMFEVTFWWRDPQGSEEYSTIKRVWVYITGVT
DHHQNSQPQSMQRIAGTDVWQWTTQLNANWRGSYCFIPTERDDIFSAPSPDRLELREGWR
KLLPQAIADPLNPQSWKGGLGHAVSALEMPQAPLQPGWDCPQAPEIPAKEIIWKSERLKN
SRRVWIFTTGDVTAEERPLAVLLDGEFWAQSMPVWPVLTSLTHRQQLPPAVYVLIDAIDT
THRAHELPCNADFWLAVQQELLPLVKVIAPFSDRADRTVVAGQSFGGLSALYAGLHWPER
FGCVLSQSGSYWWPHRGGQQEGVLLEKLKAGEVSAEGLRIVLEAGIREPMIMRANQALYA
QLHPIKESIFWRQVDGGHDALCWRGGLMQGLIDLWQPLFHDRS
>2I2XB 258 XRAY 2.50 0.184 0.231 no Methyltransferase 1 <UNP P94920_METBA> [METHANOSARCINA BARKERI]
MLDFTEASLKKVLTRYNVALEKALTPEEAAEELYPKDELIYPIAKAIFEGEEDDVVEGLQ
AAIEAGKDPIDLIDDALMVGMGVVIRLYDEGVIFLPNVMMSADAMLEGIEYCKENSGATP
KTKGTVVCHVAEGDVHDIGKNIVTALLRANGYNVVDLGRDVPAEEVLAAVQKEKPIMLTG
TALMTTTMYAFKEVNDMLLENGIKIPFACGGGAVNQDFVSQFALGVYGEEAADAPKIADA
IIAGTTDVTELREKFHKH
>3DMLA 116 XRAY 1.90 0.157 0.201 no Putative uncharacterized protein <UNP Q8KM22_PARDE> [PARACOCCUS DENITRIFICANS]
MRGSHHHHHHGSDDDDKAELRLLMFEQPGCLYCARWDAEIAPQYPLTDEGRAAPVQRLQM
RDPLPPGLELARPVTFTPTFVLMAGDVESGRLEGYPGEDFFWPMLARLIGQAEPGQ
>1GQIA 708 XRAY 1.48 0.133 0.168 no ALPHA-GLUCURONIDASE <PDB 1GQI> [PSEUDOMONAS CELLULOSA]
EDGYDMWLRYQPIADQTLLKTYQKQIRHLHVAGDSPTINAAAAELQRGLSGLLNKPIVAR
DEKLKDYSLVIGTPDNSPLIASLNLGERLQALGAEGYLLEQTRINKRHVVIVAANSDVGV
LYGSFHLLRLIQTQHALEKLSLSSAPRLQHRVVNHWDNLNRVVERGYAGLSLWDWGSLPN
YLAPRYTDYARINASLGINGTVINNVNADPRVLSDQFLQKIAALADAFRPYGIKMYLSIN
FNSPRAFGDVDTADPLDPRVQQWWKTRAQKIYSYIPDFGGFLVKADSEGQPGPQGYGRDH
AEGANMLAAALKPFGGVVFWRAFVYHPDIEDRFRGAYDEFMPLDGKFADNVILQIKNGPI
DFQPREPFSALFAGMSRTNMMMEFQITQEYFGFATHLAYQGPLFEESLKTETHARGEGST
IGNILEGKVFKTRHTGMAGVINPGTDRNWTGHPFVQSSWYAFGRMAWDHQISAATAADEW
LRMTFSNQPAFIEPVKQMMLVSREAGVNYRSPLGLTHLYSQGDHYGPAPWTDDLPRADWT
AVYYHRASKTGIGFNRTKTGSNALAQYPEPIAKAWGDLNSVPEDLILWFHHLSWDHRMQS
GRNLWQELVHKYYQGVEQVRAMQRTWDQQEAYVDAARFAQVKALLQVQEREAVRWRNSCV
LYFQSVAGRPIPANYEQPEHDLEYYKMLARTTYVPEPWHPASSSRVLK
>4HYLA 117 XRAY 1.75 0.209 0.229 no Stage II sporulation protein <UNP D0LNN2_HALO1> [HALIANGIUM OCHRACEUM]

SNATDTQIRTEQGIDIITLHGHLDTRSSPAVQAAVLPRVTAKGKMILDLREVSYMSSAGL
RVLLSLYRHTSNQQGALVLVGVSEEIRDTMEITGFWNFFTACASMDEALRILGSESA
>3W08A 352 XRAY 1.80 0.158 0.189 no Aldoxime dehydratase <UNP Q7WSJ4_9PSED> [PSEUDOMONAS CHLORORAPHIS]
MESAIDTHLKCPRTLSRRVPEEYQPPFPMWVARADEQLQQVVMGYLGVQYRGEAQREAAL
QAMRHIVSSFSLPDGPQTHDLTHHTDSSGFDNLMVVGYWKDPAAHCRWLRSAEVNDWWTS
QDRLGEGLGYFREISAPRAEQFETLYAFQDNLPGVGAVMDSTSGEIEEHGYWGSMRDRFP
ISQTDWMKPTNELQVVAGDPAKGGRVVIMGHDNIALIRSGQDWADAEAEERSLYLDEILP
TLQDGMDFLRDNGQPLGCYSNRFVRNIDLDGNFLDVSYNIGHWRSLEKLERWAESHPTHL
RIFVTFFRVAAGLKKLRLYHEVSVSDAKSQVFEYINCHPHTGMLRDAVVAPT
>2GIYA 191 XRAY 1.78 0.198 0.220 no Glycoprotein E <UNP VGLE_HHV11> [HUMAN HERPESVIRUS 1]
GAPEVSHVRGVTVRMETPEAILFSPGETFSTNVSIHAIAHDDQTYSMDVVWLRFDVPTSC
AEMRIYESCLYHPQLPECLSPADAPCAASTWTSRLAVRSYAGCSRTNPPPRCSAEAHMEP
VPGLAWQAASVNLEFRDASPQHSGLYLCVVYVNDHIHAWGHITISTAAQYRNAVVEQPLD
IEGRGHHHHHH
>2XN6A 350 XRAY 1.29 0.209 0.235 no THYROXINE-BINDING GLOBULIN <UNP THBG_HUMAN> [HOMO SAPIENS]
SSQPNATLYKMSSINADFAFNLYRRFTVETPDKNIFFSPVSISAALVMLSFGACCSTQTE
IVETLGFNLTDTPMVEIQHGFQHLICSLNFPKKELELQIGNALFIGKHLKPLAKFLNDVK
TLYETEVFSTDFSNISAAKQEINSHVEMQTKGKVVGLIQDLKPNTIMVLVNYIHFKAQWA
NPFDPSKTEDSSSFLIDKTTTVQVPMMHQMDQYYHLVDMELNCTVLQMDYSKNALALFVL
PKEGQMESVEAAMSSKTLKKWNRLLQKGWVDLFVPKFSISATYDLGATLLKMGIQHAYSE
NADFSGLTEDNGLKLSNAAHKAVLHIGEKGTEAAGAMFLEAIPRSIPNTF
>2WMYA 150 XRAY 2.21 0.209 0.261 no PUTATIVE ACID PHOSPHATASE WZB <UNP Q9X4B8_ECOLX> [ESCHERICHIA COLI]
GAMAKLMFDSILVICTGNICRSPIGERLLRRLLPSKKINSAGVGALVDHTADESAIRVAE
KNGLCLKGHRGTKFTSALARQYDLLLVMEYSHLEQISRIAPEARGKTMLFGHWLDSKEIP DPYRMSDEAFDSVYQLLEQASKRWAEKLGE
>4J5RA 146 XRAY 1.25 0.120 0.153 no O-acetyl-ADP-ribose deacetylase 1 <UNP OARD1_HUMAN> [HOMO SAPIENS]
GSHMGSRITYVKGDLFACPKTDSLAHCISEDCRMGAGIAVLFKKKFGGVQELLNQQKKSG
EVAVLKRDGRYIYYLITKKRASHKPTYENLQKSLEAMKSHCLKNGVTDLSMPRIGCGLDR
LQWENVSAMIEEVFEATDIKITVYTL
>1RRMA 386 XRAY 1.60 0.193 0.211 no Lactaldehyde reductase <UNP FUCO_ECOLI> [ESCHERICHIA COLI]
MMANRMILNETAWFGRGAVGALTDEVKRRGYQKALIVTDKTLVQCGVVAKVTDKMDAAGL
AWAIYDGVVPNPTITVVKEGLGVFQNSGADYLIAIGGGSPQDTCKAIGIISNNPEFADVR
SLEGLSPTNKPSVPILAIPTTAGTAAEVTINYVITDEEKRRKFVCVDPHDIPQVAFIDAD
MMDGMPPALKAATGVDALTHAIEGYITRGAWALTDALHIKAIEIIAGALRGSVAGDKDAG
EEMALGQYVAGMGFSNVGLGLVHGMAHPLGAFYNTPHGVANAILLPHVMRYNADFTGEKY
RDIARVMGVKVEGMSLEEARNAAVEAVFALNRDVGIPPHLRDVGVRKEDIPALAQAALDD
VCTGGNPREATLEDIVELYHTAWEGG
>3HVZA 78 XRAY 2.20 0.226 0.254 no Uncharacterized protein <UNP A7VWX7_9CLOT> [CLOSTRIDIUM LEPTUM]
MDLAPEEVFVFTPKGDVISLPIGSTVIDFAYAIHSAVGNRMIGAKVDGRIVPIDYKVKTG
EIIDVLTTKELEHHHHHH
>1CFBA 205 XRAY 2.00 0.202 NA no DROSOPHILA NEUROGLIAN <UNP NRG_DROME> [DROSOPHILA MELANOGASTER]
IVQDVPNAPKLTGITCQADKAEIHWEQQGDNRSPILHYTIQFNTSFTPASWDAAYEKVPN
TDSSFVVQMSPWANYTFRVIAFNKIGASPPSAHSDSCTTQPDVPFKNPDNVVGQGTEPNN
LVISWTPMPEIEHNAPNFHYYVSWKRDIPAAAWENNNIFDWRQNNIVIADQPTFVKYLIK
VVAINDRGESNVAAEEVVGYSGEDR

>2V8TA 302 XRAY 0.98 0.101 0.117 no MANGANESE-CONTAINING PSEUDOCATALASE <UNP Q84DB4_THETH> [THERMUS THERMOPHILUS]
MFLRIDRLQIELPMPKEQDPNAAAAVQALLGGRFGEMSTLMNYMYQSFNFRGKKALKPYY
DLIANIATEELGHIELVAATINSLLAKNPGKDLEEGVDPASTPLGFAKDVRNAAHFIAGG
ANSLVMGAMGEHWNGEYVFTSGNLILDLLHNFFLEVAARTHKLRVYEMTDNPVAREMIGY
LLVRGGVHAAAYGKALESLTGVEMTKMLPIPKIDNSKIPEAKKYMDLGFHRNLYRFSPED
YRDLGLIWKGASPEDGTEVVVVDGPPTGGPVFDAGHDAAEFAPEFHPGELYEIAKKLYEK
AK
>2BWFA 77 XRAY 1.15 0.185 0.195 no UBIQUITIN-LIKE PROTEIN DSK2 <UNP DSK2_YEAST> [SACCHAROMYCES CEREVISIAE]
LDMSLNIHIKSGQDKWEVNVAPESTVLQFKEAINKANGIPVANQRLIYSGKILKDDQTVE
SYHIQDGHSVHLVKSQP
>3RNLA 311 XRAY 1.75 0.157 0.181 no Sulfotransferase <UNP C8WTE4_ALIAD> [ALICYCLOBACILLUS ACIDOCALDARIUS SUBSP. ACIDOCALDARIUS]
SNAMGVARPNFFIVGAAKCGTSSLDRYLSQHPDIYIPPKKEAHFFSIPDFPERFTGPGDE
GMNLYTIRDEDAYMRLFDGVRGERAVGEASVFYLFYPGTAQRMYDAYPDAKILIMLRNPV
DRAFSAYMHLVRDERETLSFRESLAKEEERIRQHYEPLWYYRAVGLYAAQVKRYLDVFGR
EQVKVILFEEFARDPVQVVRDCCAFLGVSTDFVPDTSIRHNESGVPKSRSLYNFIAKPNA
LKEIVKPFIPAAVRERLGNRAKSMVLGRMEMEPDLREELTAFFAPDVARLEALIHRDLSA
WRRPARAAGSQ
>4I0OA 497 XRAY 1.90 0.182 0.214 no Protein ELYS <UNP ELYS_MOUSE> [MUS MUSCULUS]
PGSMQDLTAQVTSDLLHFPEVTIEALGEDEITLESVLRGKFAAGKNGLACLACGPQLEVV
NSLTGERLSAYRFSGVNEQPPVVLAVKEFSWHKRTGLLIGLEEADGSVLCLYDLGISRVV
KAVVLPGRVTAIEPIINHGGASASTQHLHPSLRWLFGVAAVVTDVGQILLIDLCLDDLSC
SQNEVEASDLEVITGIPAEVPHIRERVMREGRHLCFQLVSPLGVAISTLSYINRTNQLAV
GFSDGYLALWNMKSMKREYYTQLEGGRVPVHAVAFQEPENDPRNCCYLWAVQSTQDSEGD
VLSLHLLQLAFGDRKCLASGQILYEGLEYCEERYTLDLAGGTFPLRGQTSNTKLLGCQSI
ERFPSHGDREESMREALSPDTSVSVFTWQVNIYGQGKPSVYLGLFDINRWYHAQMPDSLR
SGESLHNCSYFALWSLDSVVSRTSPHHILDILVHERSLNRGVPPSYPPPEQFFNPSTFNF
DATCLLDSGVIHVTCAG
>3T8JA 311 XRAY 1.60 0.157 0.170 no Purine nucleosidase, (IunH-1) <UNP Q97ZS5_SULSO> [SULFOLOBUS SOLFATARICUS]
MRHFIIDCDTAEDDVLSLYLLLKNNIDVVAVTIVEGNISYEQEVKNALWALEQVNREIPV
YPGANKPLLKNYITVEKVHGKGGIGDVTVEPKRLKAQEKHAALAIIDLANEYAGELEFLA
ISPLTNLALAYLLDNSIVKKIKKVWVMGGAVFGIGNITPVAEFNIWVDPDAAKIVFNAGF
DITMIPWDVIINYPVTDEEWNVIKNMKTRMSELYVSMYLHYRQYSSTVQKINGHPHPDAI
TTAIAIDGSIATRREKRFVVIDNTDNITRGMTLVDRFDADTSWSDKPNAEIVYEINKKSF
MEKIYDLLNWF
>2H1EA 177 XRAY 2.20 0.192 0.246 no Chromo domain protein 1 <UNP CHD1_YEAST> [SACCHAROMYCES CEREVISIAE]
MKKHHHHHHEDFHGIDIVINHRLKTSLEEGKVLEKTVPDLNNCKENYEFLIKWTDESHLH
NTWETYESIGQVRGLKRLDNYCKQFIIEDQQVRLDPYVTAEDIEIMDMERERRLDEFEEF

HVPERIIDSQRASLEDGTSQLQYLVKWRRLNYDEATWENATDIVKLAPEQVKHFQKK
>4E2VA 386 XRAY 1.18 0.139 0.153 no Queuine tRNA-ribosyltransferase <UNP TGT_ZYMMO> [ZYMOMONAS MOBILIS]
MVEATAQETDRPRFSFSIAAREGKARTGTIEMKRGVIRTPAFMPVGTAATVKALKPETVR
ATGADIILGNTYHLMLRPGAERIAKLGGLHSFMGWDRPILTDSGGFQVMSLSSLTKQSEE
GVTFKSHLDGSRHMLSPERSIEIQHLLGSDIVMAFDEVTPYPATPSRAASSMERSMRWAK
RSRDAFDSRKEQAENAALFGIQQGSVFENLRQQSADALAEIGFDGYAVGGLAVGEGQDEM
FRVLDFSVPMLPDDKPHYLMGVGKPDDIVGAVERGIDMFDCVLPTRSGRNGQAFTWDGPI
NIRNARFSEDLKPLDSECHCAVCQKWSRAYIHHLIRAGEILGAMLMTEHNIAFYQQLMQK
IRDSISEGRFSQFAQDFRARYFARNS
>1OTGA 125 XRAY 2.10 0.179 NA no 5-CARBOXYMETHYL-2-HYDROXYMUCONATE ISOMERASE <UNP HPCD_ECOLI> [ESCHERICHIA COLI]
PHFIVECSDNIREEADLPGLFAKVNPTLAATGIFPLAGIRSRVHWVDTWQMADGQHDYAF
VHMTLKIGAGRSLESRQQAGEMLFELIKTHFAALMESRLLALSFEIEELHPTLNFKQNNV
HALFK
>3G89A 249 XRAY 1.50 0.184 0.210 no Ribosomal RNA small subunit methyltransferase G <UNP RSMG_THET8> [THERMUS THERMOPHILUS]
MFHGKHPGGLSERGRALLLEGGKALGLDLKPHLEAFSRLYALLQEASGKVNLTALRGEEE
VVVKHFLDSLTLLRLPLWQGPLRVLDLGTGAGFPGLPLKIVRPELELVLVDATRKKVAFV
ERAIEVLGLKGARALWGRAEVLAREAGHREAYARAVARAVAPLCVLSELLLPFLEVGGAA
VAMKGPRVEEELAPLPPALERLGGRLGEVLALQLPLSGEARHLVVLEKTAPTPPAYPRRP
GVPERHPLC
>3IB7A 330 XRAY 1.60 0.218 0.177 no Icc protein <UNP O06629_MYCTU> [MYCOBACTERIUM TUBERCULOSIS]
GAMGIRNSKAYVEHRLRAAEHPRPDYVLLHISDTHLIGGDRRLYGAVDADDRLGELLEQL
NQSGLRPDAIVFTGDLADKGEPAAYRKLRGLVEPFAAQLGAELVWVMGNHDDRAELRKFL
LDEAPSMAPLDRVCMIDGLRIIVLDTSVPGHHHGEIRASQLGWLAEELATPAPDGTILAL
HHPPIPSVLDMAVTVELRDQAALGRVLRGTDVRAILAGHLHYSTNATFVGIPVSVASATC
YTQDLTVAAGGTRGRDGAQGCNLVHVYPDTVVHSVIPLGGGETVGTFVSPGQARRKIAES
GIFIEPSRRDSLFKHPPMVLTSSAPRSPVD
>4I5VA 333 XRAY 2.70 0.185 0.261 no 5',5'''-P-1,P-4-tetraphosphate phosphorylase 2 <UNP APA2_YEAST> [SACCHAROMYCES CEREVISIAE]
MGHHHHHHMIEENLKQKIHDKFVAAKKNGHLKVTHAESKKLKDPQTTTQYWVTFAPSLAL
KPDANKNSDSKAEDPFANPDEELVVTEDLNGDGEYKLLLNKFPVVPEHSLLVTSEFKDQR
SALTPSDLMTAYNVLCSLQGDKDDDVTCERYLVFYNCGPHSGSSQDHKALQIMQMPEKFI
PFQDVLCNGKDHFLPTFNAEPLQDDKVSFAHFVLPLPESSDQVDEDLLAMCYVSLMQRAL
TFFQDWTNESPELTKSYNVLLTKKWICVVPRSHAKSGPPLMLNINSTGYCGMILVKDREK
LENLTEDPHLVDKSLLQCGFPNTAGQKPTEYHY
>4J5TA 811 XRAY 2.04 0.226 0.235 no Mannosyl-oligosaccharide glucosidase <UNP CWH41_YEAST> [SACCHAROMYCES CEREVISIAE]
AEFMEEYQKFTNESLLWAPYRSNCYFGMRPRYVHESPLIMGIMWFNSLSQDGLHSLRHFA
TPQDKLQKYGWEVYDPRIGGKEVFIDEKNNLNLTVYFVKSKNGENWSVRVQGEPLDPKRP
STASVVLYFSQNGGEIDGKSSLAMIGHDGPNDMKFFGYSKELGEYHLTVKDNFGHYFKNP
EYETMEVAPGSDCSKTSHLSLQIPDKEVWKARDVFQSLVSDSIRDILEKEETKQRPADLI
PSVLTIRNLYNFNPGNFHYIQKTFDLTKKDGFQFDITYNKLGTTQSISTREQVTELITWS
LNEINARFDKQFSFGEGPDSIESVEVKRRFALETLSNLLGGIGYFYGNQLIDRETEFDES
QFTEIKLLNAKEEGPFELFTSVPSRGFFPRGFYWDEGFHLLQIMEYDFDLAFEILASWFE
MIEDDSGWIAREIILGNEARSKVPQEFQVQNPNIANPPTLLLAFSEMLSRAIENIGDFNS

DSYHQVMFNSRTAKFMTNNLEANPGLLTEYAKKIYPKLLKHYNWFRKSQTGLIDEYEEIL
EDEGIWDKIHKNEVYRWVGRTFTHCLPSGMDDYPRAQPPDVAELNVDALAWVGVMTRSMK
QIAHVLKLTQDEQRYAQIEQEVVENLDLLHWSENDNCYCDISIDPEDDEIREFVCHEGYV
SVLPFALKLIPKNSPKLEKVVALMSDPEKIFSDYGLLSLSRQDDYFGKDENYWRGPIWMN
INYLCLDAMRYYYPEVILDVAGEASNAKKLYQSLKINLSNNIYKVWEEQGYCYENYSPID
GHGTGAEHFTGWTALVVNILGRFRSHHHHHH
>2VJWA 149 XRAY 2.00 0.210 0.249 no GAF FAMILY PROTEIN <UNP A0R2U9_MYCS2> [MYCOBACTERIUM SMEGMATIS]
DPATVFRLVAAEALTLTGADGTLVAVPADPDASAAEELVIVEVAGAVPAEVEASAIPVQD
NAIGQAFRDRAPRRLDVLDGPGLGGPALVLPLRATDTVAGVLVAVQGSGARPFTAEQLEM
MTGFADQAAVAWQLASSQRRMSELDILAD
>2BM1A 691 XRAY 2.60 0.220 0.297 no ELONGATION FACTOR G <UNP EFG_THETH> [THERMUS THERMOPHILUS]
MAVKVEYDLKRLRNIVIAAHIDAGKTTTTERILYYTGRIHKIGEVHEGAATMDFMEQERE
RGITITAAVTTCFWKDHRINIIDTPGHVDFTIEVERSMRVLDGAIVVFDSSQGVEPQSET
VWRQAEKYKVPRIAFANKMDKTGADLWLVIRTMQERLGARPVVMQLPIGREDTFSGIIDV
LRMKAYTYGNDLGTDIREIPIPEEYLDQAREYHEKLVEVAADFDENIMLKYLEGEEPTEE
ELVAAIRKGTIDLKITPVFLGSALKNKGVQLLLDAVVDYLPSPLDIPPIKGTTPEGEVVE
IHPDPNGPLAALAFKIMADPYVGRLTFIRVYSGTLTSGSYVYNTTKGRKERVARLLRMHA
NHREEVEELKAGDLGAVVGLKETITGDTLVGEDAPRVILESIEVPEPVIDVAIEPKTKAD
QEKLSQALARLAEEDPTFRVSTHPETGQTIISGMGELHLEIIVDRLKREFKVDANVGKPQ
VAYRETITKPVDVEGKFIRQTGGRGQYGHVKIKVEPLPRGSGFEFVNAIVGGVIPKEYIP
AVQKGIEEAMQSGPLIGFPVVDIKVTLYDGSYHEVDSSEMAFKIAGSMAIKEAVQKGDPV
ILEPIMRVEVTTPEEYMGDVIGDLNARRGQILGMEPRGNAQVIRAFVPLAEMFGYATDLR
SKTQGRGSFVMFFDHYQEVPKQVQEKLIKGQ
>3RPJA 134 XRAY 1.90 0.184 0.211 no Curlin genes transcriptional regulator <UNP B4EUU9_PROMH> [PROTEUS MIRABILIS]
SNAMTSSLHPTRGKLLKRFAQIGPYIREQQCQESQFFFDCLAVCVNKKVTPEKREFWGWW
MELERNGEQLIYYYQVGLFDKNGDWVNQVISKKDVIESIHETLIRFHDFLQAAVSELEMT
LVPDEKMSNFPLPL
>1PL8A 356 XRAY 1.90 0.195 0.222 no human sorbitol dehydrogenase <UNP DHSO_HUMAN> [HOMO SAPIENS]
AAAAKPNNLSLVVHGPGDLRLENYPIPEPGPNEVLLRMHSVGICGSDVHYWEYGRIGNFI
VKKPMVLGHEASGTVEKVGSSVKHLKPGDRVAIEPGAPRENDEFCKMGRYNLSPSIFFCA
TPPDDGNLCRFYKHNAAFCYKLPDNVTFEEGALIEPLSVGIHACRRGGVTLGHKVLVCGA
GPIGMVTLLVAKAMGAAQVVVTDLSATRLSKAKEIGADLVLQISKESPQEIARKVEGQLG
CKPEVTIECTGAEASIQAGIYATRSGGTLVLVGLGSEMTTVPLLHAAIREVDIKGVFRYC
NTWPVAISMLASKSVNVKPLVTHRFPLEKALEAFETFKKGLGLKIMLKCDPSDQNP
>3T94A 270 XRAY 1.45 0.170 0.185 no 5'-methylthioadenosine phosphorylase (MtaP) <UNP Q97W94_SULSO> [SULFOLOBUS SOLFATARICUS]
MIEQNEKASIGIIGGSGLYDPGIFSESKEIKVYTPYGQPSDFITIGKIGNKSVAFLPRHG
RGHRIPPHKINYRANIWALKELGVRWVISVSAVGSLRMDYKLGDFVIPDQFIDMTKNREY
SFFDGPVVAHVSMADPFCNSLRKLAIETAKELNIKTHESGTYICIEGPRFSTRAESRTWR
EVYKADIIGMTLVPEVNLACEAQMCYATIAMVTDYDVFAEIPVTAEEVTRVMAENTEKAK
KLLYALIQKLPEKPEEGSCSCCNSLKTALV
>3N0AA 361 XRAY 2.20 0.173 0.200 no Tyrosine-protein phosphatase auxilin <UNP AUXI_BOVIN> [BOS TAURUS]
DTLKDTSSRVIQSVTSYTKGDLDFTYVTSRIIVMSFPLDSVDIGFRNQVDDIRSFLDSRH
LDHYTVYNLSPKSYRTAKFHSRVSECSWPIRQAPSLHNLFAVCRNMYNWLLQNPKNVCVV

HCLDGRAASSILVGAMFIFCNLYSTPGPAVRLLYAKRPGIGLSPSHRRYLGYMCDLLADK
PYRPHFKPLTIKSITVSPVPFFNKQRNGCRPYCDVLIGETKIYTTCADFERMKEYRVQDG
KIFIPLSITVQGDVVVSMYHLRSTIGSRLQAKVTNTQIFQLQFHTGFIPLDTTVLKFTKP
ELDACDVPEKYPQLFQVTLDVELQPHDKVMELTPPWEHYCTKDVNPSILFSSHQEHQDTL
V
>2AHRA 259 XRAY 2.15 0.175 0.210 no putative pyrroline carboxylate reductase <UNP Q9A1S9_STRP1>
[STREPTOCOCCUS PYOGENES]
SNAMKIGIIGVGKMASAIIKGLKQTPHELIISGSSLERSKEIAEQLALPYAMSHQDLIDQ
VDLVILGIKPQLFETVLKPLHFKQPIISMAAGISLQRLATFVGQDLPLLRIMPNMNAQIL
QSSTALTGNALVSQELQARVRDLTDSFGSTFDISEKDFDTFTALAGSSPAYIYLFIEALA
KAGVKNGIPKAKALEIVTQTVLASASNLKTSSQSPHDFIDAICSPGGTTIAGLMELERLG
LTATVSSAIDKTIDKAKSL
>3S2RA 83 XRAY 1.14 0.187 0.199 no AT5g51720/MIO24_14 <UNP Q9FLI7_ARATH> [ARABIDOPSIS THALIANA]
GSHMRKQQRMVVVRAEGGGGINPEIRKNEDKVVDSVVVTELSKNITPYCRCWRSGTFPLC
DGSCVKHNKANGDNVGPLLLKKQ
>3UP3A 243 XRAY 1.25 0.167 0.180 no aceDAF-12 <PDB 3UP3> [ANCYLOSTOMA CEYLANICUM]
GSYQLNAAELQALDLIQEAFKGMNDPMEQGRQATSFLKNEKSPADIMNIMDVTMRRFVKM
AKRLPAFNDLSQDGKFALLKGGMIEMLTVRGVRRFDSSSGSWTTPTLGESSEVSINMFDQ
LNADVRSEQKMRFLQFFKIFHEDIRSNDLVISMIMLIVLFSPRDSITDPEDRRIIARHHE
QFSALLNRYLESLYGDDAHQLNEQLPTALRMLREISASSGMLFLGTVNTSEAEPLPREFF
KVE
>2QJWA 176 XRAY 1.35 0.184 0.216 no Uncharacterized protein XCC1541 <UNP Q8PAE4_XANCP>
[XANTHOMONAS CAMPESTRIS PV. CAMPESTRIS]
GMSRGHCILAHGFESGPDALKVTALAEVAERLGWTHERPDFTDLDARRDLGQLGDVRGRL
QRLLEIARAATEKGPVVLAGSSLGSYIAAQVSLQVPTRALFLMVPPTKMGPLPALDAAAV
PISIVHAWHDELIPAADVIAWAQARSARLLLVDDGHRLGAHVQAASRAFAELLQSL
>3EATX 293 XRAY 2.50 0.189 0.225 no Pyoverdine biosynthesis protein PvcB <UNP Q9I1L4_PSEAE>
[PSEUDOMONAS AERUGINOSA]
GHMNAYLSDQPVRLSPLRDEQGNQPRFGLLLEPGRPGMHVGELPAQWLKGLARSHHLLLL
RGFAAFADAESLTRYCHDFGEVMLWPFGAVLELVEQEGAEDHIFANNYVPLHWDGMYLET
VPEFQVFHCVDAPGDSDGGRTTFSSTPAALQLADSSELELWRRASGRYQRSAAHYSSRSA
APIVERHPRREFPILRFCEPPVEGDASFINPSEFHYDGIAPEQRGELLASLRRCLYHPQA
HYAHRWRSDDLVIADNLTLLHGREAFAHRAPRHLRRVHIHAEPALRNPHLQRD
>1ZUPA 315 XRAY 2.20 0.230 0.266 no hypothetical protein TM1739 <UNP Q9X261_THEMA>
[THERMOTOGA MARITIMA]
MGSDKIHHHHHHMQVRIERAERIESELEEHVGDQTFVEESRFLEEDEQREGEILDQIIFV
DGKRRSFVRITTDEGITGIFAELCVGAVIWDREGGTKTLFSPDKPPVKERVLGFSQSFQE
EGYEEVGGILFKVVKEGKDAMQSIDLYMRSLEIEEVRKHMDKNILIVKDGPAARELPFEE
NVGPIGLVKNIGVTELSKEDFKKLRFLKKGKRSKMFVSSRETPLKKVGAYVKLIDGEGIR
GLVRLETYVKDDNQIPYIRKVFDDLAKTLPHLTADLPIPRLPENILPIQFLEENLSYYLT
DKNYMNTRLFAYIGR
>3JYBA 145 XRAY 2.04 0.201 0.235 no Sensor protein <UNP Q9HUV7_PSEAE> [PSEUDOMONAS AERUGINOSA]
ATTPSANQNWRLLRDESAQLRIADVLQRKEQFRPLAKRSFIFPASPQAVWLQVQLPAQKV
PSWLWIFAPRVQYLDYYLVQDGQLVRDQHTGESRPFQERPLPSRSYLFSLPVDGKPMTLY
VRMTSNHPLMAWFDQIDEAGLVGLE
>2XEPA 458 XRAY 1.50 0.201 0.217 no ORF12 <UNP Q83Z62_STRCL> [STREPTOMYCES CLAVULIGERUS]

MMKKADSVPTPAEAALAAQTALAADDSPMGDAARWAMGLLTSSGLPRPEDVAARFIPTFA
AAGNFAETVREWRSKGPFTVRAYHPVAHKGWVVLSAPAGVRYILSLTLDSSGLIRILTLK
PETVIPDMVTWNDVEETLHTPGVQHSVYAVRLTPDGHEVLHASAPERPMPTGSAYKLYLM
RALVAEIEKGTVGWDEILTLTPELRSLPTGDMQDLPDGTRVTVRETAHKMIALSDNTGAD
LVADRLGREVVERSLAAAGHHDPSLMRPFLTSHEVFELGWGDPERRAEWVRQDEAGRREL
LEKMAGVMTVRGSDLGATVHQLGIDWHMDAFDVVRVLEGLLQDSGRDTSGTVEEILTAYP
GLLIDEERWRRVYFKAGSSPGVMMFCWLLQDHAGISYVLVLRQSADEQRLIGDGLFLRGI
GAKIIEAEAKLLSSGERRGAGTAAAGDDRASAGEAARR
>1VH5A 148 XRAY 1.34 0.188 0.228 no Hypothetical protein ydiI <UNP YDII_ECOLI> [ESCHERICHIA COLI]
MSLIWKRKITLEALNAMGEGNMVGFLDIRFEHIGDDTLEATMPVDSRTKQPFGLLHGGAS
VVLAESIGSVAGYLCTEGEQKVVGLEINANHVRSAREGRVRGVCKPLHLGSRHQVWQIEI
FDEKGRLCCSSRLTTAILEGGSHHHHHH
>2QXFA 482 XRAY 1.50 0.200 0.224 no Exodeoxyribonuclease I <UNP EX1_ECOLI> [ESCHERICHIA COLI]
MMNDGKQQSTFLFHDYETFGTHPALDRPAQFAAIRTDSEFNVIGEPEVFYCKPADDYLPQ
PGAVLITGITPQEARAKGENEAAFAARIHSLFTVPKTCILGYNNVRFDDEVTRNIFYRNF
YDPYAWSWQHDNSRWDLLDVMRACYALRPEGINWPENDDGLPSFRLEHLTKANGIEHSNA
HDAMADVYATIAMAKLVKTRQPRLFDYLFTHRNKHKLMALIDVPQMKPLVHVSGMFGAWR
GNTSWVAPLAWHPENRNAVIMVDLAGDISPLLELDSDTLRERLYTAKTDLGDNAAVPVKL
VHINKCPVLAQANTLRPEDADRLGINRQHCLDNLKILRENPQVREKVVAIFAEAEPFTPS
DNVDAQLYNGFFSDADRAAMKIVLETEPRNLPALDITFVDKRIEKLLFNYRARNFPGTLD
YAEQQRWLEHRRQVFTPEFLQGYADELQMLVQQYADDKEKVALLKALWQYADEIVEHHHH
HH
>2IP2A 334 XRAY 1.80 0.196 0.247 no Probable phenazine-specific methyltransferase <UNP Q9HWH2_PSEAE> [PSEUDOMONAS AERUGINOSA]
MNNSNLAAARNLIQVVTGEWKSRCVYVATRLGLADLIESGIDSDETLAAAVGSDAERIHR
LMRLLVAFEIFQGDTRDGYANTPTSHLLRDVEGSFRDMVLFYGEEFHAAWTPACEALLSG
TPGFELAFGEDFYSYLKRCPDAGRRFLLAMKASNLAFHEIPRLLDFRGRSFVDVGGGSGE
LTKAILQAEPSARGVMLDREGSLGVARDNLSSLLAGERVSLVGGDMLQEVPSNGDIYLLS
RIIGDLDEAASLRLLGNCREAMAGDGRVVVIERTISASEPSPMSVLWDVHLFMACAGRHR
TTEEVVDLLGRGGFAVERIVDLPMETRMIVAARA
>2HQ9A 149 XRAY 1.95 0.183 0.243 no Mll6688 protein <UNP Q988L5_RHILO> [MESORHIZOBIUM LOTI]
GMLVRTLSALECTKVLTANRVGRLACAKDGQPYVVPLYYAYSDAHLYAFSMPGKKIEWMR
ANPRVSVQVDEHGQGRGWKSVVVDGRYEELPDLIGHKLQRDHAWSVLSKHTDWWEPGALK
PVTPPTADSAPHVFFRILIEQVSGREASE
>2HAZA 105 XRAY 1.70 0.219 0.286 no Neural cell adhesion molecule 1 <UNP NCA12_HUMAN> [HOMO SAPIENS]
GSHMDTPSSPSIDQVEPYSSTAQVQFDEPEATGGVPILKYKAEWRAVGEEVWHSKWYDAK
EASMEGIVTIVGLKPETTYAVRLAALNGKGLGEISAASEFKTQPV
>2VSOE 284 XRAY 2.60 0.215 0.260 no EUKARYOTIC INITIATION FACTOR 4F SUBUNIT P150 <UNP IF4F1_YEAST> [SACCHAROMYCES CEREVISIAE]
MLVPSANRWVPKFKSKKTEKKLAPDGKTELLDKDEVERKMKSLLNKLTLEMFDAISSEIL
AIANISVWETNGETLKAVIEQIFLKACDEPHWSSMYAQLCGKVVKELNPDITDETNEGKT
GPKLVLHYLVARCHAEFDKGWTDKLPTNEDGTPLEPEMMSEEYYAAASAKRRGLGLVRFI
GFLYRLNLLTGKMMFECFRRLMKDLTDSPSEETLESVVELLNTVGEQFETDSFRTGQATL
EGSQLLDSLFGILDNIIQTAKISSRIKFKLIDIKELRHDKNWNS
>1H12A 405 XRAY 1.20 0.108 0.130 no ENDO-1,4-BETA-XYLANASE <UNP Q8RJN8> [PSEUDOALTEROMONAS HALOPLANKTIS]
AFNNNPSSVGAYSSGTYRNLAQEMGKTNIQQKVNSTFDNMFGYNNTQQLYYPYTENGVYK

AHYIKAINPDEGDDIRTEGQSWGMTAAVMLNKQEEFDNLWRFAKAYQKNPDNHPDAKKQG
VYAWKLKLNQNGFVYKVDEGPAPDGEEYFAFALLNASARWGNSGEFNYYNDAITMLNTIK
NKLMENQIIRFSPYIDNLTDPSYHIPAFYDYFANNVTNQADKNYWRQVATKSRTLLKNHF
TKVSGSPHWNLPTFLSRLDGSPVIGYIFNGQANPGQWYEFDAWRVIMNVGLDAHLMGAQA
WHKSAVNKALGFLSYAKTNNSKNCYEQVYSYGGAQNRGCAGEGQKAANAVALLASTNAGQ
ANEFFNEFWSLSQPTGDYRYYNGSLYMLAMLHVSGNFKFYNNTFN
>1VLYA 338 XRAY 1.30 0.136 0.168 no Unknown protein from 2D-page <UNP UP14_ECOLI> [ESCHERICHIA COLI]
MGSDKIHHHHHHMAFTPFPPRQPTASARLPLTLMTLDDWALATITGADSEKYMQGQVTAD
VSQMAEDQHLLAAHCDAKGKMWSNLRLFRDGDGFAWIERRSVREPQLTELKKYAVFSKVT
IAPDDERVLLGVAGFQARAALANLFSELPSKEKQVVKEGATTLLWFEHPAERFLIVTDEA
TANMLTDKLRGEAELNNSQQWLALNIEAGFPVIDAANSGQFIPQATNLQALGGISFKKGC
YTGQEMVARAKFRGANKRALWLLAGSASRLPEAGEDLELKMGENWRRTGTVLAAVKLEDG
QVVVQVVMNNDMEPDSIFRVRDDANTLHIEPLPYSLEE
>2X1QA 127 XRAY 1.06 0.214 0.229 no GELSOLIN NANOBODY <PDB 2X1Q> [LAMA GLAMA]
QVQLQESGGGLVQPGGSLRLSCAASGRTFSNYRMGWFRQAPGKEREFVATISQSGAATAY
ADSVKGRFTFSRDNAKNLLYLEMLSLEPEDTAVYYCAASSRVFYTEVLQTTTGYDYWGQG
TQVTVSS
>3GYBA 280 XRAY 1.60 0.219 0.231 no Transcriptional regulators (LACI-FAMILY TRANSCRIPTIONAL REGULATORY PROTEIN) <UNP Q8NTY1_CORGL> [CORYNEBACTERIUM GLUTAMICUM]
MSLRTQLIAVLIDDYSNPWFIDLIQSLSDVLTPKGYRLSVIDSLTSQAGTDPITSALSMR
PDGIIIAQDIPDFTVPDSLPPFVIAGTRITQASTHDSVANDDFRGAEIATKHLIDLGHTH
IAHLRVGSGAGLRRFESFEATMRAHGLEPLSNDYLGPAVEHAGYTETLALLKEHPEVTAI
FSSNDITAIGALGAARELGLRVPEDLSIIGYDNTPLAQTRLINLTTIDDNSIGVGYNAAL
LLLSMLDPEAPHPEIMHTLQPSLIERGTCAPREGHHHHHH
>3BA3A 145 XRAY 1.55 0.171 0.196 no Pyridoxamine 5'-phosphate oxidase-like protein <UNP Q890D6_LACPL> [LACTOBACILLUS PLANTARUM WCFS1]
GMDISLLKQVVQSTNKIALSTAVNNEADVKIVNFVWYEAQPDTLYFSSVKTSPALKVYDQ
NPDIAFITIPNDGTAGNPYLRAQHVKLQRSTKTMTDLLPQYLETVPNYQQVWDAIGSTLV
VFELKLTDLFVDAGVGGEKQTLTFN
>3H6QA 169 XRAY 1.64 0.162 0.193 no Macrocypin 1a <UNP B9V973_9AGAR> [MACROLEPIOTA PROCERA]
MGFEDGFYTILHLAEGQHPNSKIPGGMYASSKDGKDVPVTAEPLGPQSKIRWWIARDPQA
GDDMYTITEFRIDNSIPGQWSRSPVETEVPVYLYDRIKAEETGYTCAWRIQPADHGADGV
YHIVGNVRIGSTDWADLREEYGEPQVYMKPVPVIPNVYIPRWFILGYEE
>3L7IA 729 XRAY 2.70 0.213 0.260 no Teichoic acid biosynthesis protein F <UNP Q5HLM5_STAEQ> [STAPHYLOCOCCUS EPIDERMIDIS]
MNKLTIIVTYYNAEEYITGCLESIKQQRTQDFNLIIVNDGSTDQSKKLMDEAIKDYDKNI
RFIDLDENSGHAHARNIALEEVETPYFMFLDADDELASYAITFYLEKFNNTDGLIAPIHS
FTTQRPQFVDLDRVRVEYFNAKENINSFLRKQSACNIIFRTAIVRAHHIRFNENLNTYVD
WSFVLEYMKYVNKFVRIFNFPFYFRGEVYDPFETLTLSEQNFDILFKDYVNSFYDAIKRA
TNPKVREFIVTKMGNKIANEFEPTRYDINERYQTHKDTLVELSKFLHVHLVKNQKLINKI
ETILLMNNETDKAFKVNQFRKTLRHVKNIVLRRKNKERSLYDLTDKEDNVKPKTIVFESF
GGKNYSDSPKYIYEYMQKYYPNYRYIWSFKNPDKNVVPGSAEKVKRNSAEYYQAYSEASH
WVSNARTPLYLNKKENQTYIQTWHGTPLKRLANDMKVVRMPGTTTPKYKRNFNRETSRWD
YLISPNRYSTEIFRSAFWMDEERILEIGYPRNDVLVNRANDQEYLDEIRTHLNLPSDKKV
IMYAPTWRDDEFVSKGKYLFELKIDLDNLYKELGDDYVILLRMHYLISNALDLSGYENFA
IDVSNYNDVSELFLISDCLITDYSSVMFDYGILKRPQFFFAYDIDKYDKGLRGFYMNYME
DLPGPIYTEPYGLAKELKNLDKVQQQYQEKIDAFYDRFCSVDNGKASQYIGDLIHKDIKE
QLEHHHHHH

>1HI9A 274 XRAY 2.40 0.232 0.268 no DIPEPTIDE TRANSPORT PROTEIN DPPA <UNP DPPA_BACSU> [BACILLUS SUBTILIS]
MKLYMSVDMEGISGLPDDTFVDSGKRNYERGRLIMTEEANYCIAEAFNSGCTEVLVNDSH
SKMNNLMVEKLHPEADLISGDVKPFSMVEGLDDTFRGALFLGYHARASTPGVMSHSMIFG
VRHFYINDRPVGELGLNAYVAGYYDVPVLMVAGDDRAAKEAEELIPNVTTAAVKQTISRS
AVKCLSPAKRGRLLTEKTAFALQNKDKVKPLTPPDRPVLSIEFANYGQAEWANLMPGTEI
KTGTTTVQFQAKDMLEAYQAMLVMTELAMRTSF
>4EOJB 258 XRAY 1.65 0.197 0.215 no Cyclin-A2 <UNP CCNA2_HUMAN> [HOMO SAPIENS]
VPDYHEDIHTYLREMEVKCKPKVGYMKKQPDITNSMRAILVDWLVEVGEEYKLQNETLHL
AVNYIDRFLSSMSVLRGKLQLVGTAAMLLASKFEEIYPPEVAEFVYITDDTYTKKQVLRM
EHLVLKVLTFDLAAPTVNQFLTQYFLHQQPANCKVESLAMFLGELSLIDADPYLKYLPSV
IAGAAFHLALYTVTGQSWPESLIRKTGYTLESLKPCLMDLHQTYLKAPQHAQQSIREKYK
NSKYHGVSLLNPPETLNL
>1J5PA 253 XRAY 1.90 0.224 0.260 no ASPARTATE DEHYDROGENASE <UNP Q9X1X6_THEMA> [THERMOTOGA MARITIMA]
MGSDKIHHHHHHMTVLIIGMGNIGKKLVELGNFEKIYAYDRISKDIPGVVRLDEFQVPSD
VSTVVECASPEAVKEYSLQILKNPVNYIIISTSAFADEVFRERFFSELKNSPARVFFPSG
AIGGLDVLSSIKDFVKNVRIETIKPPKSLGLDLKGKTVVFEGSVEEASKLFPRNINVAST
IGLIVGFEKVKVTIVADPAMDHNIHIVRISSAIGNYEFKIENIPSPENPKTSMLTVYSIL
RTLRNLESKIIFG
>3CU5A 141 XRAY 2.60 0.189 0.300 no Two component transcriptional regulator, AraC family <UNP A9KIW7_CLOPH> [CLOSTRIDIUM PHYTOFERMENTANS ISDG]
MSLRILIVDDEKLTRDGLIANINWKALSFDQIDQADDGINAIQIALKHPPNVLLTDVRMP
RMDGIELVDNILKLYPDCSVIFMSGYSDKEYLKAAIKFRAIRYVEKPIDPSEIMDALKQS
IQTVLQHQAQQDSEGHHHHHH
>1QWOA 442 XRAY 1.50 0.162 0.186 no phytase <UNP PHYA_ASPFU> [ASPERGILLUS FUMIGATUS]
SAGSKSCDTVDLGYQCSPATSHLWGQYSPFFSLEDELSVSSKLPKDCRITLVQVLSRHGA
RYPTSSKSKKYKKLVTAIQANATDFKGKFAFLKTYNYTLGADDLTPFGEQQLVNSGIKFY
QRYKALARSVVPFIRASGSDRVIASGEKFIEGFQQAKLADPGATNRAAPAISVIIPESET
FNNTLDHGVCTKFEASQLGDEVAANFTALFAPDIRARAEKHLPGVTLTDEDVVSLMDMCS
FDTVARTSDASQLSPFCQLFTHNEWKKYNYLQSLGKYYGYGAGNPLGPAQGIGFTNELIA
RLTRSPVQDHTSTNSTLVSNPATFPLNATMYVDFSHDNSMVSIFFALGLYNGTEPLSRTS
VESAKELDGYSASWVVPFGARAYFETMQCKSEKEPLVRALINDRVVPLHGCDVDKLGRCK
LNDFVKGLSWARSGGNWGECFS
>5CSMA 256 XRAY 2.00 0.186 0.236 no CHORISMATE MUTASE <UNP CHMU_YEAST> [SACCHAROMYCES CEREVISIAE]
MDFTKPETVLNLQNIRDELVRMEDSIIFKFIERSHFATCPSVYEANHPGLEIPNFKGSFL
DWALSNLEIAHSRIRRFESPDETPFFPDKIQKSFLPSINYPQILAPYAPEVNYNDKIKKV
YIEKIIPLISKRDGDDKNNFGSVATRDIECLQSLSRRIHFGKFVAEAKFQSDIPLYTKLI
KSKDVEGIMKNITNSAVEEKILERLTKKAEVYGVDPTERRIERRISPEYLVKIYKEIVIP
ITKEVEVEYLLRRLEE
>3U12A 141 XRAY 2.08 0.210 0.232 no USP37 protein <UNP Q86W68_HUMAN> [HOMO SAPIENS]
MGSSHHHHHHSSGLVPRGSLKIHGPIRIRSMQTGITKWKEGSFEIVEKENKVSLVVHYNT
GGIPRIFQLSHNIKNVVLRPSGAKQSRLMLTLQDNSFLSIDKVPSKDAEEMRLFLDAVHQ
NRLPAAMKPSQGSGSFGAILG
>3ABHA 312 XRAY 2.00 0.234 0.271 no Protein kinase C and casein kinase substrate in neurons protein 2
<UNP PACN2_HUMAN> [HOMO SAPIENS]
GSSGSSGMSVTYDDSVGVEVSSDSFWEVGNYKRTVKRIDDGHRLCSDLMNCLHERARIEK

AYAQQLTEWARRWRQLVEKGPQYGTVEKAWMAFMSEAERVSELHLEVKASLMNDDFEKIK
NWQKEAFHKQMMGGFKETKEAEDGFRKAQKPWAKKLKEVEAAKKAHHAACKEEKLAISRE
ANSKADPSLNPEQLKKLQDKIEKCKQDVLKTKEKYEKSLKELDQGTPQYMENMEQVFEQC
QQFEEKRLRFFREVLLEVQKHLDLSNVAGYKAIYHDLEQSIRAADAVEDLRWFRANHGPG
MAMNWPQFEEWS
>3C0FB 91 XRAY 1.80 0.207 0.226 no Uncharacterized protein AF_1514 <UNP Y1514_ARCFU> [ARCHAEOGLOBUS FULGIDUS DSM 4304]
MEIMDEIKVNLQKEVSLEEAERYAKNIASKYGDGILLSVHDSKTGYRAPEVYCCGEKPWE
VYACNRGANLKISVNQFEFYFRIEVEGQAKY
>1Z6RA 406 XRAY 2.70 0.206 0.263 no Mlc protein <UNP MLC_ECOLI> [ESCHERICHIA COLI] MVAENQPGHIDQIKQTNAGAVYRLIDQLGPVSRIDLSRLAQLAPASITKIVHEMLEAHLV
QELEIKEAGNRGRPAVGLVVETEAWHYLSLRISRGEIFLALRDLSSKLVVEESQELALKD
DLPLLDRIISHIDQFFIRHQKKLERLTSIAITLPGIIDTENGIVHRMPFYEDVKEMPLGE
ALEQHTGVPVYIQHDISAWTMAEALFGASRGARDVIQVVIDHNVGAGVITDGHLLHAGSS
SLVEIGHTQVDPYGKRCYCGNHGCLETIASVDSILELAQLRLNQSMSSMLHGQPLTVDSL
CQAALRGDLLAKDIITGVGAHVGRILAIMVNLFNPQKILIGSPLSKAADILFPVISDSIR
QQALPAYSQHISVESTQFSNQGTMAGAALVKDAMYNGSLLIRLLQG
>3P1UA 529 XRAY 2.05 0.244 0.265 no SusD homolog <UNP A6L9L2_PARD8> [PARABACTEROIDES DISTASONIS ATCC 8503]
GNENPDKPTDDVNYNMNEPRLASTLRGGLIIEGNVEQRLKPLQIDFYSQMTVDGGGWGTK
NYIQDDEWNNLVWEEYLKQIASINIVIRSLTEKDKDAYANTIAFARIWRVYVHTLAADKF
GPMPFPAYEIVEANPPYKSLKDIYDEYFRELDAAINGFNDSAQPIFSDAGIDLIYKNDVS
KWKRFANSLRLRLAVRLTEVDQEKCIAEANAAISSPAGLISDKADNAYMPPKADGSWGQD
YNYTMFQITWSGPICMSKSVEKLVTNIGGVAWPQGVVNQTSGVAVSSVHPEKVDPRAPKI
FQPGIENGDWKGLVYGPKAEEANTGIYQSKQCAELGFIIKDGYPYKSRPYDLFLSEEVHF
LKAELYARGFIAGDAKSEYEAGVRASFATWGVTSEVDDYLTSTEKNEAGTSARYDDQQGA
GNTALEKIITQKYIAGIPDLAQEGWNDKRRLNLPRLDVAVYRDQAVYNNNDKDILKSANF
IKRMRYPTKESLINATEYEKGKSMLGGKGDIVSTPLWWDKNSNYCTSSK
>2OZVA 260 XRAY 1.70 0.180 0.214 no Hypothetical protein Atu0636 <UNP Q8UHP4_AGRT5> [AGROBACTERIUM TUMEFACIENS STR.]
MGSSHHHHHHSSGRENLYFQGHMDAMLLASLVADDRACRIADLGAGAGAAGMAVAARLEK
AEVTLYERSQEMAEFARRSLELPDNAAFSARIEVLEADVTLRAKARVEAGLPDEHFHHVI
MNPPYNDAGDRRTPDALKAEAHAMTEGLFEDWIRTASAIMVSGGQLSLISRPQSVAEIIA
ACGSRFGGLEITLIHPRPGEDAVRMLVTAIKGSRARLTFRAPLIMHETGSHAFTPFVDDL
NNGRAAYARNVRAIRTASGS
>1DJ8A 89 XRAY 2.00 0.209 0.261 no PROTEIN HNS-DEPENDENT EXPRESSION A <UNP HDEA_ECOLI> [ESCHERICHIA COLI]
ADAQKAADNKKPVNSWTCEDFLAVDESFQPTAVGFAEALNNKDKPEDAVLDVQGIATVTP
AIVQACTQDKQANFKDKVKGEWDKIKKDM
>2ZE7A 253 XRAY 2.10 0.195 0.254 no Isopentenyl transferase <UNP IPTZ_AGRT5> [AGROBACTERIUM TUMEFACIENS]
MLLHLIYGPTCSGKTDMAIQIAQETGWPVVALDRVQCCPQIATGSGRPLESELQSTRRIY
LDSRPLTEGILDAESAHRRLIFEVDWRKSEEGLILEGGSISLLNCMAKSPFWRSGFQWHV
KRLRLGDSDAFLTRAKQRVAEMFAIREDRPSLLEELAELWNYPAARPILEDIDGYRCAIR
FARKHDLAISQLPNIDAGRHVELIEAIANEYLEHALSQERDFPQWPEDGAGQPVCPVTLT
RIRGSRSHHHHHH
>2W3GA 153 XRAY 1.40 0.193 0.217 no TWO COMPONENT SENSOR HISTIDINE KINASE DEVS (GAF FAMILY PROTEIN) <UNP P95194_MYCTU> [MYCOBACTERIUM TUBERCULOSIS]

GAMDPDLEATLRAIVHSATSLVDARYGAMEVHDRQHRVLHFVYEGIDEETVRRIGHLPKG
LGVIGLLIEDPKPLRLDDVSAHPASIGFPPYHPPMRTFLGVPVRVRDESFGTLYLTDKTN
GQPFSDDDEVLVQALAAAAGIAVANARLYQQAK
>1OOOA 147 XRAY 1.85 0.215 0.262 no Mago nashi protein <UNP MGN_DROME> [DROSOPHILA MELANOGASTER]
MSTEDFYLRYYVGHKGKFGHEFLEFEFRPDGKLRYANNSNYKNDTMIRKEAFVHQSVMEE
LKRIIIDSEIMQEDDLPWPPPDRVGRQELEIVIGDEHISFTTSKTGSLVDVNRSKDPEGL
RCFYYLVQDLKCLVFSLIGLHFKIKPI
>1BS0A 384 XRAY 1.65 0.178 0.212 no 8-AMINO-7-OXONANOATE SYNTHASE <UNP BIOF_ECOLI> [ESCHERICHIA COLI]
MSWQEKINAALDARRAADALRRRYPVAQGAGRWLVADDRQYLNFSSNDYLGLSHHPQIIR
AWQQGAEQFGIGSGGSGHVSGYSVVHQALEEELAEWLGYSRALLFISGFAANQAVIAAMM
AKEDRIAADRLSHASLLEAASLSPSQLRRFAHNDVTHLARLLASPCPGQQMVVTEGVFSM
DGDSAPLAEIQQVTQQHNGWLMVDDAHGTGVIGEQGRGSCWLQKVKPELLVVTFGKGFGV
SGAAVLCSSTVADYLLQFARHLIYSTSMPPAQAQALRASLAVIRSDEGDARREKLAALIT
RFRAGVQDLPFTLADSCSAIQPLIVGDNSRALQLAEKLRQQGCWVTAIRPPTVPAGTARL
RLTLTAAHEMQDIDRLLEVLHGNG
>1VQOT 120 XRAY 2.20 0.215 0.246 no 50S ribosomal protein L24P <UNP RL24_HALMA> [HALOARCULA MARISMORTUI]
MSKQPDKQRKSQRRAPLHERHKQVRATLSADLREEYGQRNVRVNAGDTVEVLRGDFAGEE
GEVINVDLDKAVIHVEDVTLEKTDGEEVPRPLDTSNVRVTDLDLEDEKREARLESEDDSA
>1VR7A 142 XRAY 1.20 0.120 0.150 no S-adenosylmethionine decarboxylase proenzyme <UNP SPEH_THEMA> [THERMOTOGA MARITIMA]
MGSDKIHHHHHHMKSLGRHLVAEFYECDREVLDNVQLIEQEMKQAAYESGATIVTSTFHR
FLPYGVSGVVVISESHLTIHTWPEYGYAAIDLFTCGEDVDPWKAFEHLKKALKAKRVHVV
EHERGRYDEIGIPEDSPHKAAV
>2ID6A 202 XRAY 1.75 0.211 0.251 no TRANSCRIPTIONAL REGULATOR, TetR FAMILY <UNP Q9X0C0_THEMA> [THERMOTOGA MARITIMA]
GHMLSKRDAILKAAVEVFGKKGYDRATTDEIAEKAGVAKGLIFHYFKNKEELYYQAYMSV
TEKLQKEFENFLMKNRNRDIFDFMERWIEKKLEYSASHPEEADFLITLVSVDEGLRKRIL
LDLEKSQRVFFDFVREKLKDLDLAEDVTEEIALKFLMWFFSGFEEVYLRTYQGKPELLKR
DMNTLVEEVKVMLRILKKGMTK
>1GNYA 153 XRAY 1.63 0.152 0.177 no XYLANASE 10C <UNP Q59675> [PSEUDOMONAS CELLULOSA]
GNVVIEVDMANGWRGNASGSTSHSGITYSADGVTFAALGDGVGAVFDIARPTTLEDAVIA
MVVNVSAEFKASEANLQIFAQLKEDWSKGEWDCLAGSSELTADTDLTLTCTIDEDDDKFN
QTARDVQVGIQAKGTPAGTITIKSVTITLAQEA
>4FMRA 265 XRAY 2.25 0.171 0.213 no uncharacterized hypothetical protein <UNP A6L2B2_BACV8> [BACTEROIDES VULGATUS]
GAKNVLKAWLVDNTVTTDDKTDKIFQLETTRSIDKEIILDRMVAKNPGVRRETMALGIEL
MEEVVAEALMNGESVNTGLFRGVAQFRGVAKQNAWDAATNSIYVSLTQGKALREAIKDTR
VDVLGERPTKFYIGSGQDATTRATDFSATAGRNFTLFGKNLTVAGTDPSVGVTLASAATG
TVTKIDNDMIVLNEPSRLIILLPASLEDGEYMLTVTTQYRGGGGALLKTPRSTSHTIYIG
GAPETGGSTGPPGDSDGDLNENPLG
>1CC5A 83 XRAY 2.50 0.290 NA no CYTOCHROME C5 <UNP CYC5_AZOVI> [AZOTOBACTER VINELANDII]
GGGARSGDDVVAKYCNACHGTGLLNAPKVGDSAAWKTRADAKGGLDGLLAQSLSGLNAMP
PKGTCADCSDDELKAAIGKMSGL
>2GJ4A 824 XRAY 1.60 0.189 0.226 no Glycogen phosphorylase, muscle form <UNP PYGM_RABIT> [ORYCTOLAGUS CUNICULUS]
QISVRGLAGVENVTELKKNFNRHLHFTLVKDRNVATPRDYYFALAHTVRDHLVGRWIRTQ

QHYYEKDPKRIYYLSLEFYMGRTLQNTMVNLALENACDEATYQLGLDMEELEEIEEDAGL
GNGGLGRLAACFLDSMATLGLAAYGYGIRYEFGIFNQKICGGWQMEEADDWLRYGNPWEK
ARPEFTLPVHFYGRVEHTSQGAKWVDTQVVLAMPYDTPVPGYRNNVVNTMRLWSAKAPND
FNLKDFNVGGYIQAVLDRNLAENISRVLYPNDNFFEGKELRLKQEYFVVAATLQDIIRRF
KSSKFGCRDPVRTNFDAFPDKVAIQLNDTHPSLAIPELMRVLVDLERLDWDKAWEVTVKT
CAYTNHTVLPEALERWPVHLLETLLPRHLQIIYEINQRFLNRVAAAFPGDVDRLRRMSLV
EEGAVKRINMAHLCIAGSHAVNGVARIHSEILKKTIFKDFYELEPHKFQNKTNGITPRRW
LVLCNPGLAEIIAERIGEEYISDLDQLRKLLSYVDDEAFIRDVAKVKQENKLKFAAYLER
EYKVHINPNSLFDVQVKRIHEYKRQLLNCLHVITLYNRIKKEPNKFVVPRTVMIGGKAAP
GYHMAKMIIKLITAIGDVVNHDPVVGDRLRVIFLENYRVSLAEKVIPAADLSEQISTAGT
EASGTGNMKFMLNGALTIGTMDGANVEMAEEAGEENFFIFGMRVEDVDRLDQRGYNAQEY
YDRIPELRQIIEQLSSGFFSPKQPDLFKDIVNMLMHHDRFKVFADYEEYVKCQERVSALY
KNPREWTRMVIRNIATSGKFSSDRTIAQYAREIWGVEPSRQRLP
>2RBFA 54 XRAY 2.25 0.208 0.246 no Bifunctional protein putA <UNP PUTA_ECOLI> [ESCHERICHIA COLI] GHMGTTTMGVKLDDATRERIKSAATRIDRTPHWLIKQAIFSYLEQLENSDTLPE
>2F4IA 197 XRAY 2.25 0.193 0.248 no hypothetical protein TM0957 <UNP Q9X052_THEMA> [THERMOTOGA MARITIMA]
MGSDKIHHHHHHEEMEKGFDPKRYARELWFKLQDMMNEGLGYDAVEVLNTLDENPELAHQ
KFAKVVGVSNYRYYIIQGVGEIVEIKDDGILVKVRENRKVPDLFLSNHIFGNGIVNATGI
AKMEDFDRIIDFNLTATELNKIVKEEVVNSFLKQLSKGAGSVGSLVRFIAVFTLLKDEEI
KYPIEAIPLYLEIQGGF
>1R7AA 504 XRAY 1.77 0.164 0.195 no sucrose phosphorylase <UNP Q84HQ2_BIFAD> [BIFIDOBACTERIUM ADOLESCENTIS]
MKNKVQLITYADRLGDGTIKSMTDILRTRFDGVYDGVHILPFFTPFDGADAGFDPIDHTK
VDERLGSWDDVAELSKTHNIMVDAIVNHMSWESKQFQDVLAKGEESEYYPMFLTMSSVFP
NGATEEDLAGIYRPRPGLPFTHYKFAGKTRLVWVSFTPQQVDIDTDSDKGWEYLMSIFDQ
MAASHVSYIRLDAVGYGAKEAGTSCFMTPKTFKLISRLREEGVKRGLEILIEVHSYYKKQ
VEIASKVDRVYDFALPPLLLHALSTGHVEPVAHWTDIRPNNAVTVLDTHDGIGVIDIGSD
QLDRSLKGLVPDEDVDNLVNTIHANTHGESQAATGAAASNLDLYQVNSTYYSALGCNDQH
YIAARAVQFFLPGVPQVYYVGALAGKNDMELLRKTNNGRDINRHYYSTAEIDENLKRPVV
KALNALAKFRNELDAFDGTFSYTTDDDTSISFTWRGETSQATLTFEPKRGLGVDNTTPVA
MLEWEDSAGDHRSDDLIANPPVVA
>1NRIA 306 XRAY 1.90 0.216 0.246 no Hypothetical protein HI0754 <UNP Y754_HAEIN> [HAEMOPHILUS INFLUENZAE]
SNAMNDIILKSLSTLITEQRNPNSVDIDRQSTLEIVRLMNEEDKLVPLAIESCLPQISLA
VEQIVQAFQQGGRLIYIGAGTSGRLGVLDASECPPTFGVSTEMVKGIIAGGECAIRHPVE
GAEDNTKAVLNDLQSIHFSKNDVLVGIAASGRTPYVIAGLQYAKSLGALTISIASNPKSE
MAEIADIAIETIVGPEILTGSSRLKSGTAQKMVLNMLTTASMILLGKCYENLMVDVQASN
EKLKARAVRIVMQATDCNKTLAEQTLLEADQNAKLAIMMILSTLSKSEAKVLLERHQGKL
RNALSK
>3IWFA 107 XRAY 1.40 0.179 0.205 no Transcription regulator RpiR family <UNP Q8CNB1_STAES> [STAPHYLOCOCCUS EPIDERMIDIS]
MPNILYKIDNQYPYFTKNEKKIAQFILNYPHKVVNMTSQEIANQLETSSTSIIRLSKKVT
PGGFNELKTRLSKFLPKEVTQYNVELVDNESTISLKNKLHSRSKAAL
>2YZQA 282 XRAY 1.63 0.225 0.243 no Putative uncharacterized protein PH1780 <UNP O59416_PYRHO> [PYROCOCCUS HORIKOSHII]
MRVKTIMTQNPVTITLPATRNYALELFKKYKVRSFPVVNKEGKLVGIISVKRILVNPDEE
QLAMLVKRDVPVVKENDTLKKAAKLMLEYDYRRVVVVDSKGKPVGILTVGDIIRRYFAKS

EKYKGVEIEPYYQRYVSIVWEGTPLKAALKALLLSNSMALPVVDSEGNLVGIVDETDLLR
DSEIVRIMKSTELAASSEEEWILESHPTLLFEKFELQLPNKPVAEIMTRDVIVATPHMTV
HEVALKMAKYSIEQLPVIRGEGDLIGLIRDFDLLKVLVKSKA
>2OIXA 186 XRAY 1.80 0.212 0.259 no Xanthomonas outer protein D <UNP Q3BYJ5_XANC5> [XANTHOMONAS EUVESICATORIA]
ATSWLLDGHLRAYTDDLARRLRGEPNAHLLHFADSQVVTMLSSADPDQQARAQRLLAGDD
IPPIVFLPINQPNAHWSLLVVDRRNKDAVAAYHYDSMAQKDPQQRYLADMAAYHLGLDYQ
QTHEMPIAIQSDGYSAGDHVLTGIEVLAHRVLDGTFDYAGGRDLTDIEPDRGLIRDRLAQ
AEQAPA
>4IUJA 464 XRAY 1.90 0.175 0.201 no Polymerase acidic protein <UNP PA_I33A0> [INFLUENZA A VIRUS] GNARIEPFLKSTPRPLRLPDGPPCSQRSKFLLMDALKLSIEDPSHEGEGIPLYDAIKCMR
TFFGWKEPNVVKPHEKGINPNYLLSWKQVLAELQDIENEEKIPRTKNMKKTSQLKWALGE
NMAPEKVDFDDCKDVGDLKQYDSDEPELRSLASWIQNEFNKACELTDSSWIELDEIGEDA
APIEHIASMRRNYFTAEVSHCRATEYIMKGVYINTALLNASCAAMDDFQLIPMISKCRTK
EGRRKTNLYGFIIKGRSHLRNDTDVVNFVSMEFSLTDPRLEPHKWEKYCVLEVGDMLLRS
AIGHVSRPMFLYVRTNGTSKIKMKWGMEMRRCLLQSLQQIESMIEAESSVKEKDMTKEFF
ENKSETWPVGESPKGVEEGSIGKVCRTLLAKSVFNSLYASPQLEGFSAESRKLLLIVQAL
RDNLEPGTFDLGGLYEAIEECLINDPWVLLNASWFNSFLTHALR
>2XMEA 232 XRAY 1.89 0.208 0.237 no CTP-INOSITOL-1-PHOSPHATE CYTIDYLYLTRANSFERASE <UNP O29976_ARCFU> [ARCHAEOGLOBUS FULGIDUS]
MINVDGEYLKIFAGRIKLMKAVILAAGLGTRLGGVPKPLVRVGGCEIILRTMKLLSPHVS
EFIIVASRYADDIDAFLKDKGFNYKIVRHDRPEKGNGYSLLVAKNHVEDRFILTMGDHVY
SQQFIEKAVRGEGVIADREPRFVDIGEATKIRVEDGRVAKIGKDLREFDCVDTGFFVLDD
SIFEHAEKLRDREEIPLSEIVKLARLPVTYVDGELWMDVDTKEDVRRANRAL
>2XTSA 390 XRAY 1.33 0.107 0.111 no SULFITE DEHYDROGENASE <UNP P72178_PARDE> [PARACOCCUS PANTOTROPHUS]
AGTPDPLITEIQPWASEFGEAVDAHPYGLPIHFESHVKRQYVEWLTESPVSSINFTPIHA
LEGTITPQGCAFERHHSGAIELSKQDYRLMINGLVEKPLVFTFEDLLRFPRTTTTAFCEC
AANGGMEWGGAQLEGCQYTQGMIHNMEYVGVPLSVLLAEAGVKPEGKWLYAEGADASSNG
RSFPMEKVMDDVMLAFFANGEALRKEHGYPARLVVPGWEGNMWVKWVRRLGIYDKAVESR
EETSKYTDLMPDGRARKWTWVMDAKSVITSPSPQVPIRHGKGPLVISGLAWSGNGRITRV
DVSLDGGKNWTTARITGQALPKALTRFHLDIDWDGSEMLLQSRAVDETGYVQPTKDALRA
IRGRNNVYHNNGIQTWWVKADGEVENVEIA
>3FS3A 359 XRAY 2.30 0.218 0.285 no Nucleosome assembly protein 1, putative <UNP Q8I608_PLAF7> [PLASMODIUM FALCIPARUM]
MAANEGNQPIPPEEEKEISSLLESIKIGKTSLKYRCFYLYDDKMTDLTEEQKETLKKLKL
YQKEYYDYESKFEYELFLLRQKYHDLYGPIYDKRREALVGNGEAKIGTPNLPEFWLRALR
NNNTVSHVIEDHDEEILVYLNDIRCDYIKKNKEKKEGFILSFYFATNPFFSNSVLTKTYH
MKCVDCDNEPVLLHTEATVIDWYDNKNILKKNVVKKQHNKNSREVKTVQQTVNRDSFFHF
FTSHKVPNSNVIKQLSKHEVAQLEMIIEGDYEVALTIKERIIPYAVDYYLGIIIESESNS
IVSDVDSSYSSSENNSNYNSYESNNSAYNDENSNVDTNEYDDNEEEEEGAKSNEDPLTS
>1EKQA 272 XRAY 1.50 0.216 0.251 no HYDROXYETHYLTHIAZOLE KINASE <UNP THIM_BACSU> [BACILLUS SUBTILIS]
MDAQSAAKCLTAVRRHSPLVHSITNNVVTNFTANGLLALGASPVMAYAKEEVADMAKIAG
ALVLNIGTLSKESVEAMIIAGKSANEHGVPVILDPVGAGATPFRTESARDIIREVRLAAI
RGNAAEIAHTVGVTDWLIKGVDAGEGGGDIIRLAQQAAQKLNTVIAITGEVDVIADTSHV
YTLHNGHKLLTKVTGAGCLLTSVVGAFCAVEENPLFAAIAAISSYGVAAQLAAQQTADKG
PGSFQIELLNKLSTVTEQDVQEWATIERVTVS
>4KM6A 208 XRAY 1.55 0.171 0.200 no Folate receptor alpha <UNP FOLR1_HUMAN> [HOMO SAPIENS]

GSSRTELLNVCMNAKHHKEKPGPEDKLHEQCRPWRKNACCSTNTSQEAHKDVSYLYRFNW
NHCGEMAPACKRHFIQDTCLYECSPNLGPWIQQVDQSWRKERVLNVPLCKEDCEQWWEDC
RTSYTCKSNWHKGWNWTSGFNKCAVGAACQPFHFYFPTPTVLCNEIWTHSYKVSNYSRGS
GRCIQMWFDPAQGNPNEEVARFYAAAMS
>2ASTB 336 XRAY 2.30 0.203 0.221 no S-phase kinase-associated protein 2 <UNP SKP2_HUMAN> [HOMO SAPIENS]
RENFPGVSWDSLPDELLLGIFSCLCLPELLKVSGVCKRWYRLASDESLWQTLDLTGKNLH
PDVTGRLLSQGVIAFRCPRSFMDQPLAEHFSPFRVQHMDLSNSVIEVSTLHGILSQCSKL
QNLSLEGLRLSDPIVNTLAKNSNLVRLNLSGCSGFSEFALQTLLSSCSRLDELNLSWCFD
FTEKHVQVAVAHVSETITQLNLSGYRKNLQKSDLSTLVRRCPNLVHLDLSDSVMLKNDCF
QEFFQLNYLQHLSLSRCYDIIPETLLELGEIPTLKTLQVFGIVPDGTLQLLKEALPHLQI
NCSHFTTIARPTIGNKKNQEIWGIKCRLTLQKPSCL
>3JU2A 284 XRAY 1.80 0.201 0.246 no uncharacterized protein SMc04130 <UNP Q92T58_RHIME> [SINORHIZOBIUM MELILOTI]
MSLQVEGLSINLATIREQCGFAEAVDICLKHGITAIAPWRDQVAAIGLGEAGRIVRANGL
KLTGLCRGGFFPAPDASGREKAIDDNRRAVDEAAELGADCLVLVAGGLPGGSKNIDAARR
MVVEGIAAVLPHARAAGVPLAIEPLHPMYAADRACVNTLGQALDICETLGPGVGVAIDVY
HVWWDPDLANQIARAGKMKAILAHHICDWLVPTKDMLTDRGMMGDGVIDLKGIRRRIEAA
GFHGAQEVEIFSADNWWKRPADEVIATCVERYRNCCEGHHHHHH
>3AXGA 355 XRAY 2.00 0.211 0.240 no Endotype 6-aminohexanoat-oligomer hydrolase <UNP Q1EPR5_9MICO> [AGROMYCES]
MNTTPVHALTDIDGGIAVDPAPRLAGPPVFGGPGNDAFDLAPVRSTGREMLRFDFPGVSI
GAAHYEEGPTGATVIHIPAGARTAVDARGGAVGLSGGYDFNHAICLAGGASYGLEAGAGV
SGALLERLEYRTGFAEAQLVSSAVIYDFSARSTAVYPDKALGRAALEFAVPGEFPQGRAG
AGMSASAGKVDWDRTEITGQGAAFRRLGDVRILAVVVPNPVGVIMDRAGTVVRGNYDAQT
GVRRHPVFDYQEAFAEQVPPVTEAGNTTISAIVTNVRMSPVELNQFAKQVHSSMHRGIQP
FHTDMDGDTLFAVTTDEIDLPTTPGSSRGRLSVNATALGAIASEVMWDAVLEAGK
>2I6TA 303 XRAY 2.10 0.183 0.230 no UBIQUITIN-CONJUGATING ENZYME E2-LIKE ISOFORM A <UNP Q6P2F0_HUMAN> [HOMO SAPIENS]
GSSKSWANHENKTVNKITVVGGGELGIACTLAISAKGIADRLVLLDLSEGTKGATMDLEI
FNLPNVEISKDLSASAHSKVVIFTVNSLGSSQSYLDVVQSNVDMFRALVPALGHYSQHSV
LLVASQPVEIMTYVTWKLSTFPANRVIGIGCNLDSQRLQYIITNVLKAQTSGKEVWVIGE
QGEDKVLTWSGQEEVVSHTSQVQLSNRAMELLRVKGQRSWSVGLSVADMVDSIVNNKKKV
HSVSALAKGYYDINSEVFLSLPCILGTNGVSEVIKTTLKEDTVTEKLQSSASSIHSLQQQ
LKL
>4LHPA 136 XRAY 2.02 0.196 0.241 no FG41 Malonate Semialdehyde Decarboxylase <UNP F2Z288_9CORY>
[CORYNEFORM BACTERIUM]
PLIRIDLTSDRSREQRRAIADAVHDALVEVLAIPARDRFQILTAHDPSDIIAEDAGLGFQ
RSPSVVIIHVFTQAGRTIETKQRVFAAITESLAPIGVAGSDVFIAITENAPHDWSFGFGS
AQYVTGELAIPATGAA
>3Q4OA 196 XRAY 1.34 0.189 0.228 no Uncharacterized protein MJ0754 <UNP Y754_METJA> [METHANOCALDOCOCCUS JANNASCHII]
MGSSHHHHHHSSGLVPRGSHMQPISEEEKEGLIEMREEEKLARDVYLTLYNKWKLQIFKN
IAESEQTHMDAVKYLLEKYNIPDPVKNDSIGVFSNPKFEELYKKLVEKGDKSEVDALKVG
ATIEDLDIADLEKWINKTDNEDIKFVYENLMKGSRNHMRAFVRMLNNYGSNYTPQYISKE
EYEEIISSSTERGMNR
>3CNVA 162 XRAY 2.00 0.167 0.217 no Putative GntR-family transcriptional regulator <UNP Q7WD95_BORBR> [BORDETELLA BRONCHISEPTICA RB50]

VRYRFLRLAPDEEGEGGRAESRILECRRLRAPAEIARALELRAGETVVTIRRQLSMNHMP
TVIDDLWLPGTHFRGLTLELLTASKAPLYGLFESEFGVSMVRADEKLRAVAASPEIAPLL
GVEPGRPLLQVDRISYTYGDRPMEVRRGLYLTDHYHYRNSLN
>4M5DA 1237 XRAY 1.97 0.210 0.238 no U3 small nucleolar RNA-associated protein 22 <UNP UTP22_YEAST>
[SACCHAROMYCES CEREVISIAE]
MATSVKRKASETSDQNIVKVQKKHSTQDSTTDNGSKENDHSSQAINERTVPEQENDESDT
SPESNEVATNTAATRHNGKVTATESYDIHIARETAELFKSNIFKLQIDELLEQVKLKQKH
VLKVEKFLHKLYDILQEIPDWEEKSLAEVDSFFKNKIVSVPFVDPKPIPQNTNYKFNYKK
PDISLIGSFALKAGIYQPNGSSIDTLLTMPKELFEKKDFLNFRCLHKRSVYLAYLTHHLL
ILLKKDKLDSFLQLEYSYFDNDPLLPILRISCSKPTGDSLSDYNFYKTRFSINLLIGFPY
KVFEPKKLLPNRNCIRIAQESKEQSLPATPLYNFSVLSSSTHENYLKYLYKTKKQTESFV
EATVLGRLWLQQRGFSSNMSHSGSLGGFGTFEFTILMAALLNGGGINSNKILLHGFSSYQ
LFKGVIKYLATMDLCHDGHLQFHSNPENSSSSPASKYIDEGFQTPTLFDKSTKVNILTKM
TVSSYQILKEYAGETLRMLNNVVQDQFSNIFLTNISRFDNLKYDLCYDVQLPLGKYNNLE
TSLAATFGSMERVKFITLENFLAHKITNVARYALGDRIKYIQIEMVGQKSDFPITKRKVY
SNTGGNHFNFDFVRVKLIVNPSECDKLVTKGPAHSETMSTEAAVFKNFWGIKSSLRRFKD
GSITHCCVWSTSSSEPIISSIVNFALQKHVSKKAQISNETIKKFHNFLPLPNLPSSAKTS
VLNLSSFFNLKKSFDDLYKIIFQMKLPLSVKSILPVGSAFRYTSLCQPVPFAYSDPDFFQ
DVILEFETSPKWPDEITSLEKAKTAFLLKIQEELSANSSTYRSFFSRDESIPYNLEIVTL
NILTPEGYGFKFRVLTERDEILYLRAIANARNELKPELEATFLKFTAKYLASVRHTRTLE
NISHSYQFYSPVVRLFKRWLDTHLLLGHITDELAELIAIKPFVDPAPYFIPGSLENGFLK
VLKFISQWNWKDDPLILDLVKPEDDIRDTFETSIGAGSELDSKTMKKLSERLTLAQYKGI
QMNFTNLRNSDPNGTHLQFFVASKNDPSGILYSSGIPLPIATRLTALAKVAVNLLQTHGL
NQQTINLLFTPGLKDYDFVVDLRTPIGLKSSCGILSATEFKNITNDQAPSNFPENLNDLS
EKMDPTYQLVKYLNLKYKNSLILSSRKYIGVNGGEKGDKNVITGLIKPLFKGAHKFRVNL
DCNVKPVDDENVILNKEAIFHEIAAFGNDMVINFETD
>3KGWA 393 XRAY 1.65 0.171 0.198 no Alanine-glyoxylate aminotransferase <UNP Q8R128_MOUSE>
[MUS MUSCULUS]
GMGSYQLLVPPPEALSKPLSVPTRLLLGPGPSNLAPRVLAAGSLRMIGHMQKEMLQIMEE
IKQGIQYVFQTRNPLTLVVSGSGHCAMETALFNLLEPGDSFLTGTNGIWGMRAAEIADRI
GARVHQMIKKPGEHYTLQEVEEGLAQHKPVLLFLVHGESSTGVVQPLDGFGELCHRYQCL
LLVDSVASLGGVPIYMDQQGIDIMYSSSQKVLNAPPGISLISFNDKAKYKVYSRKTKPVS
FYTDITYLAKLWGCEGETRVIHHTTPVTSLYCLRESLALIAEQGLENCWRRHREATAHLH
KHLQEMGLKFFVKDPEIRLPTITTVTVPAGYNWRDIVSYVLDHFSIEISGGLGPTEERVL
RIGLLGYNATTENVDRVAEALREALQHCPKNKL
>1MNNA 340 XRAY 1.40 0.195 0.206 no NDT80 protein <UNP NDT80_YEAST> [SACCHAROMYCES CEREVISIAE]
MNEMENTVPVLQDDLVSKYERELSTEQEEDTPVILTQLNEDGTTSNYFDKRKLKIAPRST
LQFKVGPPFELVRDYCPVVESHTGRTLDLRIIPRIDRGFDHIDEEWVGYKRNYFTLVSTF
ETANCDLDTFLKSSFDLLVEDSSVEGRLRVQYFAIKIKAKNDDDDTEINLVQHTAKRDKG
PQFCPSVCPLVPSPLPKHQTIREASNVRNITKMKKYDSTFYLHRDHVNYEEYGVDSLLFS
YPEDSIQKVARYERVQFASSISVKKPSQQNKHFSLHVILGAVVDPDTFHGENPGIPYDEL
ALKNGSKGMFVYLQEMKTPPLIIRGRSPSNYASSQRITVR
>3JQOA 227 XRAY 2.60 0.229 0.259 no TraF protein <UNP Q46705_ECOLX> [ESCHERICHIA COLI]
SNSPGAQPQDNETSEGSSALAKNLTPARLKASRAGVMANPSLTVPKGKMIPCGTGTELDT
TVPGQVSCRVSQDVYSADGLVRLIDKGSWVDGQITGGIKDGQARVFVLWERIRNDQDGTI
VNIDSAGTNSLGSAGIPGQVDAHMWERLRGAIMISLFSDTLTALVNQTQSNNIQYNSTEN

SGGQLASEALRSYMSIPPTLYDQQGDAVSIFVARDLDFSGVYTLADN
>2YX0A 342 XRAY 2.21 0.194 0.242 no radical sam enzyme <UNP O59412_PYRHO> [PYROCOCCUS HORIKOSHII]
MMEMITIKPGKITVQANPNMPKEVAELFRKQHYEIVGRHSGVKLCHWLKKSLTEGRFCYK
QKFYGIHSHRCLQMTPVLAWCTHNCIFCWRPMENFLGTELPQPWDDPAFIVEESIKAQRK
LLIGYKGNPKVDKKKFEEAWNPTHAAISLSGEPMLYPYMGDLVEEFHKRGFTTFIVTNGT
IPERLEEMIKEDKLPTQLYVSITAPDIETYNSVNIPMIPDGWERILRFLELMRDLPTRTV
VRLTLVKGENMHSPEKYAKLILKARPMFVEAKAYMFVGYSRNRLTINNMPSHQDIREFAE
ALVKHLPGYHIEDEYEPSRVVLIMRDDVDPQGTGVEGRFIKH
>2WXFA 940 XRAY 1.90 0.211 0.240 no PHOSPHATIDYLINOSITOL-4,5-BISPHOSPHATE 3-KINASE CATALYTIC SUBUNIT DELTA ISOFORM <UNP Q3UDT3_MOUSE> [MUS MUSCULUS]
GGDRVKKLINSQISLLIGKGLHEFDSLRDPEVNDFRTKMRQFCEEAAAHRQQLGWVEWLQ
YSFPLQLEPSARGWRAGLLRVSNRALLVNVKFEGSEESFTFQVSTKDMPLALMACALRKK
ATVFRQPLVEQPEEYALQVNGRHEYLYGNYPLCHFQYICSCLHSGLTPHLTMVHSSSILA
MRDEQSNPAPQVQKPRAKPPPIPAKKPSSVSLWSLEQPFSIELIEGRKVNADERMKLVVQ
AGLFHGNEMLCKTVSSSEVNVCSEPVWKQRLEFDISVCDLPRMARLCFALYAVVEKAKKA
RSTKKKSKKADCPIAWANLMLFDYKDQLKTGERCLYMWPSVPDEKGELLNPAGTVRGNPN
TESAAALVIYLPEVAPHPVYFPALEKILELGRHGERGRITEEEQLQLREILERRGSGELY
EHEKDLVWKMRHEVQEHFPEALARLLLVTKWNKHEDVAQMLYLLCSWPELPVLSALELLD
FSFPDCYVGSFAIKSLRKLTDDELFQYLLQLVQVLKYESYLDCELTKFLLGRALANRKIG
HFLFWHLRSEMHVPSVALRFGLIMEAYCRGSTHHMKVLMKQGEALSKLKALNDFVKVSSQ
KTTKPQTKEMMHMCMRQETYMEALSHLQSPLDPSTLLEEVCVEQCTFMDSKMKPLWIMYS
SEEAGSAGNVGIIFKNGDDLRQDMLTLQMIQLMDVLWKQEGLDLRMTPYGCLPTGDRTGL
IEVVLHSDTIANIQLNKSNMAATAAFNKDALLNWLKSKNPGEALDRAIEEFTLSCAGYCV
ATYVLGIGDRHSDNIMIRESGQLFHIDFGHFLGNFKTKFGINRERVPFILTYDFVHVIQQ
GKTNNSEKFERFRGYCERAYTILRRHGLLFLHLFALMRAAGLPELSCSKDIQYLKDSLAL
GKTEEEALKHFRVKFNEALRESWKTKVNWLAHNVSKDNRQ
>2A2MA 258 XRAY 1.88 0.143 0.165 no hypothetical protein BT3146 <UNP Q8A309_BACTN> [BACTEROIDES THETAIOTAOMICRON]
MGSDKIHHHHHHMNDFKNQWLRKRTFAIPASRLTGRLTTLKSDVPAADSLFWKLWNGSLD
TAVQVLQTDYFKGIAAGTLDPNAYGSLMVQDGYYCFRGRDDYATAATCAQDETLREFFKA
KAKSYDEYNETYHQTWHLREASGLIPGTDIKDYADYEAYVAGSLASPYMCVVMLPCEYLW
PWIANFLDGYTPTNSLYRFWIEWNGGTPNGAYQMGNMLEQYRDKIDEDKAVEIFNTAMNY
ELKVFTSSTILTTIENGK
>3IAXB 115 XRAY 2.60 0.193 0.257 no Colicin-A <UNP CEA_CITFR> [CITROBACTER FREUNDII]
MPGFNYGGKGDGTGWSSERGSGPEPGGGSHGNSGGHDRGDSSNVGNESVTVMKPGDSYNT
PWGKVIINAAGQPTMNGTVMTADNSSMVPYGRGFTRVLNSLVNNPVSLEHHHHHH
>3N7CA 130 XRAY 2.26 0.236 0.293 no ABR034Wp <UNP Q75DJ0_ASHGO> [ASHBYA GOSSYPII]
MSLTNGEENEEVLFCEKAKLLIFDSDTKGYTSRGVGELKLLRKKDDKGKVRVLCRSEGMG
HVLLNTSVVKSFKYQPIDADNENLIKWPIITDGKLETFIIKVKQKADGRRLVGAVADAQQ
AMEGHHHHHH
>1D2TA 231 XRAY 1.90 0.219 0.257 no ACID PHOSPHATASE <UNP Q9S1A6_ESCBL> [ESCHERICHIA BLATTAE]
LALVATGNDTTTKPDLYYLKNSEAINSLALLPPPPAVGSIAFLNDQAMYEQGRLLRNTER
GKLAAEDANLSSGGVANAFSGAFGSPITEKDAPALHKLLTNMIEDAGDLATRSAKDHYMR
IRPFAFYGVSTCNTTEQDKLSKNGSYPSGHTSIGWATALVLAEINPQRQNEILKRGYELG
QSRVICGYHWQSDVDAARVVGSAVVATLHTNPAFQQQLQKAKAEFAQHQKK
>3VHXB 120 XRAY 2.81 0.262 0.232 no Kinesin-like protein KIF23 <UNP KIF23_HUMAN> [HOMO

SAPIENS]
GSLLFQPDQNAPPIRLRHRRSRSAGDRWVDHKPASNMQTETVMQPHVPHAITVSVANEKA
LAKCEKYMLTHQELASDGEIETKLIKGDIYKTRGGGQSVQFTDIETLKQESPNGSRKRRS
>1FITA 147 XRAY 1.85 0.202 0.225 no FRAGILE HISTIDINE PROTEIN <UNP FHIT_HUMAN> [HOMO SAPIENS]
MSFRFGQHLIKPSVVFLKTELSFALVNRKPVVPGHVLVCPLRPVERFHDLRPDEVADLFQ
TTQRVGTVVEKHFHGTSLTFSMQDGPEAGQTVKHVHVHVLPRKAGDFHRNDSIYEELQKH
DKEDFPASWRSEEEMAAEAAALRVYFQ
>3DXLA 303 XRAY 1.30 0.189 0.214 no Allergen Aed a 2 <UNP D7_AEDAE> [AEDES AEGYPTI]
MGPFDPEEMLFIFTRCMEDNLEDGANRLPMLAKWKEWINEPVDSPATQCFGKCVLVRTGL
YDPVAQKFDASVIQEQFKAYPSLGEKSKVEAYANAVKQLPSTNNDCAAVFKAYDPVHKAH
KDTSKNLFHGNKELTKGLYEKLGKDIRQKKQSYFEFCENKYYPAGSDKRQQLCQIRQYTV
LDDALFKEHTDCVMKGIRYITKDNQLDVEEVKRDFKLVNKDTKALEEVLNDCKSKEPSNA
KEKSWHYYKCLVESSVKDDFKEAFDYREVRSQIYAFNLPKNQAYSKPAVQSQVMEIDGKQ
CPQ
>1F1MA 164 XRAY 1.80 0.201 0.235 no OUTER SURFACE PROTEIN C <UNP Q9AGB1_BORBU> [BORRELIA BURGDORFERI]
KGPNLTEISKKITESNAVVLAVKEVETLLTSIDELAKAIGKKIKSDVSLDNEADHNGSLM
SGAYLISTLITKKISAIKDSGELKAEIEKAKKCSEEFTAKLKGEHTDLGKEGVTDDNAKK
AILKTNNDKTKGADELEKLFESVKNLSKAAKEMLTNSVKELTSP
>2P6VA 114 XRAY 2.00 0.214 0.248 no Transcription initiation factor TFIID subunit 4 <UNP TAF4_HUMAN> [HOMO SAPIENS]
TVPGATTTSSAATETMENVKKCKNFLSTLIKLASSGKQSTETAANVKELVQNLLDGKIEA
EDFTSRLYRELNSSPQPYLVPFLKRSLPALRQLTPDSAAFIQQSQQQPPPPTSQ
>2F6MA 65 XRAY 2.10 0.242 0.261 no Suppressor protein STP22 of temperature-sensitive alpha-factor receptor and arginine permease <UNP STP22_YEAST> [SACCHAROMYCES CEREVISIAE]
MTDGLNQLYNLVAQDYALTDTIEALSRMLHRGTIPLDTFVKQGRELARQQFLVRWHIQRI
TSPLS
>4EI7A 389 XRAY 1.90 0.185 0.237 no Plasmid replication protein RepX <UNP REPX_BACC1> [BACILLUS CEREUS]
MAGNFSEIESQGNISLKFGFLGLGMGGCAIAAECANKETQIKNNKYPYRAILVNTNSQDF
NKIEIKNTGNVRKIQLEGYEQGAARNPQVGEEAFVKHETKIFEAVKQEFEDRDFIWITCG
LGGGTGTGALLKAIEMLYEHDYNFGLLLTLPRDAEALKVLENATSRIRSIAMNQEAFGSI
VLIDNAKLYRKFEEENPSALANEYTSYSNKYIADALHEINLVTSSFTPFSDTHFDASEFA
QVINTPGVLSLAKLELKSNQLDTENPLGYLTQLGNALEKGVLYDTEREELESAKKSALSI
VTSPLRAGRLYNFSFLNQMENFLKERTPYVDERPIAPYVNKHTTKKEEDIVKFYSVVAGL
PLPKRVSDIIDEITRIKEEREQANSKKSN
>3T30A 562 XRAY 2.50 0.230 0.285 no Metal dependent hydrolase <UNP Q72JJ7_THET2> [THERMUS THERMOPHILUS HB27]
MSHHHHHHSSQGGPQDHVEIIPLGGMGEIGKNITVFRFRDEIFVLDGGLAFPEEGMPGVD
LLIPRVDYLIEHRHKIKAWVLTHGAEDHIGGLPFLLPMIFGKESPVPIYGARLTLGLLRG
KLEEFGLRPGAFNLKEISPDDRIQVGRYFTLDLFRMTHSIPDNSGVVIRTPIGTIVHTGD
FKLDPTPIDGKVSHLAKVAQAGAEGVLLLIADATNAERPGYTPSEMEIAKELDRVIGRAP
GRVFVTTFASHIHRIQSVIWAAEKYGRKVAMEGRSMLKFSRIALELGYLKVKDRLYTLEE
VKDLPDHQVLILATGSQGQPMSVLHRLAFEGHAKMAIKPGDTVILSSSPIPGNEEAVNRV
INRLYALGAYVLYPPTYKVHASGHASQEELKLILNLTTPRFFLPWHGEVRHQMNFKWLAE
SMSRPPEKTLIGENGAVYRLTRETFEKVGEVPHGVLYVDGLGVGDITEEILADRRHMAEE
GLVVITALAGEDPVVEVVSRGFVKAGERLLGEVRRMALEALKNGVREKKPLERIRDDIYY

PVKKFLKKATGRDPMILPVVIE
>2HBJA 410 XRAY 2.10 0.239 0.292 no Exosome complex exonuclease RRP6 <UNP RRP6_YEAST>
[SACCHAROMYCES CEREVISIAE] GMVEKPQLKFKSPIDNSESHPFIPLLKEKPNALKPLSESLRLVDDDENNPSHYPHPYEYE
IDHQEYSPEILQIREEIPSKSWDDSVPIWVDTSTELESMLEDLKNTKEIAVDLEHHDYRS
YYGIVCLMQISTRERDYLVDTLKLRENLHILNEVFTNPSIVKVFHGAFMDIIWLQRDLGL
YVVGLFDTYHASKAIGLPRHSLAYLLENFANFKTSKKYQLADWRIRPLSKPMTAAARADT
HFLLNIYDQLRNKLIESNKLAGVLYESRNVAKRRFEYSKYRPLTPSSEVYSPIEKESPWK
ILMYQYNIPPEREVLVRELYQWRDLIARRDDESPRFVMPNQLLAALVAYTPTDVIGVVSL
TNGVTEHVRQNAKLLANLIRDALRNIKNTNEEATPIPSSETKADGILLET
>1PBJA 125 XRAY 1.40 0.192 0.202 no hypothetical protein <UNP O27659_METTH>
[METHANOTHERMOBACTER THERMAUTOTROPHICUS STR. DELTA H]
MRVEDVMVTDVDTIDITASLEDVLRNYVENAKGSSVVVKEGVRVGIVTTWDVLEAIAEGD
DLAEVKVWEVMERDLVTISPRATIKEAAEKMVKNVVWRLLVEEDDEIIGVISATDILRAK
MAKRY
>2ERVA 150 XRAY 2.00 0.200 0.233 no hypothetical protein Paer03002360 <UNP Q9HVD1_PSEAE>
[PSEUDOMONAS AERUGINOSA]
ADVSAAVGATGQSGMTYRLGLSWDWDKSWWQTSTGRLTGYWDAGYTYWEGGDEGAGKHSL
SFAPVFVYEFAGDSIKPFIEAGIGVAAFSGTRVGDQNLGSSLNFEDRIGAGLKFANGQSV
GVRAIHYSNAGLKQPNDGIESYSLFYKIPI
>1GZSB 165 XRAY 2.30 0.226 0.255 no SOPE <PDB 1GZS> [SALMONELLA TYPHIMURIUM]
GSLTNKVVKDFMLQTLNDIDIRGSASKDPAYASQTREAILSAVYSKNKDQCCNLLISKGI
NIAPFLQEIGEAAKNAGLPGTTKNDVFTPSGAGANPFITPLISSANSKYPRMFINQHQQA
SFKIYAEKIIMTEVAPLFNECAMPTPQQFQLILENIANKYIQNTP
>1LMIA 131 XRAY 1.50 0.198 0.252 no Immunogenic protein MPT63/MPB63 <UNP MP63_MYCTU>
[MYCOBACTERIUM TUBERCULOSIS]
SAYPITGKLGSELTMTDTVGQVVLGWKVSDLKSSTAVIPGYPVAGQVWEATATVNAIRGS
VTPAVSQFNARTADGINYRVLWQAAGPDTISGATIPQGEQSTGKIYFDVTGPSPTIVAMN
NGMEDLLIWEP
>1C8UA 285 XRAY 1.90 0.230 0.248 no ACYL-COA THIOESTERASE II <UNP TESB_ECOLI> [ESCHERICHIA
COLI] SQALKNLLTLLNLEKIEEGLFRGQSEDLGLRQVFGGQVVGQALYAAKETVPEERLVHSFH
SYFLRPGDSKKPIIYDVETLRDGNSFSARRVAAIQNGKPIFYMTASFQAPEAGFEHQKTM
PSAPAPDGLPSETQIAQSLAHLLPPVLKDKFICDRPLEVRPVEFHNPLKGHVAEPHRQVW
IRANGSVPDDLRVHQYLLGYASDLNFLPVALQPHGIGFLEPGIQIATIDHSMWFHRPFNL
NEWLLYSVESTSASSARGFVRGEFYTQDGVLVASTVQEGVMRNHN
>3VS8A 410 XRAY 1.76 0.185 0.230 no Type III polyketide synthase <PDB 3VS8> [AZOTOBACTER
VINELANDII] HMNDMAHPNSAVLADFIPVQLAKPVPQRITLELTAYGFARAHCLSNGITDEEGFVQVYKT
VKEKFDKYAVSPAQIKQRQLVYFPKLTDIRFGDGNFDIADPEPDQAHLRLFDIKKDPRGA
DLKTRHESYAKVVGKGLEQMFEGTLEAPDDLIHVTCSGYLAPSPAERMVADRGWFETTVT
HSYNMGCYGAFPAIKMAHGMLASAQWGATPPKTRVDIAHTELMSAHNNIAESRVDNIISA
TLFSDGLIKYSVYPEDELRRQGLRGLRILAMSEHLLPDSADTMTGVPGSHQFVMTLSPLV
PAIIKRHVRAFAVDLLRRAGMDFERDKDALSFAIHPGGPKIVDHVQEELGLAEDQVAISK
SVFLENGNMSSSTIPHILKAYLEEATVGTRIACLGFGPGLTAAGLVLEKI
>1Z0WA 207 XRAY 1.20 0.137 0.181 no Putative protease La homolog type <UNP
LONH_ARCFU> [ARCHAEOGLOBUS FULGIDUS]
DYKLFITEGYEVGRVNGLAVIGESAGIVLPIIAEVTPSMSKSEGRVIATGRLQEIAREAV
MNVSAIIKKYTGRDISNMDVHIQFVGTYEGVEGDSASISIATAVISAIEGIPVDQSVAMT
GSLSVKGEVLPVGGVTQKIEAAIQAGLKKVIIPKDNIDDVLLDAEHEGKIEVIPVSRINE
VLEHVLEDGKKKNRLMSKFKELELAAV

>3V0DA 339 XRAY 1.10 0.141 0.161 no Voltage-sensor containing phosphatase <UNP Q4W8A1_CIOIN> [CIONA INTESTINALIS]
GHMKASSRRTISQNKRRYRKDGFDLDLTYVTDHVIAMSFPSSGRQSLFRNPIGEVSRFFK
TKHPDKFRIYNLCSERGYDETKFDNHVYRVMIDDHNVPTLVDLLKFIDDAKVWMTSDPDH
VIAIHSKGGKGRTGTLVSSWLLEDGKFDTAKEALEYFGSRRTDFEVGDVFQGVETASQIR
YVGYFEKIKKNYGGQLPPMKKLKVTGVTITAIQGVGRGNGSDLSMQIVSERQEVLLCKFA
EGYNCALQYDATDDCVTCEVKNCPVLAGDIKVRFMSTSKSLPRGYDNCPFYFWFNTSLVE
GDHVTLKREEIDNPHKKKTWKIYRDNFTVKLTFSDAEDI
>2E3HA 90 XRAY 1.45 0.193 0.215 no Restin <UNP REST_HUMAN> [HOMO SAPIENS]
ELKIGDRVLVGGTKAGVVRFLGETDFAKGEWCGVELDEPLGKNDGAVAGTRYFQCQPKYG
LFAPVHKVTKIGFPSTTPAKAKANAVRRVM
>2EWCA 126 XRAY 2.15 0.164 0.204 no conserved hypothetical protein <UNP Q99XS4_STRP1> [STREPTOCOCCUS PYOGENES]
MKTIRRYDVNEDRGHTGLVEAGDFYYLNYCVGNVGQDIESQINGAFDEMERRLALVGLTL
DAVVQMDCLFRDVWNIPVMEKMIKERFNGRYPARKSIQTEFAHHGGPQGLLFQVDGVAYS
KHISMT
>3OCJA 305 XRAY 1.39 0.189 0.211 no Putative exported protein <UNP Q7WBE0_BORPA> [BORDETELLA PARAPERTUSSIS]
SNAMSPSPLISHRAASADLAGMVRQARQRILLQGNVPGFDVARQIELLHGLAESELGRFL
LLYRGLNAEWTHRLVTHQPGSGALAPLERVFYERLPAVLATRERHGHFRRALQRHLRPGC
VVASVPCGWMSELLALDYSACPGVQLVGIDYDPEALDGATRLAAGHALAGQITLHRQDAW
KLDTREGYDLLTSNGLNIYEPDDARVTELYRRFWQALKPGGALVTSFLTPPPALSPDSPW
DMQAIDPHDLQLQQLVFTRLIQPRWNALRTHAQTRAQLEEAGFTDLRFEDDRARLFPTVI
ARKPA
>3FXDB 73 XRAY 2.10 0.210 0.250 no Protein IcmR <UNP Q5ZYC9_LEGPH> [LEGIONELLA PNEUMOPHILA SUBSP. PNEUMOPHILA STR. PHILADELPHIA 1]
EIGEPDVTDATLGSVYSEIISPVKDCILTVAKAVSFNPGGKDNTDAVEVLTELNTKVERA
ALNQPILTTKTER
>3DXRA 89 XRAY 2.50 0.247 0.275 no Mitochondrial import inner membrane translocase subunit TIM9
<UNP TIM9_YEAST> [SACCHAROMYCES CEREVISIAE]
GSMDALNSKEQQEFQKVVEQKQMKDFMRLYSNLVERCFTDCVNDFTTSKLTNKEQTCIMK
CSEKFLKHSERVGQRFQEQNAALGQGLGR
>2HHCA 330 XRAY 1.54 0.183 0.197 no Nodulation fucosyltransferase NodZ <UNP Q9AQ17_BRASW> [BRADYRHIZOBIUM SP.]
MTKERFVISRRRTGFGDCLWSLASAWSYAQRTGRTLVIDWRGSCYVEQPFSNAFPAFFEP
VEDIAGVPVICDDRVNQLSFPGPFFPRWWNRPSIDCINRPDEQIFRERDELTELFQARED
SEANTIVCDACLMWRCSEEAERLIFRNIKLRSEIRARIDALYEEHFSGHSIIGVHVRHGN
GEDIMEHAPYWADSELALHQVCMAIRKAKALSYPKPVKVFLCTDSAQVLDQVSGLFPDVF
AVPKRFQADRAGPLHSAEMGIEGGASALIDMYLLARCATVIRFPPTSAFTRYARLLVPRI
IEFDLSNPGHLTMIDNPYEHFAASHHHHHH
>3V46A 170 XRAY 1.55 0.174 0.210 no Cell division control protein 73 <UNP CDC73_YEAST> [SACCHAROMYCES CEREVISIAE]
GIDPFTSSGGPRKDPIILIPSAASSILTVANIKQFLLESKYVNPRNLPSVPNGLVNIEKN
FERISRPIRFIIVDNTRMFTKPEYWDRVVAIFTTGHTWQFNNYQWNSPQELFQRCKGYYF
HFAGDSVPQHVQQWNVEKVELDKNKRFKDVEVVRYFWHSLEKELISRGYR
>3K69A 162 XRAY 1.95 0.174 0.218 no Putative transcription regulator <UNP Q88ZG1_LACPL> [LACTOBACILLUS PLANTARUM]
GMGGSNMKLDFSVAVHSILYLDAHRDSKVASRELAQSLHLNPVMIRNILSVLHKHGYLTG

TVGKNGGYQLDLALADMNLGDLYDLTIPPTISYARFITGPSKTDEQADQSPIAANISETL
TDLFTVADRQYRAYYHQFTMADLQADLNHHGTFLQHEQDSES
>1SG4A 260 XRAY 1.30 0.157 0.202 no 3,2-trans-enoyl-CoA isomerase, mitochondrial <UNP D3D2_HUMAN>
[HOMO SAPIENS]
GSQRVLVEPDAGAGVAVMKFKNPPVNSLSLEFLTELVISLEKLENDKSFRGVILTSDRPG
VFSAGLDLTEMCGRSPAHYAGYWKAVQELWLRLYQSNLVLVSAINGACPAGGCLVALTCD
YRILADNPRYCIGLNETQLGIIAPFWLKDTLENTIGHRAAERALQLGLLFPPAEALQVGI
VDQVVPEEQVQSTALSAIAQWMAIPDHARQLTKAMMRKATASRLVTQRDADVQNFVSFIS
KDSIQKSLQMYLERLKEEKG
>4IKNA 261 XRAY 1.85 0.194 0.237 no AP-3 complex subunit mu-1 <UNP AP3M1_RAT>
[RATTUS NORVEGICUS]
GAMDPEFIPWRRAGVKYTNNEAYFDVVEEIDAIIDKSGSTVFAEIQGVIDACIKLSGMPD
LSLSFMNPRLLDDVSFHPCIRFKRWESERVLSFIPPDGNFRLISYRVSSQNLVAIPVYVK
HNISFKENSSCGRFDITIGPKQNMGKTIEGITVTVHMPKVVLNMNLTPTQGSYTFDPVTK
VLAWDVGKITPQKLPSLKGLVNLQSGAPKPEENPNLNIQFKIQQLAISGLKVNRLDMYGE
KYKPFKGVKYITKAGKFQVRT
>2OH1A 179 XRAY 1.46 0.170 0.193 no Acetyltransferase, GNAT family <UNP Q722P7_LISMF>
[LISTERIA MONOCYTOGENES STR. 4B]
GMNQNKITAGGLEFLVRFAAPTDRLKINDLMIDTARWLKESGSTQWSDILHGFDVHNIEQ
RIELGEVALFETEAGALAGAMIIRKTPSDWDTDLWEDLAIDKAYYLHRIMVSRAFSGISL
SKQMIYFAEKLGIEMSVPFIRLDCIESNETLNQMYVRYGFQFSGKKNGFYLYQKELSQK
>4F43A 320 XRAY 2.35 0.239 0.290 no Protelomerase <UNP Q7CWV1_AGRT5> [AGROBACTERIUM TUMEFACIENS]
DYPKTGVATSIVEKIERAEFNTAGRKPTVLLRIADFIAAMNGMDAKQDMQALWDAEIAIM
NGRAQTTIISYITKYRNAIREAFGDDHPMLKIATGDAAMYDEARRVKMEKIANKHGALIT
FENYRQVLKICEDCLKSSDPLMIGIGLIGMTGRAPYEVFTQAEFSPAPYGKGVSKWSILF
NGQAKTKQGEGTKFGITYEIPVLTRSETVLAAYKRLRESGQGKLWHGMSIDDFSSETRLL
LRDTVFNLFEDVWPKEELPKPYGLRHLYAEVAYHNFAPPHVTKNSYFAAILGHNNNDLET
SLSYMTYTLPEDRDNALARL
>1UYNX 308 XRAY 2.60 0.231 0.285 no NALP <UNP Q8GKS5> [NEISSERIA MENINGITIDIS]
DGVRIFNSLAATVYADSTAAHADMQGRRLKAVSDGLDHNGTGLRVIAQTQQDGGTWEQGG
VEGKMRGSTQTVGIAAKTGENTTAAATLGMGRSTWSENSANAKTDSISLFAGIRHDAGDI
GYLKGLFSYGRYKNSISRSTGADEHAEGSVNGTLMQLGALGGVNVPFAATGDLTVEGGLR
YDLLKQDAFAEKGSALGWSGNSLTEGTLVGLAGLKLSQPLSDKAVLFATAGVERDLNGRD
YTVTGGFTGATAATGKTGARNMPHTRLVAGLGADVEFGNGWNGLARYSYAGSKQYGNHSG
RVGVGYRF
>3MKCA 394 XRAY 1.77 0.237 0.241 no racemase <UNP B6R2Z6_9RHOB> [PSEUDOVIBRIO SP. JE062]
MSLALNPAVAPIKSIEFIPVNYQASNWSQNTVVVKVTDENGVYGLGEADGSPDAILAYAN
IETEHKWLTNITEKAIGRLPIEINAIWDAMYDATQWQGMRGLGMFALSGIDMALYDLAGK
QLGVPAYQLLGGTNKDKVHPYLTLYPAIPVDASLDVAIKGYAPLLEKAKAHNIRAVKVCV
PIKADWSTKEVAYYLRELRGILGHDTDMMVDYLYRFTDWYEVARLLNSIEDLELYFAEAT
LQHDDLSGHAKLVENTRSRICGAEMSTTRFEAEEWITKGKVHLLQSDYNRCGGLTELRRI
TEMATANNVQVMPHNWKTGITSAAAIHYQFAVGNAPYFEYVHPEFCDGELRKYLVTPEAE
LVDGGFAKPTAPGLGIDLNQEFLASLEGHHHHHH
>1GNLA 544 XRAY 1.25 0.130 0.145 no HYBRID CLUSTER PROTEIN <UNP PRIS_DESDE> [DESULFOVIBRIO DESULFURICANS]
SNAMFCYQCQETVGNKGCTQVGVCGKKPETAALQDALIYVTKGLGQIATRLRAEGKAVDH
RIDRLVTGNLFATITNANFDDDILAERVRMTCAAKKELAASLTDKSGLSDAALWEASEKS

AMLAKAGTVGVMATTDDDVRSLRWLITFGLKGMAAYAKHADVLGKHENSLDAFMQEALAK
TLDDSLSVADLVALTLETGKFGVSAMALLDAANTGTYGHPEITKVNIGVGSNPGILISGH
DLRDLEMLLKQTEGTGVDVYTHSEMLPAHYYPAFKKYAHFKGNYGNAWWKQKEEFESFNG
PVLLTTNCLVPPKDSYKDRVYTTGIVGFTGCKHIPGEIGEHKDFSAIIAHAKTCPAPTEI
ESGEIIGGFAHNQVLALADKVIDAVKSGAIKKFVVMAGCDGRAKSRSYYTDFAEGLPKDT
VILTAGCAKYRYNKLNLGDIGGIPRVLDAGQCNDSYSLAVIALKLKEVFGLEDVNDLPIV
YNIAWYEQKAVIVLLALLSLGVKNIHLGPTLPAFLSPNVAKVLVEQFNIGGITSPQDDLK
AFFG
>3QTMA 346 XRAY 2.15 0.180 0.213 no Uncharacterized protein C4B3.07 <UNP
YJ27_SCHPO> [SCHIZOSACCHAROMYCES POMBE]
MEHHHHHHTMLVYTEEDNISQLWGLYEMSREKLENDDIDASVSLVFGTIHEADRILRNTE
DISTLPKDFHAAYSSALLAVSELFEIAQKRLKETNTEESYIDAAIERAQLGLDAPGNESR
LFLALARAYLEKVRVLVWRHDNEESLANIPVTQLVNPYIEKAIQYLRPLAQDSTEYFDAL
TPDSLRPLYILSSYLFQFGDQFSEAFLLDVCSIITALWLKSVVDPNTPAYYKLIAQEAVL
NNYTTFAEYYMDLLDNSESNVDDLINKASSWLNNSVDTWNVIYTLDKSPERLLKLADIKM
DLAQIVQDEASQDNYLKEACNAIKEAQGSGVELSPDYVEFVEAYSA
>2IT2A 200 XRAY 1.50 0.250 0.265 no UPF0130 protein PH1069 <UNP Y1069_PYRHO> [PYROCOCCUS
HORIKOSHII]
MLLYMRFTENFERAKKEALMSLEIALRKGEVDEDIIPLLKKINSIENYFTTSSCSGRISV
MEMPHFGDKVNAKWLGKWHREVSLYEVLEAIKKHRSGQLWFLVRSPILHVGAKTLEDAVK
LVNLAVSCGFKYSNIKSISNKKLIVEIRSTERMDVLLGENGEIFVGEEYLNKIVEIANDQ
MRRFKEKLKRLESKINALNR
>3F3BA 126 XRAY 2.50 0.230 0.263 no Phage-like element PBSX protein xkdH <UNP XKDH_BACSU>
[BACILLUS SUBTILIS]
MSYRQMLIHRCDIYHEAAQAPSAGRFGIPADRLQPVISYPDTPDEQDVPCYFTEKTQQLI
QEEPDQTVYHSFLVHFPLSADIRVNDKIIWENHKYILKLPKRIRHHHWEVVAVRDESLLE
HHHHHH
>3DEFA 262 XRAY 1.96 0.173 0.213 no T7I23.11 protein <UNP O23680_ARATH> [ARABIDOPSIS THALIANA]
MGSLVREWVGFQQFPAATQEKLIEFFGKLKQKDMNSMTVLVLGKGGVGKSSTVNSLIGEQ
VVRVSPFQAEGLRPVMVSRTMGGFTINIIDTPGLVEAGYVNHQALELIKGFLVNRTIDVL
LYVDRLDVYAVDELDKQVVIAITQTFGKEIWCKTLLVLTHAQFSPPDELSYETFSSKRSD
SLLKTIRAGSKMRKQEFEDSAIAVVYAENSGRCSKNDKDEKALPNGEAWIPNLVKAITDV
ATNQRKAIHVDAAALEHHHHHH
>3GM8A 801 XRAY 2.40 0.229 0.287 no Glycoside hydrolase family 2, candidate beta-glycosidase
<UNP
A6KXE5_BACV8> [BACTEROIDES VULGATUS]
SLAGETINFCKGWKFHLGDAGKGASSSSYNDSQWRILNIPHDWSIEGTYKQFENGTDWQS
GFLPAGISWYRKTFTIPSKWKNKKVQILFEGVYLNSEVWINGHWLGKRPNGYISFVYDLT
PYLQEGKNQIAVKVDHSKALTGRWYTGSGIYRPVYLLVSNPTHIPYSGIHFRSKLQNKQS
ATYTLSIEIETQEKKPIKVKTYLQAPNGSIADTSEKIFVSSADSLCFLSGSIRKPLLWSP
DSPNVYTLICQLTRDNKILDECRLPVGFRQLEFNPVSGFLLNGKSLKIKGVCDHHTVGAV
GAAVPDDLLHYRLKLLKDMGCNAIRTSHNPFSPAFYNLCDTMGIMVLNEGLDGWNQPKAA
DDYGNYFDEWWQKDMTDFIKRDRNHPSIIMWSIGNEVTGATPEIQHNLVSLFHQLDPDRP
VTQGGTDPTRGMKTDYQKKFNYLDIIGFNGNGEEIGELEHFHKNYPTLCAIATEVPHTYQ
TRGVYRSQTQWRRRDFPAPWEKGNINWEQFKHRVFPIPDLTEKECFPEESDYPYYQSSYD
NASVRISARKSWQRTCSFPWLMGEFRWGSFDYLGEAEWPQRCGNFGIIDIAAIPKDAYFL
YQSLWTDKPMVHLLPHWTHPGKEGKTIPVVIYTNCDAVELFINNVSLGSKPYTGEQLIWL
VPYSPGKIEARGIKKGKIVATDCYQSAEAPHSVALASNKYSVKAGSDEVIRIEIDITDKN

GIPCPYASNELSFHVSGPLRLLGVDNGNPTDMFPYQQPHCRCFRGKCVVLLQSDEEKGKG
TLTVQGTKLVEKKLIIEVIEG
>1OFDA 1520 XRAY 2.00 0.194 0.231 no FERREDOXIN-DEPENDENT GLUTAMATE SYNTHASE 2 <UNP GLTS_SYNY3> [SYNECHOCYSTIS SP.]
CGVGFIANLRGKPDHTLVEQALKALGCMEHRGGCSADNDSGDGAGVMTAIPRELLAQWFN
TRNLPMPDGDRLGVGMVFLPQEPSAREVARAYVEEVVRLEKLTVLGWREVPVNSDVLGIQ
AKNNQPHIEQILVTCPEGCAGDELDRRLYIARSIIGKKLAEDFYVCSFSCRTIVYKGMVR
SIILGEFYLDLKNPGYTSNFAVYHRRFSTNTMPKWPLAQPMRLLGHNGEINTLLGNINWM
AAREKELEVSGWTKAELEALTPIVNQANSDSYNLDSALELLVRTGRSPLEAAMILVPEAY
KNQPALKDYPEISDFHDYYSGLQEPWDGPALLVFSDGKIVGAGLDRNGLRPARYCITKDD
YIVLGSEAGVVDLPEVDIVEKGRLAPGQMIAVDLAEQKILKNYQIKQQAAQKYPYGEWIK
IQRQTVASDSFAEKTLFNDAQTVLQQQAAFGYTAEDVEMVVVPMASQGKEPTFCMGDDTP
LAVLSHKPRLLYDYFKQRFAQVTNPPIDPLRENLVMSLAMFLGKRGNLLEPKAESARTIK
LRSPLVNEVELQAIKTGQLQVAEVSTLYDLDGVNSLEDALTNLVKTAIATVQAGAEILVL
TDRPNGAILTENQSFIPPLLAVGAVHHHLIRAGLRLKASLIVDTAQCWSTHHFACLVGYG
ASAICPYLALESVRQWWLDEKTQKLMENGRLDRIDLPTALKNYRQSVEAGLFKILSKMGI
SLLASYHGAQIFEAIGLGAELVEYAFAGTTSRVGGLTIADVAGEVMVFHGMAFPEMAKKL
ENFGFVNYRPGGEYHMNSPEMSKSLHKAVAAYKVGGNGNNGEAYDHYELYRQYLKDRPVT
ALRDLLDFNADQPAISLEEVESVESIVKRFCTGGMSLGALSREAHETLAIAMNRLGAKSN
SGEGGEDVVRYLTLDDVDSEGNSPTLPHLHGLQNGDTANSAIKQIASGRFGVTPEYLMSG
KQLEIKMAQGAKPGEGGQLPGKKVSEYIAMLRRSKPGVTLISPPPHHDIYSIEDLAQLIY
DLHQINPEAQVSVKLVAEIGIGTIAAGVAKANADIIQISGHDGGTGASPLSSIKHAGSPW
ELGVTEVHRVLMENQLRDRVLLRADGGLKTGWDVVMAALMGAEEYGFGSIAMIAEGCIMA
RVCHTNNCPVGVATQQERLRQRFKGVPGQVVNFFYFIAEEVRSLLAHLGYRSLDDIIGRT
DLLKVRSDVQLSKTQNLTLDCLLNLPDTKQNRQWLNHEPVHSNGPVLDDDILADPDIQEA
INHQTTATKTYRLVNTDRTVGTRLSGAIAKKYGNNGFEGNITLNFQGAAGQSFGAFNLDG
MTLHLQGEANDYVGKGMNGGEIVIVPHPQASFAPEDNVIIGNTCLYGATGGNLYANGRAG
ERFAVRNSVGKAVIEGAGDHCCEYMTGGVIVVLGPVGRNVGAGMTGGLAYFLDEVGDLPE
KINPEIITLQRITASKGEEQLKSLITAHVEHTGSPKGKAILANWSDYLGKFWQAVPPSEK
DSPEANNDVSLTGEKTLTSV
>1KEAA 221 XRAY 2.00 0.212 0.248 no Possible G-T mismatches repair enzyme <UNP GTMR_METTF> [METHANOTHERMOBACTER THERMAUTOTROPHICUS]
MDDATNKKRKVFVSTILTFWNTDRRDFPWRHTRDPYVILITEILLRRTTAGHVKKIYDKF
FVKYKCFEDILKTPKSEIAKDIKEIGLSNQRAEQLKELARVVINDYGGRVPRNRKAILDL
PGVGKYTCAAVMCLAFGKKAAMVDANFVRVINRYFGGSYENLNYNHKALWELAETLVPGG
KCRDFNLGLMDFSAIICAPRKPKCEKCGMSKLCSYYEKCST
>3CU2A 237 XRAY 1.91 0.168 0.205 no Ribulose-5-phosphate 3-epimerase <UNP Q0I165_HAES1> [HAEMOPHILUS SOMNUS]
GMSKLSLIQQLKQQKLSVGILSANWLQLNEEVTTLLENQINVLHFDIADGQFSSLFTVGA
IGIKYFPTHCFKDVHLMVRNQLEVAKAVVANGANLVTLQLEQYHDFALTIEWLAKQKTTY
ANQVYPVLIGACLCPETPISELEPYLDQIDVIQLLTLDPRNGTKYPSELILDRVIQVEKR
LGNRRVEKLINIDGSMTLELAKYFKQGTHQIDWLVSGSALFSGELKTNLKVVWKSSIM
>2YBXA 394 XRAY 2.56 0.191 0.238 no PHOSPHATIDYLINOSITOL-5-PHOSPHATE 4-KINASE TYPE-2 ALPHA <UNP PI42A_HUMAN> [HOMO SAPIENS]
MHHHHHHSSGVDLGTENLYFQSMDPLLSVLMWGVNHSINELSHVQIPVMLMPDDFKAYSK
IKVDNHLFNKENMPSHFKFKEYCPMVFRNLRERFGIDDQDFQNSLTRSAPLPNDSQARSG
ARFHTSYDKRYIIKTITSEDVAEMHNILKKYHQYIVECHGITLLPQFLGMYRLNVDGVEI
YVIVTRNVFSHRLSVYRKYDLKGSTVAREASDKEKAKELPTLKDNDFINEGQKIYIDDNN

KKVFLEKLKKDVEFLAQLKLMDYSLLVGIHDVERAEQEEVECEENDGEEEGESDGTHPVG
TPPDSPGNTLNSSPPLAPGEFDPNIDVYGIKCHENSPRKEVYFMAIIDILTHYDAKKKAA
HAAKTVKHGAGAEISTVNPEQYSKRFLDFIGHIL
>1Y66A 52 XRAY 1.65 0.190 0.228 no engrailed homeodomain <PDB 1Y66> [ESCHERICHIA COLI]
MKQWSEEVERKLKEFVRRHQEITQETLHEYAQKLGLNQQAIEQFFREFEQRK
>1TUWA 109 XRAY 1.90 0.199 0.245 no Tetracenomycin polyketide synthesis protein tcmI <UNP TCMI_STRGA> [STREPTOMYCES GLAUCESCENS]
MAYRALMVLRMDPADAEHVAAAFAEHDTTELPLEIGVRRRVLFRFHDLYMHLIEADDDIM
ERLYQARSHPLFQEVNERVGQYLTPYAQDWEELKDSKAEVFYSWTAPDS
>1HQZ1 141 XRAY 2.10 0.215 0.259 no ACTIN-BINDING PROTEIN <UNP ABP1_YEAST> [SACCHAROMYCES CEREVISIAE]
MALEPIDYTTHSREIDAEYLKIVRGSDPDTTWLIISPNAKKEYEPESTGSSFHDFLQLFD
ETKVQYGLARVSPPGSDVEKIIIIGWCPDSAPLKTRASFAANFAAVANNLFKGYHVQVTA
RDEDDLDENELLMKISNAAGA
>3BQPA 80 XRAY 1.30 0.177 0.234 no Proactivator polypeptide <UNP SAP_HUMAN> [HOMO SAPIENS]
DGGFCEVCKKLVGYLDRNLEKNSTKQEILAALEKGCSFLPDPYQKQCDQFVAEYEPVLIE
ILVEVMDPSFVCLKIGACPS
>1IXMA 192 XRAY 2.60 0.217 0.314 no SPORULATION RESPONSE REGULATORY PROTEIN <UNP SPOB_BACSU> [BACILLUS SUBTILIS]
MKDVSKNQEENISDTALTNELIHLLGHSRHDWMNKLQLIKGNLSLQKYDRVFEMIEEMVI
DAKHESKLSNLKTPHLAFDFLTFNWKTHYMTLEYEVLGEIKDLSAYDQKLAKLMRKLFHL
FDQAVSRESENHLTVSLQTDHPDRQLILYLDFHGAFADPSAFDDIRQNGYEDVDIMRFEI
TSHECLIEIGLD
>1M15A 357 XRAY 1.20 0.125 0.140 no arginine kinase <UNP KARG_LIMPO> [LIMULUS POLYPHEMUS]
MVDQATLDKLEAGFKKLQEASDCKSLLKKHLTKDVFDSIKNKKTGMGATLLDVIQSGVEN
LDSGVGIYAPDAESYRTFGPLFDPIIDDYHGGFKLTDKHPPKQWGDINTLVGLDPAGQFI
ISTRVRCGRSLQGYPFNPCLTAEQYKEMEEKVSSTLSSMEDELKGTYYPLTGMSKATQQQ
LIDDHFLFKEGDRFLQTANACRYWPTGRGIFHNDAKTFLVWVNEEDHLRIISMQKGGDLK
TVYKRLVTAVDNIESKLPFSHDDRFGFLTFCPTNLGTTMRASVHIQLPKLAKDRKVLEDI
ASKFNLQVRGTRGEHTESEGGVYDISNKRRLGLTEYQAVREMQDGILEMIKMEKAAA
>1RT8A 513 XRAY 2.00 0.237 0.270 no fimbrin <UNP FIMB_SCHPO> [SCHIZOSACCHAROMYCES POMBE]
GSPEFMHTINEEERREFIKHINSVLAGDPDVGSRVPINTETFEFFDQCKDGLILSKLIND
SVPDTIDERVLNKQRNNKPLDNFKCIENNNVVINSAKAMGGISITNIGAGDILEGREHLI
LGLVWQIIRRGLLGKIDITLHPELYRLLEEDETLDQFLRLPPEKILLRWFNYHLKAANWP
RTVSNFSKDVSDGENYTVLLNQLAPELCSRAPLQTTDVLQRAEQVLQNAEKLDCRKYLTP
TAMVAGNPKLNLAFVAHLFNTHPGLEPLNEEEKPEIEPFDAEGEREARVFTLWLNSLDVT
PSIHDFFNNLRDGLILLQAYDKITPNTVNWKKVNKAPASGDEMMRFKAVENCNYAVDLGK
NQGFSLVGIQGADITDGSRTLTLALVWQMMRMNITKTLHSLSRGGKTLSDSDMVAWANSM
AAKGGKGSQIRSFRDPSISTGVFVLDVLHGIKSEYVDYNLVTDGSTEELAIQNARLAISI
ARKLGAVIFILPEDIVAVRPRLVLHFIGSLMAV
>3LFRA 136 XRAY 1.53 0.177 0.212 no putative Metal ion transporter <UNP Q87VX8_PSESM> [PSEUDOMONAS SYRINGAE]
ADLQVRDIMVPRSQMISIKATQTPREFLPAVIDAAHSRYPVIGESHDDVLGVLLAKDLLP
LILKADGDSDDVKKLLRPATFVPESKRLNVLLREFRANHNHMAIVIDEYGGVAGLVTIED
VLEQIVGDIEDEHDVE
>3D59A 383 XRAY 1.50 0.142 0.191 no Platelet-activating factor acetylhydrolase <UNP PAFA_HUMAN> [HOMO SAPIENS]
AAASFGQTKIPRGNGPYSVGCTDLMFDHTNKGTFLRLYYPSQDNDRLDTLWIPNKEYFWG

LSKFLGTHWLMGNILRLLFGSMTTPANWNSPLRPGEKYPLVVFSHGLGAFRTLYSAIGID
LASHGFIVAAVEHRDRSASATYYFKDQSAAEIGDKSWLYLRTLKQEEETHIRNEQVRQRA
KECSQALSLILDIDHGKPVKNALDLKFDMEQLKDSIDREKIAVIGHSFGGATVIQTLSED
QRFRCGIALDAWMFPLGDEVYSRIPQPLFFINSEYFQYPANIIKMKKCYSPDKERKMITI
RGSVHQNFADFTFATGKIIGHMLKLKGDIDSNVAIDLSNKASLAFLQKHLGLHKDFDQWD
CLIEGDDENLIPGTNINTTNQHI
>1RORI 51 XRAY 1.10 0.159 0.184 no Ovomucoid <UNP IOVO_MELGA> [MELEAGRIS GALLOPAVO]
VDCSEYPKPACTLEYRPLCGSDNKTYGNKCNFCNAVVESNGTLTLSHFGKC
>3D5NA 197 XRAY 2.80 0.231 0.266 no Q97W15_SULSO <UNP Q97W15_SULSO> [SULFOLOBUS SOLFATARICUS]
MNIGVIILAAGEGKRFGGDKLLAKIDNTPIIMRTIRIYGDLEKIIIVGKYVNEMLPLLMD
QIVIYNPFWNEGISTSLKLGLRFFKDYDAVLVALGDMPFVTKEDVNKIINTFKPNCKAVI
PTHKGERGNPVLISKSLFNEIEKLRGDVGARVILNKIKIEELCFIECSEGVLIDIDKKED
LMRLRDFHPLEHHHHHH
>1XRKA 124 XRAY 1.50 0.175 0.194 no Bleomycin resistance protein <UNP BLE_STRHI> [STREPTOALLOTEICHUS HINDUSTANUS]
MAKLTSAVPVLTARDVAEAVEFWTDRLGFSRVFVEDDFAGVVRDDVTLFISAVQDQVVPD
NTQAWVWVRGLDELYAEWSEVVSTNFRDASGPAMTEIVEQPWGREFALRDPAGNCVHFVA
EEQD
>3KG7A 293 XRAY 2.77 0.208 0.269 no CurH <UNP Q6DNE5_9CYAN> [LYNGBYA MAJUSCULA]
SNASGGQQVHRLLGNKLELASTGQTIYHQDINLNNHPWIGDHRVYDTPVIPGVSYIAMTLA
AVGVPAAVEDINFQQPLFLAESNTTRETQLMLHTADNVGKQFVEVFSRDGAKQEEWQQHA
SMSVSENPPPPPTLSVDIPALCEQLRPLDTDTLTEIYASISLVYGPMLQAVRQAWIGEET
SLLEIEVPKALAFQLAGEPIHPVLIDACTRLTPDLFDFSSDSGVFWAPWRVKEMTLSHPT
PSRFYAYVEEPSRVNEQLQTRSYDIQLLDETGQAFGRINGFTVKRAPSQLFLK
>3LXZA 229 XRAY 1.76 0.197 0.229 no Glutathione S-transferase family protein <UNP Q88RE7_PSEPK> [PSEUDOMONAS PUTIDA]
MSLKLYGFSVSNYYNMVKLALLEKGLTFEEVTFYGGQAPQALEVSPRGKVPVLETEHGFL
SETSVILDYIEQTQGGKALLPADPFGQAKVRELLKEIELYIELPARTCYAESFFGMSVEP
LIKEKARADLLAGFATLKRNGRFAPYVAGEQLTLADLMFCFSVDLANAVGKKVLNIDFLA
DFPQAKALLQLMGENPHMPRILADKEASMPAFMEMIRSGKREGHHHHHH
>3EBEA 200 XRAY 2.30 0.205 0.247 no Protein MCM10 homolog <UNP MCM10_XENLA> [XENOPUS LAEVIS]
GPSPVGQQYHVEKFSGLRIRKPRVSSSEMERKMNGRKLIRLAQLQNKIATEKLEEEDWVT
FGVIVKKITPQSSNNGKTFSIWRLNDLKDLDKYISLFLFGDVHKEHWKTDQGTVIGLLNA
NPMKPKEGTDEVCLSVDNPQKVLLMGDAVDLGTCKARKKNGDPCTQMVNLNDCEYCQYHV
QAQYKKVSSKRADLQSSYSG
>2BJQA 345 XRAY 1.75 0.191 0.230 no MFP2A <UNP Q7YXK2_ASCSU> [ASCARIS SUUM]
MTTKEFEDTWAYNTIGSPFPDNPVRVKGQQNMYVALWYKFGKPIHGRAWNDNGNVECSFP
YNKVELTGARDLGGQIQILTATEQDPTEQFKKTGFWYEWRPYKDRVNDQLLQLVRCGQST
PVIMKTKDGKDLLGYIDMSTEVAAVGVSGKSEQVAGGPIQDMLVLFRNVKAPPKGIKIYD
DTWLDLKYRDPFPAARNPIAAGGRKVKSDDGTEMFQYVALWYEHGQPVFGRAYPDSADKT
LANFGWGGQENAGAEIGSFQMLVVPDPDILGFEYKWIPYKEAKAGGPFKPLHVGECTPCL
LKDANGTERLGNLHMGMEKATAGLAGKDSAVSGPAVGDFLVLCRN
>4GAKA 250 XRAY 1.90 0.187 0.224 no Acyl-ACP thioesterase <UNP D2QU30_SPILD> [SPIROSOMA LINGUALE]
SNAMAFIQTDTFTLRGYECDAFGRMSIPALMNLMQESANRNAIDYGIGIADLAQKGVGWM
LMRFCLRIHQYPRYGDTIQLMTYPTTVDKYFIHRDFRVLATDGTLLADARSTWLVFSMEK
RSMVPLPDFIRQLSPPANVDPLPALPLKPDFQTASFATAASKSVQVGWLNIDQNQHVNNV
AYVQWLLEGVDSEIVQTREIAEIDLVYRTESHWHDWLSVQSVTETDNSVLHRISQTESGK

DVLLARSRWR
>4K7RA 446 XRAY 2.09 0.216 0.250 no Cation efflux system protein CusC <UNP CUSC_ECOLI>
[ESCHERICHIA
COLI]
CSLAPDYQRPAMPVPQQFSLSQNGLVNAADNYQNAGWRTFFVDNQVKTLISEALVNNRDL
RMATLKVQEARAQYRLTDADRYPQLNGEGSGSWSGNLKGNTATTREFSTGLNASFDLDFF
GRLKNMSEAERQNYLATEEAQRAVHILLVSNVAQSYFNQQLAYAQLQIAEETLRNYQQSY
AFVEKQLLTGSSNVLALEQARGVIESTRSDIAKRQGELAQANNALQLLLGSYGKLPQAQT
VNSDSLQSVKLPAGLSSQILLQRPDIMEAEHALMAANANIGAARAAFFPSISLTSGISTA
SSDLSSLFNASSGMWNFIPKIEIPIFNAGRNQANLDIAEIRQQQSVVNYEQKIQNAFKEV
ADALALRQSLNDQISAQQRYLASLQITLQRARALYQHGAVSYLEVLDAERSLFATRQTLL
DLNYARQVNEISLYTALGGGHHHHHH
>3AABA 123 XRAY 1.85 0.228 0.238 no Putative uncharacterized protein ST1653 <UNP Q970D9_SULTO> [SULFOLOBUS TOKODAII]
MYYLGKELQKRSEELSRGFYELVYPPVDMYEEGGYLVVVADLAGFNKEKIKARVSGQNEL
IIEAEREITEPGVKYLTQRPKYVRKVIRLPYNVAKDAEISGKYENGVLTIRIPIAGTSVF
KFE
>3GVZA 299 XRAY 2.80 0.259 0.290 no Uncharacterized protein CV2077 <UNP Q7NWB3_CHRVO>
[CHROMOBACTERIUM VIOLACEUM ATCC 12472]
MNVMRHSWLAVAGIAGLLAFPPVDACTLWGAAGTASMEGSLLAKNRDWKPDHAQSLRLLH
PEHGYAYLGLYADNGSEPGIKAGVNQKGLAVVAAEASSLPRALRADSARHGVLTRLLRDY
GSLDEVASAADKLFAQARPVFLLLADAGGLMQVEIGQHGRYRLIRQQSGTLAHTNHYADT
SLLDGAQTIGPSSQARLERIRFLLDQHPAHTLSEFERLSRDRHDGPDNSLWRSGREHTLA
GWRIALPAGAPPRLQLTLANPGRAERDGDYALDSAFWAQPARTLLPKLAAALEHHHHHH
>1DJUA 388 XRAY 2.10 0.185 0.254 no AROMATIC AMINOTRANSFERASE <UNP O59096_PYRHO>
[PYROCOCCUS HORIKOSHII]
ALSDRLELVSASEIRKLFDIAAGMKDVISLGIGEPDFDTPQHIKEYAKEALDKGLTHYGP
NIGLLELREAIAEKLKKQNGIEADPKTEIMVLLGANQAFLMGLSAFLKDGEEVLIPTPAF
VSYAPAVILAGGKPVEVPTYEEDEFRLNVDELKKYVTDKTRALIINSPCNPTGAVLTKKD
LEEIADFVVEHDLIVISDEVYEHFIYDDARHYSIASLDGMFERTITVNGFSKTFAMTGWR
LGFVAAPSWIIERMVKFQMYNATCPVTFIQYAAAKALKDERSWKAVEEMRKEYDRRRKLV
WKRLNEMGLPTVKPKGAFYIFPRIRDTGLTSKKFSELMLKEARVAVVPGSAFGKAGEGYV
RISYATAYEKLEEAMERMERVLKERKLV
>3DR2A 305 XRAY 1.67 0.211 0.239 no Exported gluconolactonase <PDB 3DR2>
[XANTHOMONAS CAMPESTRIS PV. CAMPESTRIS]
MDSHCRVRPAGPAVPADCDPPRITHAALAARLGDARLLTLYDQATWSEGPAWWEAQRTLV
WSDLVGRRVLGWREDGTVDVLLDATAFTNGNAVDAQQRLVHCEHGRRAITRSDADGQAHL
LVGRYAGKRLNSPNDLIVARDGAIWFTDPPFGLRKPSQGCPADPELAHHSVYRLPPDGSP
LQRMADLDHPNGLAFSPDEQTLYVSQTPEQGHGSVEITAFAWRDGALHDRRHFASVPDGL
PDGFCVDRGGWLWSSSGTGVCVFDSDGQLLGHIPTPGTASNCTFDQAQQRLFITGGPCLW
MLPLP
>2O62A 270 XRAY 1.75 0.168 0.208 no hypothetical protein <PDB 2O62> [NOSTOC PUNCTIFORME]
GMKSQWECFLQNLGVWEGSFSNFSPEGTLLNDTSSRLCLEGLNNNQTVRLTLSRSGKDDV
IREFRSVGGGLLFFENGSFSEGLIQLGPFSEFGGELAFVHENRRLRLVQLFDRNGHLNGL
TLIREHLAGTPVAERPLLQINDLLGEWRGQAVTIYRDLRPPDIYSTTLKIQLDDAGRLMQ
STSFGERTITSTATIKGSIVLFDQDPEKQVQVLLLPDGASATSPLKVQLRQPLFLEAGWL
IQSDLRQRMIRSYNDKGEWVSLTLVTEERV
>2B2AA 199 XRAY 2.22 0.238 0.258 no Telomerase reverse transcriptase <UNP TERT_TETTH>

[TETRAHYMENA THERMOPHILA]
MKKHHHHHHQKINNINNNKQMLTRKEDLLTVLKQISALKYVSNLYEFLLATEKIVQTSEL
DTQFQEFLTTTIIASEQNLVENYKQKYNQPNFSQMTIKQVIDDSIILLGNKQNYVQQIGT
TTIGFYVEYENINLSRQTLYSSNFRNLLNIFGEEDFKYFLIDFLVFTKVEQNGYLQVAGV
CLNQYFSVQVKQKKWYKNN
>1GKPA 458 XRAY 1.29 0.154 0.184 no HYDANTOINASE <PDB 1GKP> [THERMUS SP.]
PLLIKNGEIITADSRYKADIYAEGETITRIGQNLEAPPGTEVIDATGKYVFPGFIDPHVH
IYLPFMATFAKDTHETGSKAALMGGTTTYIEMCCPSRNDDALEGYQLWKSKAEGNSYCDY
TFHMAVSKFDEKTEGQLREIVADGISSFKIFLSYKNFFGVDDGEMYQTLRLAKELGVIVT
AHCENAELVGRLQQKLLSEGKTGPEWHEPSRPEAVEAEGTARFATFLETTGATGYVVHLS
CKPALDAAMAAKARGVPIYIESVIPHFLLDKTYAERGGVEAMKYIMSPPLRDKRNQKVLW
DALAQGFIDTVGTDHCPFDTEQKLLGKEAFTAIPNGIPAIEDRVNLLYTYGVSRGRLDIH
RFVDAASTKAAKLFGLFPRKGTIAVGSDADLVVYDPQYRGTISVKTQHVNNDYNGFEGFE
IDGRPSVVTVRGKVAVRDGQFVGEKGWGKLLRREPMYF
>1N71A 180 XRAY 1.80 0.208 0.245 no aac(6')-Ii <UNP Q47764_ENTFC> [ENTEROCOCCUS FAECIUM]
MIISEFDRNNPVLKDQLSDLLRLTWPEEYGDSSAEEVEEMMNPERIAVAAVDQDELVGFI
GAIPQYGITGWELHPLVVESSRRKNQIGTRLVNYLEKEVASRGGITIYLGTDDLDHGTTL
SQTDLYEHTFDKVASIQNLREHPYEFYEKLGYKIVGVLPNANGWDKPDIWMAKTIIPRPD
>2XMJA 64 XRAY 1.08 0.117 0.149 no SSR2857 PROTEIN <UNP P73213_SYNY3> [SYNECHOCYSTIS SP. PCC 6803]
MTIQLTVPTIACEACAEAVTKAVQNEDAQATVQVDLTSKKVTITSALGEEQLRTAIASAG
HEVE
>3CZ1A 119 XRAY 1.50 0.153 0.198 no Pheromone-binding protein ASP1 <UNP Q9U9J6_APIME> [APIS MELLIFERA]
APDWVPPEVFDLVAEDKARCMSEHGTTQAQIDDVDKGNLVNEPSITCYMYCLLEAFSLVD
DEANVDEDIMLGLLPDQLQERAQSVMGKCLPTSGSDNCNKIYNLAKCVQESAPDVWFVI
>3EBWA 163 XRAY 2.80 0.231 0.276 no Per a 4 allergen <UNP Q1M0Y5_PERAM> [PERIPLANETA AMERICANA]
DDSCQIGTSFTGLDMTKYVGTWYELFRTPNSDEEDFTNCEYDKYTLDENGVIQVTSVAYT
NSIRGFITSTGTVPSWTEDTFDIAYGDDETWSSTYFMVGTDYQTYSIVAGCLDNDYSRHL
YWIASHETSFDDATKAKVNEVLAPYNLSLDDMEPVDQSYCVQY
>3LLKA 261 XRAY 2.00 0.237 0.272 no Sulfhydryl oxidase 1 <UNP QSOX1_HUMAN> [HOMO SAPIENS]
IAPTVWKLADRSKIYMADLESALHYILRIEVGRFPVLEGQRLVALKKFVAVLAKYFPGRP
LVQNFLHSVNEWLKRQKRNKIPYSFFKTALDDRKEGAVLAKKVNWIGCQGSEPHFRGFPC
SLWVLFHFLTVQAARQNVDHSQEAAKAKEVLPAIRGYVHYFFGCRDCASHFEQMAAASMH
RVGSPNAAVLWLWSSHNRVNARLAGAPSEDPQFPKVQWPPRELCSACHNERLDVPVWDVE
ATLNFLKAHFSPSNIILDFPA
>3K4IA 244 XRAY 1.69 0.180 0.214 no uncharacterized protein <UNP Q880F6_PSESM> [PSEUDOMONAS SYRINGAE PV. TOMATO]
MSLSVPFEYTPIAQSVLDECEHLDTASLSDALDSLGIDGGLPGIASQVPGTRCVGIAFTV
QYQPVDASEGFRGAANYIDQVPSGSVIVSSNSGRHDCTVWGDIMTHFALANGIKGTVIDG
VARDIDTVINCNYPLFSRGRFMQSAKNRTQLKAVQVPLVIDGITIQPGDLMVCDGSGCVV
VPQQLAAEVVLRARAVEQTERRIIEAISSGSTLEQARMTYRYDQPWLSEAEHGGTQEGHH
HHHH
>2OSXA 481 XRAY 1.10 0.122 0.139 no Endoglycoceramidase II <UNP O33853_RHOSO> [RHODOCOCCUS SP.]
MGSSHHHHHHSSGLVPRGSHMSGSGSGSGTALTPSYLKDDDGRSLILRGFNTASSAKSAP
DGMPQFTEADLAREYADMGTNFVRFLISWRSVEPAPGVYDQQYLDRVEDRVGWYAERGYK
VMLDMHQDVYSGAITPEGNSGNGAGAIGNGAPAWATYMDGLPVEPQPRWELYYIQPGVMR

AFDNFWNTTGKHPELVEHYAKAWRAVADRFADNDAVVAYDLMNEPFGGSLQGPAFEAGPL
AAMYQRTTDAIRQVDQDTWVCVAPQAIGVNQGLPSGLTKIDDPRAGQQRIAYCPHLYPLP
LDIGDGHEGLARTLTDVTIDAWRANTAHTARVLGDVPIILGSFGLDTTLPGARDYIERVY
GTAREMGAGVSYWSSDPGPWGPYLPDGTQTLLVDTLNKPYPRAVAGTPTEWSSTSDRLQL
TIEPDAAITAPTEIYLPEAGFPGDVHVEGADVVGWDRQSRLLTVRTPADSGNVTVTVTPA
A
>3GUYA 230 XRAY 1.90 0.202 0.249 no Short-chain dehydrogenase/reductase SDR <UNP A6B7Q2_VIBPA>
[VIBRIO PARAHAEMOLYTICUS]
MSLIVITGASSGLGAELAKLYDAEGKATYLTGRSESKLSTVTNCLSNNVGYRARDLASHQ
EVEQLFEQLDSIPSTVVHSAGSGYFGLLQEQDPEQIQTLIENNLSSAINVLRELVKRYKD
QPVNVVMIMSTAAQQPKAQESTYCAVKWAVKGLIESVRLELKGKPMKIIAVYPGGMATEF
WETSGKSLDTSSFMSAEDAALMIHGALANIGNGYVSDITVNREGHHHHHH
>3R62A 129 XRAY 1.52 0.179 0.208 no Complement factor H <UNP CFAH_HUMAN> [HOMO SAPIENS]
EAEFGKCGPPPPIDNGDITSFPLSVYAPASSVEYQCQNLYQLEGNKRITCRNGQWSEPPK
CLHPCVISREIMENYNIALRWRAKQKLYSRTGESVEFVCKRGYRLSSRSHTLRTTCWDGK
LEYPTCAKR
>3DK9A 478 XRAY 0.95 0.123 0.152 no Glutathione reductase <UNP GSHR_HUMAN> [HOMO SAPIENS]
ACRQEPQPQGPPPAAGAVASYDYLVIGGGSGGLASARRAAELGARAAVVESHKLGGTCVN
VGCVPKKVMWNTAVHSEFMHDHADYGFPSCEGKFNWRVIKEKRDAYVSRLNAIYQNNLTK
SHIEIIRGHAAFTSDPKPTIEVSGKKYTAPHILIATGGMPSTPHESQIPGASLGITSDGF
FQLEELPGRSVIVGAGYIAVEMAGILSALGSKTSLMIRHDKVLRSFDSMISTNCTEELEN
AGVEVLKFSQVKEVKKTLSGLEVSMVTAVPGRLPVMTMIPDVDCLLWAIGRVPNTKDLSL
NKLGIQTDDKGHIIVDEFQNTNVKGIYAVGDVCGKALLTPVAIAAGRKLAHRLFEYKEDS
KLDYNNIPTVVFSHPPIGTVGLTEDEAIHKYGIENVKTYSTSFTPMYHAVTKRKTKCVMK
MVCANKEEKVVGIHMQGLGCDEMLQGFAVAVKMGATKADFDNTVAIHPTSSEELVTLR
>2QVKA 192 XRAY 1.45 0.163 0.192 no Sodium/calcium exchanger 1 <UNP NAC1_CANFA> [CANIS LUPUS FAMILIARIS]
GPGHAGIFTFEEPVTHVSESIGIMEVKVLRTSGARGNVIVPYKTIEGTARGGGEDFEDTC
GELEFQNDEIVKTISVKVIDDEEYEKNKTFFLEIGEPRLVEMSEKKALLLNELGGFTITG
KYLYGQPVFRKVHAREHPIPSTVITIAEEYDDKQPLTSKEEEERRIAEMGRPILGEHTKL
EVIIEESYEFKS
>3TEEA 219 XRAY 1.95 0.201 0.218 no Flagella basal body P-ring formation protein flgA <UNP FLGA_SALTY> [SALMONELLA TYPHIMURIUM]
QDINAQLTTWFSQRLAGFSDEVVVTLRSSPNLLPSCEQPAFSMTGSAKLWGNVNVVARCA
NEKRYLQVNVQATGNYVAVAAPIARGGKLTPANVTLKRGRLDQLPPRTVLDIRQIQDAVS
LRDLAPGQPVQLTMIRQAWRVKAGQRVQVIANGEGFSVNAEGQAMNNAAVAQNARVRMTS
GQIVSGTVDSDGNILINLDPNSSSVDKLAAALEHHHHHH
>3HRZA 627 XRAY 2.20 0.181 0.226 no Cobra venom factor <UNP CO3_NAJKA> [NAJA KAOUTHIA] ALYTLITPAVLRTDTEEQILVEAHGDSTPKQLDIFVHDFPRKQKTLFQTRVDMNPAGGML
VTPTIEIPAKEVSTDSRQNQYVVVQVTGPQVRLEKVVLLSYQSSFLFIQTDKGIYTPGSP
VLYRVFSMDHNTSKMNKTVIVEFQTPEGILVSSNSVDLNFFWPYNLPDLVSLGTWRIVAK
YEHSPENYTAYFDVRKYVLPSFEVRLQPSEKFFYIDGNENFHVSITARYLYGEEVEGVAF
VLFGVKIDDAKKSIPDSLTRIPIIDGDGKATLKRDTFRSRFPNLNELVGHTLYASVTVMT
ESGSDMVVTEQSGIHIVASPYQIHFTKTPKYFKPGMPYELTVYVTNPDGSPAAHVPVVSE
AFHSMGTTLSDGTAKLILNIPLNAQSLPITVRTNHGDLPRERQATKSMTAIAYQTQGGSG
NYLHVAITSTEIKPGDNLPVNFNVKGNANSLKQIKYFTYLILNKGKIFKVGRQPRRDGQN
LVTMNLHITPDLIPSFRFVAYYQVGNNEIVADSVWVDVKDTCMGTLVVKGDNLIQMPGAA

MKIKLEGDPGARVGLVAVDKAVYVLNDKYKISQAKIWDTIEKSDFGCTAGSGQNNLGVFE
DAGLALTTSTNLNTKQRSAAKCPQPAN
>2HW2A 143 XRAY 1.45 0.137 0.185 no Rifampin ADP-ribosyl transferase <UNP O67972_MYCSM> [MYCOBACTERIUM SMEGMATIS]
MVANPPKPFEVHESGAYLHGTKAELKVGDRLVPGRESNFEAGRIMNHIYITQTLDAAVWG
AELAAGEGRGRIFIVEPEGAIEDDPNVTDKKLPGNPTRSYRTREPVWIVGELTDWVGHPP
EQLAAMRQGLEELRRKGLAVIYD
>1GG3A 279 XRAY 2.80 0.226 0.276 no ERYTHROID MEMBRANE PROTEIN 4.1R <UNP 41_HUMAN> [HOMO SAPIENS]
MHCKVSLLDDTVYECVVEKHAKGQDLLKRVCEHLNLLEEDYFGLAIWDNATSKTWLDSAK
EIKKQVRGVPWNFTFNVKFYPPDPAQLTEDITRYYLCLQLRQDIVAGRLPCSFATLALLG
SYTIQSELGDYDPELHGVDYVSDFKLAPNQTKELEEKVMELHKSYRSMTPAQADLEFLEN
AKKLSMYGVDLHKAKDLEGVDIILGVCSSGLLVYKDKLRINRFPWPKVLKISYKRSSFFI
KIRPGEQEQYESTIGFKLPSYRAAKKLWKVCVEHHTFFR
>3HIMA 211 XRAY 2.20 0.200 0.248 no Probable transcriptional regulator <UNP Q0SD48_RHOSR> [RHODOCOCCUS JOSTII]
MDASLTGAVAELGTSKAAARIRAAAIEVFAAKGYGATTTREIAASLDMSPGAVYPHYKTK
ESLLYAISLEGHHSVLAAITAADFPDIAAPDRLMSTVTAYVTWHADNRASARVGQYELRS
LSPEHFAIIADIRRSTTKVFTRIIEAGATAGDFHPFDIEAAALAITSLGIDVSRWFPSHT
YSDPRIIAARYVELALRMVGCADRQPLDKPS
>2V89A 82 XRAY 1.10 0.123 0.155 no VDJ RECOMBINATION-ACTIVATING PROTEIN 2 <UNP RAG2_MOUSE> [MUS MUSCULUS]
GPLGSPEFGYWITCCPTCDVDINTWVPFYSTELNKPAMIYCSHGDGHWVHAQCMDLEERT
LIHLSEGSNKYYCNEHVQIARA
>4FWGA 732 XRAY 1.99 0.231 0.259 no TTC1975 peptidase <UNP C9DRU9_9DEIN> [MEIOTHERMUS TAIWANENSIS]
MRLSYEALEWRTPIENSTEPVSLPPPPPFFGQERAREALELAIRGGFHAYLVGPPSLGKH
EALLAYLSTQSVETPPDLLYVPLSERKVAVMTLPSGQEIHLAEAVEGLLLEVNRLDELFR
QGSFLREKTQLEARFKEAREQQLEALRREAQEAGFALSTNGERLELTGPGPVPAELSARL
EEVTLGSMAASAELEVALRRLRRDWALHYLNNRFEPLFQRFPQARAYLEALRARLARYAE
TGEPLDPAQWRPNLLTSSSSGTPPPIVYEPYATAPRLFGRLDYLVDRGVWSTNVSLIRPG
AVHRAQGGYLILDALSLKREGTWEAFKRALRNGQVEPVTEPQAPAGLEVEPFPIQMQVML
VGTPEAFEGLEEDPAFSELFRIRAEFSPTMPASPENCTALGGWLLAQGFQLTQGGLTRLY
DEARRMAEQRDRMDARLVEIRALAEEAAVLGGGLLTAESVEQAIAAREHRSFLSEEEFLR
AVQEGVIRLRTTGRAVGEVNSLVVVEAAPYWGRPARLTARAAPGRDHLISIDREAGLGGQ
IFHKAVLTLAGYLRSRMIEHGSLPVTISLAFEQNYVSIEGDSAGLAELVAALSAIGNLPL
RQDLAVTGAVDQTGKVLAVGAINAKVEGFFRVCKALGLSGTQGVILPEANLANLTLRAEV
LEAVRAGQFHIYAVETAEQALEILAGARMEGFRGLQEKIRAGLEAFARLEEGHDKEDREK
LAAALEHHHHHH
>1O7DE 126 XRAY 2.70 0.257 0.289 no LYSOSOMAL ALPHA-MANNOSIDASE <UNP M2B1_BOVIN> [BOS TAURUS]
PRTQFSGLRRELPPSVRLLTLARWGPETLLLRLEHQFAVGEDSGRNLSSPVTLDLTNLFS
AFTITNLRETTLAANQLLAYASRLQWTTDTGPTPHPSPSRPVSATITLQPMEIRTFLASV
QWEEDG
>4JYKA 212 XRAY 1.70 0.122 0.170 no HTH-type transcriptional regulator RutR <UNP RUTR_ECOLI> [ESCHERICHIA COLI]
MTQGAVKTTGKRSRAVSAKKKAILSAALDTFSQFGFHGTRLEQIAELAGVSKTNLLYYFP

SKEALYIAVLRQILDIWLAPLKAFREDFAPLAAIKEYIRLKLEVSRDYPQASRLFCMEML
AGAPLLMDELTGDLKALIDEKSALIAGWVKSGKLAPIDPQHLIFMIWASTQHYADFAPQV
EAVTGATLRDEVFFNQTVENVQRIIIEGIRPR
>3C5PA 197 XRAY 2.90 0.178 0.226 no Protein BAS0735 of unknown function <UNP Q81UT7_BACAN>
[BACILLUS ANTHRACIS STR.] SNAMTNIIKIRASVFIPMSWTEAKMDMETGQVIQFEGDSREFTPHAVNTMRSRVEQEVVV
DFYKQEVFSYANTGITTEKVISPDGSVNKRTGKASTENIVCTDIVWNSGGVQFKMSASAS
NPLNVYAPPVDYVLNVCVKKDGSIDVQGEHDGFPCFEFYKQVDFGPFEKIYTHDFRETGD
TAAALGGNMDYSFTKRL
>3B9WA 407 XRAY 1.30 0.151 0.171 no Ammonium transporter family <UNP Q82X47_NITEU> [NITROSOMONAS
EUROPAEA] SAVAPAEINEARLVAQYNYSINILAMLLVGFGFLMVFVRRYGFSATTGTYLVVATGLPLY
ILLRANGIFGHALTPHSVDAVIYAEFAVATGLIAMGAVLGRLRVFQYALLALFIVPVYLL
NEWLVLDNASGLTEGFQDSAGSIAIHAFGAYFGLGVSIALTTAAQRAQPIESDATSDRFS
MLGSMVLWLFWPSFATAIVPFEQMPQTIVNTLLALCGATLATYFLSALFHKGKASIVDMA
NAALAGGVAIGSVCNIVGPVGAFVIGLLGGAISVVGFVFIQPMLESKAKTIDTCGVHNLH
GLPGLLGGFSAILIVPGIAVAQLTGIGITLALALIGGVIAGALIKLTGTTKQAYEDSHEF
IHLAGPEDEHKAERLVLEAKTEIQGLKNRIDAAVLSAKSEGHHHHHH
>4B4DA 262 XRAY 1.50 0.209 0.230 no FERREDOXIN-NADP REDUCTASE <UNP Q8PMH0_XANAC>
[XANTHOMONAS AXONOPODIS PV. CITRI STR. 306]
GSHMSSAFGAETVLEVRHWTDAYFSFTTTRDAGFRFENGQFVMIGLETETRPLLRAYSIA
SANWEEHLEFFSIKVPDGPLTSRLQHIQPGDKVLVGKKPTGTLLISDLHPGRNLYLLGTG
TGLAPWLSIIKDPETYERFDKVILTQGVRFVQDLAYRDYFERELPQHEFLGDLLREKLLY
YPAVTRETFANQGRLTELMADGRMQQTLGLPTLDPANDRFMICGSPQMLADLRSLLDSRG FQTSPRIGTPGHYVFERAFVEK
>3H7AA 252 XRAY 1.87 0.207 0.241 no short chain dehydrogenase <UNP Q6NDT3_RHOPA>
[RHODOPSEUDOMONAS PALUSTRIS]
MSLTPRNATVAVIGAGDYIGAEIAKKFAAEGFTVFAGRRNGEKLAPLVAEIEAAGGRIVA
RSLDARNEDEVTAFLNAADAHAPLEVTIFNVGANVNFPILETTDRVFRKVWEMACWAGFV
SGRESARLMLAHGQGKIFFTGATASLRGGSGFAAFASAKFGLRAVAQSMARELMPKNIHV
AHLIIDSGVDTAWVRERREQMFGKDALANPDLLMPPAAVAGAYWQLYQQPKSAWTFEMEI
RPYGEGHHHHHH
>3M8JA 111 XRAY 1.40 0.197 0.222 no FocB protein <UNP Q93K76_ECOLX> [ESCHERICHIA COLI]
GAMAQHEVITRGGDAFLLKLRESALSSGSMSEEQFFLLIGISSIHSDRVILAMKDYLVSG
HSRKDVCEKYQMNNGYFSTTLGRLTRLNVLVARLAPYYTDSVSAIAEAASL
>2RHWA 283 XRAY 1.57 0.173 0.200 no 2-hydroxy-6-oxo-6-phenylhexa-2,4-dienoate hydrolase
<UNP BPHD_BURXL> [BURKHOLDERIA XENOVORANS]
LTESSTSKFVKINEKGFSDFNIHYNEAGNGETVIMLHGGGPGAGGWSNYYRNVGPFVDAG
YRVILKDSPGFNKSDAVVMDEQRGLVNARAVKGLMDALDIDRAHLVGNAMGGATALNFAL
EYPDRIGKLILMGPGGLGPSMFAPMPMEGIKLLFKLYAEPSYETLKQMLQVFLYDQSLIT
EELLQGRWEAIQRQPEHLKNFLISAQKAPLSTWDVTARLGEIKAKTFITWGRDDRFVPLD
HGLKLLWNIDDARLHVFSKCGHWAQWEHADEFNRLVIDFLRHA
>2UW1A 338 XRAY 1.95 0.186 0.229 no PLASTID DELTA4 MULTIFUNCTIONAL ACYL-ACYL CARRIER PROTEIN
DESATURASE <PDB 2UW1> [HEDERA HELIX]
MQVTHSMPPQKLEIFKSLDDWARNNVLIHLKSVEKSWQPQDYLPDPVSDGFEEQVRELRE
RAKEIPDDYFVVLVGDMITEEALPTYMSMLNRCDGIKDETGAEPSAWAMWTRAWTAEENR
HGDLLNKYLYLSGRVDMRKIEKTIQYLIGSGMDIKSENSPYLGFIYTSFQERATFISHAN
TAKLAQHYGDKKLAHICGSIASDEKRHATAYTKIVEKLAEIDPDTTVIAFADMMRKKITM

PAHLMYDGSDELLFKHFTAVAQRLGVYSALDYCDILEFLVDKWNVERLTGLSDEGRKAQE
YVCELGPKIRRLEERAQGRAKEAPTMPFSWIFDRQVKL

>1JI1A 637 XRAY 1.60 0.182 0.206 no ALPHA-AMYLASE I <UNP NEPU1_THEVU> [THERMOACTINOMYCES VULGARIS]
AANDNNVEWNGLFHDQGPLFDNAPEPTSTQSVTLKLRTFKGDITSANIKYWDTADNAFHW
VPMVWDSNDPTGTFDYWKGTIPASPSIKYYRFQINDGTSTAWYNGNGPSSTEPNADDFYI
IPNFKTPDWLKNGVMYQIFPDRFYNGDSSNDVQTGSYTYNGTPTEKKAWGSSVYADPGYD
NSLVFFGGDLAGIDQKLGYIKKTLGANILYLNPIFKAPTNHKYDTQDYMAVDPAFGDNST
LQTLINDIHSTANGPKGYLILDGVFNHTGDSHPWFDKYNNFSSQGAYESQSSPWYNYYTF
YTWPDSYASFLGFNSLPKLNYGNSGSAVRGVIYNNSNSVAKTYLNPPYSVDGWRLDAAQY
VDANGNNGSDVTNHQIWSEFRNAVKGVNSNAAIIGEYWGNANPWTAQGNQWDAATNFDGF
TQPVSEWITGKDYQNNSASISTTQFDSWLRGTRANYPTNVQQSMMNFLSNHDITRFATRS
GGDLWKTYLALIFQMTYVGTPTIYYGDEYGMQGGADPDNRRSFDWSQATPSNSAVALTQK
LITIRNQYPALRTGSFMTLITDDTNKIYSYGRFDNVNRIAVVLNNDSVSHTVNVPVWQLS
MPNGSTVTDKITGHSYTVQNGMVTVAVDGHYGAVLAQ

>3OR1B 386 XRAY 1.76 0.193 0.212 no Sulfite reductase beta <PDB 3OR1> [DESULFOVIBRIO GIGAS] MAFISSGYNPAKPMENRITDIGPRKFTEFFPPVIAKNAGNWDYHEILEPGILVHVAKNGD
KVFTVRCGAARLMSTSHIREACEIAKKFCNGHLRFTTRNNIEFMVDNEETLKALVADLKT
RKFAAGSFKFPIGGTGASISNIVHTQGWVYCHTPATDASGPVKAVMDELFEEFTSMRLPA
IVRVSLACCINMCGAVHCSDIGLVGIHRKPPMIDHENLAELCEIPLAVAACPTAAVKPIT
AEVNGQKVKSVAINNDRCMYCGNCYTMCPALPLSDGTGDGIAIMVGGKISNRIKVPSFSK
VVVAFVPNEPPRWPTMAKIVKKIVEVYAEDARKYERIGDWIHRIGWETFYEKTGLEFSHH
CIDDFRDPAYYTWRQSTQFKFVSFDS

>1TU1A 148 XRAY 1.95 0.205 0.237 no hypothetical protein PA0094 <UNP Q9I738_PSEAE> [PSEUDOMONAS AERUGINOSA]
GHMTLYRLHEADLEIPDAWQDQSINIFKLPASGPAREASFVISRDASQGDAPFADYVARQ
LENAEKQLPGFKLHKRWDINIHGHAAVLLDYQWQREGRDLMLRQVFIERRPAVLITTLTT
TPADLPHHEPAWKQAMQTLVPRPTPSGS

>3HNXA 110 XRAY 1.37 0.170 0.210 no Cyanovirin-N-like protein <UNP Q5MK11_TUBBO> [TUBER BORCHII,NEUROSPORA CRASSA,TUBER BORCHII]
GSHMSYADSSRNAVLTNGGRTLRAECRNADGNWVTSELDLDTIIGNNDGHFQWGGQNFTE
TAEDIRFHPKEGAAEQPILRARLRDCNGEFHDRDVNLNRIQNVNGRLVFQ

>3EEBA 209 XRAY 2.10 0.202 0.243 no RTX toxin RtxA <UNP Q9KS12_VIBCH> [VIBRIO CHOLERAE]
ADGKILHNQNVNSWGPITVTPTTDGGETRFDGQIIVQMENDPVVAKAAANLAGKHAESSV
VVQLDSDGNYRVVYGDPSKLDGKLRWQLVGHGRDHSETNNTRLSGYSADELAVKLAKFQQ
SFNQAENINNKPDHISIVGCSLVSDDKQKGFGHQFINAMDANGLRVDVSVRSSELAVDEA
GRKHTKDANGDWVQKAENNKVSLSWDAQG

>2E11A 266 XRAY 1.73 0.188 0.217 no Hydrolase <UNP Q8P8M3_XANCP> [XANTHOMONAS CAMPESTRIS PV. CAMPESTRIS]
MHDLRISLVQGSTRWHDPAGNRDYYGALLEPLAGQSDLVILPETFTSGFSNEAIDKAEDM
DGPTVAWIRTQAARLGAAITGSVQLRTEHGVFNRLLWATPDGALQYYDKRHLFRFGNEHL
RYAAGRERLCVEWKGWRINPQVCYDLRFPVFCRNRFDVERPGQLDFDLQLFVANWPSARA
YAWKTLLRARAIENLCFVAAVNRVGVDGNQLHYAGDSAVIDFLGQPQVEIREQEQVVTTT ISAAALAEHRARFPAMLDGDSFVLGE

>2BC4A 211 XRAY 2.27 0.261 0.268 no HLA class II histocompatibility antigen, DM alpha chain <GB NP_006111> [HOMO SAPIENS]

VPEAPTPMWPDDLQNHTFLHTVYCQDGSPSVGLSEAYDEDQLFFFDFSQNTRVPRLPEFA
DWAQEQGDAPAILFDKEFCEWMIQQIGPKLDGKIPVSRGFPIAEVFTLKPLEFGKPNTLV
CFVSNLFPPMLTVNWQHHSVPVEGFGPTFVSAVDGLSFQAFSYLNFTPEPSDIFSCIVTH EIDRYTAIAYWVPRNALPSDLLEDYKDDDDK
>2CVEA 191 XRAY 1.60 0.215 0.238 no hypothetical protein TTHA1053 <UNP Q5SJF5_THET8> [THERMUS THERMOPHILUS]
MSLTLADKVVYEEEIQKSRFIAKAAPVASEEEALAFLAENREPEATHNGHAYKIGLLYRF
SDDGEPSGTAGRPILHAIEAQGLDRVAVLVVRYFGGVKLGAGGLVRAYGGVAAEALRRAP
KVPLVERVGLAFLVPFAEVGRVYALLEARALKAEETYTPEGVRFALLLPKPEREGFLRAL
LDATRGQVALE
>2R4GA 267 XRAY 1.71 0.202 0.239 no Telomerase reverse transcriptase <UNP TERT_TETTH> [TETRAHYMENA THERMOPHILA] MKGFQFKVIQEKLQGRQFINSDKIKPDHPQTIIKKTLLKEYQSKNFSCQEERDLFLEFTE
KIVQNFHNINFNYLLKKFCKLPENYQSLKSQVKQIVQSENKANQQSCENLFNSLYDTEIS
YKQITNFLRQIIQNCVPNQLLGKKNFKVFLEKLYEFVQMKRFENQKVLDYICFMDVFDVE
WFVDLKNQKFTQKRKYISDKRKILGDLIVFIINKIVIPVLRYNFYITEKHKEGSQIFYYR KPIWKLVSKLTIVKLEEENLEKVEEKL
>3CZ8A 319 XRAY 2.20 0.198 0.258 no Putative sporulation-specific glycosylase ydhD <UNP YDHD_BACSU> [BACILLUS SUBTILIS SUBSP. SUBTILIS STR. 168]
MSLSNYIAGTLSFYVLRNPDLDRELINDYAPYSSSISIFEYHIAPNGDIANQLNDAAAIE
TTWQRRVTPLATITNLTSGGFSTEIVHQVLNNPTARTNLVNNIYDLVSTRGYGGVTIDFE
QVSAADRDLFTGFLRQLRDRLQAGGYVLTIAVPAKTSDNIPWLRGYDYGGIGAVVNYMFI
MAYDWHHAGSEPGPVAPITEIRRTIEFTIAQVPSRKIIIGVPLYGYDWIIPYQPGTVASA
ISNQNAIERAMRYQAPIQYSAEYQSPFFRYSDQQGRTHEVWFEGVRSMSRKMQIVREYRL
QAIGAWQLTLAEGHHHHHH
>1LFBA 99 XRAY 2.80 0.212 0.364 no LIVER TRANSCRIPTION FACTOR (LFB1) <UNP HNF1A_MOUSE> [RATTUS NORVEGICUS]
ARIDPTKKGRRNRFKWGPASQQILFQAYERQKNPSKEERETLVEECNRAECIQRGVSPSQ
AQGLGSNLVTEVRVYNWFANRRKEEAFRHKLAMDTYKLN
>1REPC 251 XRAY 2.60 0.213 0.274 no REPLICATION INITIATION PROTEIN <UNP REPE1_ECOLI> [ESCHERICHIA COLI]
MAETAVINHKKRKNSPRIVQSNDLTEAAYSLSRDQKRMLYLFVDQIRKSDGTLQEHDGIC
EIHVAKYAEIFGLTSAEASKDIRQALKSFAGKEVVFYRPEEDAGDEKGYESFPWFIKPAH
SPSRGLYSVHINPYLIPFFIGLQNRFTQFRLSETKEITNPYAMRLYESLCQYRKPDGSGI
VSLKIDWIIERYQLPQSYQRMPDFRRRFLQVCVNEINSRTPMRLSYIEKKKGRQTTHIVF
SFRDITSMTTG
>2W31A 162 XRAY 1.50 0.168 0.207 no GLOBIN <UNP Q747F6_GEOSL> [GEOBACTER SULFURREDUCENS]
MLTMQEIKAHYRFTDEDAELLGSLFPLAETNKERLADQFYDYLLGIPETAEFLKEDLVLQ
KLKQTHQDWFVSLFAGSYDNRYIHNLQKIGHAHVRVGLNAHYVNVAMNVVRQFTLSIIQD
NFPDPEERRQRREAVEKILDINLDIMSASYREEEMRKFFVSH
>2PTRA 462 XRAY 1.85 0.183 0.216 no Adenylosuccinate lyase <UNP PUR8_ECOLI> [ESCHERICHIA COLI]
MELSSLTAVSPVDGRYGDKVSALRGIFSEYGLLKFRVQVEVRWLQKLAAHAAIKEVPAFA
ADAIGYLDAIVASFSEEDAARIKTIERTTNHDVKAVEYFLKEKVAEIPELHAVSEFIHFA
CTSEDINNLSHALMLKTARDEVILPYWRQLIDGLKDLAVQYRDIPLLSRTAGQPATPSTI
GKEMANVAYRMERQYRQLNQVEILGKINGAVGNYNAHIAAYPEVDWHQFSEEFVTSLGIQ
WNPYTTQIEPHDYIAELFDCVARFNTILIDFDRDVWGYIALNHFKQKTIAGEIGSSTMPH
KVNPIDFENSEGNLGLSNAVLQHLASKLPVSRWQRDLTDSTVLRNLGVGIGYALIAYQST
LKGVSKLEVNRDHLLDELDHNWEVLAEPIQTVMRRYGIEKPYEKLKELTRGKRVDAEGMK

QFIDGLALPEEEKARLKAMTPANYIGRAITMVDELKHHHHHH

>2WN3A 254 XRAY 1.59 0.153 0.180 no DISCOIDIN-1 SUBUNIT A <UNP DIS1A_DICDI> [DICTYOSTELIUM DISCOIDEUM]

AMSTQGLVQLLANAQCHLRTSTNYNGVHTQFNSALNYKNNGTNTIDGSEAWCSSIVDTNQ
YIVAGCEVPRTFMCVALQGRGDADQWVTSYKIRYSLDNVSWFEYRNGAAVTGVTDRNTVV
NHFFDTPIRARSIAIHPLTWNGHISLRCEFYTQPVQSSVTQVGADIYTGDNCALNTGSGK
REVVVPVKFQFEFATLPKVALNFDQIDCTDATNQTRIGVQPRNITTKGFDCVFYTWNENK
VYSLRADYIATALE

>1DYOA 160 XRAY 2.10 0.190 0.250 no ENDO-1,4-BETA-XYLANASE Y <UNP XYNY_CLOTM> [CLOSTRIDIUM THERMOCELLUM]

KPEEPDAGYYYHDTFEGSVGQWTARGPAEVLLSGRTAYKGSESLLVRNRTAAWNGAQRAL
NPRTFVPGNTYCFSVVASFIEGASSTTFCMKLQYVDGSGTQRYDTIDMKTVGPNQWVHLY
NPQYRIPSDATDMYVVYVETADDTINFYIDEAIGAVAGTVI

>1UA4A 455 XRAY 1.90 0.168 0.205 no ADP-dependent glucokinase <UNP GLKA_PYRFU> [PYROCOCCUS FURIOSUS]

MPTWEELYKNAIEKAIKSVPKVKGVLLGYNTNIDAIKYLDSKDLEERIIKAGKEEVIKYS
EELPDKINTVSQLLGSILWSIRRGKAAELFVESCPVRFYMKRWGWNELRMGGQAGIMANL
LGGVYGVPVIVHVPQLSRLQANLFLDGPIYVPTLENGEVKLIHPKEFSGDEENCIHYIYE
FPRGFRVFEFEAPRENRFIGSADDYNTTLFIREEFRESFSEVIKNVQLAILSGLQALTKE
NYKEPFEIVKSNLEVLNEREIPVHLEFAFTPDEKVREEILNVLGMFYSVGLNEVELASIM
EILGEKKLAKELLAHDPVDPIAVTEAMLKLAKKTGVKRIHFHTYGYYLALTEYKGEHVRD
ALLFAALAAAAKAMKGNITSLEEIREATSVPVNEKATQVEEKLRAEYGIKEGIGEVEGYQ
IAFIPTKIVAKPKSTVGIGDTISSSAFIGEFSFTL

>2QZJA 136 XRAY 2.89 0.257 0.285 no Two-component response regulator <UNP Q180B0_CLOD6> [CLOSTRIDIUM DIFFICILE]

MSLQTKILIIDGDKDNCQKLKGFLEEKGISIDLAYNCEEAIGKIFSNKYDLIFLEIILSD
GDGWTLCKKIRNVTTCPIVYMTYINEDQSILNALNSGGDDYLIKPLNLEILYAKVKAILR
RMNSYVNNEGHHHHHH

>3TU8A 210 XRAY 1.04 0.129 0.160 no Burkholderia Lethal Factor 1 (BLF1) <UNP Q63UP7_BURPS> [BURKHOLDERIA PSEUDOMALLEI]

PNSLEAQIRQAMKTGSTLTIEFDQALNQKSPGTLNVFLHPANGGVRIDLDSGNQGEPAKI
LWLPWKQGELQTLQPGSISTVDMLFFTYYLSGCKVFAGDGGPVWHIDAPVEANQFWRRMS
SDEWMEDWEVGTDRQVAYLHRAGQSDSLWNLSAYLEGAAPSTYGRDNLGQAVVGGIVTGR
QQMSLYQYATTSSGSSAWSPLTYTLQQRKQ

>3LTJA 201 XRAY 1.80 0.177 0.227 no AlphaRep-4 <PDB 3LTJ> [SYNTHETIC]

MRGSHHHHHHTDPEKVEMYIKNLQDDSYYVRRAAAYALGKIGDERAVEPLIKALKDEDAW
VRRAAADALGQIGDERAVEPLIKALKDEDGWVRQSAAVALGQIGDERAVEPLIKALKDED
WFVRIAAAFALGEIGDERAVEPLIKALKDEDGWVRQSAADALGEIGGERVRAAMEKLAET
GTGFARKVAVNYLETHKSLIS

>2PONA 172 XRAY 1.41 0.170 0.203 no Hypothetical protein NMB1532 <UNP Q9JYL1_NEIMB> [NEISSERIA MENINGITIDIS]

SNAMNPFETKSVTFAEPIEMLYACHGKVRRFCGQVAMLSDYIAENGCNQIVLQTIRQIAQ
YFNVAAPLHHEDEEENFFPLLLQYAPQAQESVDELLRQHIGLHDNWAAVSAEFAKLEADN
AYVPDEEAFKRFVAGYDVHLAIEEPLFDMGNTFIPKEKLTEIGEIMAARRRK

>3LLOA 143 XRAY 1.57 0.156 0.196 no Prestin <UNP S26A5_RAT> [RATTUS NORVEGICUS]

SPSYTVLGQLPDTDVYIDIDAYEEVKEIPGIKIFQINAPIYYANSDLYSSALKRKTGVNG
SENIHTVILDFTQVNFMDSVGVKTLAGIVKEYGDVGIYVYLAGCSAQVVNDLTSNRFFEN
PALKELLFHSIHDAVLGSQVREA
>1V6TA 255 XRAY 1.70 0.202 0.227 no Hypothetical UPF0271 protein PH0986 <UNP Y986_PYRHO>
[PYROCOCCUS HORIKOSHII]
MRVDLNSDLGESFGRYKLGLDEEVMKYITSANVACGWHAGDPLVMRKTVRLAKENDVQVG
AHPGYPDLMGFGRRYMKLTPEEARNYILYQVGALYAFAKAEGLELQHVKPHGALYNAMVK
EEDLARAVIEGILDFDKDLILVTLSNSRVADIAEEMGLKVAHEVFADRAYNPDGTLVPRG
RPGAVIEDKEEIAERVISMVKDGGIRAINGEWVDLKVDTICVHGDNPKAVEITSYIRKVL
EEEGVKIVPMKEFIR
>3NOWA 810 XRAY 2.99 0.202 0.235 no UNC-45 protein, SD10334p <UNP Q960B1_DROME> [DROSOPHILA
MELANOGASTER]
NAKTSTKVKQMMDLTFDLATPIDKRRAAANNLVVLAKEQTGAELLYKDHCIAKVASLTKV
EKDQDIYVNMVHLVAALCENSVERTKGVLTELGVPWFMRVLDQKHENCVSTAQFCLQTIL
NALSGLKNKPDSKPDKELCTRNNREIDTLLTCLVYSITDRTISGAARDGVIELITRNVHY
TALEWAERLVEIRGLCRLLDVCSELEDYKYESAMDITGSSSTIASVCLARIYENMYYDEA
KARFTDQIDEYIKDKLLAPDMESKVRVTVAITALLNGPLDVGNQVVAREGILQMILAMAT
TDDELQQRVACECLIAASSKKDKAKALCEQGVDILKRLYHSKNDGIRVRALVGLCKLGSY
GGQDAAIRPFGDGAALKLAEACRRFLIKPGKDKDIRRWAADGLAYLTLDAECKEKLIEDK
ASIHALMDLARGGNQSCLYGVVTTFVNLCNAYEKQEMLPEMIELAKFAKQHIPEEHELDD
VDFINKRITVLANEGITTALCALAKTESHNSQELIARVLNAVCGLKELRGKVVQEGGVKA
LLRMALEGTEKGKRHATQALARIGITINPEVSFSGQRSLDVIRPLLNLLQQDCTALENFE
SLMALTNLASMNESVRQRIIKEQGVSKIEYYLMEDHLYLTRAAAQCLCNLVMSEDVIKMF
EGNNDRVKFLALLCEDEDEETATACAGALAIITSVSVKCCEKILAIASWLDILHTLIANP
SPAVQHRGIVIILNMINAGEEIAKKLFETDIMELLSGLGQLPDDTRAKAREVATQCLAAA
ERYRIIERSDNAEIPDVFAENSKISEIIDD
>2P38A 166 XRAY 1.80 0.210 0.257 no Protein involved in ribosomal biogenesis <UNP
Q9V219_PYRAB> [PYROCOCCUS ABYSSI]
MSGELRVRRASSWELDLILKEAEKYGELLHEFFCVVEGKYRDVYAVNEEVWKIIEDINMR
PYSLGTFVGTIRVDENLVEKFYPNLEFFSLIKLEKNYVILGPKASFLFTTGKDAPKEAVR
EIKWQGSKRVVVLNDLGDIIGIGLINPKSDRRFIKNLKDVGEFLRR
>4BWZA 394 XRAY 2.98 0.224 0.248 no NA(+)/H(+) ANTIPORTER <UNP Q72IM4_THET2> [THERMUS
THERMOPHILUS]
MHGAEHLLEIFYLLLAAQVCAFIFKRLNQPVVIGEVLAGVLVGPALLGLVHEGEILEFLA
ELGAVFLLFMVGLETRLKDILAVGKEAFLVAVLGVALPFLGGYLYGLEIGFETLPALFLG
TALVATSVGITARVLQELGVLSRPYSRIILGAAVIDDVLGLIVLACVNGVAETGQVEVGA
ITRLIVLSVVFVGLAVFLSTLIARLPLERLPVGSPLGFALALGVGMAALAASIGLAPIVG
AFLGGMLLSEVREKYRLEEPIFAIESFLAPIFFAMVGVRLELSALASPVVLVAGTVVTVI
AILGKVLGGFLGALTQGVRSALTVGCGMAPRGEVGLIVAALGLKAGAVNEEEYAIVLFMV
VFTTLFAPFALKPLIAWTERERAAKEGSENLYFQ
>4HTYA 359 XRAY 2.00 0.155 0.169 no Cellulase <UNP I6PLH5_9BACT> [UNCULTURED BACTERIUM]
MGSSHHHHHHSSGLVPRGSHMDNAWETTSGWWNASDIPAFDKSKITRQLPLIKVEGNRFV
DEQGKTIVFRGVNISDPDKIDKDKRFSKKHFEVIRSWGANVVRVPVHPRAWKERGVKGYL
ELLDQVVAWNNELGIYTILDWHSIGNLKSEMFQNNSYHTTKGETFDFWRRVSERYNGINS
VAFYEIFNEPTVFNGRLGIATWAEWKAINEEAITIIQAHNPKAIALVAGFNWAYDLKEAA

ANPIDRQNIAYVSHPYPQKVGAPYQANWERDFGFMADKYPVFATEIGYQRATDKGAHIPV
IDDGSYGPRITDYFNSKGISWVAWVFDPDWSPQLFTDYQTYTPTMQGEHFRKVMLQDNK
>1FLOA 422 XRAY 2.65 0.254 0.297 no FLP RECOMBINASE <UNP FLP_YEAST> [SACCHAROMYCES CEREVISIAE]
PQFDILCKTPPKVLVRQFVERFERPSGEKIALCAAELTYLCWMITHNGTAIKRATFMSYN
TIISNSLSFDIVNKSLQFKYKTQKATILEASLKKLIPAWEFTIIPYYGQKHQSDITDIVS
SLQLQFESSEEADKGNSHSKKMLKALLSEGESIWEITEKILNSFEYTSRFTKTKTLYQFL
FLATFINCGRFSDIKNVDPKSFKLVQNKYLGVIIQCLVTETKTSVSRHIYFFSARGRIDP
LVYLDEFLRNSEPVLKRVNRTGNSSSNKQEYQLLKDNLVRSYNKALKKNAPYSIFAIKNG
PKSHIGRHLMTSFLSMKGLTELTNVVGNWSDKRASAVARTTYTHQITAIPDHYFALVSRY
YAYDPISKEMIALKDETNPIEEWQHIEQLKGSAEGSIRYPAWNGIISQEVLDYLSSYINR
RI
>3QRLA 140 XRAY 1.70 0.190 0.235 no Transcription initiation factor TFIID subunit 14 <UNP TAF14_YEAST> [SACCHAROMYCES CEREVISIAE]
GSHMVATVKRTIRIKTQQHILPEVPPVENFPVRQWSIEIVLLDDEGKEIPATIFDKVIYH
LHPTFANPNRTFTDPPFRIEEQGWGGFPLDISVFLLEKAGERKIPHDLNFLQESYEVEHV
IQIPLNKPLLTEELAKSGST
>4IABA 142 XRAY 1.66 0.164 0.184 no hypothetical protein <UNP A7V626_BACUN> [BACTEROIDES UNIFORMIS]
GQESAEFRPAELAGIWQLCHYVSEIPDVPGILKPSNTFKVLSDDGRIVNFTMIPGKDAII
TGYGTYQQLTDNSYKESIEKNIHLPMLDHKDNILEFEIGDDGVMYLKYFIAKDLNGNELN
TWFHETWKRVGMPAKFPEDLVR
>3B79A 129 XRAY 1.37 0.158 0.182 no Toxin secretion ATP-binding protein <UNP Q87FE3_VIBPA> [VIBRIO PARAHAEMOLYTICUS RIMD 2210633]
SNAMKDPLLNSLIYVSRYYGLANSPEALVNGLPLSDGKLTPFLLPRAAERAGLVAKENRA
ELEKISSLILPAILVLKGGDSCVLNSINMETREAEVTTLESGMVPISIPLEDLLEQYTGR
YFLVKKQFR
>1LFWA 470 XRAY 1.80 0.174 NA no pepV <UNP PEPV_LACDL> [LACTOBACILLUS DELBRUECKII]
MDLNFKELAEAKKDAILKDLEELIAIDSSEDLENATEEYPVGKGPVDAMTKFLSFAKRDG
FDTENFANYAGRVNFGAGDKRLGIIGHMDVVPAGEGWTRDPFKMEIDEEGRIYGRGSADD
KGPSLTAYYGMLLLKEAGFKPKKKIDFVLGTNEETNWVGIDYYLKHEPTPDIVFSPDAEY
PIINGEQGIFTLEFSFKNDDTKGDYVLDKFKAGIATNVTPQVTRATISGPDLEAVKLAYE
SFLADKELDGSFEINDESADIVLIGQGAHASAPQVGKNSATFLALFLDQYAFAGRDKNFL
HFLAEVEHEDFYGKKLGIFHHDDLMGDLASSPSMFDYEHAGKASLLNNVRYPQGTDPDTM
IKQVLDKFSGILDVTYNGFEEPHYVPGSDPMVQTLLKVYEKQTGKPGHEVVIGGGTYGRL
FERGVAFGAQPENGPMVMHAANEFMMLDDLILSIAIYAEAIYELTKDEEL
>3GWCA 258 XRAY 1.90 0.189 0.229 no Thymidylate synthase thyX <UNP THYX_MYCTU> [MYCOBACTERIUM TUBERCULOSIS]
MAETAPLRVQLIAKTDFLAPPDVPWTTDADGGPALVEFAGRACYQSWSKPNPKTATNAGY
LRHIIDVGHFSVLEHASVSFYITGISRSCTHELIRHRHFSYSQLSQRYVPEKDSRVVVPP
GMEDDADLRHILTEAADAARATYSELLAKLEAKFADQPNAILRRKQARQAARAVLPNATE
TRIVVTGNYRAWRHFIAMRASEHADVEIRRLAIECLRQLAAVAPAVFADFEVTTLADGTE
VATSPLATEALEHHHHHH
>1AOCA 175 XRAY 2.00 0.191 0.282 no COAGULOGEN <UNP COAG_TACTR> [TACHYPLEUS TRIDENTATUS]
ADTNAPICLCDEPGVLGRTQIVTTEIKDKIEKAVEAVAQESGVSGRGFSIFSHHPVFREC
GKYECRTVRPEHSRCYNFPPFTHFKSECPVSTRDCEPVFGYTVAGEFRVIVQAPRAGFRQ

CVWQHKCRFGSNSCGYNGRCTQQRSVVRLVTYNLEKDGFLCESFRTCCGCPCRSF
>2ZFYA 234 XRAY 1.69 0.195 0.239 no Ubiquitin thioesterase OTUB1 <UNP OTUB1_HUMAN> [HOMO SAPIENS]
GSEIAVQNPLVSERLELSVLYKEYAEDDNIYQQKIKDLHKKYSYIRKTRPDGNCFYRAFG
FSHLEALLDDSKELQRFKAVSAKSKEDLVSQGFTEFTIEDFHNTFMDLIEQVEKQTSVAD
LLASFNDQSTSDYLVVYLRLLTSGYLQRESKFFEHFIEGGRTVKEFCQQEVEPMCKESDH
IHIIALAQALSVSIQVEYMDRGEGGTTNPHIFPEGSEPKVYLLYRPGHYDILYK
>1VKMA 297 XRAY 1.90 0.156 0.200 no conserved hypothetical protein TM1464 <UNP Q9X1H5_THEMA> [THERMOTOGA MARITIMA]
MGSDKIHHHHHHVIIESRIEKGKPVVGMETTVFVHGLPRKEAIELFRRAKEISREKGFQL
AVIGILKGKIVAGMSEEELEAMMREGADKVGTREIPIVVAEGKNAATTVSATIFLSRRIG
IEVVVTGGTGGVHPGRVDVSQDLTEMSSSRAVLVSSGIKSILDVEATFEMLETLEIPLVG
FRTNEFPLFFSRKSGRRVPRIENVEEVLKIYESMKEMELEKTLMVLNPVPEEYEIPHDEI
ERLLEKIELEVEGKEVTPFLLKKLVEMTNGRTLKANLALLEENVKLAGEIAVKLKRS
>2DBSA 90 XRAY 2.10 0.256 0.271 no hypothetical protein TTHC002 <UNP Q5SGN2_THET8> [THERMUS THERMOPHILUS]
MVNPAERLAELDGVLMQYLLEADLLRELPPTYRLVLLPLDEPEVAAQALAWAMEAPNPEG
WPSVYALFLQGRPIRLLLLGKEVEVAPRAA
>2OCGA 254 XRAY 1.75 0.182 0.201 no Valacyclovir hydrolase <UNP BPHL_HUMAN> [HOMO SAPIENS]
SVTSAKVAVNGVQLHYQQTGEGDHAVLLLPGMLGSGETDFGPQLKNLNKKLFTVVAWDPR
GYGHSRPPDRDFPADFFERDAKDAVDLMKALKFKKVSLLGWSDGGITALIAAAKYPSYIH
KMVIWGANAYVTDEDSMIYEGIRDVSKWSERTRKPLEALYGYDYFARTCEKWVDGIRQFK
HLPDGNICRHLLPRVQCPALIVHGEKDPLVPRFHADFIHKHVKGSRLHLMPEGKHNLHLR
FADEFNKLAEDFLQ
>2ODVA 235 XRAY 2.05 0.212 0.256 no Plectin 1 <UNP Q6S383_HUMAN> [HOMO SAPIENS]
GSHMRANELQLRWQEYRELVLLLLQWMRHHTAAFEERRFPSSFEEIEILWSQFLKFKEME
LPAKEADKNRSKGIYQSLEGAVQAGQLKVPPGYHPLDVEKEWGKLHVAILEREKQLRSEF
ERLEALQRIVTKLQMEAGLAEEQLNQADALLQSDVRLLAAGKVPQRAGEVERDLDKADSM
IRLLFNDVQTLKDGRHPQGEQMYRRVYRLHKRLVAIRTEYNLRLKAGVAAPATQV
>4HI8B 72 XRAY 1.20 0.145 0.168 no LIM and senescent cell antigen-like-containing domain protein 1 <UNP LIMS1_HUMAN> [HOMO SAPIENS]
SENLYFQGSASATCERCKGGFAPAEKIVNSNGELYHEQCFVCAQCFQQFPEGLFYEFEGR
KYCEHDFQMLFA
>3MT0A 290 XRAY 1.58 0.178 0.204 no uncharacterized protein PA1789 <UNP Q9I2V3_PSEAE> [PSEUDOMONAS AERUGINOSA]
SNAMQAIRSILVVIEPDQLEGLALKRAQLIAGVTQSHLHLLVCEKRRDHSAALNDLAQEL
REEGYSVSTNQAWKDSLHQTIIAEQQAEGCGLIIKQHFPDNPLKKAILTPDDWKLLRFAP
CPVLMTKTARPWTGGKILAAVDVGNNDGEHRSLHAGIISHAYDIAGLAKATLHVISAHPS
PMLSSADPTFQLSETIEARYREACRTFQAEYGFSDEQLHIEEGPADVLIPRTAQKLDAVV
TVIGTVARTGLSGALIGNTAEVVLDTLESDVLVLKPDDIIAHLEELASKE
>3RF3A 258 XRAY 1.61 0.184 0.195 no Vinculin <UNP VINC_HUMAN> [HOMO SAPIENS]
MPVFHTRTIESILEPVAQQISHLVIMHEEGEVDGKAIPDLTAPVAAVQAAVSNLVRVGKE
TVQTTEDQILKRDMPPAFIKVENACTKLVQAAQMLQSDPYSVPARDYLIDGSRGILSGTS
DLLLTFDEAEVRKIIRVCKGILEYLTVAEVVETMEDLVTYTKNLGPGMTKMAKMIDERQQ
ELTHQEHRVMLVNSMNTVKELLPVLISAMKIFVTTKNSKNQGIEEALKNRNFTVEKMSAE

INEIIRVLQLTSWDEDAW
>3NYMA 128 XRAY 1.90 0.174 0.208 no uncharacterized protein <UNP Q9JS17_NEIMB> [NEISSERIA MENINGITIDIS SEROGROUP B]
SNAMETLNDIKKILINVGLYQGFDLTDPKVSEEVNHETANMKWIKDYTSDGNWDNEFKED
LKNFLDYMEVCQLALNDKNFKIASNSLFMAMIYAGNLSLIFDSIKTDISTLLSAEYKKNS
FSWPSLDE
>3JU8A 490 XRAY 1.82 0.160 0.196 no Succinylglutamic semialdehyde dehydrogenase <UNP ASTD_PSEAE> [PSEUDOMONAS AERUGINOSA]
SNAMMSTHYIAGQWLAGQGETLESLDPVGQGVVWSGRGADATQVDAAVCAAREAFPAWAR
RPLEQRIELLERFAATLKSRADELARVIGEETGKPLWESATEVTSMVNKVAISVQAFRER
TGEKSGPLADATAVLRHKPHGVVAVFGPYNFPGHLPNGHIVPALLAGNCVVFKPSELTPK
VAELTLKAWIQAGLPAGVLNLVQGGRETGVALAAHRGLDGLFFTGSSRTGNLLHSQFGGQ
PQKILALEMGGNNPLVVEEVADLDAAVYTIIQSAFISAGQRCTCARRLLVPQGAWGDALL
ARLVAVSATLRVGRFDEQPAPFMGAVISLSAAEHLLKAQEHLIGKGAQPLLAMTQPIDGA
ALLTPGILDVSAVAERPDEEFFGPLLQVIRYSDFAAAIREANATQYGLAAGLLSDSRERF
EQFLVESRAGIVNWNKQLTGAASSAPFGGIGASGNHRPSAYYAADYCAYPVASLESPSVS
LPATLTPGIS
>2HLYA 207 XRAY 1.60 0.174 0.214 no Hypothetical protein Atu2299 <UNP Q8UD29_AGRT5> [AGROBACTERIUM TUMEFACIENS STR.]
GYFEGMLIKQTDYFRIYRVINSLLISQNADPASASMYFSTFGAFILQQHYKVKAVPKGGL
AAYNLGGTVLLFADHREDGYVTGAGENFHCWVEADGWAIDFMAPAFSEGTDALAVPAKMF
QRPLSAMAASINDLGQSGDFFYRSEPEATARRFADWHKQAMIGDMASVAANWFRKSPKQM
AASLSVTDRDGKARTVPLTGEMLTGAW
>1KWGA 645 XRAY 1.60 0.168 0.184 no BETA-GALACTOSIDASE <UNP O69315_9DEIN> [THERMUS THERMOPHILUS]
MLGVCYYPEHWPKERWKEDARRMREAGLSHVRIGEFAWALLEPEPGRLEWGWLDEAIATL
AAEGLKVVLGTPTATPPKWLVDRYPEILPVDREGRRRRFGGRRHYCFSSPVYREEARRIV
TLLAERYGGLEAVAGFQTDNEYGCHDTVRCYCPRCQEAFRGWLEARYGTIEALNEAWGTA
FWSQRYRSFAEVELPHLTVAEPNPSHLLDYYRFASDQVRAFNRLQVEILRAHAPGKFVTH
NFMGFFTDLDAFALAQDLDFASWDSYPLGFTDLMPLPPEEKLRYARTGHPDVAAFHHDLY
RGVGRGRFWVMEQQPGPVNWAPHNPSPAPGMVRLWTWEALAHGAEVVSYFRWRQAPFAQE
QMHAGLHRPDSAPDQGFFEAKRVAEELAALALPPVAQAPVALVFDYEAAWIYEVQPQGAE
WSYLGLVYLFYSALRRLGLDVDVVPPGASLRGYAFAVVPSLPIVREEALEAFREAEGPVL
FGPRSGSKTETFQIPKELPPGPLQALLPLKVVRVESLPPGLLEVAEGALGRFPLGLWREW
VEAPLKPLLTFQDGKGALYREGRYLYLAAWPSPELAGRLLSALAAEAGLKVLSLPEGLRL
RRRGTWVFAFNYGPEAVEAPASEGARFLLGSRRVGPYDLAVWEEA
>2B4HA 254 XRAY 1.60 0.204 0.225 no Outer capsid protein VP4 <UNP Q91HI9_ROTRH> [RHESUS ROTAVIRUS]
MGSSHHHHHHSSGLVPRGSHMRAQANEDIVVSKTSLWKEMQYNRDITIRFKFASSIVKSG
GLGYKWSEISFKPANYQYTYTRDGEEVTAHTTCSVNGMNDFNFNGGSLPTDFVISRYEVI
KENSYVYVDYWDDSQAFRNMVYVRSLAANLNSVICTGGDYSFALPVGQWPVMTGGAVSLH
SAGVTLSTQFTDFVSLNSLRFRFRLTVEEPSFSITRTRVSRLYGLPAANPNNGKEYYEVA
GRFSLISLVPSNDD
>4G0AA 317 XRAY 2.10 0.175 0.205 no Non-structural protein 2 <UNP NSP2_ROTSR> [SIMIAN 11 ROTAVIRUS]
MAELACFCYPHLENDSYKFIPFNNLAIKAMLTAKVDKKDMDKFYDSIIYGIAPPPQFKKR

YNTNDNSRGMNFETIMFTKVAMLICEALNSLKVTQANVSNVLSRVVSIRHLENLVIRKEN
PQDILFHSKDLLLKSTLIAIGQSKEIETTITAEGGEIVFQNAAFTMWKLTYLEHQLMPIL
DQNFIEYKVTLNEDKPISDVHVKELVAELRWQYNKFAVITHGKGHYRIVKYSSVANHADR
VYATFKSNVKTGVNNDFNLLDQRIIWQNWYAFTSSMKQGNTLDVCKRLLFQKMKPEKNPF
KGLSTDRKMDEVSQVGV
>2XZEA 146 XRAY 1.75 0.193 0.227 no STAM-BINDING PROTEIN <UNP STABP_HUMAN> [HOMO SAPIENS]
MSDHGDVSLPPEDRVRALSQLGSAVEVNEDIPPRRYFRSGVEIIRMASIYSEEGNIEHAF
ILYNKYITLFIEKLPKHRDYKSAVIPEKKDTVKKLKEIAFPKAEELKAELLKRYTKEYTE
YNEEKKKEAEELARNMAIQQELEKEK
>3AJIB 83 XRAY 2.05 0.171 0.229 no Proteasome (Prosome, macropain) 26S subunit, ATPase, 4 <UNP Q6ZWN9_MOUSE> [MUS MUSCULUS]
MDRRQKRLIFSTITSKMNLSEEVDLEDYVARPDKISGADINSICQESGMLAVRENRYIVL
AKDFEKAYKTVIKKDEQEHEFYK
>3L4RA 170 XRAY 1.45 0.158 0.168 no Minor allergen Can f 2 <UNP ALL2_CANFA> [CANIS FAMILIARIS]
MEGNHEEPQGGLEELSGRWHSVALASNKSDLIKPWGHFRVFIHSMSAKDGNLHGDILIPQ
DGQCEKVSLTAFKTATSNKFDLEYWGHNDLYLAEVDPKSYLILYMINQYNDDTSLVAHLM
VRDLSRQQDFLPAFESVCEDIGLHKDQIVVLSDDDRCQGSRDLEHHHHHH
>3PIUA 436 XRAY 1.35 0.149 0.171 no 1-aminocyclopropane-1-carboxylate synthase <UNP 1A1C_MALDO> [MALUS DOMESTICA]
MRMLSRNATFNSHGQDSSYFLGWQEYEKNPYHEVHNTNGIIQMGLAENQLCFDLLESWLA
KNPEAAAFKKNGESIFAELALFQDYHGLPAFKKAMVDFMAEIRGNKVTFDPNHLVLTAGA
TSANETFIFCLADPGEAVLIPTPYYPGFDRDLKWRTGVEIVPIHCTSSNGFQITETALEE
AYQEAEKRNLRVKGVLVTNPSNPLGTTMTRNELYLLLSFVEDKGIHLISDEIYSGTAFSS
PSFISVMEVLKDRNCDENSEVWQRVHVVYSLSKKDLGLPGFRVGAIYSNDDMVVAAATKM
SSFGLVSSQTQHLLSAMLSDKKLTKNYIAENHKRLKQRQKKLVSGLQKSGISCLNGNAGL
FCWVDMRHLLRSNTFEAEMELWKKIVYEVHLNISPGSSCHCTEPGWFRVCFANLPERTLD
LAMQRLKAFVGEYYNV
>2OZ4A 265 XRAY 2.70 0.209 0.253 no Intercellular adhesion molecule 1 <UNP Q5NKV7_HUMAN> [HOMO SAPIENS]
VLPATPPQLVSPRVLEVDTQGTVVCSLDGLFPVSEAQVHLALGDQRLNPTVTYGNDSFSA
KASVSVTAEDEGTQRLTCAVILGNQSQETLQTVTIYSFPAPNVILTKPEVSEGTEVTVKC
EAHPRAKVTLNGVPAQPLGPRAQLLLKATPEDNGRSFSCSATLEVAGQLIHKNQTRELRV
LYGPRLDERDCPGNWTWPENSQQTPMCQAWGNPLPELKCLKDGTFPLPIGESVTVTRDLE
GTYLCRARSTQGEVTREVTVNVLSP
>3MDPA 142 XRAY 1.90 0.183 0.227 no Cyclic nucleotide-binding domain (CNMP-BD) protein <UNP Q39VF8_GEOMG> [GEOBACTER METALLIREDUCENS]
GMISPERLRVYRFFASLTDEQLKDIALISEEKSFPTGSVIFKENSKADNLMLLLEGGVEL
FYSNGGAGSAANSTVCSVVPGAIFGVSSLIKPYHYTSSARATKPVRVVDINGARLREMSE
NNQALGQVLMNNVAAAVLARLH
>1B9LA 120 XRAY 2.90 0.188 0.259 no EPIMERASE <UNP FOLX_ECOLI> [ESCHERICHIA COLI]
MAQPAAIIRIKNLRLRTFIGIKEEEINNRQDIVINVTIHYPADKARTSEDINDALNYRTV
TKNIIQHVENNRFSLLEKLTQDVLDIAREHHWVTYAEVEIDKLHALRYADSVSMTLSWQR
>4HH3C 240 XRAY 1.75 0.171 0.198 no AppA protein <UNP Q53119_RHOSH> [RHODOBACTER SPHAEROIDES]
VEADTFALYALTEAQAGRSGRAKAVARLSDLLSTDPLGRLTEVEELLRAHAPTAADFARL
FEACAERLTRALAEDRISRMQVTLAYSALQMALRRIHHLPDPQKSVGAVLVAGVPGHKPI

LEAALAAEMLRAVGWSTSVVHPESVAALAARLKTSRTSTLVVAPSLLEGTEQEADTLRFV
SALRARTDLPGLSILVGGRLAQLPPSKLKDSGADAGFAHLALLPAALARVASSAHHHHHH
>3I6EA 385 XRAY 1.70 0.236 0.263 no Muconate cycloisomerase I <UNP Q5LM96_SILPO> [RUEGERIA POMEROYI]
MSLGDLEQKIIAMDLWHLALPVVSARDHGIGRVEGSCEIVVLRLVAEGGAEGFGEASPWA
VFTGTPEASYAALDRYLRPLVIGRRVGDRVAIMDEAARAVAHCTEAKAALDSALLDLAGR
ISNLPVWALLGGKCRDTIPLSCSIANPDFDADIALMERLRADGVGLIKLKTGFRDHAFDI
MRLELIARDFPEFRVRVDYNQGLEIDEAVPRVLDVAQFQPDFIEQPVRAHHFELMARLRG
LTDVPLLADESVYGPEDMVRAAHEGICDGVSIKIMKSGGLTRAQTVARIAAAHGLMAYGG
DMFEAGLAHLAGTHMIAATPEITLGCEFYQASYFLNEDILETPFRVEAGQVIVPDGPGLG
ARADPEKLEHYAVRRSGEGHHHHHH
>3TRTA 77 XRAY 2.30 0.234 0.298 no Vimentin <UNP VIME_HUMAN> [HOMO SAPIENS]
GGSKPDCTAAMRDVRQQYESVAAKNLQEAEEWYKSKFADLSEAANRNNDALRQAKQESTE
YRRQVQSLTMEVDALKG

>1GHEA 177 XRAY 1.55 0.209 0.230 no ACETYLTRANSFERASE <UNP TTR_PSESZ> [PSEUDOMONAS SYRINGAE PV. TABACI]
MNHAQLRRVTAESFAHYRHGLAQLLFETVHGGASVGFMADLDMQQAYAWCDGLKADIAAG
SLLLWVVAEDDNVLASAQLSLCQKPNGLNRAEVQKLMVLPSARGRGLGRQLMDEVEQVAV
KHKRGLLHLDTEAGSVAEAFYSALAYTRVGELPGYCATPDGRLHPTAIYFKTLGQPT
>1QFOA 119 XRAY 1.85 0.200 0.234 no SIALOADHESIN <PIR S50065> [MUS MUSCULUS]
TWGVSSPKNVQGLSGSCLLIPCIFSYPADVPVSNGITAIWYYDYSGKRQVVIHSGDPKLV
DKRFRGRAELMGNMDHKVCNLLLKDLKPEDSGTYNFRFEISDSNRWLDVKGTTVTVTTD
>3QVAA 116 XRAY 1.75 0.200 0.223 no Transthyretin-like protein <UNP A6T926_KLEP7> [KLEBSIELLA PNEUMONIAE SUBSP. PNEUMONIAE]
ENLYFQGHMSTLSTHILDISTGTPAEGVTVSLSREGETLANLVTNAQGRIATFSAAPLPA
GRYCLTAETGAWFARAGRESVFTRAQIDFVIGEAAEDHFHLPFLIAPGGWSTYRGS
>2RAAA 204 XRAY 2.12 0.217 0.251 no Pyruvate synthase subunit porC <UNP PORC_THEMA> [THERMOTOGA MARITIMA MSB8] MGSDKIHHHHHHMPVAKKYFEIRWHGRAGQGAKSASQMLAEAALEAGKYVQAFPEYGAER
TGAPMRAFNRIGDEYIRVRSAVENPDVVVVIDETLLSPAIVEGLSEDGILLVNTVKDFEF
VRKKTGFNGKICVVDATDIALQEIKRGIPNTPMLGALVRVTGIVPLEAIEKRIEKMFGKK
FPQEVIDANKRALRRGYEEVKCSE
>1WE3O 100 XRAY 2.80 0.239 0.279 no cpn10(GroES) <UNP CH10_THET2> [THERMUS THERMOPHILUS]
AAEVKTVIKPLGDRVVVKRIEEEPKTKGGIVLPDTAKEKPQKGKVIAVGTGRVLENGQRV
PLEVKEGDIVVFAKYGGTEIEIDGEEYVILSERDLLAVLQ
>3K3VA 100 XRAY 1.80 0.208 0.224 no Protein SMY2 <UNP SMY2_YEAST> [SACCHAROMYCES CEREVISIAE]
GSNGMSQLPAPVSVESSWRYIDTQGQIHGPFTTQMMSQWYIGGYFASTLQISRLGSTPET
LGINDIFITLGELMTKLEKYDTDPFTTFDKLHVQTTSSDS
>3S6MA 167 XRAY 1.65 0.148 0.183 no Peptidyl-prolyl cis-trans isomerase <UNP Q3JQT3_BURP1> [BURKHOLDERIA PSEUDOMALLEI]
GPGSMVELHTNHGVIKLELDEAKAPKTVENFLNYVKKGHYDGTIFHRVINGFMIQGGGFE
PGLKQKPTDAPIANEANNGLKNDTYTIAMARTNDPHSATAQFFINVNDNEFLNHSSPTPQ
GWGYAVFGKVVEGQDIVDKIKAVKTGSKGFHQDVPNDDVVIEKAVVV
>4F8LA 145 XRAY 1.50 0.141 0.182 no pH 6 antigen <UNP PSAA_YERPE> [YERSINIA PESTIS]

MNTFHVDFAPNTGEIFAGKQPGDVTMFTLTMGDTAPHGGWRLIPTGDSKGGYMISADGDY
VGLYSYMMSWVGIDNNWYINDDSPKDIKDHLYVKAGTVLKPTTYKFTGRVEEYVFDNKQS
TVINSKDVSGEVTVKQGLEHHHHHH
>3IUOA 122 XRAY 1.60 0.190 0.221 no ATP-dependent DNA helicase RecQ <UNP Q7MX11_PORGI>
[PORPHYROMONAS GINGIVALIS] ENEIERPEDMRVRTLANKSKMKVSIVQQIDRKVALDDIAVSHGLDFPELLSEVETIVYSG
TRINIDYFINEVMDEDHLEDIFEYFKESTTDSLEEAMQELGKDYSEEEIRLVRIKFLSEM
AN
>1YQSA 349 XRAY 1.05 0.114 0.136 no D-alanyl-D-alanine carboxypeptidase <UNP DAC_STRSR>
[STREPTOMYCES SP.]
ADLPAPDDTGLQAVLHTALSQGAPGAMVRVDDNGTIHQLSEGVADRATGRAITTTDRFRV
GSVTKSFSAVVLLQLVDEGKLDLDASVNTYLPGLLPDDRITVRQVMSHRSGLYDYTNDMF
AQTVPGFESVRNKVFSYQDLITLSLKHGVTNAPGAAYSYSNTNFVVAGMLIEKLTGHSVA
TEYQNRIFTPLNLTDTFYVHPDTVIPGTHANGYLTPDEAGGALVDSTEQTVSWAQSAGAV
ISSTQDLDTFFSALMSGQLMSAAQLAQMQQWTTVNSTQGYGLGLRRRDLSCGISVYGHTG
TVQGYYTYAFASKDGKRSVTALANTSNNVNVLNTMARTLESAFCGKPTT
>1J1IA 296 XRAY 1.86 0.217 0.248 no meta cleavage compound hydrolase <UNP Q84II3_9BURK>
[JANTHINOBACTERIUM]
MLNKAEQISEKSERAYVERFVNAGGVETRYLEAGKGQPVILIHGGGAGAESEGNWRNVIP
ILARHYRVIAMDMLGFGKTAKPDIEYTQDRRIRHLHDFIKAMNFDGKVSIVGNSMGGATG
LGVSVLHSELVNALVLMGSAGLVVEIHEDLRPIINYDFTREGMVHLVKALTNDGFKIDDA
MINSRYTYATDEATRKAYVATMQWIREQGGLFYDPEFIRKVQVPTLVVQGKDDKVVPVET
AYKFLDLIDDSWGYIIPHCGHWAMIEHPEDFANATLSFLSLRVDITPAAAHHHHHH
>2A2FX 325 XRAY 2.50 0.243 0.276 no Exocyst complex component Sec15 <UNP SEC15_DROME>
[DROSOPHILA MELANOGASTER]
MVNILWELLHNMRDHYNEVLLQRWVHVFREILDKEQFLPMVVQNTEEYECIIERFPFHSE
QLENAPFPKKFPFSRMVPEVYHQAKEFMYACMKFAEELTLSPNEVAAMVRKAANLLLTRS
FSGCLSVVFRQPSITLTQLIQIIIDTQYLEKAGPFLDEFVCHMTNTERSVSQTPSAMFHV
ARQDAEKQVGLRICSKIDEFFELSAYDWLLVEPPGIASAFITDMISYLKSTFDSFAFKLP
HIAQAACRRTFEHIAEKIYSIMYDEDVKQISTGALTQINLDLMQCEFFAASEPVPGLKEG
ELSKYFLRNRQLLDLLILEHHHHHH
>2WEKA 341 XRAY 1.90 0.168 0.214 no ZINC-BINDING ALCOHOL DEHYDROGENASE DOMAIN- CONTAINING
PROTEIN 2 <UNP ZADH2_HUMAN> [HOMO SAPIENS]
SMMQKLVVTRLSPNFREAVTLSRDCPVPLPGDGDLLVRNRFVGVNASDINYSAGRYDPSV
KPPFDIGFEGIGEVVALGLSASARYTVGQAVAYMAPGSFAEYTVVPASIATPVPSVKPEY
LTLLVSGTTAYISLKELGGLSEGKKVLVTAAAGGTGQFAMQLSKKAKCHVIGTCSSDEKS
AFLKSLGCDRPINYKTEPVGTVLKQEYPEGVDVVYESVGGAMFDLAVDALATKGRLIVIG
FISGYQTPTGLSPVKAGTLPAKLLKKSASVQGFFLNHYLSKYQAAMSHLLEMCVSGDLVC
EVDLGDLSPEGRFTGLESIFRAVNYMYMGKNTGKIVVELPH
>4GMFA 372 XRAY 1.85 0.198 0.242 no Yersiniabactin biosynthetic protein YbtU <UNP
A1JTG0_YERE8> [YERSINIA ENTEROCOLITICA SUBSP. ENTEROCOLITICA]
MPSASPKQRVLIVGAKFGEMYLNAFMQPPEGLELVGLLAQGSARSRELAHAFGIPLYTSP
EQITGMPDIACIVVRSTVAGGAGTQLARHFLARGVHVIQEHPLHPDDISSLQTLAQEQGC
CYWINTFYPHTRAGRTWLRDAQQLRRCLAKTPPVVHATTSRQLLYSTLDLLLLALGVDTA
AVECDVVGSFSDFHCLRLFWPEGEACLLLQRYLDPDDPDMHSLIMHRLLLGWPEGHLSLE

ASYGPVIWSSSLFVADHQENAHSLYRRPEILRDPPGLTRSAAPLSWRDCCETVGPEGVSW
LLHQLRSHLAGEHPPVACQNVHQIALSRLWQQILRKTGNAEIRRLTPPHHDRLAGFYNDD
DKEALEHHHHHH
>2EDMA 161 XRAY 2.20 0.278 0.278 no 22kDa structural protein VP22 <UNP Q9ICG6_WSSV> [SHRIMP WHITE SPOT SYNDROME VIRUS]
SVVANYDQMMRVPIQRRAKVMSIRGERSYNTPLGKVAMKNGLSDKDMKDVSADLVISTVT
APRTDPAGTGAENSNMTLKILNNTGVDLLINDITVRPTVIAGNIKGNTMSNTYFSSKDIK
SSSSKITLIDVCSKFEDGAAFEATMNIGFTSKNVIDIKDEI
>1H3FA 432 XRAY 2.00 0.234 0.261 no TYROSYL-TRNA SYNTHETASE <UNP SYY_THETH> [THERMUS THERMOPHILUS]
MAGTGHTPEEALALLKRGAEEIVPEEELLAKLKEGRPLTVKLGADPTRPDLHLGHAVVLR
KMRQFQELGHKVVLIIGDFTGMIGDPSGRSKTRPPLTLEETRENAKTYVAQAGKILRQEP
HLFELRYNSEWLEGLTFKEVVRLTSLMTVAQMLEREDFKKRYEAGIPISLHELLYPFAQA
YDSVAIRADVEMGGTDQRFNLLVGREVQRAYGQSPQVCFLMPLLVGLDGREKMSKSLDNY
IGLTEPPEAMFKKLMRVPDPLLPSYFRLLTDLEEEEIEALLKAGPVPAHRVLARLLTAAY
ALPQIPPRIDRAFYESLGYAWEAFGRDKEAGPEEVRRAEARYDEVAKGGIPEEIPEVTIP
ASELKEGRIWVARLFTLAGLTPSNAEARRLIQNRGLRLDGEVLTDPMLQVDLSRPRILQR
GKDRFVRVRLSD
>1YBKA 52 XRAY 1.45 0.211 0.226 no tetrabrachion <UNP Q54436_STAMA> [STAPHYLOTHERMUS MARINUS]
GSIINETADDIVYRLTVIIDDRYESLKNLITLRADRLEMIINDNVSTILASI
>2X4IA 114 XRAY 2.20 0.243 0.277 no UNCHARACTERIZED PROTEIN 114 <UNP Y114_SIRV1> [SULFOLOBUS ISLANDICUS RUDIVIRUS 1 VARIANT XX]
MNKVYLANAFSINMLTKFPTKVVIDKIDRLEFCENIDNEDIINSIGHDSTIQLINSLCGT
TFQKNRVEIKLEKEDKLYVVQISQRLEEGKILTLEEILKLYESGKVQFFEIIVD
>2F5TX 233 XRAY 1.45 0.164 0.195 no archaeal transcriptional regulator TrmB <GB AAG45392> [THERMOCOCCUS LITORALIS]
AIWRSRSFDEAIEMFRESLYSAKNEVIVVTPSEFFETIREDLIKTLERGVTVSLYIDKIP
DLSEFKGKGNFFVRQFYKLNHLIGMTDGKEVVTIQNATFDSIGPPSFKSTYPEIIFSQYS
LIIEIFKESTLEKEIIGNPKDIRFFAMFHAVDFVKNHLKNRNIYAEITGKNLESGRLETL
TGRVVGYTLSLREAVNNIHLETENGVVKVGGMFAVIEDYESTEIKFIMGGSRS
>3FNIA 159 XRAY 2.30 0.212 0.252 no Putative diflavin flavoprotein A 3 <UNP DFA3_ANASP> [NOSTOC SP.]
TKAETSIGVFYVSEYGYSDRLAQAIINGITKTGVGVDVVDLGAAVDLQELRELVGRCTGL
VIGMSPAASAASIQGALSTILGSVNEKQAVGIFETGGGDDEPIDPLLSKFRNLGLTTAFP
AIRIKQTPTENTYKLCEEAGTDLGQWVTRDRLEHHHHHH
>1WC2A 181 XRAY 1.20 0.147 0.162 no ENDOGLUCANASE <UNP GUN_MYTED> [MYTILUS EDULIS]
NQKCSGNPRRYNGKSCASTTNYHDSHKGACGCGPASGDAQFGWNAGSFVAAASQMYFDSG
NKGWCGQHCGQCIKLTTTGGYVPGQGGPVREGLSKTFMITNLCPNIYPNQDWCNQGSQYG
GHNKYGYELHLDLENGRSQVTGMGWNNPETTWEVVNCDSEHNHDHRTPSNSMYGQCQCAH
Q
>3L0FA 162 XRAY 1.35 0.136 0.175 no C-phycocyanin alpha chain <UNP PHCA_THEEB> [THERMOSYNECHOCOCCUS ELONGATUS]
MKTPITEAIAAADTQGRFLSNTELQAVDGRFKRAVASMEAARALTNNAQSLIDGAAQAVY
QKFPYTTTMQGSQYASTPEGKAKCARDIGYYLRMVTYCLVAGGTGPMDEYLIAGLSEINS

TFDLSPSWYIEALKYIKANHGLTGQAAVEANAYIDYAINALS
>4KQPA 232 XRAY 0.95 0.115 0.120 no Glutamine ABC transporter permease and substrate binding protein protein <UNP Q9CES5_LACLA> [LACTOCOCCUS LACTIS SUBSP. LACTIS]
GMATPKKDVYTIASDNSFAPFEFQNDDKQFTGIDVDLLNAIAKNQGFKLKWNFIGFQAAV
DSVQSGHADGMMSGMSITDARKQVFDYGSPYYSSNLTIATSSTDDSIKSWKDLKGKTLGA
KNGTASFDYLNAHAKEYGYTVKTFTDATTMYSSLNNGSINALMDDEPVIKYAIKQGQKFA
TPIKPIPDGQYGFAVKKGSNPELIEMFNNGLANLRANGEYDKIIDKYLESDA
>2EB1A 200 XRAY 2.00 0.212 0.234 no Endoribonuclease Dicer <UNP DICER_HUMAN> [HOMO SAPIENS]
MNHLISGFENFEKKINYRFKNKAYLLQAFTHASYHYNTITDCYQRLEFLGDAILDYLITK
HLYEDPRQHSPGVLTDLRSALVNNTIFASLAVKYDYHKYFKAVSPELFHVIDDFVQFQLE
KNEMQGMDSELRRSEEDEEKEEDIEVPKAMGDIFESLAGAIYMDSGMSLETVWQVYYPMM
RPLIEKFSANVPRSHHHHHH
>2JHPA 644 XRAY 2.50 0.229 0.289 no VP4 CORE PROTEIN <UNP VP4_BTV10> [BLUETONGUE VIRUS 10 (ISOLATE USA)]
MPEPHAVLYVTNELSHIVKDGFLPIWKLTGDESLNDLWLENGKYATDVYAYGDVSKWTIR
QLRGHGFIFISTHKNVQLADIIKTVDVRIPREVARSHDMKAFENEIGRRRIRMRKGFGDA
LRNYAFKMAIEFHGSEAETLNDANPRLHKIYGMPEIPPLYMEYAEIGTRFDDEPTDEKLV
SMLDYIVYSAEEVHYIGCGDLRTLMQFKKRSPGRFRRVLWHVYDPIAPECSDPNVIVHNI
MVDSKKDILKHMNFLKRVERLFIWDVSSDRSQMNDHEWETTRFAEDRLGEEIAYEMGGAF
SSALIKHRIPNSKDEYHCISTYLFPQPGADADMYELRNFMRLRGYSHVDRHMHPDASVTK
VVSRDVRKMVELYHGRDRGRFLKKRLFEHLHIVRKNGLLHESDEPRADLFYLTNRCNMGL
EPSIYEVMKKSVIATAWVGRAPLYDYDDFALPRSTVMLNGSYRDIRILDGNGAILFLMWR
YPDIVKKDLTYDPAWAMNFAVSLKEPIPDPPVPDISLCRFIGLRVESSVLRVRNPTLHET
ADELKRMGLDLSGHLYVTLMSGAYVTDLFWWFKMILDWSAQNREQKLRDLKRSAAEVIEW
KEQMAERPWHVRNDLIAALREYKRKMGMREGASIDSWLELLRHL
>1WURA 220 XRAY 1.82 0.206 0.237 no GTP cyclohydrolase I <UNP Q5SH52_THET8> [THERMUS THERMOPHILUS]
MSPGPQSGGQERGSMERKMVELEDTGLTFATEVDLERLQALAAEWLQVIGEDPGREGLLK
TPERVAKAWAFLTRGYRQRLEEVVGGAVFPAEGSEMVVVKGVEFYSMCEHHLLPFFGKVH
IGYIPDGKILGLSKFARIVDMFARRLQVQERLAVQIAEAIQEVLEPQGVGVVVEGVHLCM
MMRGVEKQHSRTVTSAMLGVFRENQKTREEFLSHLRDGTA
>3TBIA 115 XRAY 3.00 0.253 0.295 no RNA polymerase-associated protein Gp33 <UNP VG33_BPT4> [ENTEROBACTERIA PHAGE T4]
GPHMTQFSLNDIRPVDETGLSEKELSIKKEKDEIAKLLDRQENGFIIEKMVEEFGMSYLE
ATTAFLEENSIPETQFAKFIPSGIIEKIQSEAIDENLLRPSVVRCEKTNTLDFLL
>2RDMA 132 XRAY 1.76 0.189 0.220 no Response regulator receiver protein <UNP A6UIY7_9RHIZ> [SINORHIZOBIUM MEDICAE]
MSLEAVTILLADDEAILLLDFESTLTDAGFLVTAVSSGAKAIEMLKSGAAIDGVVTDIRF
CQPPDGWQVARVAREIDPNMPIVYISGHAALEWASNGVPDSIILEKPFTSAQLITAVSQL
LNAREGHHHHHH
>3M7KA 142 XRAY 1.92 0.173 0.201 no restriction endonuclease PacI <PDB 3M7K> [PSEUDOMONAS ALCALIGENES]
MTQCPRCQRNLAADEFYAGSSKMCKGCMTWQNLSYNANKEGHANTFTKATFLAWYGLSAQ
RHCGYCGISEAGFTSLHRTNPRGYHIQCLGVDRSDSFEGYSPQNARLACFICNRIKSNIF

SASEMDVLGEAISKAWHGRGIA
>3TOWA 152 XRAY 1.34 0.166 0.193 no Multivesicular body subunit 12B <UNP F125B_HUMAN> [HOMO SAPIENS]
MDPITGVGVVASRNRAPTGYDVVAQTADGVDADLWKDGLFKSKVTRYLCFTRSFSKENSH
LGNVLVDMKLIDIKDTLPVGFIPIQETVDTQEVAFRKKRLCIKFIPRDSTEAAICDIRIM
GRTKQAPPQYTFIGELNSMGIWYRMGHHHHHH
>3E7LA 63 XRAY 2.25 .211 0.243 no Transcriptional regulator (NtrC family) <UNP O66551_AQUAE> [AQUIFEX AEOLICUS]
RDLSYLLKIKELKEAKKEFEKIFIEEKLREYDYDLKRTAEEIGIDLSNLYRKIKSLNIRV
KSS
>2XF1A 129 XRAY 1.96 0.189 0.230 no COFILIN ACTIN-DEPOLYMERIZING FACTOR HOMOLOG 1 <UNP CADF1_PLAF7> [PLASMODIUM FALCIPARUM]
GPLGSMISGIRVNDNCVTEFNNMKIRKTCCGWIIFVIQNCCEIIIHSKGASTTLTELVQS
IDKNNEIQCAYVVFDAVSKIHFFMYARESSNSRDRMTYASSKQAILKKIEGVNVLTSVIE
SAQDVADLK
>1T77A 414 XRAY 2.40 0.220 0.258 no Lipopolysaccharide-responsive and beige-like anchor protein <UNP LRBA_HUMAN> [HOMO SAPIENS]
GPVSLSTPAQLVAPSVVVKGTLSVTSSELYFEVDEEDPNFKKIDPKILAYTEGLHGKWLF
TEIRSIFSRRYLLQNTALEIFMANRVAVMFNFPDPATVKKVVNFLPRVGVGTSFGLPQTR
RISLASPRQLFKASNMTQRWQHREISNFEYLMFLNTIAGRSYNDLNQYPVFPWVITNYES
EELDLTLPTNFRDLSKPIGALNPKRAAFFAERYESWEDDQVPKFHYGTHYSTASFVLAWL
LRIEPFTTYFLNLQGGKFDHADRTFSSISRAWRNSQRDTSDIKELIPEFYYLPEMFVNFN
NYNLGVMDDGTVVSDVELPPWAKTSEEFVHINRLALESEFVSCQLHQWIDLIFGYKQQGP
EAVRALNVFYYLTYEGAVNLNSITDPVLREAVEAQIRSFGQTPSQLLIEPHPPR
>1MTYG 162 XRAY 1.70 0.183 NA no METHANE MONOOXYGENASE HYDROXYLASE <UNP MEMG_METCA> [METHYLOCOCCUS CAPSULATUS STR. BATH]
LGIHSNDTRDAWVNKIAHVNTLEKAAEMLKQFRMDHTTPFRNSYELDNDYLWIEAKLEEK
VAVLKARAFNEVDFRHKTAFGEDAKSVLDGTVAKMNAAKDKWEAEKIHIGFRQAYKPPIM
PVNYFLDGERQLGTRLMELRNLNYYDTPLEELRKQRGVRVVH
>1XAWA 140 XRAY 1.45 0.275 0.284 no Occludin <UNP OCLN_HUMAN> [HOMO SAPIENS]
AKGRAGRSKRTEQDHYETDYTTGGESCDELEEDWIREYPPITSDQQRQLYKRNFDTGLQE
YKSLQSVLDEINKELSRLDKELDDYREESEEYMAAADEYNRLKQVKGSADYKSKKNHCKQ
LKSKLSHIKKMVGDYDRQKT
>2YFVC 63 XRAY 2.32 0.222 0.257 no SCM3 <UNP Q6CL77_KLULA> [KLUYVEROMYCES LACTIS NRRL Y-1140]
RDGVVYIMSKENRLIPKLSDEEVMERHKKADENMKRVWSQIIQKYESIDNQGDVIDLQTG
EVI
>3RQ9A 85 XRAY 1.00 0.147 0.176 no Type VI secretion immunity protein <UNP Q9IOD9_PSEAE> [PSEUDOMONAS AERUGINOSA]
MNLKPQTLMVAIQCVAARTRELDAQLQNDDPQNAAELEQLLVGYDLAADDLKNAYEQALG
QYSGLPPYDRLIEEPASLEHHHHHH
>4F67A 265 XRAY 1.79 0.170 0.201 no UPF0176 protein lpg2838 <UNP Y2838_LEGPH> [LEGIONELLA PNEUMOPHILA SUBSP. PNEUMOPHILA]
MGHHHHHHSHMVKDIIIASFYKFIPLNDFRSLREPILTKMHEIGIKGTIILAHEGVNGGF
AGNREQMNVFYDYLRSDSRFADLHFKETYDNKNPFDKAKVKLRKEIVTMGVQKVDPSYNA

GTYLSPEEWHQFIQDPNVILLDTRNDYEYELGTFKNAINPDIENFREFPDYVQRNLIDKK
DKKIAMFCTGGIRCEKTTAYMKELGFEHVYQLHDGILNYLESIPESESLWEGKCFVFDDR
VAVDQKLDRVYPQLPQDYKYEREQK
>3LHLA 287 XRAY 2.30 0.193 0.238 no Putative agmatinase <UNP Q18A84_CLOD6> [CLOSTRIDIUM DIFFICILE]
MSLNYEESNLIVFGVGFDGTTSNRPGARFASSSMRKEFYGLETYSPFLDLDLEDYNICDY
GDLEISVGSTEQVLKEIYQETYKIVRDSKVPFMIGGEHLVTLPAFKAVHEKYNDIYVIHF
DAHTDLREEYNNSKNSHATVIKRIWDIVGDNKIFQFGIRSGTKEEFKFATEEKHTYMEIG
GIDTFENIVNMLNGKNIYLTIDLDVLDASVFPGTGTPEPGGVNYREFQEIFKIIKNSNIN
IVGCDIVELSPDYDTTGVSTVIACKILRELCLIISDKIKEGHHHHHH
>2A1RA 430 XRAY 2.60 0.221 0.236 no Poly(A)-specific ribonuclease PARN <UNP PARN_HUMAN> [HOMO SAPIENS]
MEIIRSNFKSNLHKVYQAIEEADFFAIDGEFSGISDGPSVSALTNGFDTPEERYQKLKKH
SMDFLLFQFGLCTFKYDYTDSKYITKSFNFYVFPKPFNRSSPDVKFVCQSSSIDFLASQG
FDFNKVFRNGIPYLNQEEERQLREQYDEKRSQANGAGALSYVSPNTSKCPVTIPEDQKKF
IDQVVEKIEDLLQSEENKNLDLEPCTGFQRKLIYQTLSWKYPKGIHVETLETEKKERYIV
ISKVDEEERKRREQQKHAKEQEELNDAVGFSRVIHAIANSGKLVIGHNMLLDVMHTVHQF
YCPLPADLSEFKEMTTCVFPRLLDTKLMASTQPFKDIINNTSLAELEKRLKETPFNPPKV
ESAEGFPSYDTASEQLHEAGYDAYITGLCFISMANYLGSFLSPPKIHVSARSKLIEPFFN
KLFLMRVMDI
>2OGYA 262 XRAY 2.30 0.188 0.234 no 5-methyltetrahydrofolate corrinoid/iron sulfur protein methyltransferase <UNP Q46389_MOOTH> [MOORELLA THERMOACETICA]
MLIIGERINGMFGDIKRAIQERDPAPVQEWARRQEEGGARALDLNVGPAVQDKVSAMEWL
VEVTQEVSNLTLCLDSTNIKAIEAGLKKCKNRAMINSTNAEREKVEKLFPLAVEHGAALI
GLTMNKTGIPKDSDTRLAFAMELVAAADEFGLPMEDLYIDPLILPANVAQDHAPEVLKTL
QQIKMLADPAPKTVLGLSAVSQNCQNRPLINRTFLAMAMACGLDAAIADACDEALIETAA
TAEILLNQTVYCDSFVKMFKTR
>1ZHXA 438 XRAY 1.50 0.225 0.233 no KES1 protein <UNP KES1_YEAST> [SACCHAROMYCES CEREVISIAE]
GAMDPSQYASSSSWTSFLKSIASFNGDLSSLSAPPFILSPISLTEFSQYWAEHPELFLEP
SFINDDNYKEHCLIDPEVESPELARMLAVTKWFISTLKSQYCSRNESLGSEKKPLNPFLG
ELFVGKWENKEHPEFGETVLLSEQVSHHPPVTAFSIFNDKNKVKLQGYNQIKASFTKSLM
LTVKQFGHTMLDIKDESYLVTPPPLHIEGILVASPFVELEGKSYIQSSTGLLCVIEFSGR
GYFSGKKNSFKARIYKDSKDSKDKEKALYTISGQWSGSSKIIKANKKEESRLFYDAARIP
AEHLNVKPLEEQHPLESRKAWYDVAGAIKLGDFNLIAKTKTELEETQRELRKEEEAKGIS
WQRRWFKDFDYSVTPEEGALVPEKDDTFLKLASALNLSTKNAPSGTLVGDKEDRKEDLSS
IHWRFQRELWDEEKEIVL
>2XUAA 266 XRAY 1.90 0.185 0.241 no 3-OXOADIPATE ENOL-LACTONASE <UNP Q13KT2_BURXL> [BURKHOLDERIA XENOVORANS] GSHMPYAAVNGTELHYRIDGERHGNAPWIVLSNSLGTDLSMWAPQVAALSKHFRVLRYDT
RGHGHSEAPKGPYTIEQLTGDVLGLMDTLKIARANFCGLSMGGLTGVALAARHADRIERV
ALCNTAARIGSPEVWVPRAVKARTEGMHALADAVLPRWFTADYMEREPVVLAMIRDVFVH
TDKEGYASNCEAIDAADLRPEAPGIKVPALVISGTHDLAATPAQGRELAQAIAGARYVEL
DASHISNIERADAFTKTVVDFLTEQK
>3QWEA 279 XRAY 2.40 0.227 0.231 no GEM-interacting protein <UNP GMIP_HUMAN> [HOMO SAPIENS]
GGEELDLRLIRTKGGVDAALEYAKTWSRYAKELLAWTEKRASYELEFAKSTMKIAEAGKV

SIQQQSHMPLQYIYTLFLEHDLSLGTLAMETVAQQKRDYYQPLAAKRTEIEKWRKEFKEQ
WMKEQKRMNEAVQALRRAQLQYVQRSEDLRARSQGSPEDSAPQASPGPSKQQERRRRSRE
EAQAKAQEAEALYQACVREANARQQDLEIAKQRIVSHVRKLVFQGDEVLRRVTLSLFGLR
GAQAERGPRAFAALAECCAPFEPGQRYQEFVRALRPEAP
>2DXAA 166 XRAY 1.58 0.180 0.215 no Protein ybaK <UNP YBAK_ECOLI> [ESCHERICHIA COLI]
GSSGSSGMTPAVKLLEKNKISFQIHTYEHDPAETNFGDEVVKKLGLNPDQVYKTLLVAVN
GDMKHLAVAVTPVAGQLDLKKVAKALGAKKVEMADPMVAQRSTGYLVGGISPLGQKKRLP
TIIDAPAQEFATIYVSGGKRGLDIELAAGDLAKILDAKFADIARRD
>2W6KA 145 XRAY 1.70 0.202 0.250 no COBE <UNP Q9HZQ0_PSEAE> [PSEUDOMONAS AERUGINOSA]
GSHMPLPIPSLLIAGIGCRRGCSAEHLRALLERTLGEHGRSLAELDALASIDGKRDEPGL
RQLATLLERPVHFLAPAVLHDYEPRLLSPSAVALRETGCSSVAEAAALALAERLGGGRAD
LLGAKRSDDRASIALARLLTERELP
>3EGNA 143 XRAY 2.50 0.199 0.236 no RNA-binding protein 40 <UNP RBM40_HUMAN> [HOMO SAPIENS]
GPLGSDSDEMPSECISRRELEKGRISREEMETLSVFRSYEPGEPNCRIYVKNLAKHVQEK
DLKYIFGRYVDFSSETQRIMFDIRLMKEGRMKGQAFIGLPNEKAAAKALKEANGYVLFGK PMVVQFARSARPKQDPKEGKRKC
>1ITHA 141 XRAY 2.50 0.147 NA no HEMOGLOBIN (CYANO MET) <UNP HBF1_URECA> [URECHIS CAUPO]
GLTAAQIKAIQDHWFLNIKGCLQAAADSIFFKYLTAYPGDLAFFHKFSSVPLYGLRSNPA
YKAQTLTVINYLDKVVDALGGNAGALMKAKVPSHDAMGITPKHFGQLLKLVGGVFQEEFS
ADPTTVAAWGDAAGVLVAAMK
>3UJCA 266 XRAY 1.19 0.146 0.167 no Phosphoethanolamine N-methyltransferase <UNP Q6T755_PLAFA> [PLASMODIUM FALCIPARUM]
MTLIENLNSDKTFLENNQYTDEGVKVYEFIFGENYISSGGLEATKKILSDIELNENSKVL
DIGSGLGGGCMYINEKYGAHTHGIDICSNIVNMANERVSGNNKIIFEANDILTKEFPENN
FDLIYSRDAILALSLENKNKLFQKCYKWLKPTGTLLITDYCATEKENWDDEFKEYVKQRK
YTLITVEEYADILTACNFKNVVSKDLSDYWNQLLEVEHKYLHENKEEFLKLFSEKKFISL
DDGWSRKIKDSKRKMQRWGYFKATKN
>3PKZA 124 XRAY 1.80 0.209 0.246 no Recombinase Sin <UNP D2J612_STAAU> [STAPHYLOCOCCUS AUREUS]
MIIGYARVSSLDQNLERQLENLKTFGAEKIFTEKQSGKSIENRPILQKALNFVEMGDRFI
VESIDRLGRNYNEVIHTVNYLKDKEVQLMITSLPMMNEVIGNPLLDKFMKDLIIRILAMV
SEQE
>3T97B 65 XRAY 2.80 0.231 0.271 no Nuclear pore complex protein Nup54 <UNP NUP54_RAT> [RATTUS NORVEGICUS]
GSHMTKQHQTRLDIISEDISELQKNQTTTMAKIAQYKRKLMDLSHRTLQVLIKQEIQRKS
GYAIQ
>1L6PA 125 XRAY 1.65 0.141 0.226 no Thiol:disulfide interchange protein dsbD <UNP DSBD_ECOLI> [ESCHERICHIA COLI] GLFDAPGRSQFVPADQAFAFDFQQNQHDLNLTWQIKDGYYLYRKQIRITPEHAKIADVQL
PQGVWHEDEFYGKSEIYRDRLTLPVTINQASAGATLTVTYQGCADAGFCYPPETKTVPLS
EVVAN
>3EZQB 122 XRAY 2.73 0.236 0.278 no Protein FADD <UNP FADD_HUMAN> [HOMO SAPIENS]
GEEDLCAAFNVICDNVGKDWRRLARQLKVSDTKIDSIEDRYPRNLTERVRESLRIWKNTE
KENATVAHLVGALRSCQMNLVADLVQEVQQARDLQNRSGAMSPMSWNSDASTSEASHHHH
HH
>4EDPA 351 XRAY 1.85 0.164 0.191 no ABC transporter, substrate-binding protein <UNP

Q0TR20_CLOP1> [CLOSTRIDIUM PERFRINGENS]
SNAMKKKILATLLTGLVLGTSLVGCGKTEGAEAGKKLVVSTWGLNEDVLKETVFEPFAKE
HGVEIVLDIGNNSERLTKMKNNPNSQIDITYLAESFAEQGVEAGIFDKLDYSKIPNASEM
NEKAKSTVEAGYGPAYTLNSIGIVVDPSAGIEINSWEDLWKPELKNKIAIPDITTTNGPA
MVEIAAEKAGVDVKTDNGEAAFKELEALKPNVVKTYSKSSDLANMFSNGEIVAAVASDFA
FGTISKAKPEVINVIPESGTYLNFNTININKNSKNKDLAYEFINYALSKEVQEKTAKALN
ESPVNKEVKLSEEETKNLTYGPVVDNAKVIDFKFVNSVMDQWVNNWNRIMN
>3RJ2X 138 XRAY 2.20 0.201 0.275 no Putative uncharacterized protein <UNP Q5YFA7_9VIRU>
[SINGAPORE GROUPER IRIDOVIRUS]
MGWAIVANCEFVNATGKKTTILVNENWAKYCWIWTYKFPEKYTLLRYSVDGEMFMRHRVT
FFNATGRYITHTHLNHGLEDVLEGSLAVPKDAAYARIHAAINVSLTNPGDVHMHYDETEG
EQIRSYDAAEFARTLAAV
>2Q66A 525 XRAY 1.80 0.191 0.225 no Poly(A) polymerase <UNP PAP_YEAST> [SACCHAROMYCES
CEREVISIAE]
KVFGITGPVSTVGATAAENKLNDSLIQELKKEGSFETEQETANRVQVLKILQELAQRFVY
EVSKKKNMSDGMARDAGGKIFTYGSYRLGVHGPGSDIDTLVVVPKHVTREDFFTVFDSLL
RERKELDEIAPVPDAFVPIIKIKFSGISIALICARLDQPQVPLSLTLSDKNLLRNLDEKD
LRALNGTRVTDEILELVPKPNVFRIALRAIKLWAQRRAVYANIFGFPGGVAWAMLVARIC
QLYPNACSAVILNRFFIILSEWNWPQPVILKPIEDGPLQVRVWNPKIYAQDRSHRMPVIT
PAYPSMCATHNITESTKKVILQEFVRGVQITNDIFSNKKSWANLFEKNDFFFRYKFYLEI
TAYTRGSDEQHLKWSGLVESKVRLLVMKLEVLAGIKIAHPFTKPFESSYCCPTEDDYEMI
QDKYGSHKTETALNALKLVTDENKEEESIKDAPKAYLSTMYIGLDFNIENKKEKVDIHIP
CTEFVNLCRSFNEDYGDHKVFNLALRFVKGYDLPDEVFDENEKRP
>3O3XA 198 XRAY 1.45 0.152 0.181 no gp41-5 <PDB 3O3X> [ARTIFICIAL GENE]
SGIVQQQNNLLRAIEAQQHLLQLTVWGIKQLQARILSGGSGGWMEWDREINNYTSLIHSL
IEESQNQQEKNEQELLGGSGGSGIVQQQNNLLRAIEAQQHLLQLTVWGIKQLQARILSGG
SGGWMEWDREINNYTSLIHSLIEESQNQQEKNEQELLGGSGGSGIVQQQNNLLRAIEAQQ
HLLQLTVWGIKQLQARIL
>3KWRA 97 XRAY 1.45 0.164 0.181 no Putative RNA-binding protein <UNP Q88W58_LACPL>
[LACTOBACILLUS PLANTARUM]
GMNHNYVEVKYPAIFRDEGTYWDVRFPDVPAAQTFGASVQVAADNAANALAIALFEQSLP
PASDPQYWRLASTEFVVWITMADVQFGPGADTPEPMN
>3DSBA 157 XRAY 1.48 0.170 0.205 no Putative acetyltransferase <UNP Q185U9_CLOD6>
[CLOSTRIDIUM DIFFICILE]
SNAEELIEIREARMDDLDTIAKFNYNLAKETEGKELDMDVLTKGVKALLLDERKGKYHVY
TVFDKVVAQIMYTYEWSDWRNGNFLWIQSVYVDKEYRRKGIFNYLFNYIKNICDKDENIV
GMRLYVEKENINAKATYESLNMYECDYNMYEYEVIHS
>1Z72A 225 XRAY 1.45 0.152 0.167 no transcriptional regulator, putative <UNP Q97RS7_STRPN>
[STREPTOCOCCUS PNEUMONIAE] SNAMETQDYAFQPGLTVGELLKSSQKDWQAAINHRFVKELFAGTIENKVLKDYLIQDYHF
FDAFLSMLGACVAHADKLESKLRFAKQLGFLEADEDGYFQKAFKELKVAENDYLEVTLHP
VTKAFQDLMYSAVASSDYAHLLVMLVIAEGLYLDWGSKDLALPEVYIHSEWINLHRGPFF
AEWVQFLVDELNRVGKNREDLTELQQRWNQAVALELAFFDIGYDV
>2W79A 241 XRAY 1.85 0.206 0.267 no 1-(5-PHOSPHORIBOSYL)-5-[(5-PHOSPHORIBOSYLAMINO)
METHYLIDENEAMINO] IMIDAZOLE-4-CARBOXAMIDE ISOMERASE <UNP HIS4_THEMA> [THERMOTOGA MARITIMA]

MLVVPAIDLFRGKVARMIKGRKENTIFYEKDPVELVEKLIEEGFTLIHVVDLSNAIENSG
ENLPVLEKLSEFAEYIQIGGGIRSLDYAEKLRKLGYRRQIVSSKVLEDPSSLKSLREIDV
EPVFSLVTRGGRVAFKGWLAEEEIDPVSLLKRLKEYGLEEIVHTEIEKVGTLQEHDFSLT
KKIAIEAEVKVLAAGGISSENSLKTAQKVHTETNGLLKGVIVGRAFLEGILTVEVMKRYA
R
>4EGTA 357 XRAY 2.00 0.201 0.232 no Major capsid protein VP60 <UNP Q3HNQ1_RHDV> [RABBIT HEMORRHAGIC DISEASE VIRUS]
GPLGSAPSSKTVDSISPAGLLTTPVLTGVGNDNRWNGQIVRLQPVPGGFSTCNRHWNLNG
STYGWSSPRFADIDHRRGSASYSGNSSTNVLQFWYANAGSAIDNPISQVAPDGFPDMSFV
PFNSPNIPTAGWVGFGGIWNSNNGAPAATTVQAYELGFATGAPNNLQPTTNTSGAQTVAK
SIYAVVTGTNQNPTGLFVMASGVISTPNASAVTYTPQPDRIVTTPGTPAAAPVGKNTPIM
FASVVRRTGDVNAAAGSTNGTQYGTGSQPLPVTIGLSLNNYSSALMPGQFFVWQLTFASG
FMEIGLSVDGYFYAGTEASTTLIDLTELIDVRPVGPRPSKSTLVFNLGGTTNGFSYV
>1UZKA 162 XRAY 1.35 0.202 0.261 no FIBRILLIN-1 <UNP FBN1_HUMAN> [HOMO SAPIENS]
TDVNECLDPTTCISGNCVNTPGSYICDCPPDFELNPTRVGCVDTRSGNCYLDIRPRGDNG
DTACSNEIGVGVSKASCCCSLGKAWGTPCEMCPAVNTSEYKILCPGGEGFRPNPITVILE
DIDECQELPGLCQGGKCINTFGSFQCRCPTGYYLNEDTRVCD
>3HHFA 213 XRAY 2.30 0.204 0.258 no Transcriptional regulator, LysR family <UNP Q9JXW7_NEIMB> [NEISSERIA MENINGITIDIS SEROGROUP B]
GPEIPQGVLSVDSAMPMVLHLLAPLAAKFNERYPHIRLSLVSSEGYINLIERKVDIALRA
GELDDSGLRARHLFDSRFRVIASPEYLAKHGTPQSTEELAGHQCLGFTEPGSLNTWAVLD
AQGNPYKISPHFTASSGEILRSLCLSGCGIVCLSDFLVDNDIAEGKLIPLLAEQTSDKTH
PFNAVYYSDKAVNLRLRVFLDFLVEELGNNLCG
>2VK2A 306 XRAY 1.20 0.179 0.190 no ABC TRANSPORTER PERIPLASMIC-BINDING PROTEIN YTFQ <PDB 2VK2> [ESCHERICHIA COLI]
APLTVGFSQVGSESGWRAAETNVAKSEAEKRGITLKIADGQQKQENQIKAVRSFVAQGVD
AIFIAPVVATGWEPVLKEAKDAEIPVFLLDRSIDVKDKSLYMTTVTADNILEGKLIGDWL
VKEVNGKPCNVVELQGTVGASVAIDRKKGFAEAIKNAPNIKIIRSQSGDFTRSKGKEVME
SFIKAENNGKNICMVYAHNDDMVIGAIQAIKEAGLKPGKDILTGSIDGVPDIYKAMMDGE
ANASVELTPNMAGPAFDALEKYKKDGTMPEKLTLTKSTLYLPDTAKEELEKKKNMGYLEH
HHHHHH
>1Y6ZA 263 XRAY 1.88 0.208 0.268 no heat shock protein, putative <UNP Q8IL32_PLAF7> [PLASMODIUM FALCIPARUM]
GHQLPIWKQDEKSLTENDYYSFYKNTFKAYDDPLAYVHFNVEGQISFNSILYIPGSLPWE
LSKNMFDEESRGIRLYVKRVFINDKFSESIPRWLTFLRGIVDSENLPLNVGREILQKSKM
LSIINKRIVLKSISMMKGLKETGGDKWTKFLNTFGKYLKIGVVEDKENQEEIASLVEFYS
INSGDKKTDLDSYIENMKEDQKCIYYISGENKKTAQNSPSLEKLKALNYDVLFSLEPIDE FCLSSLTVNKYKGYEVLDVNKAD
>2YNMD 530 XRAY 2.10 0.201 0.233 no LIGHT-INDEPENDENT PROTOCHLOROPHYLLIDE REDUCTASE SUBUNIT B <UNP CHLB_PROMA> [PROCHLOROCOCCUS MARINUS]
MELTLWTYEGPPHIGAMRIATSMKGLHYVLHAPQGDTYADLLFTMIERRGSRPPVTYTTF
QARDLGGDTAELVKGHIFEAVERFKPEALLVGESCTAELIQDQPGSLAKGMGLNIPIVSL
ELPAYSKKENWGASETFYQLIRGLLKEISEDSSNNAKQSWQEEGRRPRVNLLGPSLLGFR
CRDDVLEIQKILGENGIDINVIAPLGASPSDLMRLPKADANVCLYPEIAESTCLWLERNF
KTPFTKVVPIGVKATQDFLEELYELLGMEVSNSISNSDQSKLPWYSKSVDSNYLTGKRVF

IFGDGTHVLAAARIANEELGFEVVGIGTYSREMARKVRAAATELGLEALITNDYLEVEES
IKECAPELVLGTQMERHSAKRLGIPCAVISTPMHVQDVPARYSPQMGWEGANVIFDDWVH
PLMMGLEEHLIGMFRHDFEFTDGHQSHLGHLGGHASETKTSSKGINQSPNNHSPAGESIH
WTSEGESELAKIPFFVRGKVRRNTEKYARQAGCREIDGETLLDAKAHFGA
>3S84A 273 XRAY 2.40 0.202 0.229 no Apolipoprotein A-IV <UNP APOA4_HUMAN> [HOMO SAPIENS]
GATELHERLAKDSEKLKEEIGKELEELRARLLPHANEVSQKIGDNLRELQQRLEPYADQL
RTQVNTQAEQLRRQLTPYAQRMERVLRENADSLQASLRPHADELKAKIDQNVEELKGRLT
PYADEFKVKIDQTVEELRRSLAPYAQDTQEKLNHQLEGLTFQMKKNAEELKARISASAEE
LRQRLAPLAEDVRGNLRGNTEGLQKSLAELGGHLDQQVEEFRRRVEPYGENFNKALVQQM
EQLRQKLGPHAGDVEGHLSFLEKDLRDKVNSFF
>3FIJA 254 XRAY 2.30 0.240 0.269 no Lin1909 protein <UNP Q92AL3_LISIN> [LISTERIA INNOCUA]
MSLKPVIGITGNRLVKGVDVFYGHRVTYTQQRYVDAIQKVGGFPIALPIDDPSTAVQAIS
LVDGLLLTGGQDITPQLYLEEPSQEIGAYFPPRDSYEIALVRAALDAGKPIFAICRGMQL
VNVALGGTLYQDISQVETKALQHLQRVDEQLGSHTIDIEPTSELAKHHPNKKLVNSLHHQ
FIKKLAPSFKVTARTADGMIEAVEGDNLPSWYLGVQWHPELMFQTDPESEQLFQALVDES
KKTMVKEGHHHHHH
>1NEPA 130 XRAY 1.70 0.195 0.212 no Epididymal secretory protein E1 <UNP NPC2_BOVIN> [BOS TAURUS]
EPVKFKDCGSWVGVIKEVNVSPCPTQPCKLHRGQSYSVNVTFTSNTQSQSSKAVVHGIVM
GIPVPFPIPESDGCKSGIRCPIEKDKTYNYVNKLPVKNEYPSIKVVVEWELTDDKNQRFF
CWQIPIEVEA
>1XLYA 234 XRAY 1.95 0.206 0.241 no SHE2p <UNP YKNO_YEAST> [SACCHAROMYCES CEREVISIAE]
DIKVTPGTSELVEQILALLSRYLSSYIHVLNKFISHLRRVATLRFERTTLIKFVKKLRFY
NDSVLSYNASEFINEGKNELDPEADSFDKVILPIASMFVKSVETFDLLNYYLTQSLQKEI
LSKTLNEDLTLTAESILAIDDTYNHFVKFSQWMIESLRIGSNLLDLEVVQFAIKSADEDG
TNIGETDNIFLQEILPVNSEEEFQTLSAAWHSILDGKLSALDEEFDVVATKWHD
>1ISPA 181 XRAY 1.30 0.192 0.232 no lipase <UNP LIP_BACSU> [BACILLUS SUBTILIS]
AEHNPVVMVHGIGGASFNFAGIKSYLVSQGWSRDKLYAVDFWDKTGTNYNNGPVLSRFVQ
KVLDETGAKKVDIVAHSMGGANTLYYIKNLDGGNKVANVVTLGGANRLTTGKALPGTDPN
QKILYTSIYSSADMIVMNYLSRLDGARNVQIHGVGHIGLLYSSQVNSLIKEGLNGGGQNT
N
>1H8UA 117 XRAY 1.80 0.234 0.264 no EOSINOPHIL GRANULE MAJOR BASIC PROTEIN 1 <UNP EMBP_HUMAN> [HOMO SAPIENS]
TCRYLLVRSLQTFSQAWFTCRRCYRGNLVSIHNFNINYRIQCSVSALNQGQVWIGGRITG
SGRCRRFQWVDGSRWNFAYWAAHQPWSRGGHCVALCTRGGYWRRAHCLRRLPFICSY
>3LMAA 347 XRAY 1.99 0.170 0.211 no Stage V sporulation protein AD (SpoVAD) <UNP Q65HU8_BACLD> [BACILLUS LICHENIFORMIS]
MKLTGKQTWEFENPLFVNSSGTAVGPKEKEGPLGHLFDKSYDEMHCNQKNWEMAERKLME
DAVQSALSKQNLKKEDIDIFLAGDLLNQNVTANYVARHLKIPFLCLFGACSTSMESIAIS
SALIDGGFAKRALAATSSHNATAERQFRYPTEYGGQKPGTATSTVTGSGAVVLSQQPGGI
KITSATVGRVIDLGITDSQDMGSAMAPAAADTIKQHLEDLGRTPDDYDLILTGDLSGVGS
PILKDLLKEEGINVGTKHNDCGLMIYTPDQQVFAGGSGCACSAVVTFAHIFKEIEAGRLN
RVLVVATGALLSPTIIQQKESIPCIAHGVVFERAERGNALEHHHHHH
>3W54A 329 XRAY 2.30 0.188 0.227 no Alternative oxidase, mitochondrial <UNP AOX_TRYBB>

[TRYPANOSOMA BRUCEI BRUCEI]
MFRNHASRITAAAAPWVLRTACRQKSDAKTPVWGHTQLNRLSFLETVPVVPLRVSDESSE
DRPTWSLPDIENVAITHKKPNGLVDTLAYRSVRTCRWLFDTFSLYRFGSITESKVISRCL
FLETVAGVPGMVGGMLRHLSSLRYMTRDKGWINTLLVEAENERMHLMTFIELRQPGLPLR
VSIIITQAIMYLFLLVAYVISPRFVHRFVGYLEEEAVITYTGVMRAIDEGRLRPTKNDVP
EVARVYWNLSKNATFRDLINVIRADEAEHRVVNHTFADMHEKRLQNSVNPFVVLKKNPEE
MYSNQPSGKTRTDFGSEGAKTASNVNKHV
>1O7JA 327 XRAY 1.00 0.110 0.128 no L-ASPARAGINASE <UNP ASPG_ERWCH> [ERWINIA CHRYSANTHEMI]
ADKLPNIVILATGGTIAGSAATGTQTTGYKAGALGVDTLINAVPEVKKLANVKGEQFSNM
ASENMTGDVVLKLSQRVNELLARDDVDGVVITHGTDTVEESAYFLHLTVKSDKPVVFVAA
MRPATAISADGPMNLLEAVRVAGDKQSRGRGVMVVINDRIGSARYITKTNASTLDTFRAN
EEGYLGVIIGNRIYYQNRIDKLHTTRSVFDVRGLTSLPKVDILYGYQDDPEYLYDAAIQH
GVKGIVYAGMGAGSVSVRGIAGMRKALEKGVVVMRSTRTGNGIVPPDEELPGLVSDSLNP
AHARILLMLALTRTSDPKVIQEYFHTY
>2EHWA 120 XRAY 2.22 0.220 0.284 no Hypothetical protein TTHB059 <UNP Q53WA3_THET8> [THERMUS THERMOPHILUS] MACHELSALRIAIGELLEKEAHDLLHEREELAPVLGQRPELKRLAEAKTLPALEEALREA
LLHLEERAAQEPEEPYWRGLLLAVEAMEGRLKALRAEAEALYQDLDALHGRLHRLFPRRR
>1WDEA 294 XRAY 2.00 0.199 0.244 no Probable diphthine synthase <UNP DPHB_AERPE> [AEROPYRUM PERNIX]
MARGREAVTLLLVGWGYAPGMQTLEALDAVRRADVVYVESYTMPGSSWLYKSVVEAAGEA
RVVEASRRDLEERSREIVSRALDAVVAVVTAGDPMVATTHSSLAAEALEAGVAVRYIPGV
SGVQAARGATMLSFYRFGGTVTLPGPWRGVTPISVARRIYLNLCAGLHTTALLDVDERGV
QLSPGQGVSLLLEADREYAREAGAPALLARLPSVLVEAGAGGGHRVLYWSSLERLSTADV
EGGVYSIVIPARLSGVEEWLLAAASGQRRPLEYDRSVYETVEENCKKGVYMEPV
>2NR4A 213 XRAY 1.85 0.223 0.256 no Conserved hypothetical protein <UNP Q8PVV4_METMA> [METHANOSARCINA MAZEI]
SLIGFLSDRQGFEKQYHDIDLSSFGIREGISEIIASTGFEHPNAAPIGIVMKGERPFVRL
FKGSHTWENVLKEKCLASNVVYDPILFVRSTFSDLVPSEFEYVDAGEFKFPVLKEAIAWV
VFECINLRNTDQSLVADLVPLNAGFNERNIKELPVPNRGFNAVLEATVHATRYQLTGEEK
YLELIRHYESLASKCGGDAEKKAMKLIYEALGL
>2I9CA 123 XRAY 2.00 0.176 0.202 no Hypothetical protein RPA1889 <UNP Q6N8L4_RHOPA> [RHODOPSEUDOMONAS PALUSTRIS]
MSKLDLHQMTTQDLVALFAKVTVEQDDALLGNQISRFNRLFGVMAEIADELKARDGDQRT
ALLSLFEYPNMQVRLQAAKLTLAVAPVKAREQLEAIVSSKWFPQAGDAGMCLDLLDDGTF
KPK
>2EK0A 90 XRAY 1.90 0.229 0.255 no Stage V sporulation protein S (SpoVS) related protein <UNP Q5SK02_THET8> [THERMUS THERMOPHILUS]
METLRVSSKSRPNSVAGAIAAMLRTKGEVEVQAIGPQAVNQAVKAIAIARGYIAPDNLDL
VVKPAFVKLELENEERTALKFSIKAHPLET
>3PHXA 185 XRAY 1.60 0.142 0.192 no RNA-directed RNA polymerase L <UNP L_CCHFI> [CRIMEAN-CONGO HEMORRHAGIC FEVER VIRUS]
GPMDFLRSLDWTQVIAGQYVSNPRFNISDYFEIVRQPGDGNCFYHSIAELTMPNKTDHSY
HYIKRLTESAARKYYQEEPEARLVGLSLEDYLKRMLSDNEWGSTLEASMLAKEMGITIII
WTVAASDEVEAGIKFGDGDVFTAVNLLHSGQTHFDALRILPQFETDTREALSLMDRVIAV

DQLTS
>3LL2A 123 XRAY 0.97 0.144 0.155 no Griffithsin <UNP GRFIN_GRISQ> [GRIFFITHSIA]
SSTHRKFGGSGGSPFSGSGLSSIAVRSGSYLDAIIIDGVHHGGSGGNLSPTFTFGSGEYI
SNMTIRSGDYIDNISFETNMGRRFGPYGGSGGSANTLSNVKVIQINGSAGDYLDSLDIYY
EQY
>2BONA 332 XRAY 1.90 0.222 0.267 no LIPID KINASE <UNP YEGS_ECOLI> [ESCHERICHIA COLI]
MHHHHHHGSTSLYKKAGSETLYIQGMAEFPASLLILNGKSTDNLPLREAIMLLREEGMTI
HVRVTWEKGDAARYVEEARKFGVATVIAGGGDGTINEVSTALIQCEGDDIPALGILPLGT
ANDFATSVGIPEALDKALKLAIAGDAIAIDMAQVNKQTCFINMATGGFGTRITTETPEKL
KAALGSVSYIIHGLMRMDTLQPDRCEIRGENFHWQGDALVIGIGNGRQAGGGQQLCPNAL
INDGLLQLRIFTGDEILPALVSTLKSDEDNPNIIEGASSWFDIQAPHDITFNLDGEPLSG
QNFHIEILPAALRCRLPPDCPLLRSTHHHHHH
>2VDFA 253 XRAY 1.95 0.227 0.272 no OUTER MEMBRANE PROTEIN <PDB 2VDF> [NEISSERIA MENINGITIDIS]
AQELQTANEFTVHTDLSSISSTRAFLKEKHKAAKHIGVRADIPFDANQGIRLEAGFGRSK
KNIINLETDENKLGKTKNVKLPTGVPENRIDLYTGYTYTQTLSDSLNFRVGAGLGFESSK
DSIKTTKHTLHSSRQSWLAKVHADLLSQLGNGWYINPWSEVKFDLNSRYKLNTGVTNLKK
DINQKTNGWGFGLGANIGKKLGESASIEAGPFYKQRTYKESGEFSVTTKSGDVSLTIPKT
SIREYGLRVGIKF
>4NKTA 341 XRAY 1.90 0.180 0.212 no Poly(A) RNA polymerase protein cid1 <UNP CID1_SCHPO> [SCHIZOSACCHAROMYCES POMBE]
GDMSHKEFTKFCYEVYNEIKISDKEFKEKRAALDTLRLCLKRISPDAELVAFGSLESGLA
LKNSDMDLCVLMDSRVQSDTIALQFYEELIAEGFEGKFLQRARIPIIKLTSDTKNGFGAS
FQCAIGFNNRLAIHNTLLLSSYTKLDARLKPMVLLVKHWAKRKQINSPYFGTLSSYGYVL
MVLYYLIHVIKPPVFPNLLLSPLKQEKIVDGFDVGFDDKLEDIPPSQNYSSLGSLLHGFF
RFYAYKFEPREKVVTFRRPDGYLTKQEKGWTSATEHTGSADQIIKDRYILAIEDPFEISH
NVGRTVSSSGLYRIRGEFMAASRLLNSRSYPIPYDSLFEEA
>2HWKA 320 XRAY 2.45 0.208 0.242 no helicase nsP2 <UNP POLN_EEVVT> [VENEZUELAN EQUINE ENCEPHALITIS VIRUS (STRAIN TC-83)]
DVFQNKANVCWAKALVPVLKTAGIDMTTEQWNTVDYFETDKAHSAEIVLNQLCVRFFGLD
LDSGLFSAPTVPLSIRNNHWDNSPSPNMYGLNKEVVRQLSRRYPQLPRAVATGRVYDMNT
GTLRNYDPRINLVPVNRRLPHALVLHHNEHPQSDFSSFVSKLKGRTVLVVGEKLSVPGKM
VDWLSDRPEATFRARLDLGIPGDVPKYDIIFVNVRTPYKYHHYQQCEDHAIKLSMLTKKA
CLHLNPGGTCVSIGYGYADRASESIIGAIARQFKFSRVCKPKSSLEETEVLFVFIGYDRK
ARTHNPYKLSSTLTNIYTGS
>2Q18X 293 XRAY 2.10 0.202 0.219 no 2-keto-3-deoxy-D-arabinonate dehydratase <UNP Q97UA0_SULSO> [SULFOLOBUS SOLFATARICUS]
MKLFRVVKRGYYISYAILDNSTIIRLDEDPIKALMRYSENKEVLGDRVTGIDYQSLLKSF
QINDIRITKPIDPPEVWGSGISYEMARERYSEENVAKILGKTIYEKVYDAVRPEIFFKAT
PNRCVGHGEAIAVRSDSEWTLPEPELAVVLDSNGKILGYTIMDDVSARDLEAENPLYLPQ
SKIYAGCCAFGPVIVTSDEIKNPYSLDITLKIVREGRVFFEGSVNTNKMRRKIEEQIQYL
IRDNPIPDGTILTTGTAIVPGRDKGLKDEDIVEITISNIGTLITPVKKRRKIT
>2JE6B 250 XRAY 1.60 0.216 0.249 no EXOSOME COMPLEX EXONUCLEASE 1 <PDB 2JE6> [SULFOLOBUS SOLFATARICUS]

GHMREMLQVERPKLILDDGKRTDGRKPDELRSIKIELGVLKNADGSAIFEMGNTKAIAAV
YGPKEMHPRHLSLPDRAVLRVRYHMTPFSTDERKNPAPSRREIELSKVIREALESAVLVE
LFPRTAIDVFTEILQADAGSRLVSLMAASLALADAGIPMRDLIAGVAVGKADGVIILDLN
ETEAMWGEADMPIAMMPSLNQVTLFQLNGSMTPDEFRQAFDLAVKGINIIYNLEREALKS
KYVEFKEEGV
>2B06A 155 XRAY 1.40 0.190 0.219 no MutT/nudix family protein <GB AAK75341> [STREPTOCOCCUS PNEUMONIAE] MSRSQLTILTNICLIEDLETQRVVMQYRAPENNRWSGYAFPGGHVENDEAFAESVIREIY
EETGLTIQNPQLVGIKNWPLDTGGRYIVICYKATEFSGTLQSSEEGEVSWVQKDQIPNLN
LAYDMLPLMEMMEAPDKSEFFYPRRTEDDWEKKIF
>2WLRA 423 XRAY 1.45 0.176 0.196 no PUTATIVE THIOSULFATE SULFURTRANSFERASE YNJE <UNP YNJE_ECOLI> [ESCHERICHIA COLI]
MASAELAKPLTLDQLQQQNGKAIDTRPSAFYNGWPQTLNGPSGHELAALNLSASWLDKMS
TEQLNAWIKQHNLKTDAPVALYGNDKDVDAVKTRLQKAGLTHISILSDALSEPSRLQKLP
HFEQLVYPQWLHDLQQGKEVTAKPAGDWKVIEAAWGAPKLYLISHIPGADYIDTNEVESE
PLWNKVSDEQLKAMLAKHGIRHDTTVILYGRDVYAAARVAQIMLYAGVKDVRLLDGGWQT
WSDAGLPVERGTPPKVKAEPDFGVKIPAQPQLMLDMEQARGLLHRQDASLVSIRSWPEFI
GTTSGYSYIKPKGEIAGARWGHAGSDSTHMEDFHNPDGTMRSADDITAMWKAWNIKPEQQ
VSFYCGTGWRASETFMYARAMGWKNVSVYDGGWYEWSSDPKNPVATGERGPDSSKLEHHH
HHH
>2W7VA 95 XRAY 2.30 0.216 0.256 no GENERAL SECRETION PATHWAY PROTEIN L <UNP Q87TC9_VIBPA> [VIBRIO PARAHAEMOLYTICUS] MSGGSTDVAMLSWLAALPATLGQVKDLEITSFKYDGQRGEVRIHARSSDFQPFEQARVKL
AEKFNVEQGQLNRSDNVVMGSFVLKRQLEHHHHHH
>1M2XA 223 XRAY 1.50 0.190 0.208 no class B carbapenemase BlaB-1 <UNP BLAB1_FLAME> [ELIZABETHKINGIA MENINGOSEPTICA]
DVKIEKLKDNLYVYTTYNTFNGTKYAANAVYLVTDKGVVVIDCPWGEDKFKSFTDEIYKK
HGKKVIMNIATHSHDDRAGGLEYFGKIGAKTYSTKMTDSILAKENKPRAQYTFDNNKSFK
VGKSEFQVYYPGKGHTADNVVVWFPKEKVLVGGCIIKSADSKDLGYIGEAYVNDWTQSVH
NIQQKFSGAQYVVAGHDDWKDQRSIQHTLDLINEYQQKQKASN
>3C9UA 342 XRAY 1.48 0.201 0.219 no Thiamine monophosphate kinase <UNP O67883_AQUAE> [AQUIFEX AEOLICUS]
MGSHHHHHHDITSLYKKAGSAAAVLEENLYFQGSFTMRLKELGEFGLIDLIKKTLESKVI
GDDTAPVEYCSKKLLLTTDVLNEGVHFLRSYIPEAVGWKAISVNVSDVIANGGLPKWALI
SLNLPEDLEVSYVERFYIGVKRACEFYKCEVVGGNISKSEKIGISVFLVGETERFVGRDG
ARLGDSVFVSGTLGDSRAGLELLLMEKEEYEPFELALIQRHLRPTARIDYVKHIQKYANA
SMDISDGLVADANHLAQRSGVKIEILSEKLPLSNELKMYCEKYGKNPIEYALFGGEDYQL
LFTHPKERWNPFLDMTEIGRVEEGEGVFVDGKKVEPKGWKHF
>2ANXA 146 XRAY 1.04 0.117 0.150 no Lysozyme <UNP LYS_BPP22> [ENTEROBACTERIA PHAGE P22]
MMQISSNGITRLKREEGERLKAYSDSRGIPTIGVGHTGKVDGNSVASGMTITAEKSSELL
KEDLQWVEDAISSLVRVPLNQNQYDAMCSLIFNIGKSAFAGSTVLRQLNLKNYQAAADAF
LLWKKAGKDPDILLPRRRRERALFLS
>2HLRA 100 XRAY 1.20 0.221 0.229 no Bone morphogenetic protein receptor type-2 <UNP Q91WY9_RAT> [OVIS ARIES] ALCAFKDPYQQDLGIGESRISHENGTILCSKGSTCYGLWEKSKGDINLVKQGCWSHIGDP
QECHYEECVVTTTPPSIQNGTYRFCCCSTDLCNVNFTENF
>4MOMA 756 XRAY 2.19 0.188 0.240 no Putative uncharacterized protein <UNP Q5ZSU4_LEGPH>

[LEGIONELLA PNEUMOPHILA SUBSP. PNEUMOPHILA]
SNAMSTSEKDVREQKVKTVTLSFLGTGQHREKVHHILTSFHNTISEVNKDNPTVAMRMFD
GPGSEPKSGDSKDPIPGTYIYNPKDNSKILISPVISQTITNAIQKLTGNLAGEGIEHLLF
EAVLYLNDIIEKNGGKLPETVNLHGFSRGADTCMRMANLLYQLYPDIKVNLFLIDQVPGP
GKRDDPHSYTVPPNVEHFESTLMLHEYRPGFDPQHSGRYVIADPEKTKVVVKPYYGEHNT
GNRVTEDPNTNHTAILLNDDMNRFCRETGSLPSVGISPPIIARVGDKKEEVRTHSELSPE
KRFELLCGMKENEWGYAKLTKKYHERSILSKREDYVQDSRLFVNQEHRELFKQLYPKSFN
WFFEKNHGGQTKKEEVIVELKSLSEDPRYEHFFSSLAKHFQINENNIAGTLPEPSGIDRD
EKSSFGQPPVRDRLSYLQHSLTSIANYYHYHCDEKSSTNESVKNLLLERVKESRTKPDSE
AIKHLEQTMDEVRQILESKNEKGFLWQQINHISPNARQYCEQVKAALREHLEHNQVLSDT
QKEEIRKAMDRMDNIVNDSSKDSQQKYREIRREVIELNAKATTPEDDNQLTRSHFQKAYF
ELSGDTQKTLNLESLSQTLNQLSKAHYGETSMTDKITQRLDGYKNRNWFWNSVKEVLNFF
NIPLPKLHSEVKEQIADKLKERLVDLKEKGMGNDVNAITRELGKAREDLIEHYKKTSKLE
MGELDKIINKSMEELLVARKVTKDLVHEEVSQVKLN
>3PICA 375 XRAY 1.90 0.195 0.234 no Cip2 <UNP Q7Z9N1_TRIRE> [HYPOCREA JECORINA]
QQTSGAGGATCSALPGSITLRSNAKLNDLFTMFNGDKVTTKDKFSCRQAEMSELIQRYEL
GTLPGRPSTLTASFSGNTLTINCGEAGKSISFTVTITYPSSGTAPYPAIIGYGGGSLPAP
AGVAMINFNNDNIAAQVNTGSRGQGKFYDLYGSSHSAGAMTAWAWGVSRVIDALELVPGA
RIDTTKIGVTGCSRNGKGAMVAGAFEKRIVLTLPQESGAGGSACWRISDYLKSQGANIQT
ASEIIGEDPWFSTTFNSYVNQVPVLPFDHHSLAALIAPRGLFVIDNNIDWLGPQSCFGCM
TAAHMAWQALGVSDHMGYSQIGAHAHCAFPSNQQSQLTAFVQKFLLGQSTNTAIFQSDFS
ANQSQWIDWTTPTLS
>3R45C 81 XRAY 2.60 0.187 0.245 no Holliday junction recognition protein <UNP HJURP_HUMAN>
[HOMO SAPIENS]
SMLGTLRAMEGEDVEDDQLLQKLRASRRRFQRRMQRLIEKYNQPFEDTPVVQMATLTYET
PQGLRIWGGRLIKERNEGEIQ
>1ZIWA 680 XRAY 2.10 0.210 0.248 no Toll-like receptor 3 <UNP TLR3_HUMAN> [HOMO SAPIENS]
KCTVSHEVADCSHLKLTQVPDDLPTNITVLNLTHNQLRRLPAANFTRYSQLTSLDVGFNT
ISKLEPELCQKLPMLKVLNLQHNELSQLSDKTFAFCTNLTELHLMSNSIQKIKNNPFVKQ
KNLITLDLSHNGLSSTKLGTQVQLENLQELLLSNNKIQALKSEELDIFANSSLKKLELSS
NQIKEFSPGCFHAIGRLFGLFLNNVQLGPSLTEKLCLELANTSIRNLSLSNSQLSTTSNT
TFLGLKWTNLTMLDLSYNNLNVVGNDSFAWLPQLEYFFLEYNNIQHLFSHSLHGLFNVRY
LNLKRSFTKQSISLASLPKIDDFSFQWLKCLEHLNMEDNDIPGIKSNMFTGLINLKYLSL
SNSFTSLRTLTNETFVSLAHSPLHILNLTKNKISKIESDAFSWLGHLEVLDLGLNEIGQE
LTGQEWRGLENIFEIYLSYNKYLQLTRNSFALVPSLQRLMLRRVALKNVDSSPSPFQPLR
NLTILDLSNNNIANINDDMLEGLEKLEILDLQHNNLARLWKHANPGGPIYFLKGLSHLHI
LNLESNGFDEIPVEVFKDLFELKIIDLGLNNLNTLPASVFNNQVSLKSLNLQKNLITSVE
KKVFGPAFRNLTELDMRFNPFDCTCESIAWFVNWINETHTNIPELSSHYLCNTPPHYHGF
PVRLFDTSSCKDSAHHHHHH
