# Supplementary material for: Identification of DNA-binding proteins using multi-features fusion and binary firefly optimization algorithm
Source: BMC Bioinformatics. 2016 Aug 26;17(1):323. doi: 10.1186/s12859-016-1201-8 (PMC5002159; doi:10.1186/s12859-016-1201-8)
Supplement: Additional file 2: — The large-scale testing dataset compiled from UniProt. (PDF 477 kb) [file 12859_2016_1201_MOESM2_ESM.pdf]

Additional file 2

Category of DNA-binding proteins: A. thaliana
Number of sequences: 929

Q96524	F4KF14	Q94KL5	P55826	Q9FLW0
P18064	Q9SAJ6	Q9FXG8	Q9C5U3	Q9ASS4
Q8S8P5	Q8VY13	Q6EVK6	Q9SZD4	O48788
Q9XFH4	Q9FY99	Q8S897	Q9SEI3	C0LGG8
Q43125	Q9SEE5	Q9SJJ3	O04019	Q0WR59
Q9S7C9	P25858	F4IVI0	Q9LTX3	Q9FMD7
Q9FFY9	Q1WIQ6	Q9FIM9	Q9SEI4	Q93Y06
Q9SUP6	P27900	Q9ZWC8	Q9MAK9	Q9LRP3
Q9SGY1	O49506	Q9ZPS9	Q9SL67	Q9M8T0
Q9FWX7	Q9SIV3	Q9LZF6	Q9SSB5	Q8VZG8
Q9C8H0	Q93VR3	P54609	Q94BQ2	Q9FM85
Q9C8J8	Q9LYU4	Q38870	Q8H1G6	Q9LVN2
Q9LHD1	Q9LRR9	Q6NLQ6	Q9SEI2	Q9SB61
Q8RWI9	Q9LRS0	P92948	Q9SY73	Q9ZPW7
Q9C9W0	Q9LSV0	Q06850	Q9SSB4	Q9LXG0
Q9M2V5	P93031	Q42438	Q9STF2	Q9FMY7
Q9LJX0	Q9XIP7	Q9ZSA4	B9DFG5	Q9FKP8
Q84M24	Q02283	Q9SCN8	Q9ZU40	Q9SVL0
Q9C8G9	Q94A76	P24100	Q9SSA8	Q9SEZ1
Q9LZ98	Q05466	O80345	Q96529	Q9LQW3
Q9MAG3	Q0WQG8	A8R7E6	P32746	O64722
Q84TH5	Q6STH5	Q9ZV56	Q9SMQ6	Q9FRL5
Q9FIB4	Q9ZVW2	O80842	Q9ZRE2	Q9FIW9
Q8GZ52	P46604	Q8RWC9	Q9CB01	Q9FKJ9
Q9ZUT8	B9DFX7	Q9XIW0	Q38902	Q9M9S0
Q7PC85	Q9SZC9	Q9LZW4	Q8H156	Q9LHF0
Q9LK64	O49596	Q94C40	Q9LUM4	Q949M9
Q9ZUU9	Q9LX66	Q94CG0	Q9LM15	B3H5S5
Q9M9E1	P93047	Q93VD3	O23657	Q9LPD0
Q9SW08	Q42344	Q84VQ3	P28188	Q9T074
Q9SYI3	Q9C9W5	F4JY24	P31582	Q9ZW35
Q9SIT6	P51818	Q0PCS3	O80501	Q9SNB4
Q8VZZ4	Q9LTT3	Q9SN43	Q8W3M3	Q9FE20
Q9STT5	O49597	P25859	Q9FJN8	Q9FNP1
Q9ZU35	O03986	P16127	Q9SIP0	Q8RY16
Q9FLX5	F4JFN3	Q9SIW2	P92963	Q9SSL9
Q9FLT5	Q9LVW2	O22932	Q38937	Q9LV48
Q9M1C7	O49595	Q5XF33	Q9SN68	O49562

Q01593	Q9SGS2	Q9FI56	P41916	Q9MAC9
Q9SZE1	Q43386	Q9SUL7	Q7G1L2	Q8W1X2
Q9FKF2	P27323	Q9C562	Q9SW63	Q9FFW5
Q9C8H1	Q42525	Q9SEZ7	P28185	Q9M076
Q8RXN0	P41151	Q9LP51	Q38922	Q7X9V2
Q9C8K2	Q8LDF9	O80902	Q6J9S1	Q5YGP8
Q9SKX0	P55737	Q8W1D5	P28187	Q9S775
Q7FB56	Q9SIF2	Q8LF80	P92978	O64682
Q9LSJ6	Q8RWZ3	Q8VYJ7	Q9SMR4	Q9C6T1
O80946	P93834	Q9ZUL5	Q9SK03	Q8S9J1
Q9C8T1	Q8VZG2	Q94BR5	P82280	Q5YGP7
Q9LFG8	Q9LPS1	Q9LYQ8	Q9SL02	P83483
Q7XA72	Q8LPJ5	Q2V452	P28186	P48731
Q9LSJ2	P41376	Q9LEU7	Q8LC30	P46668
Q9FUT3	Q9SA34	O65554	Q8H1E4	P83484
Q9S725	Q56XE8	Q9MAM1	P42736	O64629
Q9SMT7	Q9SCT4	Q93Z18	Q9FJR0	Q9M077
Q9SH69	F4J6F6	Q9SXJ7	Q9FME0	Q38897
Q9FFR3	P41377	P42762	Q38912	P46667
Q9M2V6	Q9CAI7	Q9STV4	Q38919	Q8RWU4
Q9LSJ5	Q9C5S2	O22971	P41917	Q94F62
Q3EDJ0	Q9SUG3	P92937	P10896	Q1PFD1
Q9ZR72	O80568	Q9FJ55	Q9SD82	Q9SW80
Q9M3B9	Q9FK35	Q9FJ54	Q9FNM7	Q9SIW1
Q9M1Q9	Q93YN9	Q9LDI3	Q93ZG7	Q9M2Z1
Q93YS4	O81893	P42730	Q9SQV1	Q9FWS9
Q3E9B8	O82514	Q9LF37	Q9LH76	O49545
Q9LVM1	Q93VJ2	Q9FY74	Q9LPG6	Q9SJ56
Q9ZUT0	Q8RWY3	Q6NPP4	Q8H136	O65685
Q9LFH0	Q9SBA5	Q8GSA7	P47924	Q9LZM8
Q9M0D0	Q9FKP4	Q9LHE4	Q6NLQ7	O65440
Q7PC81	Q9LX99	Q8LPI7	Q8LA13	O49397
Q9STT8	P42158	O23463	Q9SYM5	P46897
Q7DM58	Q9SWG0	Q9LSP8	Q9CAM7	Q940D0
Q8LPT1	P48001	Q9FIL7	Q9M2F9	P92549
Q9CAF5	Q9FPQ8	Q9FYG2	Q9FN89	Q9ZPY9
Q9LK62	P46640	Q8VZJ9	Q9LVI6	Q11207
Q8LPK0	Q9LDN0	O49564	Q9FFH1	Q93V99
Q9M0M2	P46639	Q9LDT0	Q8W4J9	Q9M7Q7
Q8H1R4	Q84JS6	Q9LDN1	Q42484	Q84WU8
Q9M3D6	P92958	Q9XEC7	O64973	Q8VZI8
Q94FB9	P48000	Q9CAL2	F4HY56	Q8L743
Q9XF19	P42818	Q9FNE1	Q8L840	Q8VYG2

Q9M0G9	Q38997	Q9C5S8	Q39214	Q9M092
Q8RY46	Q8L7Y8	Q9M9Y8	Q9M667	Q9SJK6
Q8LPQ6	P48002	Q9ZP16	Q9FT70	Q9C5T3
Q94A18	P43288	Q0PW40	Q9SJT1	Q93WU9
Q6NLC1	Q9SJD4	Q8L710	Q9LK36	O04292
Q9FLT4	Q940B8	O65472	Q8GYD9	Q9FXD6
Q9MAH4	Q39011	Q8W4G6	Q9SKB2	Q9ZWJ9
Q9FLT8	Q38933	Q8RX80	Q39203	Q07970
Q9C7F8	Q9CAP8	Q9LMB9	O04130	Q8LAH7
Q9LZJ5	Q9M9L8	O65479	O81905	Q8GYB8
Q9M2V7	F4I3V3	Q6NQ87	Q8RWZ5	Q9SGW8
Q9LK50	Q00958	O64817	D8WUA4	Q9FUP0
Q9FT51	P93655	O65476	Q01474	O82533
Q84K47	Q9M9L7	Q8GWJ7	O82663	Q9M0V6
Q8LPJ4	F4HQ17	Q3E9X6	Q9ZPX5	Q42545
O81016	F4KA50	Q9T0J1	Q9SYI0	Q9FKW6
Q7PC86	F4HQ23	Q8S9L6	Q9XIC7	Q9C9L5
Q9XIE2	Q9SJF1	Q9LDS6	Q9XGX0	Q9S9M5
Q7PC84	Q9S7U9	Q9LDQ3	Q8L607	Q9S9M2
Q1PEH6	Q39008	Q9SYS7	O24629	Q8RY17
Q9SYI2	Q9SFB6	Q8L7G3	P52839	C0LGH2
O80725	Q9LFH9	O65483	Q9SKG5	O04567
Q9M1H3	P0C5E2	Q9LYU7	Q9M1T1	Q944A7
Q9STT7	F4HQ22	Q9ASQ5	Q9M8S6	Q9FHD7
Q9LV93	D7SFH9	Q8GYA4	Q9C9C9	Q8RYC8
Q9STT6	Q94CK9	Q8H199	Q9FII7	Q9C8N9
Q9FNB5	Q9SHS8	O65482	Q8L7L1	Q9LP07
Q8LGU1	Q94A06	O65405	Q9SYX2	Q9SZX3
P06525	Q9FF31	Q9LDM5	Q9FJL0	Q9ZPX1
Q9LYS2	F4KA51	Q9XEC8	Q9FZ80	O04291
Q8LEF6	F4HQ20	Q9SYS3	Q39192	Q66GP9
Q9FWX8	Q3ECH2	Q9LZU4	Q39193	Q9SB48
Q9C7F2	Q9LSR8	O65468	Q9SD40	Q9SLG0
Q9C6W5	Q9M9E0	Q9FIU5	Q9LQZ5	P18616
Q9LSJ8	Q9C6K9	Q08467	F4KH89	Q9M8Z5
Q9FJH6	Q8VYG0	Q08466	Q56YN8	Q9C923
Q0WML0	Q9LJD8	O64816	Q9LUL4	Q9S7E4
Q9FF46	Q9LV58	P27450	Q940H6	Q9S9P8
Q9LZB8	Q9SJI8	Q9M0X5	P43292	Q9SCZ4
Q8LPK2	O22042	Q9CAL3	Q38874	Q8W493
Q42093	P93819	Q9XEC6	O22558	Q9FGM0
Q7PC88	Q8RXG3	O23081	Q9C9D0	Q9LXJ0
Q7PC87	Q9FZ36	Q9C5S9	Q9M2U4	Q9LN59

Q8H0V6	Q9LYG3	O80574	Q8RWL6	Q9S9M3
Q7PC83	Q9LPD9	P84634	F2Y4A3	O22176
Q7PC82	Q9FL33	Q93Y16	Q8RWZ1	Q9STX0
Q7GB25	P23686	Q3EBC8	Q9C5P1	Q9ZNT0
Q9FHF1	Q9LXT3	Q38953	Q9C8M9	Q0WNY5
Q9LHK4	Q0WVF5	Q8VYV7	Q9SI19	Q9XI90
Q9SZR9	O80786	Q9SP32	Q9FXH7	O80462
Q8L7L5	Q9FT73	Q9LXW7	P37107	O80623
A1L4X7	Q9LUT2	Q9M5K2	O65517	C6KIE6
Q9SJG4	Q8W3L1	O82497	Q9SJT8	C0LGN2
O22130	F4KAB8	Q9FNY3	Q05153	Q0WTB4
Q8GXB3	P57106	Q8H1F6	F4HW65	O23661
Q9LID6	Q9LKA3	Q9SYS6	Q9C5P4	P93024
Q8LPN5	Q8LCU7	Q8S3C9	F4IHS2	Q9FGV1
Q9LTA2	P43299	Q9SE83	O23627	Q9SKN5
Q9SR17	Q9SJL8	P93835	F4JTP5	Q9ZPY6
Q8GWQ2	Q9ZP06	O82132	Q9C5P0	Q84K00
Q0PGJ6	Q9MBA2	Q9FHI7	Q9LZV4	Q9SKT7
Q9M338	P17562	Q9M5K3	Q9M084	Q9ZNT1
Q96329	Q39023	Q9M0L0	Q9SSK1	Q94BV7
Q940I0	Q9C6D2	P42697	Q9SW96	Q6NPS8
O23620	O24617	Q9LQ55	Q9ZPI1	P33207
O49658	Q9SMV7	Q8LFT2	P38558	O49213
Q9FIR1	Q39024	Q9T0J6	Q9AT00	Q9LMU0
Q9FW44	Q39026	Q9FV70	P38557	Q9MB58
Q9M9R4	O04716	P0DI16	Q9LK35	Q8W035
Q9SB31	Q9LK95	Q84XF3	Q56WH1	Q9C5T4
Q4V3E0	Q9SZP1	F4HPR5	B9DHQ0	Q9SB92
O80834	Q9FE25	Q05762	Q9SLF3	Q9C516
Q38898	F4I460	Q8RWL0	Q9LUS2	Q9C8M5
Q9SCX5	F4IRU3	Q9FNY2	O81283	Q9S9M1
Q56YU0	Q0WPU1	O82133	Q8H0U5	Q9SEI0
Q9SZ70	Q9M2K0	Q9FHJ2	F4IW47	Q9LZV6
Q9LVB0	F4K5J1	Q8LF21	Q9FYK0	Q9SXB8
O49662	F4HXP9	Q9LQK0	Q8RWV0	C0LGJ1
Q9C9K7	F4HYV5	Q9CAI1	O23680	Q9ZQ70
Q9FHM5	Q9C8W9	Q9SVA6	Q6S5G3	Q9LQE8
Q84TF0	Q9LKG8	F4IBE4	P43298	Q84WU6
Q8GXE6	O04017	Q84WD3	Q9LK43	Q9LSD6
O22812	F4HWY6	Q8VZC7	P30182	Q9FKT0
Q9M2S3	F4JIU4	Q8W4H7	Q9M2X3	Q84N64
Q9LZX7	Q9LKB9	Q8GTY0	Q9FJW5	Q38851
Q8VYJ2	F4I5Q6	Q9ZT91	Q9CA28	F4IVR7

O82166	Q9SCL7	Q9LW09	Q9SIT1	F4JM19
Q6DBQ1	Q9FNV8	Q9SSR8	Q8VWK4	F4K0A6
Q38998	Q96276	Q9FNX5	Q6R0E3	Q9LD44
Q8L7G0	Q9LTF7	Q8LSZ4	Q8L7L8	Q9FYK5
O80452	F4IUG9	P0DH99	Q38906	Q9C6I6
Q38914	Q9LHE9	Q9C641	Q9SNB9	Q8VY00
P36397	Q9ZNU2	O23116	Q9C7B1	Q9SAD4
Q9ZTX9	Q9SL41	Q9FNY0	Q9M347	O80340
Q9ZTX8	Q9LR74	Q9LFQ9	Q9FJA2	Q9LMT9
Q9FX25	Q9FWX2	Q9ASR1	B5X582	Q39191
Q93YR9	Q8H1Q2	Q9FV71	O65041	Q8VYA3
Q94JM3	Q9FH59	Q9FPR3	P93028	O04336
Q9XID4	Q9FNV9	Q9STX5	Q9M336	O22921
Q9LQE3	Q38850	Q0WL56	Q9C7W7	Q9C519
Q9C5W9	Q9ZTC3	Q94CF0	O64700	Q9XID3
Q9C7I9	O65508	B3LF48	Q9STX2	Q94A68
Q9C8N7	Q9SPG9	O82653	Q9LZI2	Q9LUL2
P93022	Q9LXV2	F4IW10	Q7X8C5	Q8GXQ3
Q9XED8	Q9SSA1	Q8LDC8	Q9SA25	

Category of DNA-binding proteins: Fruit fly
Number of sequences: 143

P15348	A4VCL2	P51023	P22058	Q9VGW6
P28159	Q8SWV6	P52304	P08181	Q9V461
Q24492	P18106	P48601	Q24210	Q00449
P23625	P02835	O18413	Q9VBW6	P24785
P16378	P25157	P08646	Q9VBW9	P04197
Q7KU24	Q8MT06	P08645	Q9VCU9	Q9W3C1
P20353	Q05337	O18334	O76324	Q27571
Q9W252	Q9VEI9	P04388	P19109	P10676
P20354	Q02748	P35600	P23128	P54351
P00522	Q24208	P53034	Q24533	Q9VPD2
P29673	Q02280	Q94523	P07548	P46461
P45888	Q9V3I5	P30052	P49762	Q9VJI9
P40945	Q9V4L4	Q27324	P23023	P16554
P40946	P54367	Q9VWQ2	O77051	Q95RR8
Q9VW15	P81900	Q9VK34	P11536	P31368
Q7JWD3	P83099	P32031	P08736	P35875
P35381	P05130	Q9VL52	Q27368	P17917
Q05825	P13678	P32030	P20105	O97143
Q9W0K4	P91660	Q9VF26	P45447	P51022
P09081	P17210	Q9U5L1	P40427	P40797
Q9VC57	P46863	P28668	Q9VM92	Q9VSJ5
Q9W0K7	O61267	P06606	P09052	A8JNJ2
Q9VGI8	Q9W590	P06603	P20153	Q9VZV3
Q9VNP5	Q7KUT2	P61857	Q9VA38	
P08630	Q9VNS0	P06605	Q95T10	
P23573	Q26454	P06604	P28167	
Q9VT57	Q00748	Q7KN62	P28166	
Q9NGC3	P49735	O77277	Q7KV83	
O16102	Q9XYU1	Q9NBK5	Q8IRE7	
O77059	Q9XYU0	P20659	Q9I7T8	

Category of DNA-binding proteins: S. cerevisiae
Number of sequences: 314

Q12154	P16521	P28742	P07272	P13574
P22336	P36041	P38691	P33298	Q03497
P11938	P32324	Q06147	Q01939	P12866
P06786	P32476	P13186	P53131	P32916
P25847	P38071	P36004	P33299	P38699
P08539	P38137	P34253	P20095	P36057
P01123	P35731	Q12246	P53549	P32597
P26754	P41813	P53145	P40327	P25302
P07271	Q04433	P36775	P21372	P04802
P06780	P00360	P30665	P33297	Q07478
Q03834	P00359	P29496	P47116	P08153
P40433	P00358	P53091	P31374	P32944
P39925	P14065	P29469	P41909	P40340
P00330	P43535	P38132	P34230	Q02457
P00331	Q12263	Q12019	P80210	P18412
P43633	P15442	O14467	P31244	P36145
P25377	Q08726	P36060	P12753	P42938
P46367	P32836	P33310	P25454	Q03533
P54115	P32835	P24279	P38953	P09733
P32789	P02309	P33311	P01119	P09734
P38116	P47122	P07884	P39729	Q01852
Q02804	Q03532	P19358	P19541	P23254
P32381	P10823	P53166	P14736	P22515
P11076	P41911	P32333	P22216	P52488
P05085	P42950	P25336	P32863	P38820
Q12386	P38970	P47047	P32849	P26370
P53974	P25364	P25846	P38623	P38735
P49090	P51979	P47069	P06838	P18562
P40467	P0CE41	Q05473	P01120	P22219
P53104	Q12753	P0CY06	P53879	P21576
P40416	P34243	P28737	Q08904	Q04500
P40471	Q04458	P0CY08	P25611	Q06685
Q01389	P0CY07	P53128	Q03195	P52917
P14772	P0CY11	P32492	P38766	P36017
P32639	P02829	Q04439	P38712	P39961
P43569	P34244	P38626	Q01163	Q12222
P23293	P32485	P19524	Q12464	P14680
Q03941	P33416	P36006	P38990	P39109
P32468	P0CY09	P38830	P20606	P36086

P27636	P31539	P16603	P39954	Q12152
P32457	Q08732	P25293	P18759	P12688
P25342	Q05549	Q03435	P39955	P53049
P32458	P32481	P52920	P11792	P48559
P09119	P53685	Q08972	P46954	Q99260
P40558	P10081	P11633	Q08491	P43591
P19073	P06168	P36003	Q99287	P38146
P25694	P38697	Q12181	P07560	P51996
P32797	P04807	P11632	Q00416	P36018
P32562	P53115	P38219	Q07657	P32939
P22516	P38991	P40010	P35207	Q04116
Q03654	P05986	P39720	P32908	Q12244
P32657	P32361	P36023	P47037	P34161
P27895	P17119	P15873	P06700	P40341
P27697	P39962	P13259	Q03656	P38555
P15790	P22517	P29468	P36048	
P19454	Q06554	P38126	P35187	
P22204	P23292	P33751	P20424	
P24783	P07278	P08018	P38128	
P32328	P15454	P12383	P39073	
P20449	P28743	P33302	P32364	
P06634	P23291	P00560	P22082	
P20448	P25389	P17157	P41901	
P39009	P14681	P16862	P25808	
P09624	P13185	P52960	P12954	
P02994	P06242	Q12236	P50104	

Category of DNA-binding proteins: Mouse
Number of sequences: 424

P31266	Q03963	Q60751	O35942	Q60855
Q9Z2D6	Q9QXB9	Q8BK63	Q8BU40	P51448
P60766	Q99MU3	P08414	A2AI05	P51450
Q9R1E0	Q9Z2R9	Q9CXV0	Q02780	Q6NTA4
P02340	P56931	Q9D2Z8	P97863	Q80X95
P00520	Q8R0K9	P68181	Q8R4B8	Q99K70
Q60611	Q61502	Q9DC28	Q8C6J9	A2A825
Q3UHJ0	Q58FA4	O35066	Q8CIZ9	Q6IMB1
Q61102	P00375	P28738	P70255	Q9WTM5
P50544	O35261	Q9WV04	P70257	O35488
B5X0E4	O54917	P05532	Q60641	Q91VE0
Q9JJ59	Q01279	Q62120	O88942	Q60714
Q61271	P39053	P05132	Q60591	P60122
Q8VHH7	Q61501	Q8BTH8	Q3UP24	Q9JHW4
Q4JIM5	Q6S7F2	Q8C4X2	Q8K120	Q8VDQ8
Q8K440	P10126	Q52KG5	Q99MA9	Q62231
Q61288	Q61527	Q9QWT9	P42582	Q61321
P31750	Q9EQY0	P31324	Q8BVE8	P70178
Q8BXK8	Q03145	Q04447	P97305	A2AKX3
P19091	Q61772	P28867	O54949	Q91ZW3
Q8BXL7	O09127	P68404	P29477	P42208
P84078	P54763	Q62101	Q3UV55	Q923E4
Q8CG76	Q8BHK9	Q8BZ03	Q64249	Q8R216
Q640N2	Q8CBF3	P0C605	Q2LKU9	Q9WVC6
P61208	P41163	O35071	Q91WS2	Q8R104
Q99PE9	Q8CHI8	Q61768	Q9Z0J4	Q8CG48
O54984	P70424	Q02111	Q60644	Q8CG46
P70704	P81270	P18654	P46460	Q9CW03
Q925I1	P15037	P06151	O88708	Q8CG47
P27040	P27577	B1AVY7	Q640Q5	Q924W5
O70126	P41164	P48025	Q8CGY8	P53783
Q61687	P16092	P06240	P41778	Q9QYY8
Q91YE5	O89106	Q6ZPI3	P17918	P17433
P79621	Q8K2I9	Q8CGK3	Q8CIN4	Q62232
P28028	P34152	P63006	Q61036	P48432
Q5RJI5	Q9QVP9	P25911	O88554	Q9CU62
O88700	P11276	Q5S006	Q8CEE6	Q8CI15
P36895	P16879	Q9WU40	Q3UA06	P53784
Q9Z1S0	P35583	P49717	P11103	Q811W0

P35991	Q64732	P97311	Q69Z38	P05480
Q69Z98	P58462	P08923	Q8CIW5	O70551
P49615	Q8BPY9	Q99MV5	P09041	O88697
P11440	P35582	Q9EQJ9	P06803	Q80TF6
Q8R3L8	P35584	Q8VHJ5	Q99LC9	Q9WTK7
Q99J95	Q9WVH4	Q9R1L5	Q07832	O55098
P30658	Q61080	P21440	Q60806	Q8CGC7
Q6A068	P16858	P49138	P70268	P99024
P27790	P06537	Q2KHI9	Q64702	Q14BI7
P40201	Q3V3I2	P14152	Q9JHG7	O70306
Q9Z265	Q8K349	P06795	Q99MQ3	O35144
P26361	P20612	Q60954	Q922R0	Q01853
O70496	O70443	P97367	O88685	Q9EPZ6
P97377	P36916	Q8BVN9	Q61136	Q8C0Y1
O35491	Q9DC51	Q63943	P54775	O35857
A2A8L1	Q6R0H7	Q8CIP4	P62196	P68372
Q91XQ5	P30678	P49718	Q8R4E6	Q9CWU0
Q9QUP4	P21278	P21447	Q64434	Q64163
P22518	P30677	Q3UMW7	P63011	O55047
Q9D5U8	P63094	Q9CWV1	O35295	Q08639
O35280	Q6PGG6	P97310	Q3TMX7	A2ASS6
Q9R194	P20029	P32442	Q9CQD1	Q01320
P41241	Q8R316	Q8CFN5	Q99N57	Q9ER41
P09581	P62806	Q9JI70	P15919	Q64511
Q60737	Q9QZR5	Q62504	P62492	Q8CGV9
P97784	P70349	Q9WTU6	P56371	Q68FE9
O70589	P30681	Q9WUI1	P63001	Q3UVR3
P56546	O54879	Q9Z1B7	E9Q9D5	Q80UG8
P53564	Q8R1H0	Q8BG51	O08674	A2APC3
O54833	P17095	P47811	Q08297	Q925K9
Q8VDF3	P42581	Q8CE90	Q61411	Q02053
Q91VR5	P17156	Q7TT50	P32883	Q3U3Q1
Q62167	P63017	Q9WUI0	Q8VEA8	Q80X41
Q61496	Q61696	P23249	P43352	Q8BN21
Q61655	P63158	Q9R1X5	Q62193	Q8VEJ9
Q9JJY4	P17879	Q91Y86	P08556	P46467
O70133	P16627	O08911	P35546	Q8VCS3
Q9ESV0	P09022	Q99MT2	P35601	P70121
Q810A7	Q91VC3	Q9R1S7	Q9WUK4	P43404
Q8BG36	Q60680	P13705	Q99J62	Q9R0G7
Q99J87	O88351	Q61006	P48377	Q8C0C0
P70397	Q62406	Q64331	P48379	Q6NZP1
Q61880	Q9R0T8	Q02566	Q8VEE4	E9PZI6

Q8BYH0	P81067	P09922	P48381	H3BKF3
Q9JHU4	P70671	Q8VDD5	Q9D0F6	E9QKG2
P70396	P15208	P97479	P62746	

Category of DNA-binding proteins: Human

Number of sequences: 1049

P04637	P17844	P14735	P23511	Q92963
P78363	Q53GQ0	Q92985	Q96PY6	Q9Y572
P27694	Q9Y394	O75874	P51955	P52198
Q9P2D1	Q8N5I4	P38919	Q13469	O75116
P13569	Q8IY37	O15111	Q9NPP4	P51449
Q16539	Q9C098	P06213	Q7RTR0	P61587
P10275	Q9BTZ2	P78415	Q12857	Q04912
O95477	Q08211	P55010	Q7RTR2	Q8WZA2
P54274	Q9UIK4	Q14164	Q9Y239	Q5VZM2
Q15554	O43237	Q13418	O95803	Q9NQL2
Q8WWZ7	Q8N8A6	Q00978	Q9HC98	Q92730
Q8IZY2	Q96C10	O14920	Q14934	Q13464
Q8WWZ4	Q9NZQ0	P20839	O00712	P35398
Q86UK0	P35659	Q9NWZ3	Q8N4C6	P08922
P33897	Q7Z5P4	Q08881	Q9C000	Q01974
P61221	P56937	O60674	Q86SG6	O95398
P00519	Q9BPW9	Q9ULG1	Q8TD19	Q9Y4G8
O00763	Q9NUU7	Q9Y3D8	Q12968	Q9HB90
Q2M2I8	Q9UMR2	P13861	Q14938	P10301
Q9NUT2	Q08345	Q9NYR9	P04629	Q7L523
Q9NRK6	Q9NR30	P48730	Q96RI1	P62070
Q09428	O00571	P49674	P78426	Q9BU20
P28288	Q9H0S4	P08069	Q9UBE8	Q9Y230
Q9UG63	Q9NQI0	P51617	P29475	O00442
Q5FVE4	Q9BQ39	P78368	P46459	P19793
Q8N139	P26196	Q9Y6M4	Q8TB37	Q5K4L6
O94911	P09417	Q6PHW0	Q5SY16	Q6T310
Q8IUA7	Q6UX07	Q9HCP0	P35228	Q96HN2
Q9UBJ2	Q7L2E3	P12532	O96028	Q9NR31
Q9UNQ0	Q9H2U1	P46013	P20393	Q9NZ71
Q9H172	Q9UPY3	P23458	Q9NTK5	Q9UBT2
Q9H222	Q9Y5R6	P52333	Q9Y5Y2	Q9Y3Z3
Q96GR2	Q8NBQ5	P17612	Q13415	Q9Y6B6
Q9BZC7	O43143	Q13557	Q9H244	Q9UPW6
O75027	O60231	Q9ULD8	O60285	Q9NVA2
O95342	P55265	O60341	O60313	Q9Y265
Q8NE71	O60479	Q9Y496	Q96RQ9	P57772
Q9NUB1	P49366	P33176	O15294	Q9UHD8
P01011	O43812	P31323	Q13177	Q9Y6X0

Q07912	Q8TE73	Q9NYS0	O75914	Q7Z333
Q9NR19	Q9C0G6	Q8N752	Q9NQU5	Q01826
Q9H2P0	Q9NYC9	P48729	O95747	Q53H47
Q96CM8	O95057	Q9BZL6	Q9P286	Q9NTG7
Q96P47	P50570	Q04759	Q5VST9	Q9Y6E7
Q99758	O00429	P10644	Q9UGN5	Q8N6T7
Q86UQ4	Q9Y295	Q9UQM7	Q86U86	Q15477
Q2M3G0	Q6IAN0	Q15058	Q15645	Q7Z7L1
Q9NP58	Q01094	Q12756	Q96RG2	Q15019
Q9NP78	Q15329	O60333	P12004	O00141
O60706	O75461	Q03164	Q13153	Q8NDV3
O14678	Q96AV8	Q15139	Q58A45	P28370
P45844	P19525	Q02156	O43252	Q9H0K1
P42684	Q9P2K8	Q15418	P09874	Q8IXJ6
Q4G176	A0AVK6	Q9H1H9	P40424	Q8N196
O60503	O60869	Q9NQT8	O15530	O60264
Q9BRQ8	P68104	Q96L93	P40425	Q9H4L7
O60218	O00418	Q96Q89	O43933	Q9NXA8
Q04828	Q13627	Q9ULI4	P51160	Q14683
Q7Z591	Q9UBX2	P52732	Q9NTI5	Q9UQE7
Q8NI60	O00148	Q02241	Q13608	P41225
O43306	Q9P225	Q05655	Q9H792	O43175
P17516	Q92630	P41743	Q96BD5	Q9Y2K2
Q5T2L2	Q16254	P14618	Q9BXM7	Q9NRC8
P31749	Q13838	Q8NCM2	P17858	O00570
P31751	Q9P2D7	O00139	Q96RR1	Q9UBP0
Q9Y243	Q8WXX0	Q12840	P09619	P17947
P53396	Q14204	O60282	P11309	Q9UIU6
P36896	Q9BQI3	Q2M1P5	Q9BVI0	P51532
Q08462	Q9UQ16	P04183	P48736	Q969G3
O60266	O00716	Q8NEZ4	Q15126	Q96EB6
Q9UPQ3	Q9NZN3	Q13976	Q99453	P42285
O94788	P32519	Q9P2E2	Q504Y2	P23497
Q9H161	Q14209	Q8N4N8	Q9WJR5	P38935
Q6IQ32	P13639	O15066	P63133	O95347
P49189	Q9NZN4	Q9BW19	Q16512	Q9NTJ3
Q9UM73	P28324	Q9BVG8	P53350	Q8IY18
Q99490	Q96L91	Q96RR4	Q12837	Q96SB8
O14727	Q15375	Q05513	Q9UQG0	P12931
P42330	P29322	Q96JN0	Q01851	Q08945
P00352	Q5T890	Q7Z4S6	P10266	Q15772
P49419	P18074	O43896	Q9BXR3	Q9NYA1
P61204	Q03468	P10721	Q07869	Q6ZRS2

P18085	P00533	Q9UMN6	P37231	P08240
P84085	Q04743	P05771	P63136	O43704
P15121	Q2NKX8	Q15349	P63135	Q9P2P6
P84077	P07992	P51812	P63132	P48431
Q13795	O00757	O95835	O75360	P35712
P56559	Q15723	P00338	Q9NYY3	Q05066
P62330	P78545	Q9UJU2	Q9H4B4	O00338
P40616	Q99607	P43405	P35998	Q8WU08
P10398	P21860	P07195	Q13523	Q9NRA0
Q9Y4B4	Q15303	Q9UPM6	Q9ULL5	Q15208
Q9Y689	Q76MJ5	O75449	P54821	Q9Y5M8
Q96BM9	P41161	Q9NRM7	P62333	Q9Y2H1
Q3SXY8	P41212	P53671	P43686	Q96SB4
Q68CP9	O75460	Q6ZMQ8	P62195	Q13043
Q9NVJ2	P11474	Q8IWU2	Q92620	O94804
Q9NVI7	P03372	P36776	P17980	Q15831
P36404	P14921	Q86WA8	Q92786	Q9Y6E0
P49703	P15036	Q38SD2	P62191	Q9NRP7
P00966	P43268	Q99683	Q13882	Q9C0K7
Q9NR48	Q8IXL6	Q96Q04	P31939	Q06330
O43681	P62508	Q5S007	Q15257	Q15046
Q8NBU5	Q14289	Q9H089	Q96QR8	Q9P289
Q96QE3	Q8IYD8	Q9Y2U8	O00391	Q9UEW8
O43488	P49327	Q9BXT6	O15315	P14868
P36405	P16591	Q12851	P51153	P07814
P40617	P02751	O60307	P57729	P13984
Q9H0F7	Q16875	Q9Y6R4	P20337	Q5JPH6
Q9Y2Q0	P07332	O95819	P61020	O75478
Q5T9A4	Q05397	Q5TCQ9	Q9NRW1	Q71U36
Q9ULI0	Q6PIW4	O15021	O15067	Q7L7X3
Q8WXF7	Q9UPW0	P25205	Q9UJV8	P04350
Q9P241	Q9H334	P33993	O43502	P68371
Q9UIF9	Q9BVA6	P07948	Q9H0T7	P07437
Q9NPZ5	P55317	Q8IVH8	P61019	Q9UGU0
Q9BUT1	P55318	Q96QZ7	Q15771	Q9H2K8
O94766	Q12948	P49137	P63000	Q03518
Q8NFC6	Q16676	P27448	P11233	Q9UL54
P54132	Q12947	Q5TCX8	O75771	Q9UHD2
O14965	Q08050	P49736	P61026	Q14186
Q96GD4	Q12778	P33992	O14966	Q9GZN2
Q8WY36	P06241	P08235	P51151	P68366
Q14692	P11362	Q96JY0	O75943	Q9NRH3
P46100	P22607	Q16644	Q92878	Q15583

Q13705	P11413	Q14566	Q92698	P10827
P51813	Q12952	Q9UJA3	P11234	Q03519
P33076	Q12946	Q9NU22	Q9Y272	Q16594
O60566	P55316	Q02080	P61106	Q587J7
Q06187	P04150	Q14814	P62820	P55072
O43683	Q9Y261	P29376	Q9ULC3	P10828
P15056	Q12950	Q9P0L2	P51157	Q8NDG6
Q9NSY1	Q12951	Q7KZI7	Q13637	P35590
Q8TDC3	Q01167	Q96L34	P20336	Q02763
Q8IWQ3	O43524	P51608	Q86YS6	Q9UKE5
P11586	P55040	Q6P0Q8	P51148	Q00059
Q13873	P04406	P33991	P15918	Q14188
Q14781	P32455	Q9NXL9	Q6IQ22	Q9NWX6
P21127	Q9H0R5	Q9Y2H9	Q9ULW5	O94842
Q9BWU1	P32456	P08183	P20339	Q17RP2
P06493	Q49A26	Q02078	P20340	O94900
Q00534	Q96PP9	O00470	Q9H0N0	Q96KB5
P50750	Q96PP8	P50222	P61006	O14656
P60953	P38405	Q8IXI2	P10114	O43615
Q9NYV4	Q03113	P08581	Q9Y3L5	Q86UE8
P50613	P30679	P40925	P62491	P31314
P07199	P63096	P21439	O00194	O14657
Q02224	P11488	Q06413	Q9H082	Q8WZ42
Q99459	A8MTJ3	O14770	Q96S21	Q9Y2W1
O94921	P19086	Q8IWI9	Q9NP72	Q8N2E6
P24941	Q9NVN8	Q9H2W2	Q9BZG1	P11388
Q00535	Q9HCN4	Q8IWA4	Q15286	Q02880
P49336	Q9H9Y4	Q8TDZ2	P20338	Q5JU69
Q96G23	Q8WTQ7	P28482	P51149	Q6ZSZ6
Q12873	P29992	P45983	Q3YEC7	Q63HK5
Q14839	O95837	Q15759	P15153	Q9BXA6
Q14004	P09471	Q96T58	Q14088	Q5TCY1
O14647	P50148	P53778	Q6ZRP7	Q6IQ55
O14757	P63092	Q9HCE1	P57735	P33981
Q9UQ88	P19087	O94851	Q13636	O95922
O96017	P36915	Q7RTP6	Q9NP90	Q6EMB2
Q96RK0	P11021	Q8IXI1	Q06609	A6PVC2
P51793	P08754	P53779	P04049	Q16881
P51798	Q5JWF2	P43246	P62834	Q86VQ6
P49761	Q9BVP2	Q92887	O14807	Q14679
O14646	Q9UHW5	O15438	P01111	P29597
Q8TD26	Q969Y2	O95255	P51159	O75643
Q9HCK8	A4D1E9	P27361	Q9UBG7	P17480

Q3L8U1	Q14344	P45984	P43351	Q9BWV7
P51790	P04899	O15264	P62826	Q3SXZ7
Q92989	P32298	Q9NPJ1	P61224	P22314
O94983	P08631	Q9HBH9	Q15907	Q15029
Q8TDI0	P62805	O14733	P0C0E4	Q8TBC4
Q7L1S5	P48637	Q9BT17	Q92900	Q8IYT8
Q9HAZ1	Q9Y450	O15439	Q6NUM9	P21796
Q9Y259	Q9BYK8	Q96J65	P10276	O75385
Q9NRB3	P49773	P20585	P01112	O43314
Q7LFX5	Q9P1Z3	Q9Y2G1	P01116	P30530
Q9Y6Y1	Q9H422	P33527	Q8WXH6	Q6PHR2
P49759	Q00839	O15440	Q9UBK7	Q8NBZ7
P49760	P20823	Q5T3U5	Q8TC12	Q99986
Q9H078	Q14527	P52701	Q8TAI7	Q9BYP7
Q14028	P17096	Q9H4K7	P35250	O75351
Q96CD2	Q9UGU5	O75648	P48378	Q9H4A3
A5YM72	Q9Y663	P35580	Q8NBN7	Q9Y3S1
O43186	Q3SXM5	P35749	P40937	P35968
Q49AN0	P09429	Q86UW6	P48380	Q6PFW1
P68400	O15347	B0I1T2	Q15382	O75717
P41240	Q8IX15	Q9ULV0	P08134	Q9UN37
Q13363	Q9BPY8	B2RTY4	P57078	Q86Y07
Q8IWT3	P0DMV9	Q9H0A0	P61586	Q9NZC7
P19784	P26583	P35579	Q99578	A3KMH1
O14936	P34931	O00159	Q9UBZ9	O75063
O14529	P17066	O94832	P15927	Q14191
Q16526	Q86Z02	Q13402	P35251	O75695
P07333	Q6NT76	Q9Y623	P35249	Q9H6S0
O14578	Q9ULV5	Q9UJ70	P22670	Q9C0A1
P04839	Q9NYD6	A7E2Y1	O00212	Q15911
P39880	P51553	Q9UM54	Q15669	Q9H4I2
Q92841	P11142	Q9NX02	P17081	P43403
Q9UHI6	Q9UPZ9	Q9UHQ9	Q7L0Q8	Q9Y6X8
Q5T1V6	Q2VIR3	O94916	O94762	Q5FWF4
Q9Y6G9	P41091	P08651	Q96D21	Q8IYH5
O15075	Q03933	P20591	Q13546	Q96TA2
Q86XP3	P54652	P20592	O94844	P37275
Q9NXZ2	O60841	Q9Y2K3	P62745	O60315
Q7L014	P0DMV8	Q8WX94	P84095	Q9UKY1
Q8NHQ9	P31276	Q8IVL1	Q9BYZ6	Q86UP3
Q9NY93	P12268	Q9UHB4	O43353	O43167
O95786	P09629	O95644	Q9ULI2	
